# Visible-Light-Induced Bond Homolysis in Titanacyclopentadienes for the Catalytic Cyclodimerization of Internal Alkynes

**DOI:** 10.1021/jacs.5c13492

**Published:** 2025-09-30

**Authors:** Maxi L. Heldner, Tobias Körner, Corinna Czernetzki, Patrick T. Geppert, Agnieszka Nowak-Król, Gabriele Hierlmeier

**Affiliations:** † Institute of Inorganic Chemistry, 9190 Julius-Maximilians University of Würzburg, Am Hubland, 97074 Würzburg, Germany; ‡ Institute for Sustainable Chemistry & Catalysis with Boron, 9190 Julius-Maximilians University of Würzburg, Am Hubland, 97074 Würzburg, Germany

## Abstract

Ligand-to-metal charge transfer (LMCT) processes offer significant potential in photochemical synthesis but remain comparatively underdeveloped, relative to metal-to-ligand charge transfer (MLCT) pathways. Titanium organyls are particularly promising in this context, enabling the direct generation of carbon-centered radicals. However, reports on their photochemistry have remained scarce. Herein, we present the visible-light-induced homolytic cleavage of a Ti–C bond in a titanacyclopentadiene complex supported by a bulky pyridine-diamido ligand. The resulting biradicaloid undergoes a selective skeletal rearrangement via a H atom shift to form a titanacyclopentene. This stoichiometric transformation was translated into a novel catalytic cyclodimerization of internal alkynes. With 2-butyne, the unusual dimer 1,2,3-trimethyl-4-methylenecyclobutene, a strained and readily functionalizable small ring, was obtained quantitatively. Mechanistic investigations of this previously unreported catalytic transformation including quantum chemical calculations, single-turnover experiments, quantum yield determination by actinometry, and reaction kinetics reveal key features of the underlying reactivity and indicate that visible-light-induced Ti–C bond homolysis is the rate-determining step. Overall, our findings demonstrate that the combination of a sterically demanding ligand, which suppresses alkyne cyclotrimerization, with visible-light-induced bond homolysis offers a new entry point into catalytic alkyne oligomerization chemistry.

## Graphical abstract

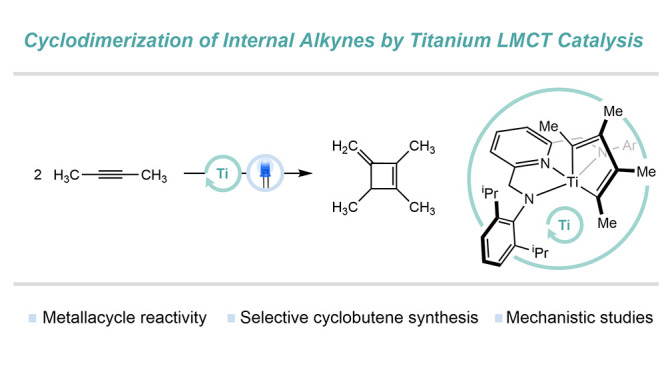

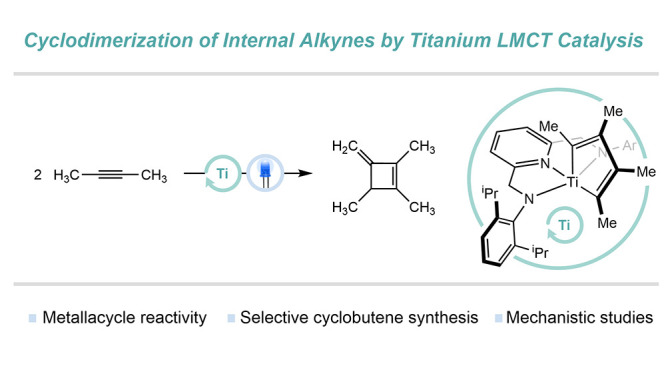

## Introduction

Charge transfer processes are central to the reactivity of photoactive metal complexes. Among these, ligand-to-metal charge transfer (LMCT) remains underutilized compared to the well-established metal-to-ligand charge transfer (MLCT) processes.^1^ However, LMCT states offer access to fundamentally distinct reactivity that cannot be achieved through MLCT. In this context, complexes of group IV metals with strongly donating ligands are ideal candidates to study LMCT processes, due to the electron-deficient nature of the electropositive metal centers.

Within the group 4 triad, excitation of LMCT transitions typically initiates reactivity through two primary pathways: excited-state electron transfer (ES-ET) and visible-light-induced bond homolysis (VLIH) (Figure 1A). Milsmann and co-workers reported a notable example of the former reactivity mode with zirconium complexes capable of ES-ET (**A**, Figure 1B).^2,3^ Exceptional long excited-state lifetimes in the microsecond range were achieved by tuning the electronic properties of the bis­(pyridinedipyrrolide) ligand. Notably, thermally activated delayed fluorescence (TADF) is the source of the luminescence of complex **A** and can be harnessed for diverse photoredox catalytic applications. Apart from this prominent example of LMCT-induced ES-ET, visible-light-induced homolysis is observed in a considerably larger number of group IV metal complexes, particularly those based on titanium. Most of these studies and catalytic applications are based on Ti–X (with X = Cl, OR; Figure 1C) bond homolysis and subsequent reactivity of the generated radicals in H atom transfer (HAT) reactions, as, for instance, reported by Kanai and Mitsunuma,^4^ Schelter,^5,6^ or in β-scission, as reported by Zuo.^7^ In contrast, the generated Ti­(III) complex can also be of synthetic use, as demonstrated by Gansäuer and co-workers.^8,9^

**Figure 1:**
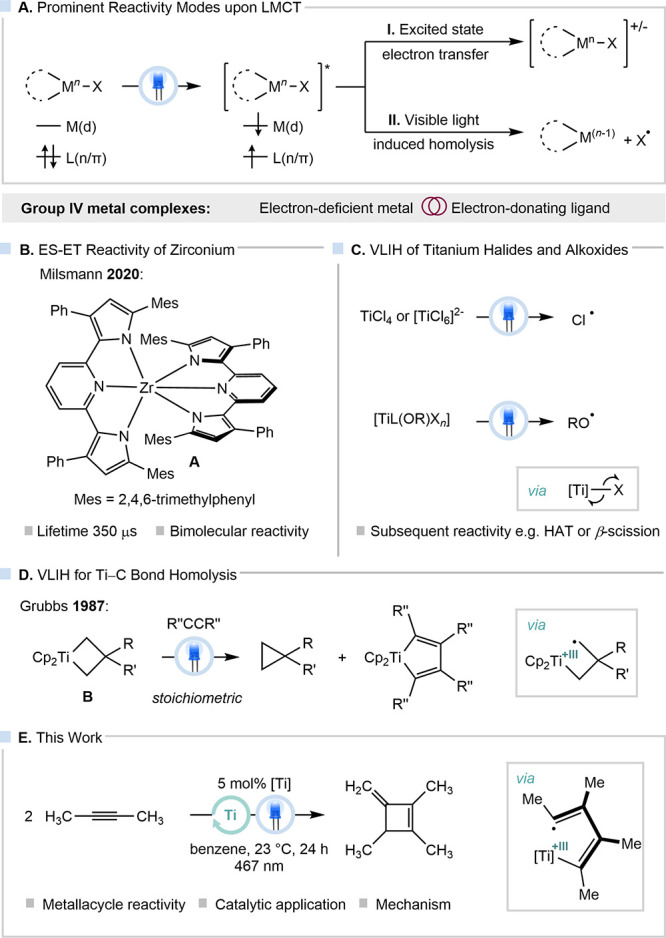
(A) LMCT-induced reactivity with group IV metal complexes; (B) ES-ET chemistry with a zirconium photosensitizer; (C) selected examples for LMCT-induced Ti–X bond homolysis; (D) Ti–C bond homolysis in a titanacyclobutane and subsequent reactivity; and (E) VLIH in a titanacyclopentadiene for the catalytic synthesis of a cyclobutene.

While homolysis of Ti–X (X = halide, alkoxide) bonds is well-established upon LMCT, photochemical cleavage of Ti–C bonds is not as widely encountered in the literature.^10^ A notable example is the photochemistry of titanacyclobutanes (**B** in Figure 1D) with alkynes, as studied by Grubbs and co-workers.^11^ Upon irradiation of complex **B** in the presence of suitable trapping reagents, the formation of stoichiometric amounts of cyclopropane and the corresponding trapping product, e.g., titanacyclopentadienes, was observed. Studies of the stereochemistry of the α-carbon are consistent with the formation of a 1,4-biradical, as evidenced by the loss of stereochemical information in the product. Lastly, photoinduced reductive elimination promoted by LMCT has also been reported for complexes of the type Cp_2_MR_2_ (M = Ti, Zr, Hf; R = alkyl, aryl, alkynyl).^12,13^ Bidentate pyridyl-pyrrolide complexes of zirconium were also employed in the stoichiometric formation of an η^4^-cyclobutadienyl zirconium complex upon photolytic bibenzyl elimination.^14^

Given the lack of methods for Ti–C bond homolysis and the synthetic potential of carbon-centered radicals,^15^ there is strong motivation to explore such LMCT-induced VLIH processes. Herein, we present our investigation of the VLIH of a pincer-supported titanacyclopentadiene, which undergoes a selective rearrangement of the carbon skeleton of the metallacycle upon irradiation. This reactivity was translated into a catalytic process, leading to the discovery of a novel and selective protocol for the cyclodimerization of 2-butyne to a methylenecyclobutene derivative, a strained small molecule, obtained in quantitative yield. Detailed mechanistic studies, including stoichiometric reactions and reaction kinetics, support a pathway involving a titanium–carbon biradical intermediate that facilitates catalytic C–C bond formation.

## Results and Discussion

### VLIH of a Titanacyclopentadiene

Considering the lack of reports on the reactivity of titanacyclopentadienes under photochemical conditions and the well-documented loss of cyclopentadienyl radicals upon irradiation of titanocene systems,^16^ the photochemical reactivity of the recently reported pyridinediamido (PDA)-supported titanacyclopentadiene was investigated.^17^ (^iPr^PDA)­Ti­(C_4_Me_4_) (^iPr^PDA = 2,6-(2,6-iPr_2_C_6_H_3_NCH_2_)_2_C_5_H_3_N^2–^, **1-C**_**4**_ **Me**_**4**_) was obtained from the reaction of the corresponding dibenzyl complex (^iPr^PDA)­Ti­(CH_2_Ph)_2_ (**1-(CH**_**2**_ **Ph)**_**2**_) and 2-butyne according to a recently published protocol.^18^ The UV–vis absorption spectrum of **1-C**_**4**_ **Me**_**4**_ in benzene features two broad absorption bands at 340 and 480 nm. Time-dependent DFT (TD-DFT) calculations were conducted to simulate these spectra and elucidate the origin of the electronic transitions. Calculations using the B3LYP and TPSSh functionals in combination with def2-TZVP as the basis set show reasonable agreement with the experimental spectra (see Figure 2A and SI Figure S24). The lowest-energy excitations at 464 and 451 nm were found to originate predominantly (98%) from a filled molecular orbital of the carbon skeleton of the titanacyclopentadiene to an empty orbital on the titanium pyridine moiety, consistent with a ligand-to-metal charge transfer process (B3LYP, Figure 2B). These preliminary findings suggest that the titanium–carbon bond within the metallacycle might be labile under blue light irradiation.

**Figure 2:**
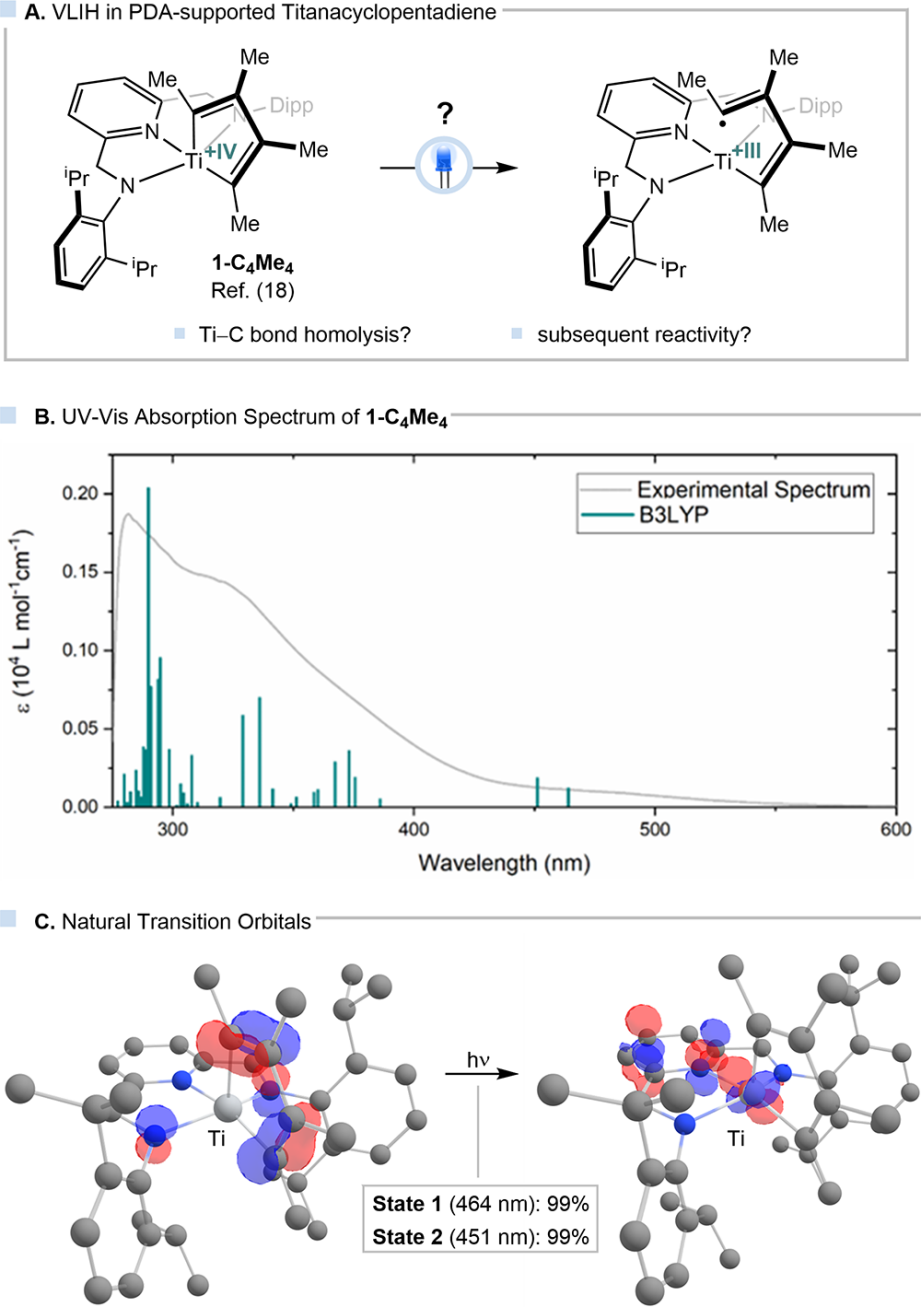
(A) VLIH in a pyridinediamido-supported titanacyclopentadiene; (B) experimental and computed UV–vis absorption spectra; and (C) natural transition orbitals (NTOs) of lowest-energy transitions using B3LYP as a functional.

Based on both the absorption spectrum and theoretical insights, irradiation of a solution of **1-C**_**4**_ **Me**_**4**_ in benzene with blue light (λ = 467 nm) was investigated. Under these conditions, the complex underwent selective conversion (95% NMR yield) to the new titanacyclopentene (^iPr^PDA)­Ti­(CH_2_C_7_H_10_) (**1-CH**_**2**_ **C**_**7**_ **H**_**10**_) (Figure 3, A), which was isolated from the reaction mixture in quantitative yields. The ^1^H NMR spectrum of **1-CH**_**2**_ **C**_**7**_ **H**_**10**_ exhibits two new doublets with chemical shifts at 5.37 and 4.85 ppm (^2^ *J* = 21.4 Hz) for the CH_2_ protons of the PDA ligand and a new characteristic singlet at 2.14 ppm, attributed to the titanium-bound methylene. The molecular structure of **1-CH**_**2**_ **C**_**7**_ **H**_**10**_ in the solid state is shown in Figure 3B and reveals a rearrangement in the metallacycle carbon skeleton. This is evidenced by the single bonds between the C­(sp^3^) carbon atom (C32), and C33 (1.499(2) Å), and an exocyclic double bond (C33–C36:1.347(2) Å).^19^ The latter bond length is comparable to that of the internal titanacycle double bond C34–C35 (1.358(2) Å). Structurally related mononuclear metallacyclopentenes featuring an exocyclic C=C double bond are scarce, with one example of a tantalum complex reported by Schrock and co-workers obtained by the (thermal) rearrangement of a tantalacyclobutene.^20^ Hence, the rearrangement of **1-C**_**4**_ **Me**_**4**_ to **1-CH**_**2**_ **C**_**7**_ **H**_**10**_ is a rare example of selective photochemical metallacycle reactivity.^21^ Notably, even though the rearrangement of **1-C**_**4**_ **Me**_**4**_ to **1-CH**_**2**_ **C**_**7**_ **H**_**10**_ results in the loss of π-conjugation within the metallacycle, the overall reaction is thermodynamically favorable, with a calculated Gibbs free energy of –6.3 kcal·mol^–1^ (B3LYP, def2-TZVP level of theory). **1-CH**_**2**_ **C**_**7**_ **H**_**10**_ is photochemically inert under irradiation at 370–467 nm.

**Figure 3:**
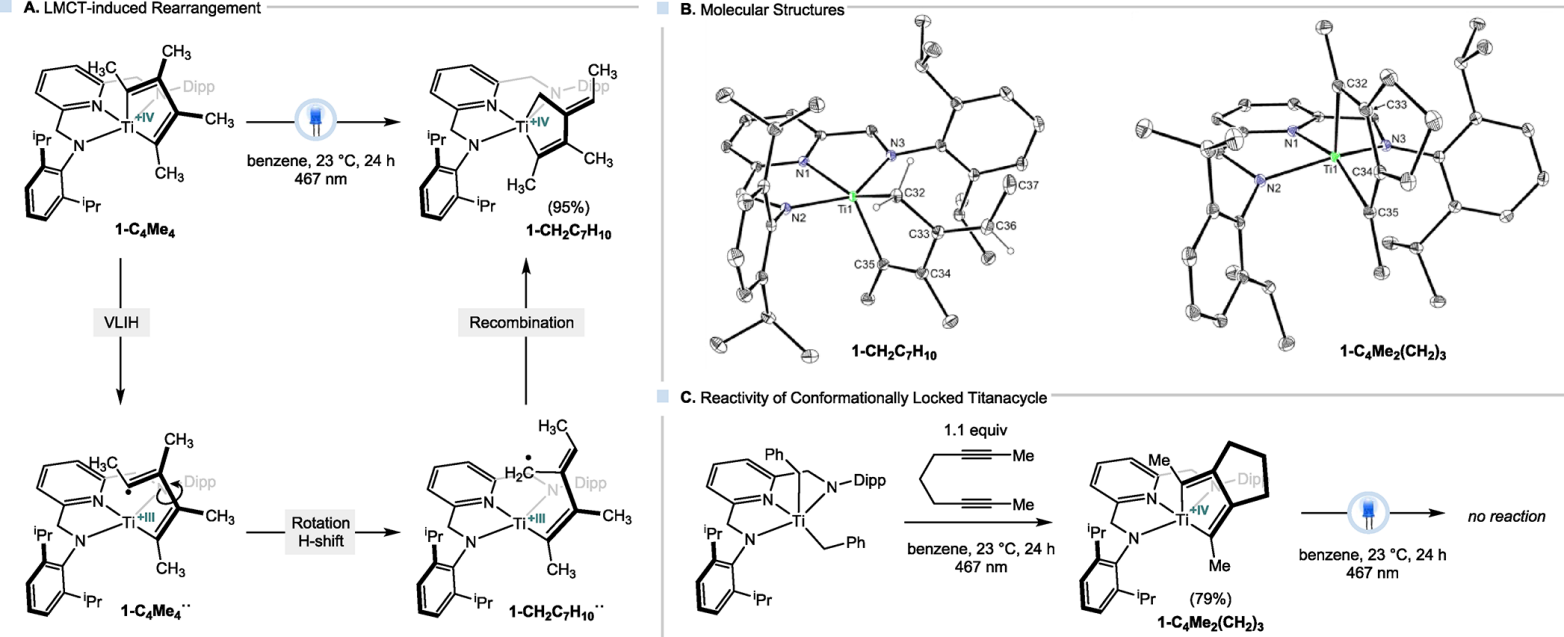
(A) Photochemical rearrangement of a titanacylcopentadiene; (B) molecular structures of **1-CH**_**2**_ **C**_**7**_ **H**_**10**_ and **1-C**_**4**_ **Me**_**2**_ **(CH**_**2**_ **)**_**3**_ in the solid state with 30% probability ellipsoids, H atoms except for selected atoms on the metallacycle are omitted; and (C) synthesis and photochemical reactivity of conformationally locked titanacycle.

In light of the LMCT suggested by the TD-DFT calculations, a plausible mechanism for the rearrangement of the titanacyclopentadiene begins with homolytic cleavage of the Ti–C bond (VLIH), generating **1-C**_**4**_ **Me**_**4**_ **^··^** (Figure 3A). This proposed biradicaloid is related to a previously isolated 1-titanacyclobuta-2,3-diene by Reiß and Beweries, which exhibits contributions of a structure with biradicaloid character, containing a Ti­(III) center and a monoanionic radical ligand in its ground state.^22^ Furthermore, this reactivity is in line with previous reports by Grubbs and co-workers on the isomerization of titanacyclobutanes upon irradiation.^11^ Considering the instability of the generated vinylic radical,^23^ the subsequent transformation likely proceeds via C–C bond rotation, followed by a hydrogen shift to form the intermediate **1-CH**_**2**_ **C**_**7**_ **H**_**10**_ **^··^**,^24^ and radical recombination to afford **1-CH**_**2**_ **C**_**7**_ **H**_**10**_. The formation of the rearranged titanacycle **1-CH**_**2**_ **C**_**7**_ **H**_**10**_ thus requires both a C–C bond rotation and a hydrogen shift. To probe this mechanistic pathway, the structurally related titanacyclopentadiene **1-C**_**4**_ **Me**_**2**_ **(CH**_**2**_ **)**_**3**_ was synthesized by the photochemical reaction of **1-(CH**_**2**_ **Ph)**_**2**_ with nona-2,7-diyne and isolated in 79% yield (Figure 3C). Single crystals suitable for X-ray diffraction were obtained from *n*-hexane and confirm the formation of a novel metallacycle in which C–C bond rotation around the C33–C34 bond is hindered. Inspection of the NTOs of **1-C**_**4**_ **Me**_**2**_ **(CH**_**2**_ **)**_**3**_ reveals that the lowest-energy transitions strongly resemble those in **1-C**_**4**_ **Me**_**4**_ (see Figure S22 in the SI). Despite these close electronic and structural similarities to **1-C**_**4**_ **Me**_**4**_, no reaction was observed upon irradiation of a benzene solution of **1-C**_**4**_ **Me**_**2**_ **(CH**_**2**_ **)**_**3**_ at 467 or 427 nm for 24 h. This lack of reactivity suggests that C–C bond rotation is a necessary step in the photochemical rearrangement of **1-C**_**4**_ **Me**_**4**_ and further indicates that the subsequent hydrogen shift occurs after this rotation.^25^

### Catalytic Cyclodimerization

According to McConville and co-workers, PDA-supported titanacyclopentadienes do not undergo catalytic alkyne (3-hexyne, diphenylacetylene, and trimethylsilylacetylene) cyclotrimerization under thermal conditions.^17^ Considering that the LMCT-induced bond homolysis in **1-C**_**4**_ **Me**_**4**_ generates an open coordination site on the titanium center, the intermediate **1-C**_**4**_ **Me**_**4**_ **^··^** may exhibit additional reactivity in the presence of other coordinating reactants. Given that selective *in situ* formation of **1-C**_**4**_ **Me**_**4**_ from **1-(CH**_**2**_ **Ph)**_**2**_ has been previously established,^18^ 20 equiv of 2-butyne were added to a benzene solution of **1-(CH**_**2**_ **Ph)**_**2**_ (5 mol %). The reaction mixture was irradiated at 467 nm for 24 h (Table 1, entry 1). Gratifyingly, the reaction proceeded quantitatively with catalytic generation of a single organic product (>95%). Analysis of the ^1^H NMR spectrum showed two characteristic vinylic signals indicative of an exocyclic double bond at chemical shifts of 4.50 and 4.59 ppm. Closer inspection of the ^1^H, ^13^C­{^1^H}, as well as ^1^H–^13^C-HSQC and -HMBC NMR spectra (SI Figures S30–S36) revealed the formation of 1,2,3-trimethyl-4-methylenecyclobutene (C_8_H_12_, Table 1), which has been previously observed in attempts to generate tetramethylcyclobutadiene.^26^ 1,2,3-Trimethyl-4-methylenecyclobutene was isolated from the reaction mixture as a stock solution in benzene quantitatively or as a neat substance in 80% yield when the reaction was conducted in toluene and the dimer was distilled off.

**Table 1:**
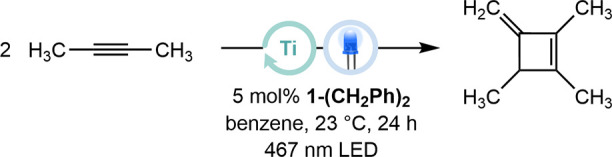
Variation of Reaction Conditions in the Titanium-Catalyzed Cyclodimerization of 2-Butyne^b^

entry	deviation from standard conditions	conv.[Table-fn t1fn3]	yield[Table-fn t1fn3]
1	none	100%	>95%
2	no **1-(CH**_**2**_ **Ph)**_**2**_	0%	0%
3	^iPr^PDAH_2_ instead of **1-(CH**_**2**_ **Ph)**_**2**_	0%	0%
4	no light, rt	0%	0%
5	no light, 60 °C	0%	0%
6	**2-(CH**_**2**_ **Ph)**_**2**_ instead of **1-(CH**_**2**_ **Ph)**_**2**_	57%	9%
7	[Cp_2_Ti(η^2^-Me_3_SiCCSiMe_3_)] instead of **1-(CH**_**2**_ **Ph)**_**2**_	90%	0%
8	Ti(CH_2_Ph)_4_ instead of **1-(CH**_**2**_ **Ph)**_**2**_	89%	0%
9	**1-C**_**4**_ **Me**_**4**_ instead of **1-(CH**_**2**_ **Ph)**_**2**_	95%	85%
10	**1-CH**_**2**_ **C**_**7**_ **H**_**10**_ instead of **1-(CH**_**2**_ **Ph)**_**2**_	0%	0%
11	LED intensity 25% instead of 50%	85%	78%

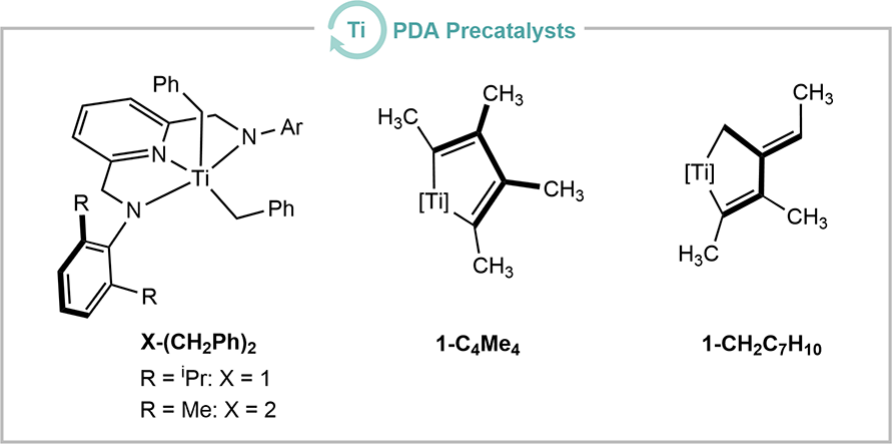

a Standard conditions: 0.37 mmol 2-butyne, [Ti] = 0.05 mol/L, 50% Kessil lamp intensity.

b Conversion and yield were determined by ^1^H NMR spectroscopy using HMDSO as internal standard.

Catalytic dimerization of terminal alkynes to enynes or cumulenes is well-precedented in the literature.^27^ In contrast, internal alkynes usually undergo trimerization to arenes.^28^ While the stoichiometric cyclodimerization of alkynes to form cyclobutadienyl complexes is well-established in the literature,^29^ and the stoichiometric release of alkyne dimers from metal complexes has also been established,^30^ catalytic cyclodimerizations of internal alkynes are almost unprecedented. A single related catalytic reaction was reported by Hashimoto and co-workers, who used a nickel hydride catalyst to cyclodimerize 3-hexyne.^31^ However, only 36% yield of the cyclodimer was obtained, along with three additional alkyne trimers including cyclopentadienes and hexaethylbenzene. Moreover, the reaction required harsh conditions of 120 °C.^32,33^ Thus, the quantitative formation of 1,2,3-trimethyl-4-methylenecyclobutene by photoinduced titanium catalysis presents a novel approach in alkyne oligomerization chemistry.^34,35^

While initial cyclodimerization experiments using **1-(CH**_**2**_ **Ph)**_**2**_ and 2-butyne showed quantitative formation of the dimer, further conditions and catalysts were tested to elucidate the role of the titanium complex and reaction conditions. In the absence of **1-(CH**_**2**_ **Ph)**_**2**_ or in the presence of the preligand ^iPr^PDAH_2_ alone (entries 2 and 3), no conversion or product formation was observed. Similar findings were obtained when irradiation was omitted or substituted with thermal activation (60 °C) (entries 4 and 5). Hence, entries 2–5 indicate that both the titanium complex and irradiation are essential for catalytic cyclodimerization. Next, steric effects of the ligand were examined by replacing the *iso*-propyl group on the amido moieties with a methyl group (**2-(CH**_**2**_ **Ph)**_**2**_; entry 6). Surprisingly, this minor modification significantly reduced both the product yield and selectivity.^36^ Using Rosenthal’s complex [Cp_2_Ti­(η^2^-Me_3_SiCCSiMe_3_)] ^37,38^ resulted in conversion of the alkyne but gave a complex mixture of oligomers including hexamethylbenzene (entry 7). A similar outcome was observed for Ti­(CH_2_Ph)_4_ (entry 8). In contrast, the isolated titanacyclopentadiene **1-C**_**4**_ **Me**_**4**_ (entry 9) demonstrated catalytic activity comparable to that of **1-(CH**_**2**_ **Ph)**_**2**_, with formation of the cyclobutene in 85% yield. Employing the rearranged metallacycle **1-CH**_**2**_ **C**_**7**_ **H**_**10**_ under catalytic conditions did not lead to any conversion or product formation. These results clearly indicate that **1-CH**_**2**_ **C**_**7**_ **H**_**10**_ is not a competent precatalyst or intermediate in the reaction, but rather the catalyst deactivation product formed in the absence of alkyne (*vide infra*). Lastly, reduced light intensity (25% instead of 50%) afforded lower yields, indicating that the reaction rate is dependent on the light intensity (entry 11).

### Mechanistic Experiments

Given that these results represent a highly unusual catalytic and selective cyclodimerization of an internal alkyne to a methylenecyclobutene, the mechanism of this reaction was studied in detail by a series of stoichiometric experiments and reaction kinetics. First, single-turnover experiments using **1-C**_**4**_ **Me**_**4**_ were conducted (Scheme 1A). Upon irradiation (467 nm) of a benzene-*d*_6_ solution containing the metallacycle **1-C**_**4**_ **Me**_**4**_ and two equivalents of 3-hexyne, the formation of **1-C**_**4**_ **Et**_**4**_ was observed along with one equivalent of the cyclobutene. In contrast, when the sterically demanding alkyne bis­(trimethylsilyl)­acetylene was used under otherwise identical conditions, only the rearranged metallacycle **1-CH**_**2**_ **C**_**7**_ **H**_**10**_ was formed (*vide supra*). These results support the conclusion that alkyne coordination is essential for catalytic turnover and demonstrate that steric hindrance can impede this step, leading to catalyst deactivation. To probe ground-state interactions of **1-C**_**4**_ **Me**_**4**_ with 2-butyne, UV–vis and EXSY NMR experiments were conducted. The UV–vis absorption spectrum of **1-C**_**4**_ **Me**_**4**_ in benzene showed a slightly increased signal intensity in the presence of 2-butyne, indicating a possible light-harvesting function of the alkyne (SI Figure S3). In contrast, EXSY NMR at −40, 25, and 75 °C did not reveal any new signals and cross-peaks between the methyl groups of the alkyne and those of the titanacycle, indicating no chemical exchange of 2-butyne with **1-C**_**4**_ **Me**_**4**_ (SI Figures S50–S52). These findings suggest that alkyne coordination likely occurs directly after Ti–C bond homolysis.

**Scheme 1:**
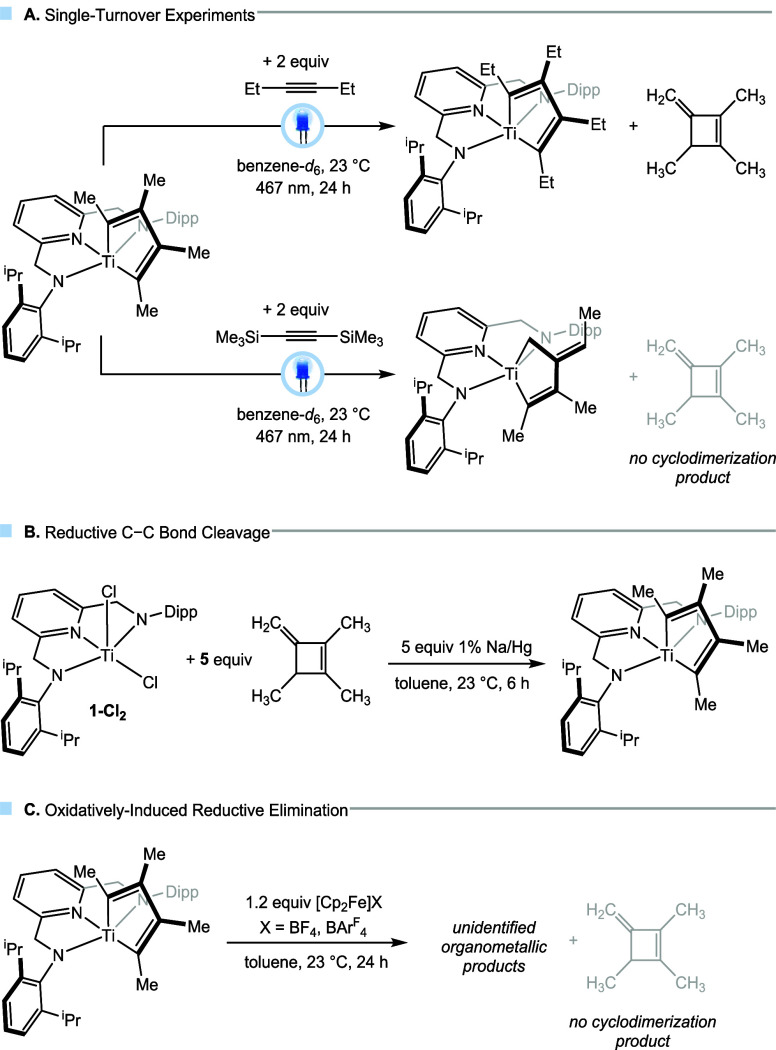
Stoichiometric Mechanistic Experiments; (A) Single-Turnover Experiments, (B) Reductive C–C Bond Cleavage, and (C) Oxidation of **1-C**_**4**_ **Me**_**4**_

To test the reversibility of C–C bond formation under reductive conditions, isolated methylenecyclobutene was reacted with (^iPr^PDA)­TiCl_2_ (**1-Cl**_**2**_) and 5 equiv of 1% Na/Hg (Scheme 1B). Interestingly, the formation of **1-C**_**4**_ **Me**_**4**_ was observed under these conditions, indicating C–C bond cleavage under reductive conditions. Importantly, a control experiment confirmed that the methylenecyclobutene does not form 2-butyne in the presence of sodium amalgam (see Supporting Information and Figures S55–56). Lastly, to probe the possibility of product release upon oxidation (oxidatively induced reductive elimination), 1.2 equiv of ferrocenium salts (FcBAr^F^_4_, BAr^F^_4_ = tetrakis­[3,5-bis­(trifluormethyl)­phenyl]­borate and FcBF_4_) were added to a benzene-*d*_6_ solution of **1-C**_**4**_ **Me**_**4**_. However, this reaction resulted only in the unselective formation of several organometallic species, with no cyclodimer observed in the resulting ^1^H NMR spectrum (Scheme 1C and SI Figures S57 and S58).

To assess whether the reaction proceeds via a photoinduced long-lived radical chain mechanism, a light-on/light-off experiment was conducted using *in situ* LED-NMR spectroscopy (Figure 4A).^39^ The resulting data clearly show that the reaction occurs only under irradiation, indicating that it is driven exclusively by light. However, such experiments are not sensitive to short-lived radical chain processes.^40^ Therefore, to further probe the mechanism, chemical actinometry was performed to determine the overall quantum yield of the catalytic cyclodimerization reaction. Potassium reineckate (K­[Cr­(NH_3_)_2_(SCN)_4_]) was used as a chemical actinometer to quantify the photon flux similarly to the procedure reported by Milsmann.^2b^ Using this method, the reaction quantum yield was determined as 0.1% (see SI pp 13 for details), ruling out a radical chain mechanism. Furthermore, to distinguish between an excited-state electron transfer mechanism and one involving VLIH, Stern–Volmer quenching experiments were performed. In contrast to what would be expected for dynamic quenching by the alkyne, the (weak) emission of **1-C**_**4**_ **Me**_**4**_ at 380 nm remained unaffected upon the addition of increasing amounts of alkyne (see SI Figure S7 for details). This observation does not support a photoinduced electron transfer process involving the alkyne.

**Figure 4:**
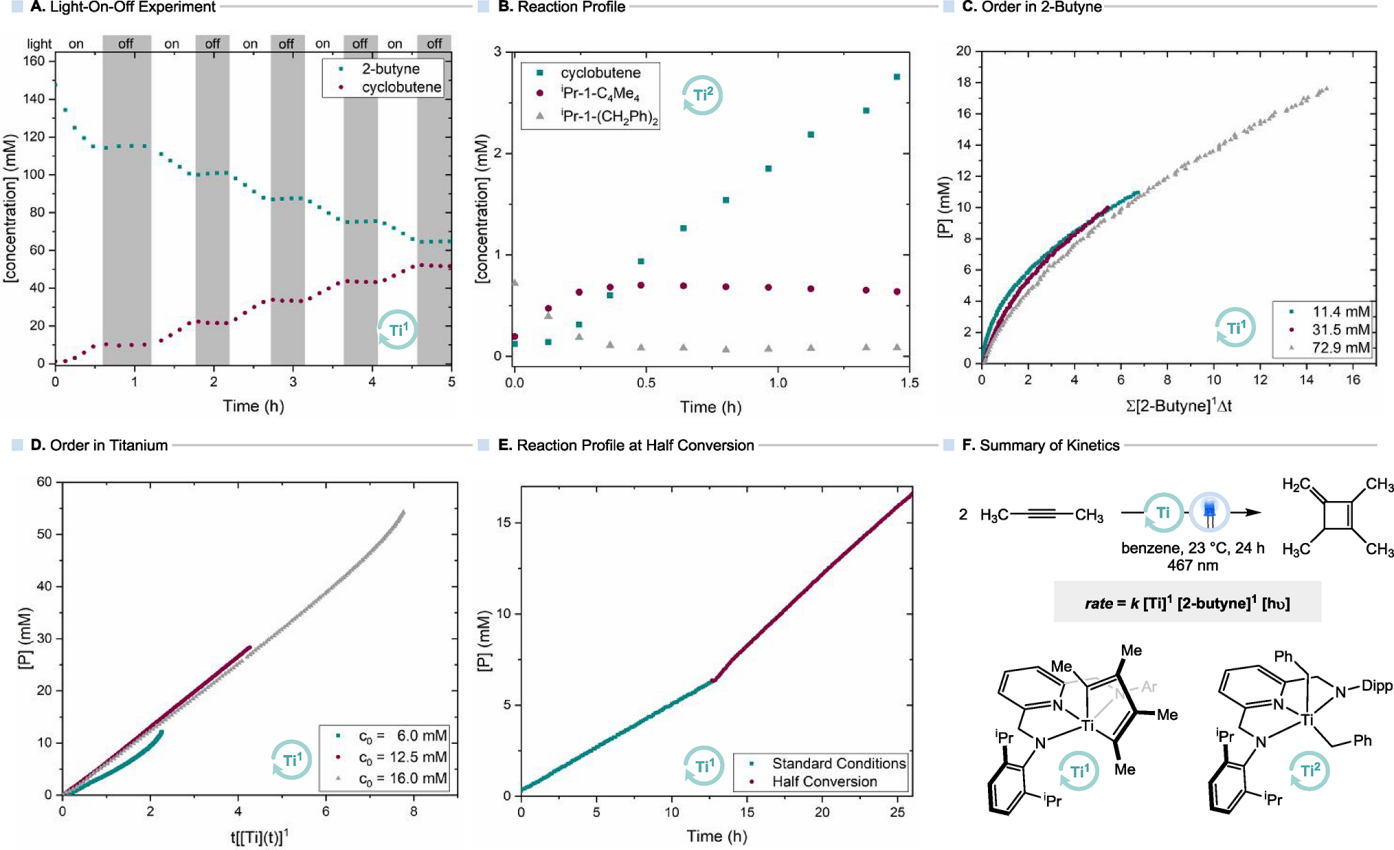
(A) Light-on–off experiment (400 nm LED); (B) reaction profile starting with **1-(CH**_**2**_ **Ph)**_**2**_ as the precatalyst; (C) VTNA plot for determination of the order in 2-butyne; (D) VTNA plot for determination of the order in titanium; (E) kinetics at half conversion; and (F) summary of kinetics. All data points were obtained by ^1^H NMR spectroscopy using (Me_3_Si)_2_O as an internal standard and 460–470 nm LED unless otherwise noted.

Finally, kinetic studies on the catalytic cyclodimerization were carried out by using *in situ* LED ^1^H NMR spectroscopy. Initially, a reaction profile was recorded for the reaction of 2-butyne with **1-(CH**_**2**_ **Ph)**_**2**_ (5 mol %) in benzene-*d*_6_ (Figure 4B). The profile revealed a clear formation of methylenecyclobutene over time and a decline of **1-(CH**_**2**_ **Ph)**_**2**_. **1-C**_**4**_ **Me**_**4**_ was identified as the major (>90%) organometallic species in the reaction mixture after the first hour of illumination, indicating that it is the resting state during catalysis. Notably, a gradual decline in the concentration of **1-C**_**4**_ **Me**_**4**_ was observed over the course of 10 h, accompanied by the accumulation of **1-CH**_**2**_ **C**_**7**_ **H**_**10**_, as identified by the continuously increasing intensity of the doublets at 5.37 and 4.85 ppm in the ^1^H NMR spectra, providing evidence for its role as the catalyst deactivation product. Further kinetic studies varying the concentration of 2-butyne yielded the best fit in VTNA for a first-order dependence in the substrate (Figure 4C; see SI Figures S16–S18 for additional VTNA plots).^41^ Although preliminary analysis of the data at different catalyst loadings suggested a second-order dependence in titanium,^42^ correcting for the actual concentration of active catalyst revealed a clear first-order dependence. (Figure 4D; see SI Figures S10–S15 for additional VTNA plots). This behavior, together with the accumulation of **1-CH**_**2**_ **C**_**7**_ **H**_**10**_, indicates catalyst deactivation during the reaction, as indicated by our initial reaction profiles. The observed slower rate at 50% conversion, compared to a reaction starting directly at 50% conversion (0.17 mmol of the cyclobutene was added at the start of the reaction), confirms catalyst deactivation over time (Figure 4E). In conclusion, the kinetic data are most consistent with a rate-determining step that is first order in both the substrate and catalyst. Additionally, the reaction rate is influenced by photon flux, as lower product yields were observed when light intensity was reduced (see entry 11 in Table 1), suggesting that a photochemical step is also involved in the rate-determining step (Figure 4F).

Lastly, to probe the potential involvement of paramagnetic and NMR-silent Ti­(III) species, *in situ* EPR and UV–vis spectroscopy were also performed during 467 nm irradiation. However, no Ti­(III) signal was detected, indicating that such intermediates are too short-lived to be observed using steady-state techniques (see SI Figures S8 and S21).

### Mechanistic Proposal

Based on experimental and computational findings, the mechanism outlined in Scheme 2 is proposed for the photocatalytic cyclodimerization of 2-butyne. The precatalyst **1-(CH**_**2**_ **Ph)**_**2**_ is activated in the presence of excess alkyne and light, selectively forming **1-C**_**4**_ **Me**_**4**_, which also serves as the resting state. TD-DFT calculations indicate a ligand-to-metal charge transfer, which is proposed to induce Ti–C bond homolysis, generating a biradicaloid (**int-1**) resembling previously isolated titanacyclobutadienes.^22^ Upon homolysis, the open coordination site on Ti­(III) is occupied by an additional molecule of alkyne, consistent with the observed first-order dependence on 2-butyne. Kinetic data support this visible-light-induced homolysis (VLIH) as the rate-determining step. Presumably, alkyne coordination at this step occupies the metalloradical’s MO, preventing recombination to form **1-(CH**_**2**_ **C**_**7**_ **H**_**10**_ **)**. Subsequently, a hydrogen atom shift stabilizes the radical on an sp^3^-hybridized carbon adjacent to a vinylic substructure (allylic radical, **int-2**). This intermediate undergoes a 4-exo-trig radical cyclization, affording a cyclobutene coordinated to a Ti­(IV) center (**int-3**). In the presence of excess alkyne, the methylenecyclobutene is released, and the titanacyclopentadiene **1-C**_**4**_ **Me**_**4**_ is regenerated through oxidative cyclization, completing the catalytic cycle.^43^

**Scheme 2:**
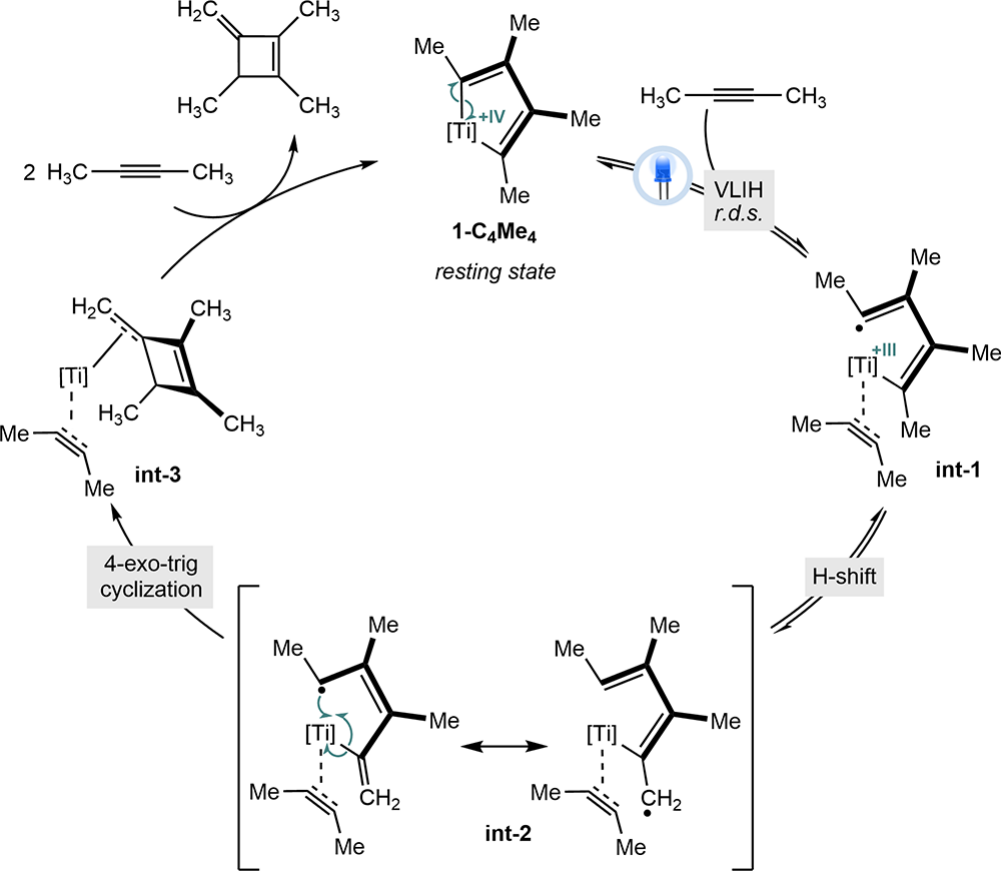
Proposed Mechanism for the PDA-Titanium-Catalyzed Cyclodimerization of 2-Butyne

### Reaction Scope

In addition to mechanistic investigations, an extension of the reaction to other substrates was explored. When 3-hexyne was subjected to the optimized reaction conditions shown in Table 1, the reaction proceeded with a lower conversion (75%) and yielded an isomeric mixture of dimers, including the previously observed one (Figure 5).^31^ The UV–vis absorption spectrum of isolated metallacycle **1-(C**_**4**_ **Et**_**4**_ **)** in benzene exhibits features similar to those of **1-(C**_**4**_ **Me**_**4**_ **)**, indicating that the same wavelength induces LMCT in both complexes (see Figure S9 of the SI). Nonsymmetric alkynes were also examined. The reaction of 2-pentyne and 2-hexyne resulted in conversions of 58 and 32%, respectively, whereas 1-phenyl-1-propyne did not afford any new organic products. Instead, formation of the titanacyclopentadiene was observed. Similarly, cyclooctyne, diphenylacetylene, and the bisalkyne nona-2,7-diyne only afforded the metallacycles, with no further catalytic formation of cyclodimer observed (Figure 5, for additional details, see Table S11 of the SI). The lack of catalytic activity can be rationalized in part by the substitution pattern of the alkynes used.^44^ Diphenylacetylene has no C–H bonds in the α-position to the alkyne, and the bis­(alkyne) forms a conformationally locked metallacycle (Figure 3), which is unreactive. In contrast, the reduced reactivity of other bisalkyl-substituted alkynes such as 3-hexyne may be attributed to the increased stability and decreased reactivity of radical intermediates such as **int-2**. Lastly, the cross-dimerization of two distinct alkynes was also attempted by subjecting a 1:1 mixture of 2-butyne and 3-hexyne to the catalytic conditions. Analysis by ^1^H NMR spectroscopy and GC mass spectrometry showed both dimerization and cross dimerization (SI Figures S88–S92).

**Figure 5:**
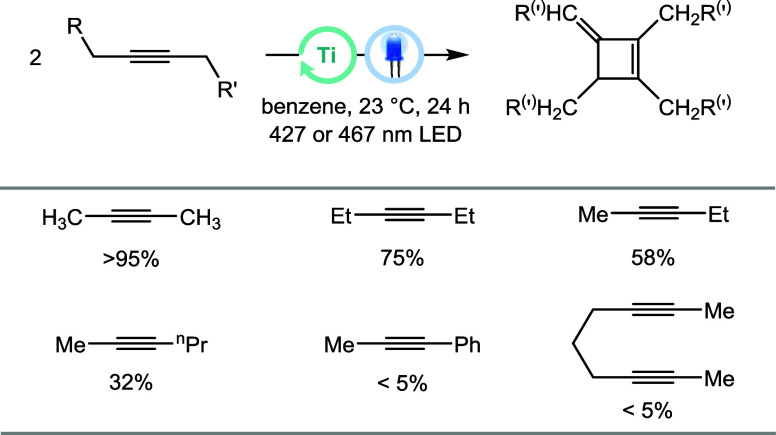
Conversions of alkynes in catalytic cyclodimerization; standard conditions: 0.37 mmol of alkyne, [Ti] = 0.05 mol/L, 50% Kessil lamp intensity; conversion was determined by ^1^H NMR spectroscopy using HMDSO as an internal standard.

## Conclusions

In summary, LMCT-induced Ti–C bond homolysis in a pincer-supported titanacyclopentadiene enabled a rare and selective rearrangement of the carbon skeleton within the metallacycle via a hydrogen atom shift. The visible-light-induced bond homolysis was rationalized by TD–DFT calculations, and the critical role of C–C bond rotation was confirmed by the lack of reactivity in a conformationally locked metallacycle. This stoichiometric reactivity was leveraged for the development of a catalytic alkyne cyclodimerization under mild conditions, affording the highly strained and readily functionalizable 1,2,3-trimethyl-4-methylenecyclobutene in quantitative yields. Suitable postmodification may include metathesis,^45^ Wacker oxidation,^46^ epoxidation,^47^ or hydrofunctionalizations.^48^ Detailed studies on catalyst identity and reaction conditions revealed the importance of sterics in this transformation. The sterically demanding pyridine-diamido ligand is crucial here, as it suppresses alkyne cyclotrimerization, which is typically observed with metallacyclopentadienes.^49^ Single-turnover experiments further demonstrated that while steric hindrance is essential, sufficient space must remain at the open coordination site to allow substrate binding and catalytic turnover. Thus, achieving a balance between steric protection to prevent trimerization and maintaining accessibility for alkyne coordination is key for catalytic turnover. Mechanistic investigations, including light-on-light-off experiments and quantum yield determination, support a mechanism involving visible-light-induced homolysis and the formation of a Ti–C biradicaloid. Kinetic analyses indicate that the VLIH step is rate-determining. This study introduces a new non-metallocene titanium photocatalyst, offering a new perspective in alkyne oligomerization chemistry, which is typically dominated by trimerization to arenes and dimerization to enynes. The new cyclodimerization enables the formation of a methylenecyclobutene with a substantially higher molecular complexity than the simple starting material 2-butyne. This reaction is enabled not by electronically tuned ligands but by a catalyst structure that exploits steric control to prevent trimerization and promote Ti–C bond homolysis. Ongoing work in our laboratory focuses on further mechanistic studies and extension of the substrate scope.

## Supplementary Material



## Notes

Experimental details, reaction optimization, NMR spectra, single-crystal X-ray diffraction data, and computational details (PDF)
