# Supplementary material for: Visible-Light-Induced Bond Homolysis in Titanacyclopentadienes for the Catalytic Cyclodimerization of Internal Alkynes
Source: J Am Chem Soc. 2025 Sep 30;147(41):37741–50. doi: 10.1021/jacs.5c13492 (PMC12532294; doi:10.1021/jacs.5c13492)
Supplement: Supplementary file 1 [file ja5c13492_si_001.pdf]

Supplementary Information for

Visible-Light-Induced Bond Homolysis in Titanacyclopentadienes for the Catalytic Cyclodimerization of Internal Alkynes

Maxi L. Heldner, Tobias Körner, Corinna Czernetzki, Patrick T. Geppert, Agnieszka Nowak-Król, Gabriele Hierlmeier*

Institute for Inorganic Chemistry, Julius-Maximilians-Universität Würzburg, Am Hubland, 97074 Würzburg, Germany
E-mail: gabriele.hierlmeier@uni-wuerzburg.de

Table of Contents

1. Methods and Materials 2
2. Synthetic Procedures of Novel Compounds 3
3. Optical Spectra 6
4. Reaction Quantum Yield Determination 13
5. Kinetic Measurements 15
6. EPR Measurements 33
7. DFT Calculations 34
8. X-ray Crystallographic Details 42
9. Alternative Mechanisms 44
10. NMR and GC-MS Spectra of Isolated Compounds 46
12. Substrate Scope 73
12. References 99

1. Methods and Materials

All manipulations were performed either under an atmosphere of dry argon or in vacuo using standard Schlenk line or glovebox techniques. Deuterated solvents were dried over molecular sieves and degassed by three freeze-pump-thaw cycles prior to use. All other solvents were distilled and degassed from appropriate drying agents (CaH_2 or 4 Å molecular sieves). Both deuterated and non-deuterated solvents were stored under argon over activated 4 Å molecular sieves. NMR spectra were acquired either on a Bruker Avance 600 (operating at 600 MHz for ^1H and 150 MHz for ^{13}C) Bruker Avance 500 (operating at 500 MHz for ^1H and 125 MHz for ^{13}C) or a Bruker Avance 400 NMR (operating at 400 MHz for 1H and 100 MHz for ^{13}C). Chemical shifts (δ) are given in ppm and internally referenced to the carbon nuclei ($^{13}C\{^1H\}$) or residual protons (^1H) of the solvent. Microanalyses (C, H, N) were performed on an Elementar vario MICRO cube elemental analyzer at the University of Regensburg and the Julius Maximilians University Würzburg. EPR measurements at X-band (9.4 GHz) were carried out at room temperature using a Bruker ELEXSYS E580 EPR spectrometer. The UV-Vis absorption spectra were measured on a JASCO V-660 UV-Vis spectrometer using standard quartz glass cuvettes. The emission spectra were measured on an Edinburgh Instruments FLSP920 spectrometer equipped with a double monochromator for both excitation and emission, operating in right-angle geometry mode. The GC-MS data was acquired on an Agilent 7890A gaschromatograph. Photoreactions were carried out in J Young NMR tubes or conventional Schlenk flasks using Kessil lamps as the light source. The samples were placed 5 cm in front of the lamp. During the irradiation the reactions were cooled using two fans. If not otherwise mentioned, the Kessil lamps were run at 0.5 intensity. The optical power of the LED under these conditions is 150 mW/cm^2.

$Ti(CH_2Ph)_4$,[1] $Cp_2Ti(\eta^2\text{-}Me_3SiCCSiMe_3)$,[2] $^{R}PDA\text{-}H_2$ (R = Me, ^{i}Pr),[3] $^{R}PDATi(CH_2Ph)_2$ (R = ^{i}Pr, Me),[4] $^{iPr}PDATiCl_2$,[5] cyclooctyne,[6] 2,7-nonadiyne[7] were prepared following literature procedures.

2. Synthetic Procedures of Novel Compounds

(^{iPr}PDA)Ti($CH_2C_7H_{10}$)

In a glovebox, a Schlenk flask was charged with (^{iPr}PDA)Ti(CH_2Ph)$_2$ (30.0 mg, 49.0 µmol, 1.0 equiv.) and benzene (0.5 mL, 0.1 M) and transferred outside the glovebox. The flask was placed in front of a Kessil lamp and irradiated for 16 h at 467 nm. Subsequently, the solvent was removed under reduced pressure and the residue was washed with cold *n*-hexane (−30 °C, 3 x 1 mL). The product was obtained quantitatively as an orange solid.

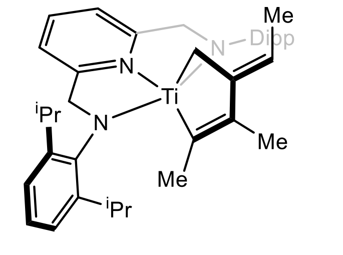

$C_{39}H_{53}N_3Ti$, MW = 611.74 g/mol

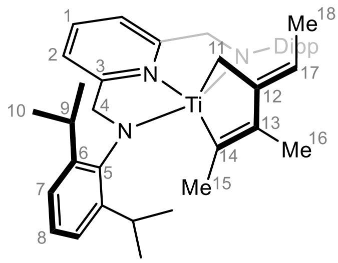

NMR assignment

Yield: 30 mg (quantitative).

^{1}H NMR (300 MHz, benzene-*d$_6$*): δ [ppm] = 7.33 (dd, ^3J = 7.4, 1.9 Hz, 2H, C7), 7.24 (t, ^3J = 7.6 Hz, 2H, C8), 7.22 (dd, ^3J = 7.4, 1.9 Hz, 2H, C7), 6.81 (t, ^3J = 7.7 Hz, 1H, C1), 6.37 (d, ^3J = 7.8 Hz, 2H, C2), 5.37 (d, ^2J = 21.4 Hz, 2H, C4), 4.92 (q, ^3J = 6.8 Hz, 1H, C17), 4.85 (d, ^2J = 21.3 Hz, 2H, C4), 4.13 (sept, ^3J = 6.8 Hz, 2H, C9), 3.44 (sept, ^3J = 6.9 Hz, 2H, C9), 2.14 (s, 2H, C11), 1.76 (s, 3H, C16), 1.66 (s, 3H, C15), 1.44 (d, ^3J = 6.9 Hz, 6H, C10), 1.42 (d, ^3J = 6.7 Hz, 6H, C10), 1.33 (d, ^3J = 6.9 Hz, 6H, C10), 1.19 (d, ^3J = 6.8 Hz, 6H, C10), 0.89 (t, ^3J = 6.9 Hz, 3H, C18).

^{13}C{^{1}H} NMR (126 MHz, benzene-*d$_6$*): δ [ppm] = 209.1 (s, C_q, C14), 162.4 (s, C_q, C3), 152.4 (s, C_q, C5), 149.4 (s, C_q, C13), 146.0 (s, C_q, C6), 144.3 (s, C_q, C6), 140.1 (s, C_q, C12), 137.9 (s, CH, C1), 126.5 (s, CH, C8), 123.9 (s, CH, C7), 123.6 (s, CH, C7), 116.9 (s, CH, C2), 112.6 (s, CH, C17), 86.7 (s, CH_2, C11), 69.3 (s, CH_2, C4), 28.9 (s, CH, C9), 27.6 (s, CH, C9), 27.2 (s, CH_3, C10), 26.8 (s, CH_3, C10), 24.2 (s, CH_3, C10), 24.0 (s, CH_3, C10), 18.7 (s, CH_3, C16), 15.1 (s, CH_3, C15), 14.4 (s, CH_3, C18).

The peaks in the ^{13}C{^{1}H} NMR spectrum at δ = 209.1 ppm and δ = 140.1 ppm were identified by the resonances in the ^{1}H-^{13}C-HMBC NMR spectrum (Figure S27).

Elemental Analysis: calc. for $C_{39}H_{53}N_3Ti$ [611.74 g/mol] C 76.57, H 8.73, N 6.87; found: C 77.30, H 9.36, N 6.81.

(^{iPr}PDA)Ti ($C_4Me_2(CH_2)_3$)

In a glovebox, a Schlenk flask was charged with (^{iPr}PDA)Ti($CH_2Ph)_2$ (20.0 mg, 0.03 mmol, 1.0 equiv.) and benzene (1.5 mL, 0.02 M) was added. 2,7-Nonadiyne (3.50 mg, 0.03 mmol, 1.0 equiv.) was added to the suspension and the flask was sealed and transferred outside the glovebox. The flask was placed in front of a Kessil lamp and irradiated for 16 h at 467 nm. Subsequently, the solvent was removed under reduced pressure and the residue washed with cold *n*-hexane (−30 °C, 3 x 1 mL). The product was obtained as a yellow solid.

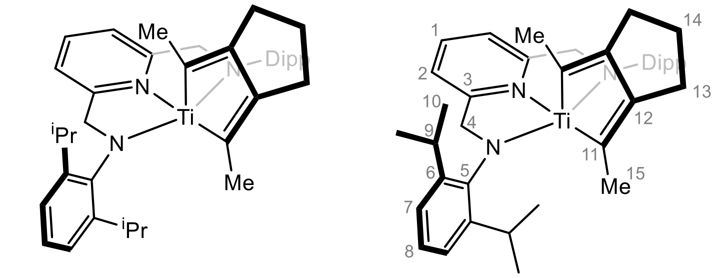

$C_{40}H_{53}N_3Ti$, MW = 623.75 g/mol   NMR assignment

Yield: 14.4 mg (80%)

^{1}H NMR (400 MHz, benzene-d_6): δ [ppm] = 7.13 – 7.05 (m, 6H, C7,C8), 6.96 (t, ^3J = 7.7 Hz, 1H, C1), 6.54 (d, ^3J = 7.6 Hz, 2H, C2), 4.86 (s, 2H, C4), 3.51 (sept, ^3J = 6.8 Hz, 4H, C9), 2.78 (t, ^3J = 7.6 Hz, 4H, C13), 1.87 (s, 6H, C15), 1.32 (m, 2H, C14), 1.27 (d, ^3J = 6.8 Hz, 12H, C10), 1.20 (d, ^3J = 6.9 Hz, 12H, C10).

^{13}C{^{1}H} NMR (101 MHz, benzene-d_6): δ [ppm] = 212.2 (s, C_q, C11), 162.6 (s, C_q, C3), 153. 7 (s, C_q, C5), 143.2 (s, C_q, C6), 137.5 (s, CH, C1), 133.0 (s, C_q, C12), 124.4 (s, CH, C8), 123.3 (s, CH, C7), 116.8 (s, CH, C2), 67.5 (s, CH_2, C4), 37.8 (s, CH_2, C14), 28.3 (s, CH, C9), 26.4 (s, CH_3, C10), 24.3 (s, CH_3, C10), 21.6 (s, CH_3, C15), 18.6 (s, CH_2, C13).

Elemental Analysis: calc. for $C_{40}H_{53}N_3Ti$ [623.75 g/mol] C 77.02, H 8.56, N 6.74; found: C 77.46, H 9.01, N 6.30.

1,2,3-Trimethyl-4-methylenecyclobutene

Isolation as neat substance:

A J Young NMR tube was charged with (^{iPr}PDA)Ti($CH_2Ph)_2$ (12.7 mg, 18.0 µmol, 5 mol%), 2-butyne (20 mg, 0.37 mmol, 1.0 equiv.) and toluene (0.4 mL, 45 mM). The tube was placed in front of a 467 nm Kessil lamp and irradiated for 24 h at room temperature. The (neat) product was distilled into a pre-weighed J Young NMR tube under argon at 100 °C and ambient

pressure. The product mass was determined, and the product was afforded as colorless liquid in 80% yield (15.9 mg, 0.29 mmol).

<u>Isolation as stock solution in benzene-d_6:</u>

A J Young NMR tube was charged with $(^{iPr}PDA)Ti(CH_2Ph)_2$ (12.7 mg, 18.0 µmol, 5 mol%), 2-butyne (20 mg, 0.37 mmol, 1.0 equiv.) and benzene-d_6 (0.4 mL, 45 mM). HMDSO (10 µL, 47 µmol) was added to quantify the yield by ^{1}H NMR spectroscopy. The tube was placed in front of a 467 nm Kessil lamp and irradiated for 24 h at room temperature. Full conversion and selective formation of 1,2,3-trimethyl-4-methylenecyclobutene was confirmed by ^{1}H NMR spectroscopy. The product was condensed into another J Young tube under reduced pressure (10^{-2} mbar) using a vacuum line, affording a solution of 1,2,3-trimethyl-4-methylenecyclobutene in benzene-d_6 (c = 0.46 M) for further reactivity study and analysis in quantitative yield.

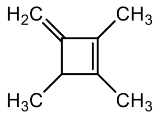

C_8H_{12}, MW = 108.18 g/mol

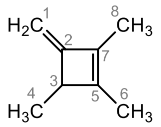

NMR assignment

^{1}H NMR (600 MHz, benzene-d_6): δ [ppm] = 4.59 (s, 1H, C1), 4.49 (s, 1H, C1), 2.98 (m, 1H, C3), 1.52 (m, 3H, C6), 1.50 (m, 3H, C8), 1.12 (d, *J* = 6.8 Hz, 3H, C4).

^{13}C NMR (101 MHz, benzene-d_6): δ [ppm] = 155.0 (s, C_q, C5), 150.8 (s, C_q, C7), 138.1 (s, C_q, C2), 90.9 (s, CH_2, C1), 45.2 (s, CH, C3), 15.8 (s, CH_3, C4), 11.2 (s, CH_3, C8), 9.0 (s, CH_3, C6).

GC-MS: [M] = 108.18 g/mol. m/z = 108 $[M]^+$

The spectroscopic data is in agreement with previous reports.[8]

3. Optical Spectra

UV-Vis Absorption Spectra

(^{iPr}PDA)Ti(CH_2Ph)$_2$

In a glovebox, (^{iPr}PDA)Ti(CH_2Ph)$_2$ (1.6 mg, 2.3 μmol) was dissolved in *n*-hexane (10 mL). 1 mL of this stock solution was diluted with 9 mL *n*-hexane (23 μM). The solution was transferred to a Quartz cuvette and the UV-Vis absorption spectrum was obtained.

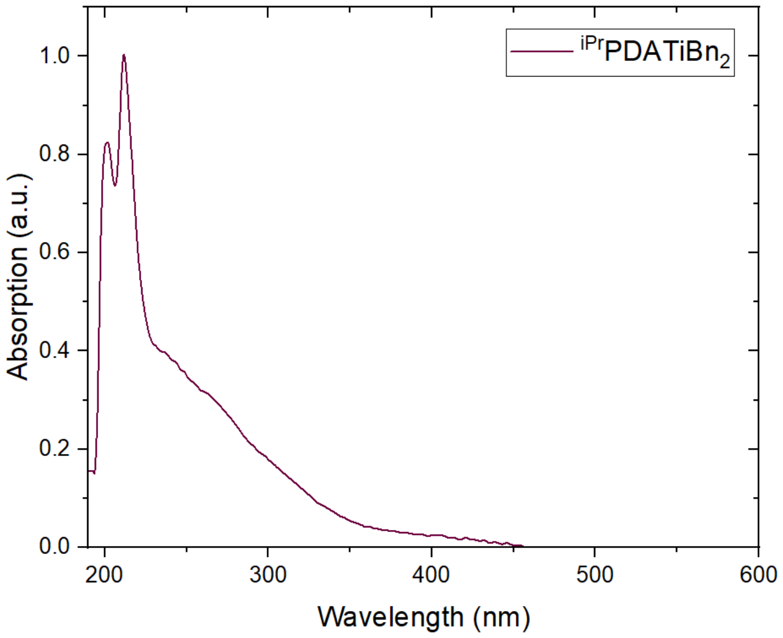

Figure S1. UV-Vis absorption spectrum of (^{iPr}PDA)Ti(CH_2Ph)$_2$ in *n*-hexane.

(^{iPr}PDA)Ti(C_4Me_4)

In a glovebox, (^{iPr}PDA)Ti(C_4Me_4) (1.5 mg, 2.3 µmol) was dissolved in *n*-hexane (10 mL). 1 mL of this stock solution was diluted with 9 mL *n*-hexane (23 µM). The solution was transferred to a Quartz cuvette. After the UV-Vis absorption spectrum was obtained, 1 equivalent 2-butyne and excess 2-butyne were added subsequently. The UV-Vis absorption spectrum of neat 2-butyne was performed with a 0.12 M solution in *n*-hexane.

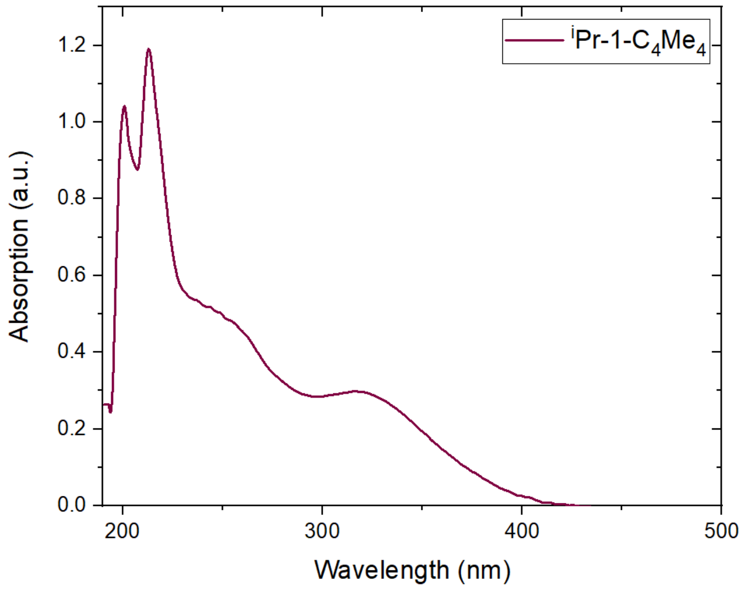

Figure S2. UV-Vis absorption spectrum of (^{iPr}PDA)Ti(C_4Me_4) in *n*-hexane.

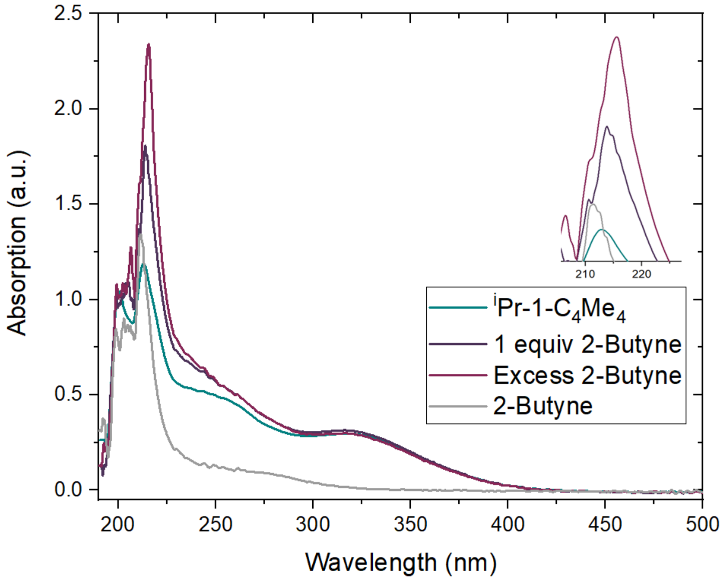

Figure S3. UV-Vis absorption spectrum of (^{iPr}PDA)Ti(C_4Me_4) in the presence of 2-butyne in *n*-hexane.

Determination of the extinction coefficient

The extinction coefficient of (^{iPr}PDA)Ti(C_4Me_4) was determined using a benzene solution at the absorption maximum λ_{max} = 318 nm. In an argon glovebox, a 1.63 mM stock solution of (^{iPr}PDA)Ti(C_4Me_4) (5.0 mg, 8.2 µmol) in benzene with a total volume of 5.0 mL was obtained with a 5.0 mL measuring flask. With this stock solution three dilutions (204 µM, 102 µM, 51 µM) were prepared. From each dilution 3 mL were transferred into a Quartz cuvette and a UV-Vis absorption spectrum was obtained.

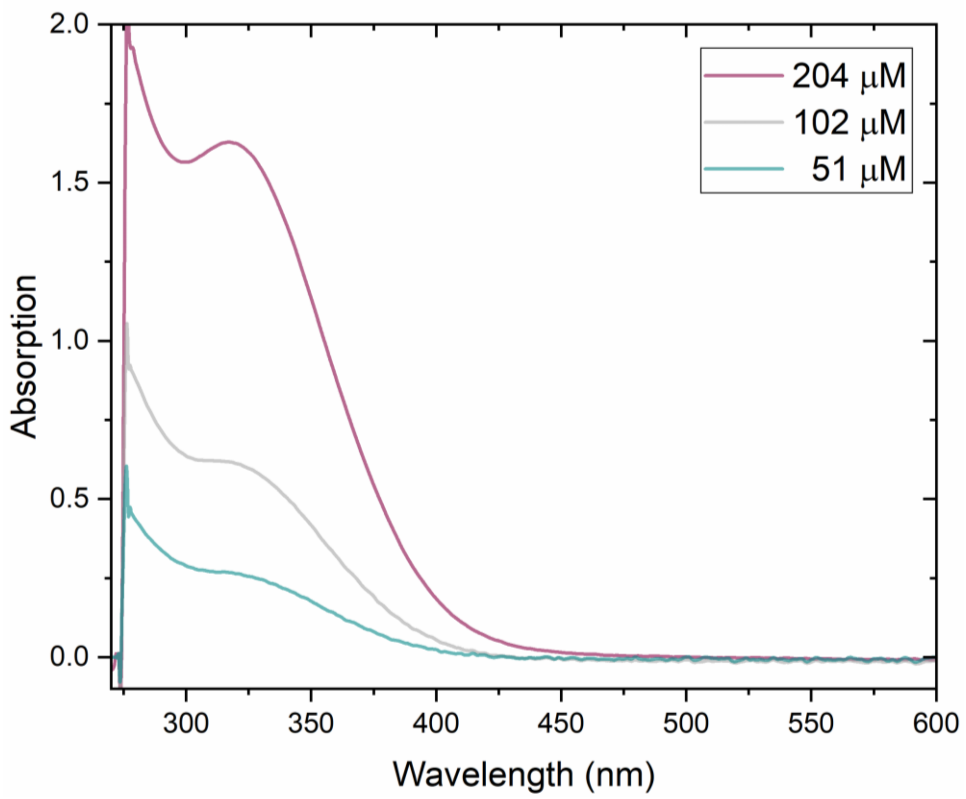

Figure S4. UV-Vis absorption spectrum for the three concentrations of (^{iPr}PDA)Ti(C_4Me_4) in benzene.

The absorption for λ_{max} at 318 nm was plotted against concentration and the extinction coefficient was obtained from the slope of the linear fit. The molar extinction coefficient for (^{iPr}PDA)Ti(C_4Me_4) is $\varepsilon = 9.0 \cdot 10^3$ L mol^{-1} cm^{-1} in benzene at λ_{max} = 318 nm.

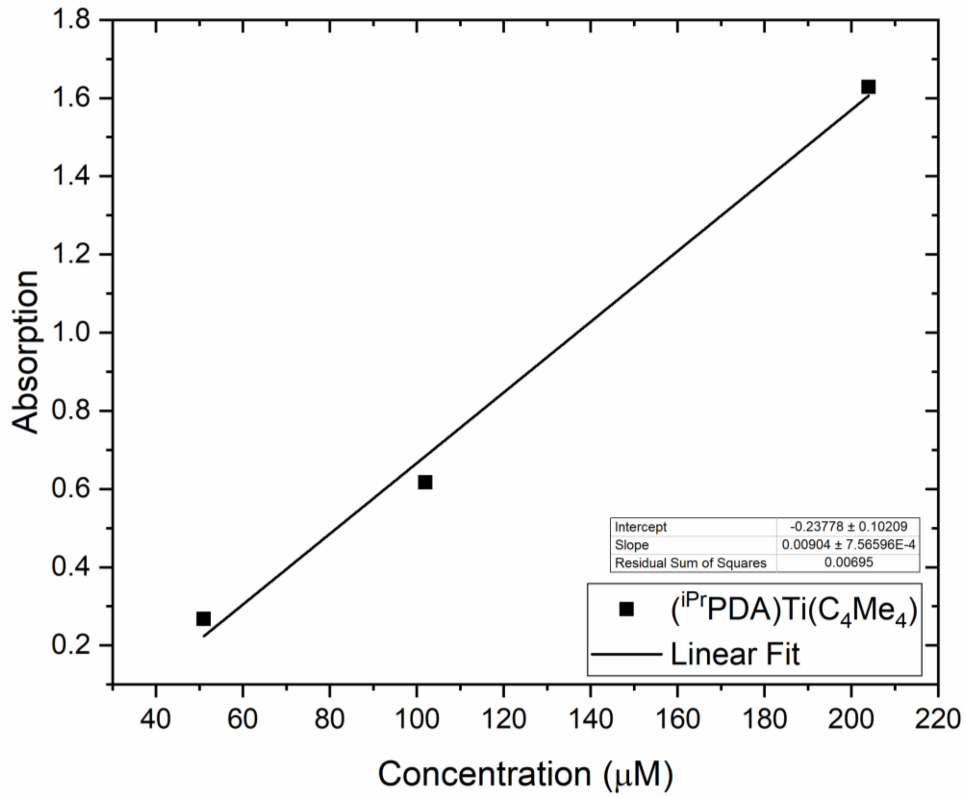

Figure S5. Linear Fit obtained for the three concentrations of (^{iPr}PDA)Ti(C_4Me_4) in benzene.

Emission Spectrum of (^{iPr}PDA)Ti(C_4Me_4)

In a glovebox, (^{iPr}PDA)Ti(C_4Me_4) (2.0 mg, 3.3 µmol) was dissolved in *n*-hexane (10 mL). 1 mL of this stock solution was diluted with 9 mL *n*-hexane (33 µM). The solution was transferred into a Quartz cuvette. After the emission spectrum was obtained (Figure S6), 10 µL, 30 µL (total amount 40 µL), and 60 µL 2-butyne (total amount 100 µL) were added subsequently. The sample showed a very weak fluorescence at 380 nm. Through the addition of 2-butyne the intensity of this peak is slightly increased, suggesting a possible coordinating interaction between the alkyne and the metal complex (Figure S7). 2-Butyne does not quench the excited state.

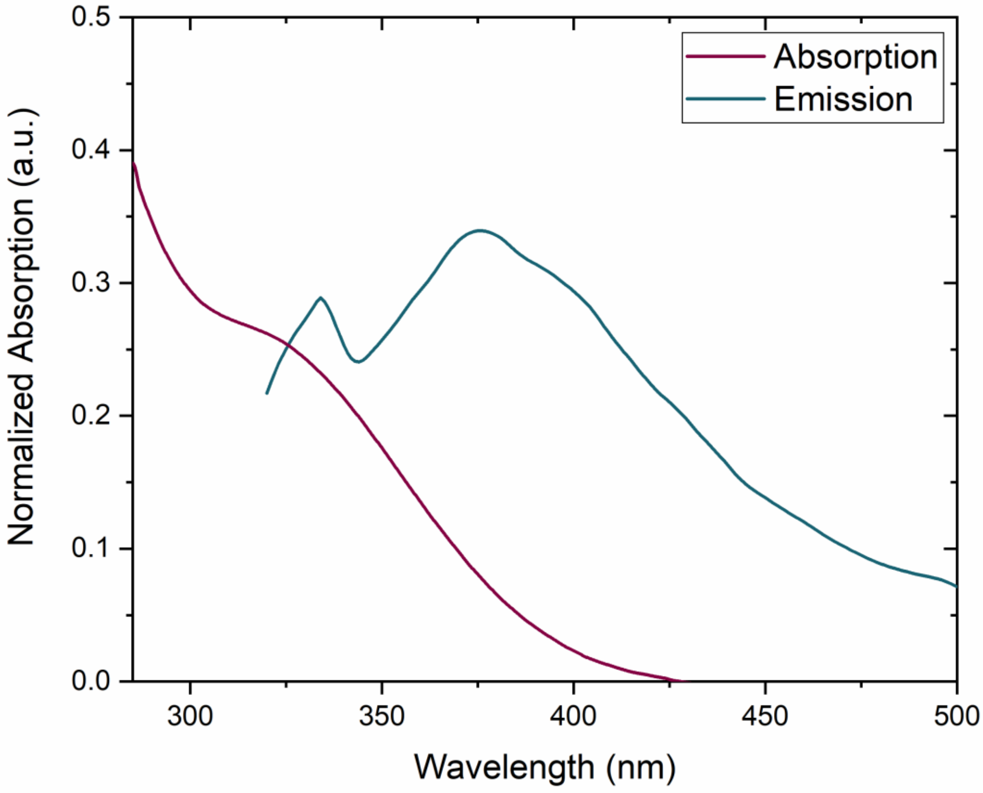

Figure S6. Emission spectrum of (^{iPr}PDA)Ti(C_4Me_4) in *n*-hexane in comparison to UV-Vis absorption spectrum. Excitation at 280 nm.

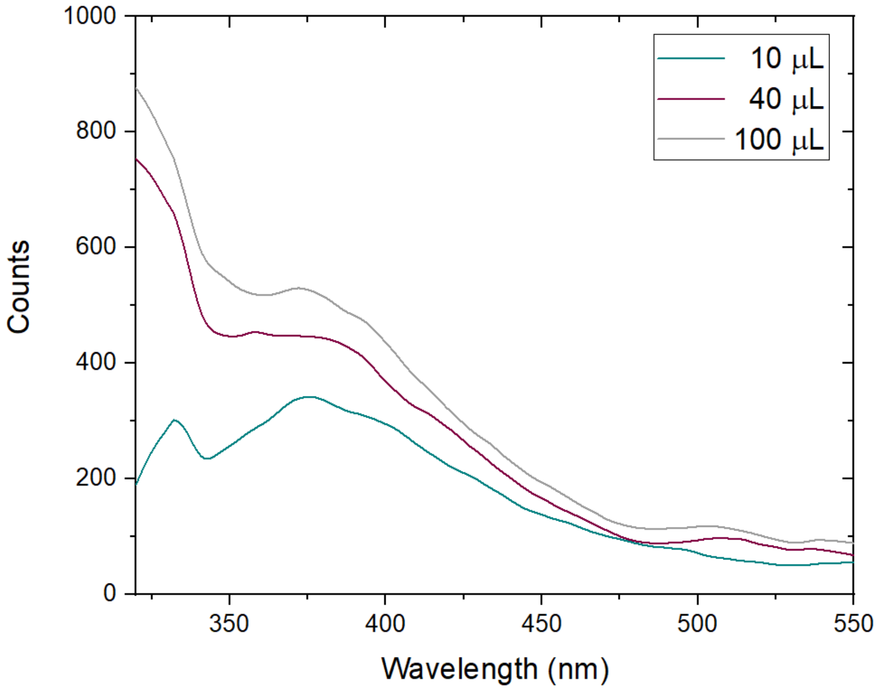

Figure S7. Emission spectrum of (^{iPr}PDA)Ti(C_4Me_4) in the presence of 2-butyne in *n*-hexane. Excitation at 280 nm.

UV-Vis measurement with steady state photolysis

In a glovebox, (^{iPr}PDA)Ti(C_4Me_4) (5.6 mg, 9.3 μmol, 1.0 equiv) was dissolved in benzene (15 mL) and 29 μL 2-butyne (0.37 mmol, 20 equiv) were added. 0.5 mL of this stock solution was diluted with 2.5 mL benzene (103 μM). The solution was transferred into a Quartz cuvette. A UV-Vis absorption spectrum was obtained before irradiation. Afterwards, the cuvette was placed in front of the 467 nm Kessil LED and irradiated for a certain period before another UV-Vis absorption spectrum was measured (after 1 min, 2 min, 5 min, 10 min, 30 min, 60 min). The stacked UV-Vis absorption spectra are plotted in Figure S8.

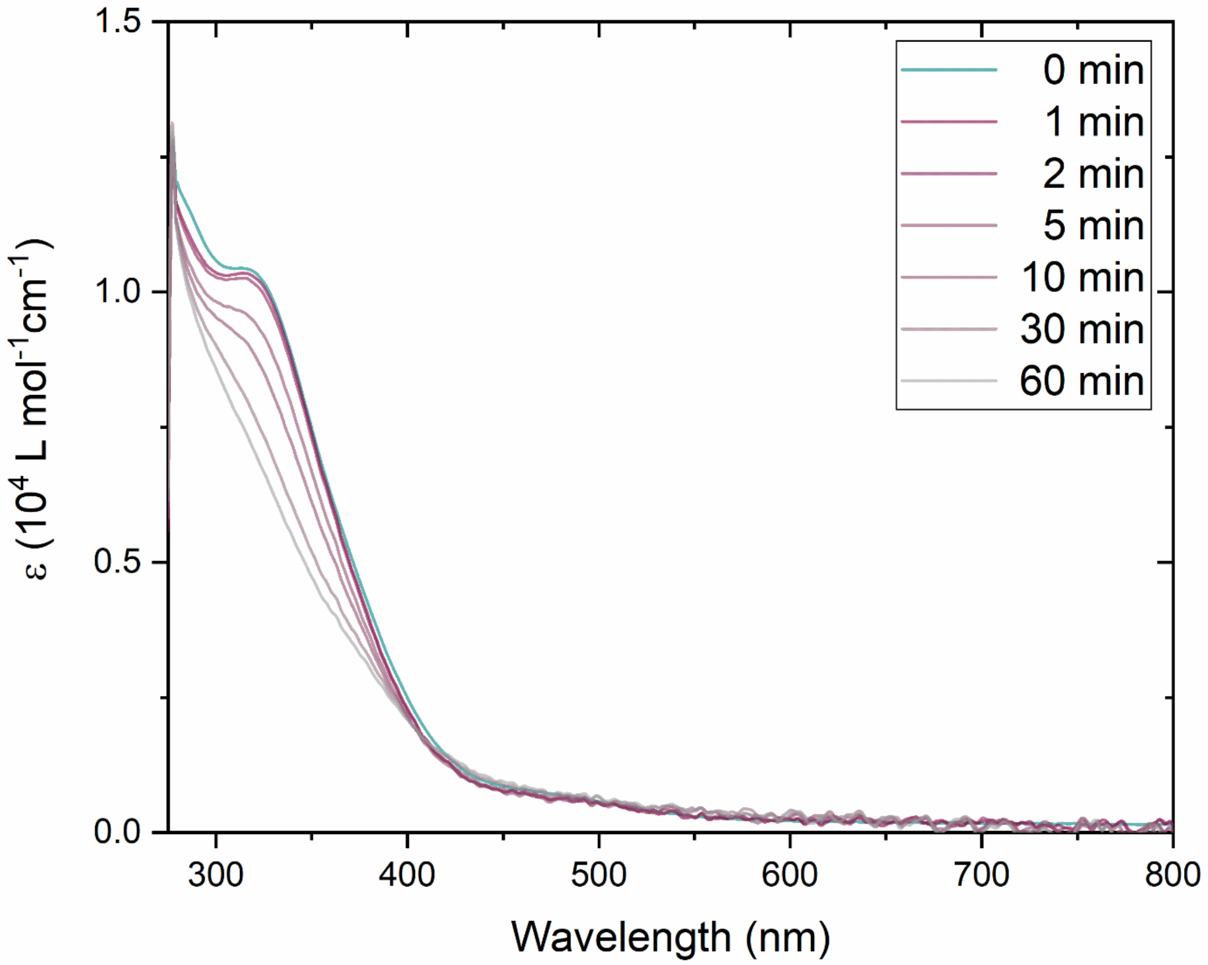

Figure S8. UV-Vis spectra changes during irradiation at 467 nm in benzene over one hour.

UV-Vis absorption spectrum of (^{iPr}PDA)Ti(C_4Et_4)

In a glovebox, (^{iPr}PDA)Ti(C_4Et_4) (2.0 mg, 3.0 μmol) was dissolved in n-hexane (10 mL). 1 mL of this stock solution was diluted with 2 mL n-hexane (33 μM). The solution was transferred into a Quartz cuvette. To compare the absorption with (^{iPr}PDA)Ti(C_4Me_4), both UV-Vis absorption spectra were plotted in the same graph (**Figure S9**).

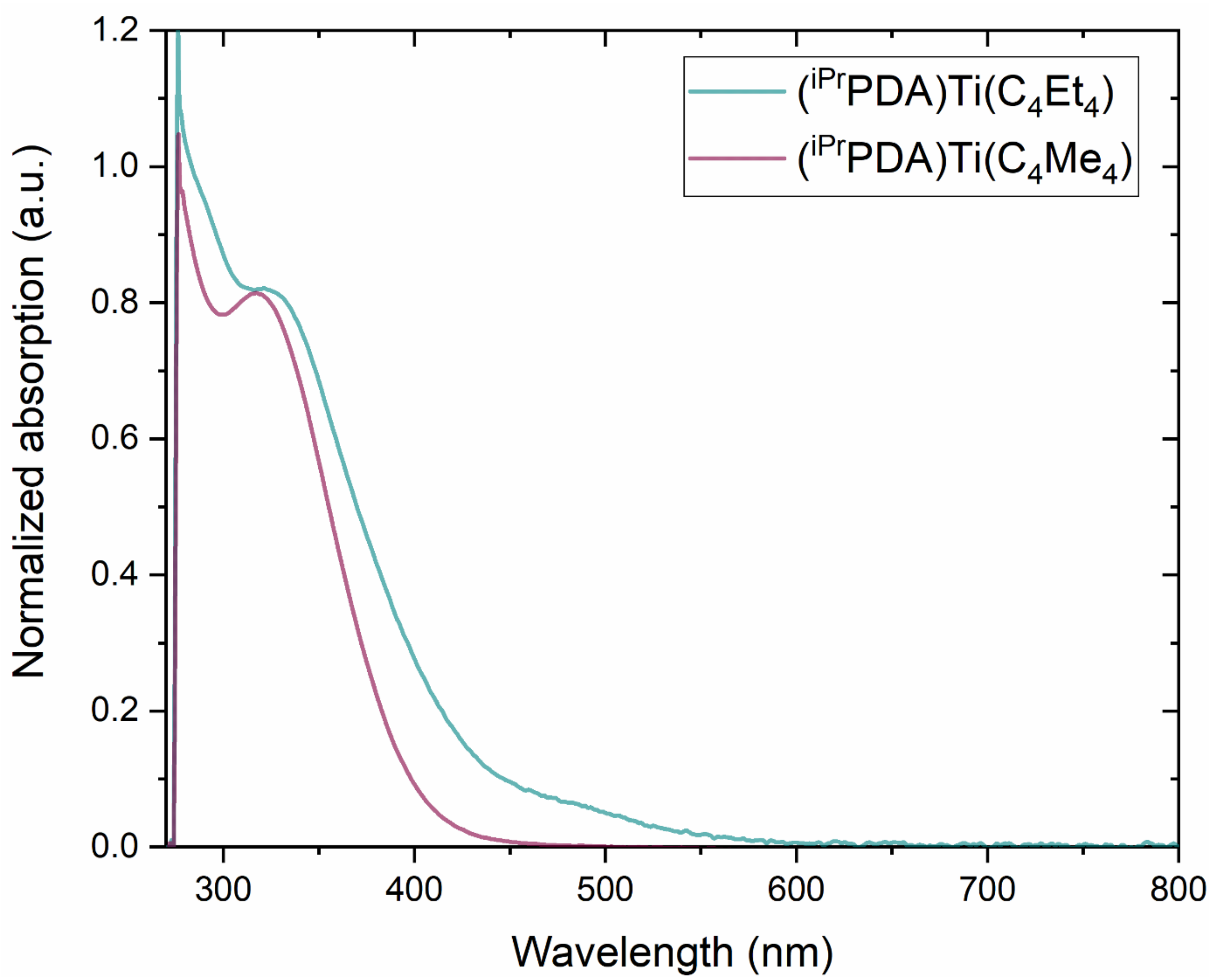

Figure S9. UV-Vis absorption spectra of (^{iPr}PDA)Ti(C_4Et_4) and (^{iPr}PDA)Ti(C_4Me_4) in benzene.

4. Reaction Quantum Yield Determination

The reaction quantum yield was determined using Reinecke's salt actinometry following a previously described procedure.[9] The potassium reineckate was obtained from $NH_4[Cr(NH_3)_2(NCS)_4]$ according to a literature procedure.[10] 50 mL of an aqueous 0.02 M solution of $K[Cr(NH_3)_2(NCS)_4]$ was prepared using a measuring flask. Additionally, a combined aqueous solution of 0.1M $Fe(NO)_3$ and 0.5 M $HClO_4$ solution was prepared. Three samples of $K[Cr(NH_3)_2(NCS)_4]$ were irradiated with a 467 nm LED in a Quartz cuvette for 40 s at room temperature. Subsequently, 0.5 mL of the photolyzed solution were added to 2.0 mL of the $Fe(NO)_3$/ $HClO_4$ solution and after 2 min the absorbance of $[Fe^{III}(SCN)]^{2+}$ at 450 nm was measured by UV-Vis spectroscopy. This was repeated with three unphotolyzed samples of potassium reineckate. The photo-aquation was calculated with the following equation:

$$\text{mol of Fe}^{\text{III}}(\text{SCN})^{2+} = \frac{V \cdot \Delta A}{l \cdot \varepsilon}$$

V is the volume of the solution (0.0025 L), ΔA is the difference in the absorbance at 450 nm between photolyzed and unphotolyzed sample solution, l is the path length (1 cm), and ε is the molar absorption coefficient at 450 nm (4300 L mol^{-1} cm^{-1}).

Table S1. Absorbance of photolyzed solutions and blanks.

Run	Absorbance of photolyzed sample at 450 nm	Absorbance of non-photolyzed sample at 450 nm	ΔA	$[Fe^{III}(SCN)]^{2+}$ (mol)
1	2.37507	-0.01190	2.38697	1.38777E-6
2	2.39327	-0.02844	2.42171	1.40797E-6
3	2.38374	-0.02874	2.41248	1.40260E-6

The fraction of light was calculated by the following equation:

$$f = 1 - 10^{-|A|}$$

A was obtained from 0.02 M aqueous solutions of potassium reineckate. The absorbance was measured at 450 nm.

Table S2. Fraction of Light.

Run	Absorbance of $K[Cr(NH_3)_2(NCS)_4]$ solution at 467 nm	Fraction of Light (f)
1	-0.02362	0.05294
2	-0.02638	0.05894
3	-0.02490	0.05572

Having the fraction of light at hand, the photon flux of the LED setup can be calculated using the following equation:

$$Photon\ Flux = \frac{mol\ of\ Fe^{III}(SCN)^{2+}}{\Phi \cdot t \cdot f}$$

Where t is the time of irradiation and f is the fraction of light. The quantum yield Φ of the Reinecke's salt is 0.311 at 450 nm and 0.299 at 505 nm. Both values gave similar values for the reaction quantum yield.[10]

Table S3. Photonflux is determined with Fraction of Light.

Run	$[Fe^{III}(SCN)]^{2+}$ (mol)	Fraction of Light (f)	Radiation time (s)	Photon Flux (einstein s^{-1})
1	1.38777E-6	0.05294	40	2.11740E-6
2	1.40797E-6	0.05894	40	1.91855E-6
3	1.40260E-6	0.05572	40	2.02022E-6
Average				2.01872E-6

Next, the reaction quantum yield was determined. In an Ar glovebox, a quartz cuvette was charged with 2-butyne (20.0 mg, 0.37 mmol, 1.0 equiv.), (^{iPr}PDA)Ti(C_4Me_4) (11.3 mg, 0.02 mmol, 5.0 mol%) and 3 mL of benzene-d_6. The cuvette was sealed and irradiated for 3 h (= 10800 s) at 467 nm. After 3 h 10 µL of HMDSO were added as internal standard and the product formation was determined via ^{1}H NMR. The reaction quantum yield was calculated with the following equation:

$$\Phi = \frac{mol\ of\ Pdt}{Photon\ flux \cdot t \cdot f}$$

Table S4. Reaction Quantum Yield.

Run	Mol of Product	Φ
1	1.39459E-6	0.00115
2	1.40678E-6	0.00128
3	1.40037E-6	0.00121
Average		**0.0012**

5. Kinetic Measurements

Kinetic measurements were performed using an *in situ* LED NMR illumination device with glass fiber as previously described by Gschwind and co-workers.[11] Stock solutions for the kinetic measurements were prepared in an argon glovebox. For the determination of the order in titanium three different catalyst concentrations of (^{iPr}PDA)Ti(C_4Me_4) in benzene-d_6 [3 mol% (6.0 mM), 5 mol% (12.5 mM), and 7 mol% (16.0 mM) of (^{iPr}PDA)Ti(C_4Me_4)] were tested. To each solution, 2-butyne (29.0 µL, 0.37 mmol, 1.0 equiv.) and hexamethyldisiloxane (10.0 µL, 0.05 mmol) were added. From these solutions 0.2 mL were transferred to the *in situ* LED NMR setup and the tube was sealed. All entries were irradiated using a blue LED overnight.

For the determination of the order in 2-butyne, (^{iPr}PDA)Ti(C_4Me_4) (11.3 mg, 18.0 µmol, 1.0 equiv.) was weighed into a vial. The complex was dissolved in 1.5 mL benzene-d_6 and 2-butyne (10.0, 20.0 or 40.0 equiv.) and hexamethyldisiloxane (10.0 µL, 0.05 mmol) were added. From these solutions 0.2 mL were transferred to the *in situ* LED NMR setup and the tube was sealed. All entries were irradiated using a blue LED overnight. Note that concentration changes from addition of µL-amounts of butyne and internal standard are neglectable here.

The resulting spectra were plotted using the VTNA method. The best overlay of the three curves represents the order in 2-butyne or titanium. It is worth noting that catalyst decomposition has a strong influence on the reaction profiles and the overlay obtained. The actual catalyst concentration was determined for each time point in Figure S10, Figure S11 and Figure S12 and results in the best overlay for first order in titanium.

In contrast, Figure S13, Figure S14 and Figure S15 were obtained using the initial catalyst concentration and erroneously give the best overlay for second order in titanium.

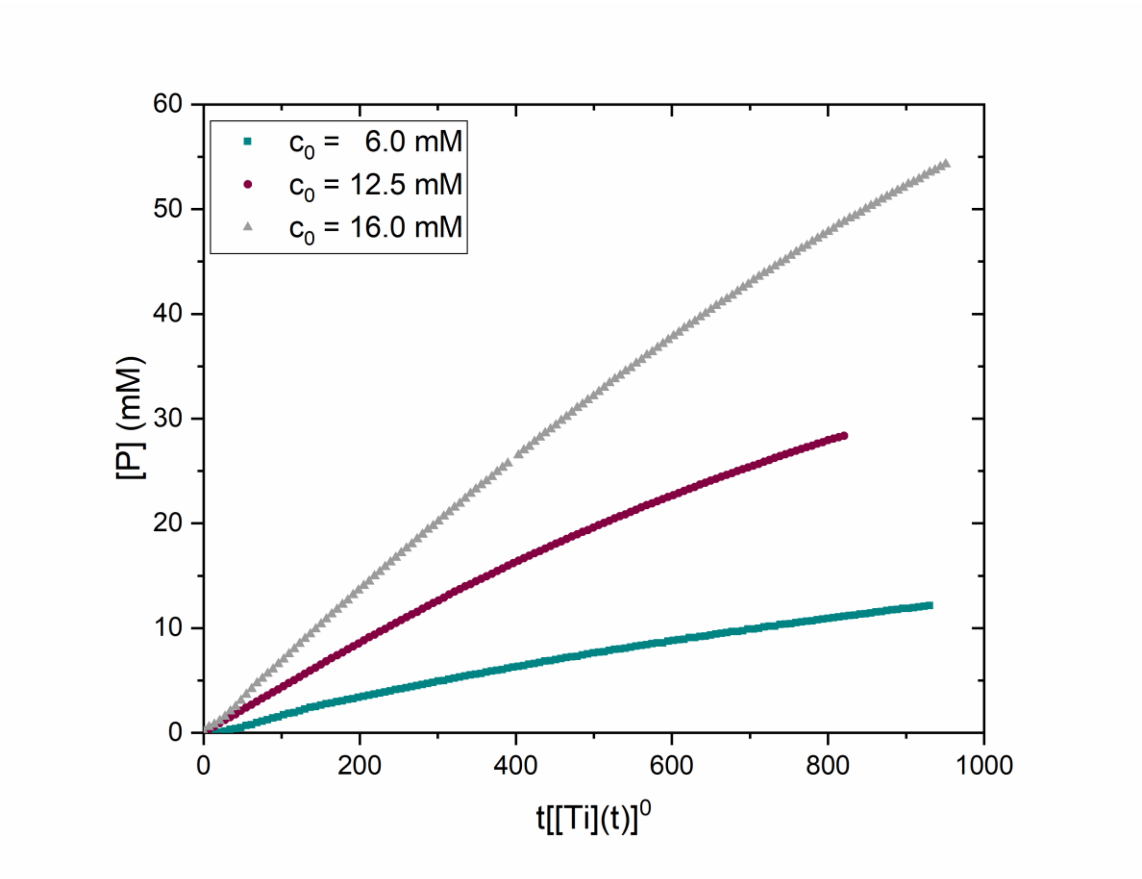

Figure S10. VTNA plot for zeroth order in titanium with time-dependent titanium concentration.

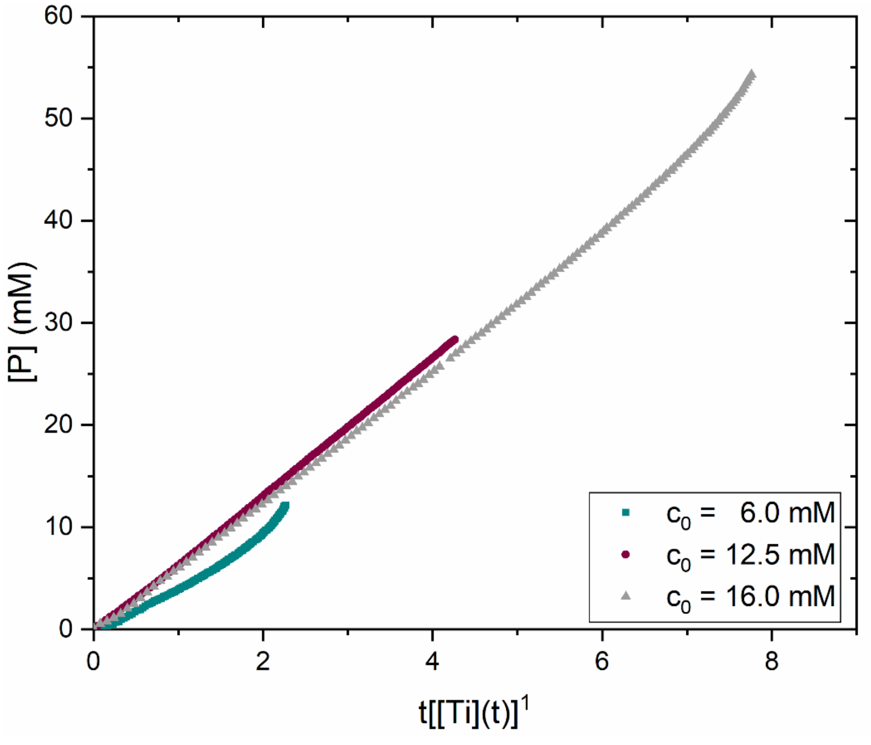

Figure S11. VTNA plot for first order in titanium with time-dependent titanium concentration.

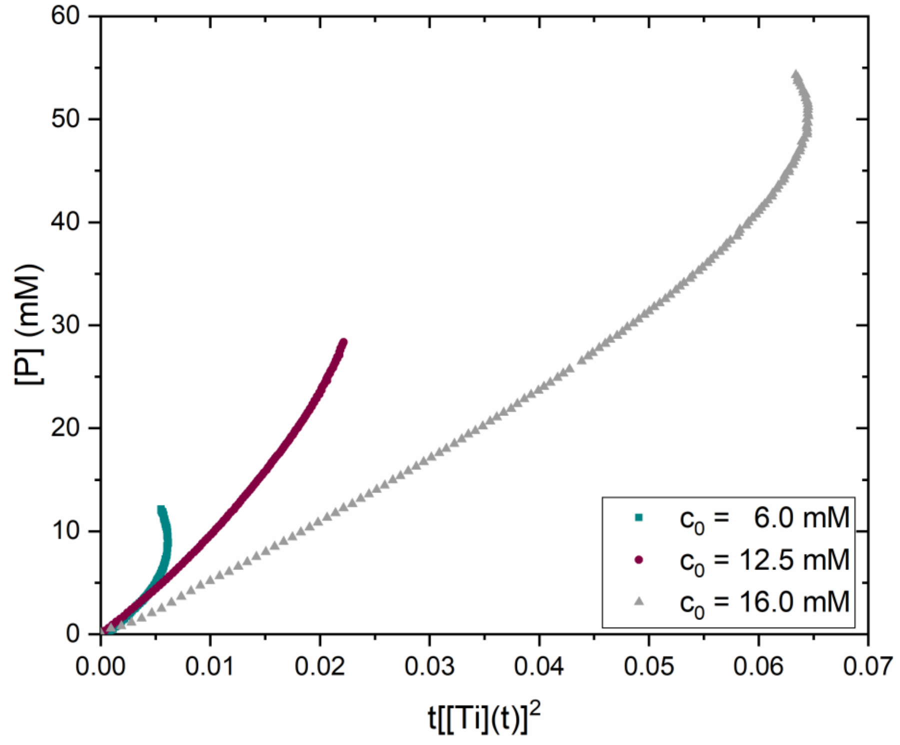

Figure S12. VTNA plot for second order in titanium with time-dependent titanium concentration.

Table S5. NMR Data Points for VTNA plots of the varied (^{iPr}PDA)Ti(C_4Me_4) concentrations. The concentrations were obtained using HMDSO as internal standard.

Time (min)	$[Ti]_{6mM}$ (M)	$[Ti]_{12.5mM}$ (M)	$[Ti]_{16mM}$ (M)
0	0.00494	0.00976	0.0117
6.84	0.00495	0.01092	0.01175
13.68	0.00493	0.01086	0.01173
20.52	0.00492	0.01084	0.01171
27.36	0.00492	0.01082	0.01169
34.2	0.00491	0.01079	0.01167
41.04	0.00489	0.01077	0.01163
47.88	0.00488	0.01075	0.01162
54.72	0.00488	0.01074	0.01159
61.56	0.00486	0.0107	0.01156
68.4	0.00485	0.01068	0.01153
75.24	0.00485	0.01063	0.01152
82.08	0.00483	0.01062	0.0115
88.92	0.00482	0.0106	0.01147
95.76	0.00481	0.01057	0.01145
102.6	0.0048	0.01053	0.01142
109.44	0.00477	0.01052	0.01139
116.28	0.00475	0.01047	0.01138
123.12	0.00472	0.01045	0.01135
129.96	0.00472	0.01043	0.01133
136.8	0.00469	0.01041	0.0113
143.64	0.00466	0.01037	0.01128
150.48	0.00463	0.01033	0.01126
157.32	0.00461	0.01033	0.01123

164.16	0.00457	0.01028	0.01122
171	0.00455	0.01026	0.01119
177.84	0.00451	0.01021	0.01116
184.68	0.0045	0.01021	0.01115
191.52	0.00447	0.01016	0.01113
198.36	0.00445	0.01013	0.01111
205.2	0.00442	0.01009	0.01108
212.04	0.00439	0.01007	0.01105
218.88	0.00438	0.01002	0.01103
225.72	0.00435	0.01002	0.011
232.56	0.00432	0.00998	0.01098
239.4	0.0043	0.00995	0.01096
246.24	0.00427	0.00992	0.01094
253.08	0.00425	0.0099	0.01092
259.92	0.00422	0.00986	0.0109
266.76	0.00419	0.00984	0.01087
273.6	0.00418	0.00978	0.01086
280.44	0.00416	0.00977	0.01084
287.28	0.00412	0.00976	0.0108
294.12	0.0041	0.00971	0.01078
300.96	0.00409	0.00969	0.01076
307.8	0.00404	0.00965	0.01074
314.64	0.00403	0.00963	0.01072
321.48	0.00401	0.00961	0.01069
328.32	0.00399	0.00957	0.01068
335.16	0.00396	0.00956	0.01065
342	0.00395	0.00952	0.01063
348.84	0.00392	0.00949	0.01061
355.68	0.00389	0.00947	0.01059
362.52	0.00388	0.00943	0.01056
369.36	0.00385	0.00941	0.01054
376.2	0.00384	0.00939	0.01052
383.04	0.00381	0.00935	0.01049
389.88	0.00379	0.00935	0.01047
396.72	0.00377	0.00931	0.00556
403.56	0.00375	0.00929	0.01042
410.4	0.00373	0.00924	0.01041
417.24	0.00371	0.00923	0.01037
424.08	0.00369	0.0092	0.01035
430.92	0.00366	0.00917	0.01033
437.76	0.00365	0.00916	0.0103
444.6	0.00363	0.00913	0.01029
451.44	0.00361	0.00911	0.01026
458.28	0.00359	0.00907	0.01023
465.12	0.00358	0.00905	0.01022
471.96	0.00354	0.00904	0.0102
478.8	0.00351	0.00901	0.01018
485.64	0.00351	0.00897	0.01015
492.48	0.0035	0.00895	0.01012
499.32	0.00348	0.00892	0.0101
506.16	0.00346	0.0089	0.01009
513	0.00344	0.00888	0.01006
519.84	0.00342	0.00884	0.01004
526.68	0.0034	0.00883	0.01001
533.52	0.00338	0.0088	0.00999
540.36	0.00335	0.00878	0.00997
547.2	0.00333	0.00875	0.00993
554.04	0.00332	0.00873	0.00992
560.88	0.0033	0.0087	0.00989
567.72	0.00328	0.00869	0.00987
574.56	0.00326	0.00865	0.00984

581.4	0.00325	0.00863	0.00981
588.24	0.00321	0.0086	0.0098
595.08	0.00321	0.00858	0.00977
601.92	0.0032	0.00854	0.00974
608.76	0.00318	0.00853	0.00971
615.6	0.00317	0.00849	0.00971
622.44	0.00315	0.00849	0.00967
629.28	0.00313	0.00845	0.00962
636.12	0.00312	0.00844	0.00962
642.96	0.00309	0.0084	0.00959
649.8	0.00307	0.00838	0.00956
656.64	0.00305	0.00835	0.00954
663.48	0.00305	0.00833	0.00951
670.32	0.00301	0.00832	0.00948
677.16	0.003	0.00828	0.00946
684	0.00298	0.00825	0.00943
690.84	0.00297	0.00823	0.00941
697.68	0.00297	0.00822	0.00937
704.52	0.00294	0.00818	0.00935
711.36	0.00292	0.00816	0.00932
718.2	0.00291	0.00815	0.0093
725.04	0.0029	0.00812	0.00927
731.88	0.00287	0.0081	0.00923
738.72	0.00287	0.00808	0.00922
745.56	0.00285	0.00805	0.00917
752.4	0.00282	0.00804	0.00916
759.24	0.0028	0.008	0.00912
766.08	0.0028	0.008	0.00909
772.92	0.00277	0.00796	0.00906
779.76	0.00275	0.00793	0.00904
786.6	0.00275	0.00791	0.00901
793.44	0.00273	0.00788	0.00898
800.28	0.00271	0.00787	0.00894
807.12	0.0027	0.00784	0.00892
813.96	0.00268	0.00781	0.0089
820.8	0.00266	0.00779	0.00886
827.64	0.00265	0.01066	0.00882
834.48	0.00263	0.01061	0.00878
841.32	0.00262		0.00876
848.16	0.00261		0.00871
855	0.00259		0.00869
861.84	0.00259		0.00865
868.68	0.00258		0.00862
875.52	0.00256		0.00858
882.36	0.00254		0.00855
889.2	0.00252		0.00851
896.04	0.00252		0.00847
902.88	0.00249		0.00844
909.72	0.00248		0.00839
916.56	0.00245		0.00836
923.4	0.00245		0.00832
930.24	0.00244		0.00828
937.08			0.00823
943.92			0.00821
950.76			0.00816

Table S6. NMR Data Points for VTNA plots of the product concentrations. The concentrations were obtained using HMDSO as internal standard.

Time (min)	$[P]_{6mM}$ (mM)	$[P]_{12.5mM}$ (mM)	$[P]_{16mM}$ (mM)
0	-0.29414	-0.05168	0.12052
6.84	-0.10467	0.34322	0.56339
13.68	-0.06803	0.61433	0.79662
20.52	0.03106	0.907	1.14516
27.36	0.15299	1.20517	1.53732
34.2	0.29864	1.47131	2.06275
41.04	0.37616	1.75976	2.50101
47.88	0.50018	2.09303	3.07005
54.72	0.69098	2.39895	3.63696
61.56	0.75557	2.68918	4.18494
68.4	0.97443	2.99312	4.72475
75.24	1.10771	3.27036	5.16876
82.08	1.22827	3.5684	5.65054
88.92	1.3962	3.86723	6.06307
95.76	1.50881	4.14713	6.58073
102.6	1.70899	4.45266	7.01762
109.44	1.82753	4.76009	7.5267
116.28	1.92498	5.02474	7.98873
123.12	2.07445	5.3362	8.49337
129.96	2.23578	5.63582	8.97441
136.8	2.41323	5.93862	9.41264
143.64	2.47753	6.20043	9.89467
150.48	2.6364	6.51875	10.36785
157.32	2.74595	6.82723	10.84442
164.16	2.83427	7.12887	11.30515
171	2.97602	7.39359	11.75562
177.84	3.02393	7.66716	12.23013
184.68	3.16107	7.98539	12.64775
191.52	3.22496	8.24362	13.15778
198.36	3.38352	8.5268	13.58512
205.2	3.48401	8.82237	14.04042
212.04	3.55857	9.11689	14.44134
218.88	3.67415	9.37457	14.93916
225.72	3.77096	9.68347	15.3587
232.56	3.89326	9.92282	15.82366
239.4	3.97897	10.20534	16.26234
246.24	4.12337	10.50211	16.72716
253.08	4.19241	10.74245	17.17044
259.92	4.30229	11.04702	17.58778
266.76	4.37822	11.30599	18.01777
273.6	4.50673	11.55428	18.48343
280.44	4.60348	11.85514	18.91166
287.28	4.69138	12.11295	19.39181
294.12	4.79662	12.39125	19.75699
300.96	4.9483	12.64221	20.19279
307.8	4.98135	12.88661	20.6704
314.64	5.08766	13.1916	21.06257
321.48	5.20718	13.46073	21.50191
328.32	5.30648	13.69785	21.89074
335.16	5.39801	13.97308	22.3606
342	5.50516	14.19884	22.81983
348.84	5.58481	14.44742	23.25018
355.68	5.65483	14.70129	23.63225
362.52	5.77737	14.93973	24.02306
369.36	5.86994	15.17203	24.43995
376.2	5.95591	15.43594	24.9001
383.04	6.02176	15.69055	25.32298

389.88	6.15988	15.96151	25.71412
396.72	6.26005	16.17832	1.40939
403.56	6.31606	16.41922	26.51082
410.4	6.40496	16.67077	26.97161
417.24	6.52794	16.9098	27.32513
424.08	6.59837	17.13185	27.79543
430.92	6.71042	17.33972	28.208
437.76	6.8234	17.59841	28.60131
444.6	6.87198	17.82246	28.98406
451.44	6.97233	18.06235	29.35567
458.28	7.06781	18.25009	29.80303
465.12	7.17713	18.50323	30.18199
471.96	7.24494	18.75004	30.59221
478.8	7.29038	18.94964	31.01468
485.64	7.41233	19.17331	31.35722
492.48	7.50636	19.3672	31.78159
499.32	7.60288	19.59333	32.14815
506.16	7.68672	19.82878	32.55469
513	7.72146	20.03669	32.94158
519.84	7.84989	20.23843	33.3933
526.68	7.94347	20.45626	33.75985
533.52	8.00051	20.66672	34.10779
540.36	8.0609	20.85974	34.51043
547.2	8.16394	21.08688	34.80641
554.04	8.25571	21.29502	35.2789
560.88	8.33281	21.52596	35.60324
567.72	8.41237	21.71935	36.04496
574.56	8.48516	21.91357	36.36389
581.4	8.57836	22.10737	36.77448
588.24	8.56615	22.30426	37.13947
595.08	8.747	22.52591	37.5034
601.92	8.81607	22.6827	37.8704
608.76	8.88189	22.89748	38.20701
615.6	8.9169	23.10413	38.62598
622.44	9.06798	23.29446	38.96013
629.28	9.09047	23.45286	39.27828
636.12	9.18908	23.7124	39.67857
642.96	9.20607	23.88222	40.02281
649.8	9.30289	24.05765	40.38709
656.64	9.40444	24.25341	40.76582
663.48	9.47881	24.43993	41.1022
670.32	9.53871	24.61798	41.42313
677.16	9.63928	24.78315	41.76316
684	9.66644	24.97322	42.13284
690.84	9.73457	25.16639	42.51402
697.68	9.88287	25.3381	42.8114
704.52	9.90864	25.50722	43.21972
711.36	9.9683	25.67792	43.5378
718.2	10.08624	25.88725	43.90044
725.04	10.16288	26.03043	44.15036
731.88	10.16181	26.23824	44.5512
738.72	10.34759	26.41107	44.88738
745.56	10.37759	26.58225	45.16213
752.4	10.41775	26.75695	45.56633
759.24	10.48788	26.9155	45.89578
766.08	10.56656	27.10708	46.22207
772.92	10.62908	27.25094	46.52207
779.76	10.66696	27.41249	46.86744
786.6	10.77967	27.59916	47.17591
793.44	10.85358	27.74927	47.51585
800.28	10.90027	27.93195	47.84278

807.12	10.97668	28.08141	48.14461
813.96	11.0456	28.22462	48.54973
820.8	11.11222	28.36394	48.7868
827.64	11.18762		49.13413
834.48	11.22998		49.37678
841.32	11.30261		49.67669
848.16	11.3554		50.00117
855	11.43158		50.3061
861.84	11.52127		50.58191
868.68	11.56887		50.91124
875.52	11.66027		51.20201
882.36	11.7173		51.48915
889.2	11.77179		51.77992
896.04	11.8481		52.05808
902.88	11.86873		52.36734
909.72	11.92313		52.59455
916.56	12.01383		52.85815
923.4	12.07503		53.1922
930.24	12.11899		53.51238
937.08			53.71417
943.92			54.02629
950.76			54.29189

VTNA Plots using the initial titanium precatalyst concentration:

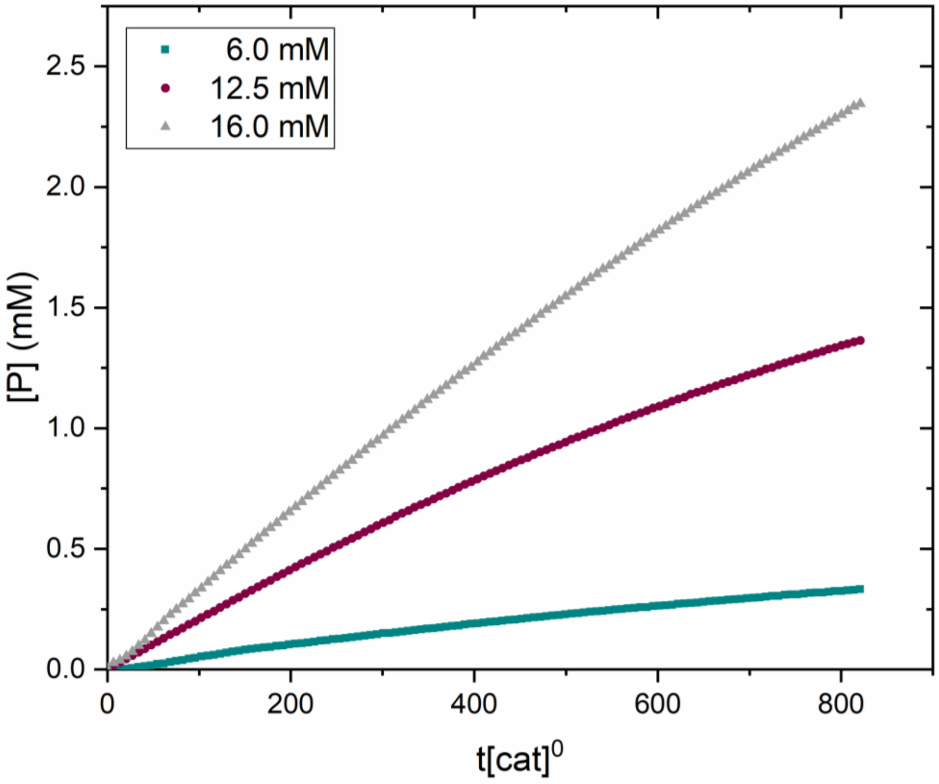

Figure S13. VTNA-plot for zeroth order in titanium.

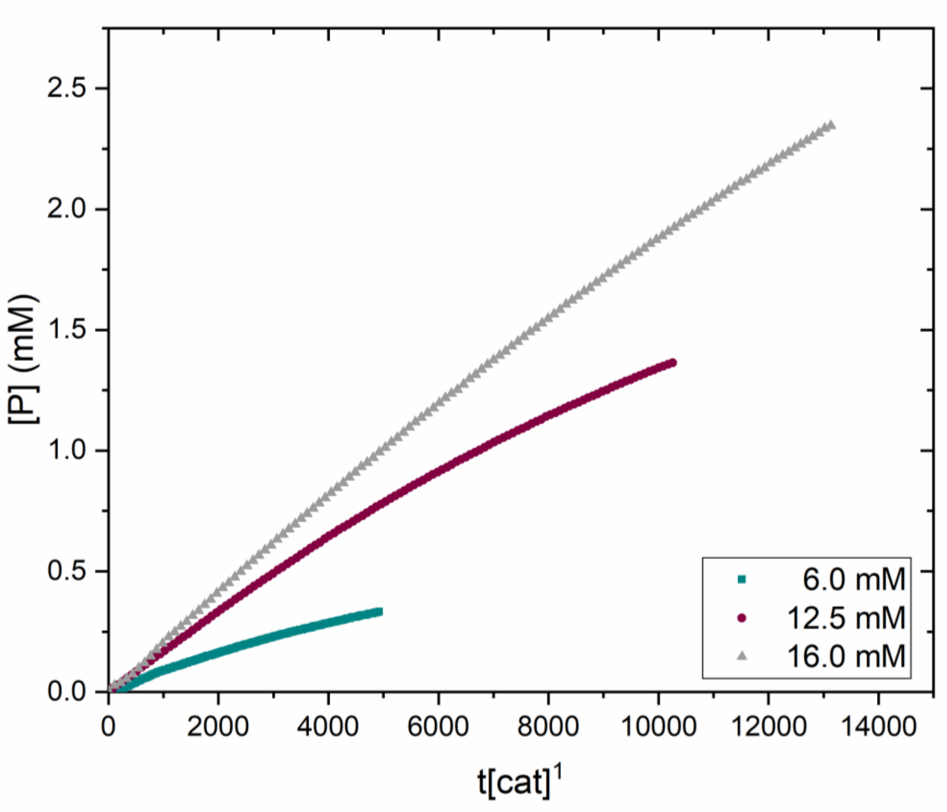

Figure S14. VTNA-plot for first order in titanium.

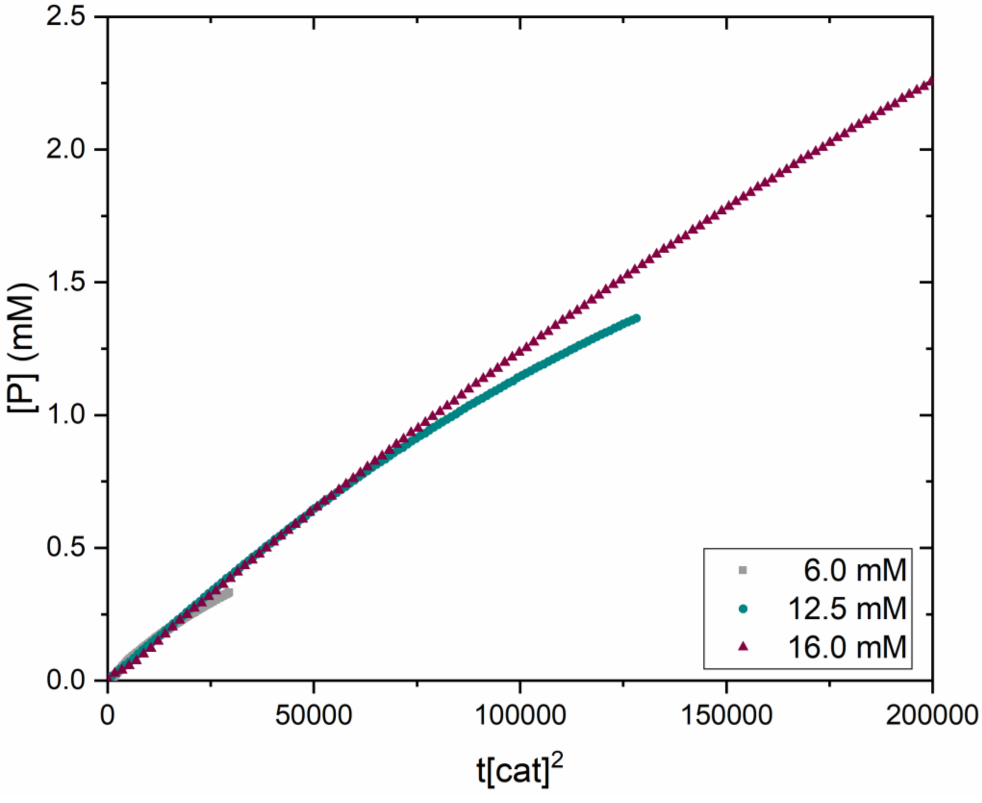

Figure S15. VTNA-plot for second order in titanium.

VTNA Plots for the order in the substrate:

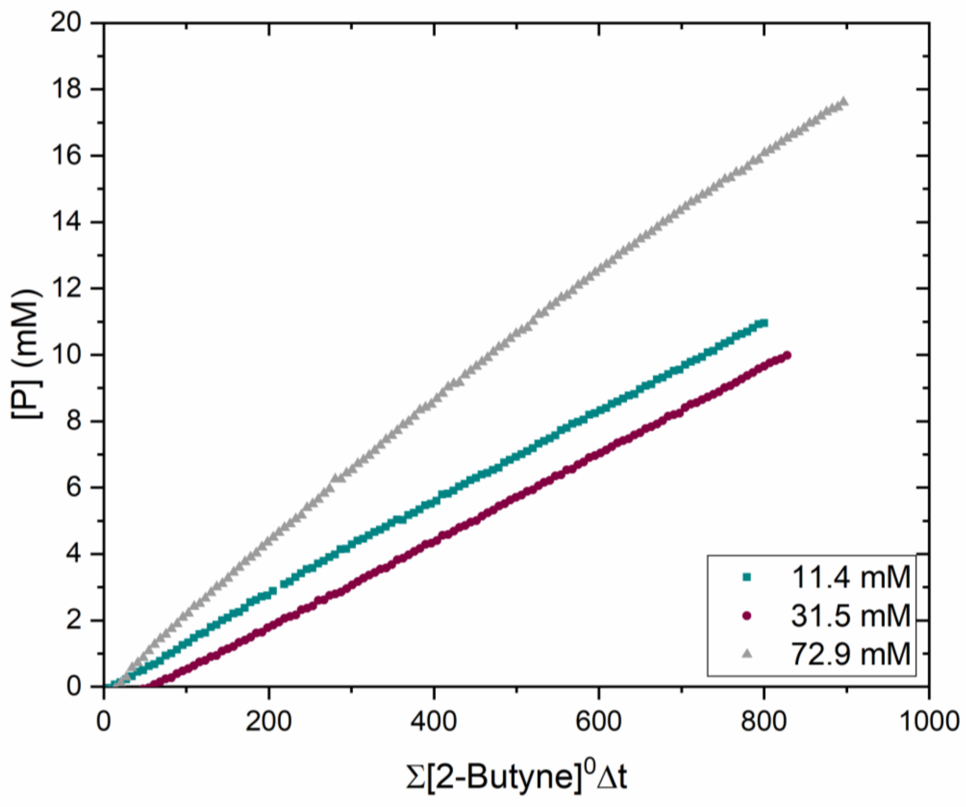

Figure S16. VTNA-Plot for the zeroth order in 2-butyne.

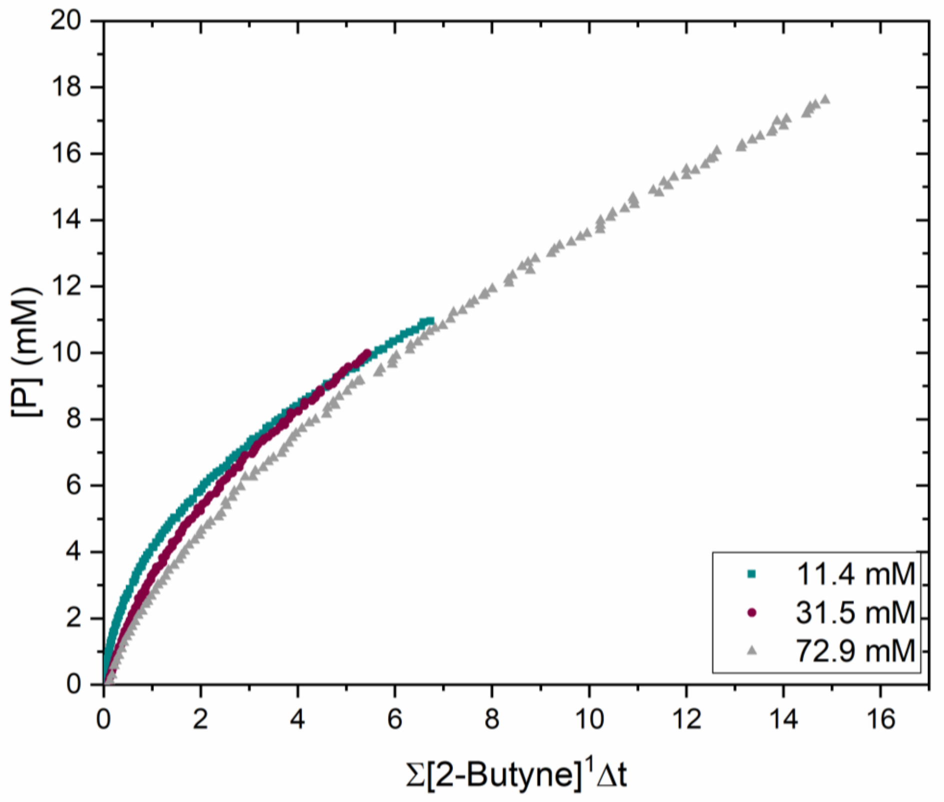

Figure S17. VTNA-Plot for the first order in 2-butyne.

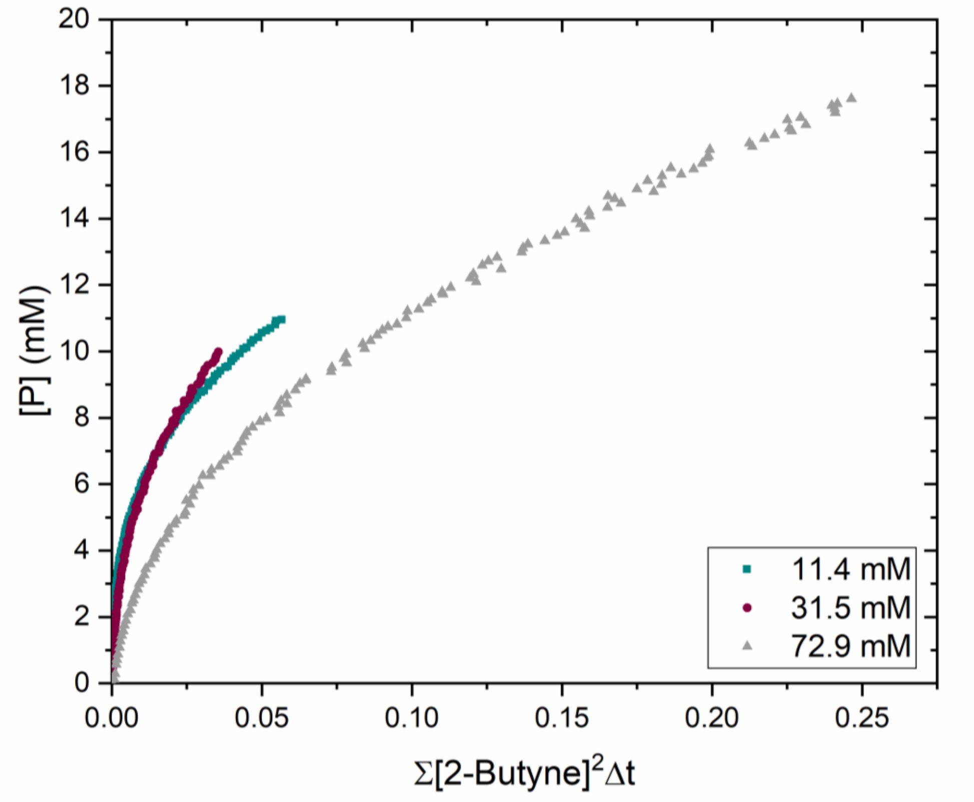

Figure S18. VTNA-Plot for the second order in 2-butyne.

For the processing of the 2-butyne concentrations the following equation was used.

$$\Sigma[\frac{c_{n+1} - c_n}{2}]^{\alpha}\Delta t$$

Where c is the concentration of 2-butyne (mM), α is the order of the substrate, and t is the time (min). The plotted data points can be found in Table S7.

Table S7. Data Points of the varied 2-butyne concentrations.

Time (min)	$[butyne]_{11.4mM}$ (M)	[P] of initial $[butyne]_{11.4mM}$ (M)	$[butyne]_{31.5mM}$ (M)	[P] of initial $[butyne]_{31.5mM}$ (M)	$[butyne]_{72.9mM}$ (M)	[P] of initial $[butyne]_{72.9mM}$ (M)
0	0.011437	-2.39606E-4	0.031529	-7.45346E-4	0.072937	-2.95536E-4
6.84	0.011271	-3.77163E-5	0.03107	-5.29687E-4	0.07191	-1.55487E-4
13.68	0.011257	5.5898E-5	0.031035	-4.69758E-4	0.071775	-5.62687E-5
20.52	0.011238	1.2801E-4	0.031028	-3.90754E-4	0.071692	1.11087E-4
27.36	0.011231	2.18961E-4	0.031028	-2.6164E-4	0.07167	2.84275E-4
34.2	0.011228	3.05384E-4	0.031018	-2.04923E-4	0.071646	5.64354E-4
41.04	0.011226	4.42338E-4	0.031022	-1.67711E-4	0.071654	7.10878E-4
47.88	0.011218	4.92449E-4	0.031001	-7.01895E-5	0.071627	8.70433E-4
54.72	0.011199	6.18792E-4	0.030986	-3.15825E-5	0.071574	0.00108
61.56	0.011191	6.65785E-4	0.030965	6.85995E-5	0.071575	0.00127
68.4	0.01118	7.68191E-4	0.030977	1.45822E-4	0.071539	0.00144
75.24	0.011161	9.30467E-4	0.030959	2.39863E-4	0.071507	0.00158
82.08	0.01116	9.97348E-4	0.03099	2.66899E-4	0.071504	0.00175
88.92	0.011128	0.00111	0.030945	3.95437E-4	0.071477	0.0019
95.76	0.011125	0.00125	0.030936	4.68999E-4	0.071449	0.00209
102.6	0.011121	0.00133	0.030935	5.42136E-4	0.071386	0.00221
109.44	0.0111	0.00146	0.030931	6.28427E-4	0.071366	0.00241
116.28	0.011087	0.00158	0.030909	7.49906E-4	0.071364	0.0025
123.12	0.01107	0.00162	0.030923	7.9113E-4	0.071343	0.00266
129.96	0.011058	0.0018	0.030906	9.02075E-4	0.071305	0.00283
136.8	0.01104	0.00186	0.030913	9.39807E-4	0.071302	0.003
143.64	0.01103	0.002	0.030875	0.00107	0.071261	0.00311
150.48	0.01101	0.00208	0.030887	0.00115	0.071238	0.00327
157.32	0.011001	0.00219	0.030862	0.00121	0.071237	0.00345
164.16	0.010995	0.00224	0.03086	0.00134	0.071169	0.00359
171	0.010976	0.00237	0.030852	0.0014	0.071114	0.00376
177.84	0.010966	0.00254	0.030848	0.0015	0.071136	0.00389
184.68	0.010943	0.00261	0.030844	0.00162	0.071131	0.00403
191.52	0.010928	0.0027	0.030831	0.00163	0.0711	0.0042
198.36	0.010924	0.00274	0.030823	0.00178	0.071048	0.00435
205.2	0.010906	0.00288	0.030806	0.00186	0.071017	0.00449
212.04		-9.87E-4	0.030801	0.00195	0.071041	0.00464
218.88	0.010879	0.00308	0.030779	0.00205	0.07099	0.00479
225.72	0.010858	0.00316	0.030801	0.00212	0.070984	0.00491
232.56	0.010859	0.0033	0.030794	0.00216	0.070903	0.00505
239.4	0.010842	0.00341	0.030768	0.00231	0.070911	0.00517
246.24	0.010824	0.00353	0.030759	0.00236	0.070879	0.00539
253.08	0.010811	0.00357	0.030756	0.00243	0.070954	0.0055
259.92	0.010807	0.00371	0.030764	0.0026	0.070899	0.00564
266.76	0.01079	0.00378	0.03072	0.00261	0.070919	0.00582
273.6	0.010777	0.0039	0.030741	0.00275	0.070876	0.00596
280.44	0.01076	0.00397	0.030709	0.0028	0.070857	0.00625
287.28	0.010735	0.00413	0.030726	0.00285	0.070798	0.00626
294.12	0.010735	0.00415	0.030723	0.00295	0.070811	0.00643
300.96	0.010716	0.00429	0.030706	0.00307	0.070755	0.00654
307.8	0.010692	0.0044	0.030684	0.00317	0.070732	0.00672
314.64	0.010691	0.00445	0.030695	0.00327	0.070713	0.00683
321.48	0.010686	0.00455	0.03068	0.00336	0.070657	0.00696
328.32	0.010669	0.00466	0.030665	0.00344	0.070674	0.00711
335.16	0.010655	0.00473	0.030667	0.00354	0.070662	0.00727
342	0.010647	0.00482	0.030645	0.00358	0.070666	0.00745
348.84	0.010631	0.00493	0.030619	0.00368	0.070666	0.00757
355.68	0.010615	0.00503	0.030635	0.00382	0.07064	0.00772
362.52	0.010607	0.00501	0.030617	0.00387	0.070602	0.00789
369.36	0.010584	0.00516	0.030615	0.00397	0.070575	0.00799
376.2	0.01058	0.00524	0.030601	0.00407	0.070497	0.00815

383.04	0.010566	0.00534	0.03058	0.00417	0.070531	0.00834
389.88	0.010547	0.00546	0.030591	0.00429	0.070489	0.00842
396.72	0.010539	0.0055	0.030578	0.00432	0.070552	0.00851
403.56	0.010523	0.00559	0.030561	0.0044	0.070534	0.00868
410.4	0.010495	0.00579	0.030556	0.00456	0.070494	0.00883
417.24	0.010488	0.00581	0.03055	0.00458	0.070486	0.00903
424.08	0.010483	0.00591	0.030552	0.00469	0.07047	0.00913
430.92	0.010475	0.00603	0.030546	0.0048	0.070486	0.00916
437.76	0.010463	0.00611	0.030533	0.00485	0.070353	0.00938
444.6	0.010446	0.00621	0.030526	0.00496	0.070369	0.00952
451.44	0.010432	0.00628	0.030513	0.00501	0.070303	0.00965
458.28	0.010418	0.00639	0.030491	0.00514	0.070338	0.00978
465.12	0.010409	0.00644	0.03046	0.00524	0.070346	0.00991
471.96	0.010392	0.00652	0.030497	0.00532	0.070265	0.01008
478.8	0.010375	0.0066	0.030467	0.00545	0.070295	0.01022
485.64	0.010368	0.00675	0.03045	0.00552	0.070272	0.01032
492.48	0.010352	0.00683	0.030441	0.00563	0.070258	0.01049
499.32	0.010342	0.00692	0.030438	0.00571	0.07025	0.01064
506.16	0.010325	0.00699	0.030404	0.00577	0.07024	0.01073
513	0.010309	0.00709	0.030403	0.00589	0.070215	0.01082
519.84	0.010292	0.00718	0.030398	0.00592	0.070189	0.01101
526.68	0.010285	0.00732	0.030408	0.00605	0.070202	0.01121
533.52	0.01028	0.00739	0.030389	0.00616	0.070167	0.01126
540.36	0.010251	0.00748	0.030381	0.00621	0.070147	0.01146
547.2	0.010235	0.00757	0.03037	0.00635	0.070147	0.01157
554.04	0.010225	0.00772	0.03036	0.00638	0.070115	0.01172
560.88	0.010214	0.00779	0.030352	0.00653	0.070135	0.01179
567.72	0.01019	0.00791	0.030335	0.00655	0.070116	0.01192
574.56	0.010186	0.00798	0.030338	0.00669	0.07003	0.0121
581.4	0.010176	0.00804	0.030335	0.00677	0.070069	0.01221
588.24	0.010163	0.00819	0.03033	0.0069	0.070074	0.01234
595.08	0.010145	0.00823	0.030291	0.00696	0.069983	0.01248
601.92	0.010138	0.00833	0.03029	0.00704	0.070073	0.01259
608.76	0.01013	0.00839	0.030292	0.00713	0.070065	0.01272
615.6	0.010111	0.00852	0.030287	0.00724	0.070048	0.01283
622.44	0.010096	0.00859	0.030268	0.00734	0.069974	0.01298
629.28	0.010087	0.00867	0.030265	0.00741	0.069986	0.01311
636.12	0.010066	0.00877	0.030253	0.00747	0.069984	0.01323
642.96	0.010053	0.00881	0.030238	0.00758	0.069941	0.01333
649.8	0.010029	0.00895	0.030225	0.00765	0.069915	0.01348
656.64	0.010034	0.00907	0.03021	0.00778	0.069906	0.01359
663.48	0.010009	0.00911	0.030194	0.00784	0.069854	0.0137
670.32	0.010005	0.00925	0.030215	0.00791	0.069885	0.01383
677.16	0.009993	0.00931	0.030192	0.00802	0.069914	0.01399
684	0.009981	0.00941	0.030185	0.00815	0.069884	0.01408
690.84	0.009956	0.00951	0.030202	0.00819	0.069903	0.01421
697.68	0.009947	0.00955	0.030169	0.00824	0.06986	0.01434
704.52	0.009933	0.00969	0.030145	0.00841	0.069833	0.01446
711.36	0.009924	0.00978	0.030155	0.00851	0.069867	0.01459
718.2	0.009919	0.00985	0.030124	0.00856	0.069902	0.01467
725.04	0.009901	0.00993	0.030117	0.00865	0.069781	0.01482
731.88	0.009889	0.01007	0.030122	0.00873	0.069844	0.0149
738.72	0.009876	0.01011	0.03011	0.00881	0.069788	0.01503
745.56	0.00986	0.01025	0.030122	0.00888	0.069842	0.01514
752.4	0.009851	0.01034	0.030085	0.00901	0.069815	0.01528
759.24	0.009831	0.01042	0.030075	0.00907	0.069775	0.01533
766.08	0.00982	0.01055	0.030072	0.00917	0.069756	0.01549
772.92	0.009805	0.01062	0.030076	0.00927	0.069832	0.01552
779.76	0.00979	0.01069	0.030062	0.00938	0.06976	0.01566
786.6	0.009772	0.0108	0.030062	0.00946	0.069762	0.01583
793.44	0.009775	0.01092	0.030048	0.00958	0.06977	0.01587

800.28	0.009755	0.01095	0.030021	0.00966	0.069781	0.01607
807.12			0.030011	0.00976	0.069685	0.01617
813.96			0.030015	0.00982	0.069706	0.01627
820.8			0.030014	0.00988	0.069682	0.0164
827.64			0.030007	0.00998	0.06967	0.01652
834.48				0.01007	0.069641	0.01664
841.32					0.069661	0.01671
848.16					0.069635	0.01682
855					0.069692	0.01697
861.84					0.069673	0.01704
868.68					0.069606	0.01719
875.52					0.06962	0.01731
882.36					0.069639	0.01741
889.2					0.06964	0.01746
896.04					0.06962	0.0176

Reaction Profile Starting from Half Conversion

A J Young NMR tube was charged with (^{iPr}PDA)Ti(C_4Me_4) (10.5 mg, 17.0 µmol, 10.0 mol% with respect to 2-butyne; 5 mol% with respect to the total amount of starting material and product (x2)), a solution of cyclobutene (18.5 mg, 0.17 mmol, 1.0 equiv.) in benzene-d_6 (0.24 M) and 2-butyne (13.0 µL, 0.17 mmol, 1.0 equiv.). The solution was diluted to a total volume of 1.53 mL. Using a syringe, 0.2 mL of this solution were transferred to the in-situ LED NMR setup and irradiated at 460 nm overnight. The obtained spectra were plotted in the same graph as the previously obtained plot for the standard conditions. The graph shows catalyst deactivation during the reaction.

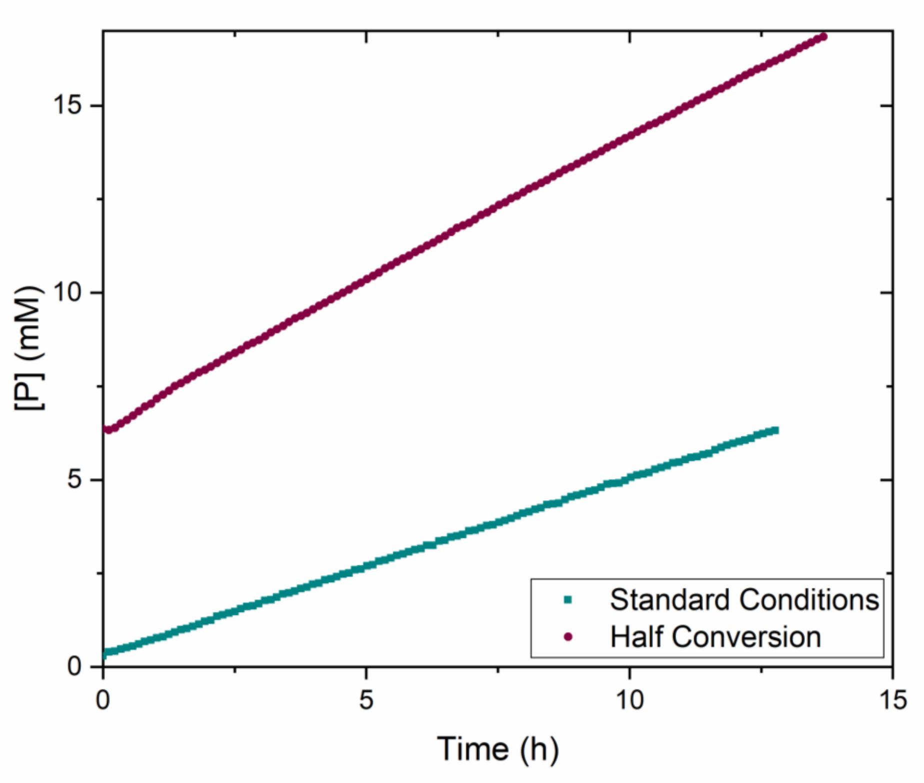

Figure S19. Reaction profile at half conversion compared to the profile starting without product.

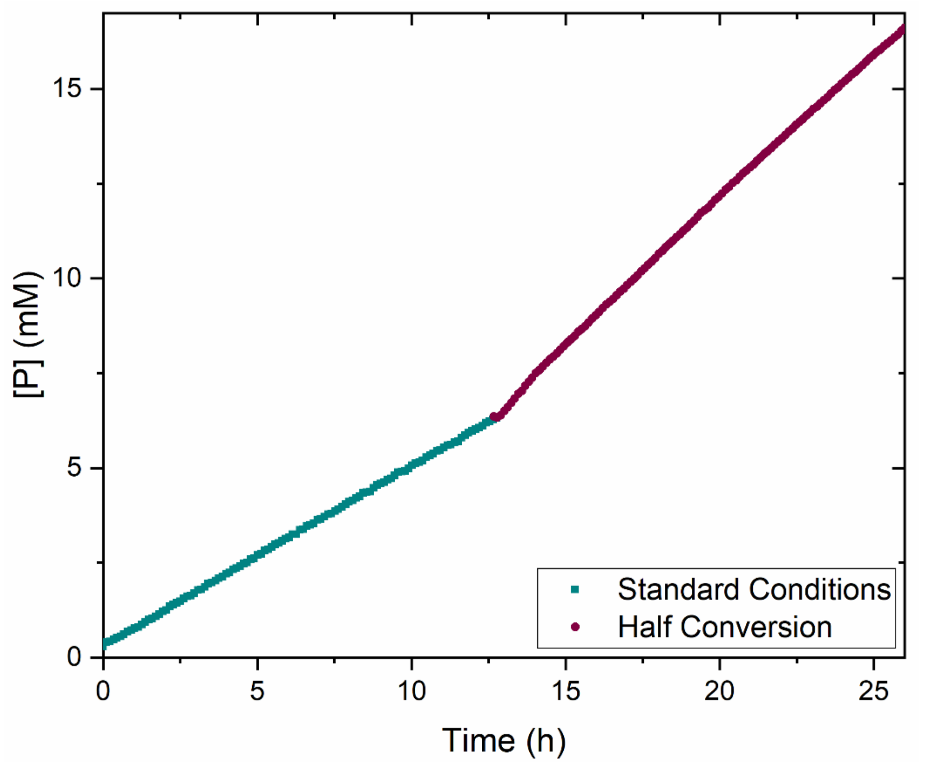

Figure S20. Reaction profile at half conversion compared to the profile starting without product. The starting point of the red curve was shifted by time + 12.6 h.

Table S8. Data Points for the plots in Figure S20.

Time (min)	[P] for standard conditions (M)	Time (min)	[P] for half conversion (M)
0	-1.16285E-4	424.8	0.22036
6.84	7.72255E-4	431.64	0.21866
13.68	0.00138	438.48	0.21809
20.52	0.00204	445.32	0.2175
27.36	0.00271	452.16	0.21735
34.2	0.00331	459	0.21675
41.04	0.00396	465.84	0.21667
47.88	0.00471	472.68	0.21654
54.72	0.0054	479.52	0.21605
61.56	0.00605	486.36	0.21613
68.4	0.00673	493.2	0.21581
75.24	0.00736	500.04	0.21612
82.08	0.00803	506.88	0.21545
88.92	0.0087	513.72	0.21537
95.76	0.00933	520.56	0.21557
102.6	0.01002	527.4	0.21537
109.44	0.01071	534.24	0.21502
116.28	0.01131	541.08	0.21516
123.12	0.01201	547.92	0.21494
129.96	0.01268	554.76	0.21464
136.8	0.01336	561.6	0.21423
143.64	0.01395	568.44	0.2139
150.48	0.01467	575.28	0.2138
157.32	0.01536	582.12	0.21401
164.16	0.01604	588.96	0.21399
171	0.01664	595.8	0.21379
177.84	0.01725	602.64	0.21353
184.68	0.01797	609.48	0.21314
191.52	0.01855	616.32	0.21326
198.36	0.01919	623.16	0.21309
205.2	0.01985	630	0.21288
212.04	0.02051	636.84	0.21266
218.88	0.02109	643.68	0.21243
225.72	0.02179	650.52	0.21239
232.56	0.02233	657.36	0.21225
239.4	0.02296	664.2	0.21226
246.24	0.02363	671.04	0.21191
253.08	0.02417	677.88	0.21173
259.92	0.02486	684.72	0.21152
266.76	0.02544	691.56	0.21142
273.6	0.026	698.4	0.21131
280.44	0.02667	705.24	0.21122
287.28	0.02725	712.08	0.21099
294.12	0.02788	718.92	0.2107
300.96	0.02844	725.76	0.21059
307.8	0.02899	732.6	0.2105
314.64	0.02968	739.44	0.21024
321.48	0.03029	746.28	0.21014
328.32	0.03082	753.12	0.20995
335.16	0.03144	759.96	0.20965
342	0.03195	766.8	0.20954
348.84	0.03251	773.64	0.20932
355.68	0.03308	780.48	0.20923
362.52	0.03361	787.32	0.20911
369.36	0.03414	794.16	0.20877
376.2	0.03473	801	0.20855
383.04	0.0353	807.84	0.20853

389.88	0.03591	814.68	0.20825
396.72	0.0364	821.52	0.20785
403.56	0.03694	828.36	0.20782
410.4	0.03751	835.2	0.20775
417.24	0.03805	842.04	0.20758
424.08	0.03855	848.88	0.20741
430.92	0.03901	855.72	0.20714
437.76	0.0396	862.56	0.20699
444.6	0.0401	869.4	0.20673
451.44	0.04064	876.24	0.20628
458.28	0.04106	883.08	0.20605
465.12	0.04163	889.92	0.20615
471.96	0.04219	896.76	0.20602
478.8	0.04264	903.6	0.20589
485.64	0.04314	910.44	0.20575
492.48	0.04358	917.28	0.20564
499.32	0.04408	924.12	0.20536
506.16	0.04461	930.96	0.20521
513	0.04508	937.8	0.20479
519.84	0.04554	944.64	0.20457
526.68	0.04603	951.48	0.20442
533.52	0.0465	958.32	0.20431
540.36	0.04693	965.16	0.20421
547.2	0.04745	972	0.20396
554.04	0.04791	978.84	0.20385
560.88	0.04843	985.68	0.20379
567.72	0.04887	992.52	0.20373
574.56	0.04931	999.36	0.20351
581.4	0.04974	1006.2	0.20361
588.24	0.05018	1013.04	0.20337
595.08	0.05068	1019.88	0.20325
601.92	0.05104	1026.72	0.20319
608.76	0.05152	1033.56	0.20302
615.6	0.05198	1040.4	0.20278
622.44	0.05241	1047.24	0.20256
629.28	0.05277	1054.08	0.20238
636.12	0.05335	1060.92	0.20214
642.96	0.05374	1067.76	0.20205
649.8	0.05413	1074.6	0.20178
656.64	0.05457	1081.44	0.20159
663.48	0.05499	1088.28	0.2014
670.32	0.05539	1095.12	0.20123
677.16	0.05576	1101.96	0.20113
684	0.05619	1108.8	0.20084
690.84	0.05662	1115.64	0.20075
697.68	0.05701	1122.48	0.20058
704.52	0.05739	1129.32	0.2005
711.36	0.05778	1136.16	0.20031
718.2	0.05825	1143	0.2002
725.04	0.05857	1149.84	0.19992
731.88	0.05904	1156.68	0.19971
738.72	0.05942	1163.52	0.1996
745.56	0.05981	1170.36	0.19941
752.4	0.0602	1177.2	0.19927
759.24	0.06056	1184.04	0.19902
766.08	0.06099	1190.88	0.19896
772.92	0.06131	1197.72	0.19895
779.76	0.06168	1204.56	0.1987
786.6	0.0621	1211.4	0.19856
793.44	0.06244	1218.24	0.19844
800.28	0.06285	1225.08	0.19824

807.12	0.06318	1231.92	0.198
813.96	0.06351	1238.76	0.19793
820.8	0.06382	1245.6	0.19765

6. EPR Measurements

In an argon glovebox, (^{iPr}PDA)Ti(C_4Me_4) (11.3 mg, 18.0 μmol, 5 mol%) was weighed into a vial. The complex was dissolved in 1.5 mL benzene-d_6 and 2-butyne (29.0 mL, 0.37 mmol, 1.0 equiv.) were added. From this solution 0.2 mL were transferred to the *in situ* LED NMR set-up and the tube was sealed. EPR measurements were performed at room temperature before irradiation (green curve) and while irradiating for 30 minutes (red curve).

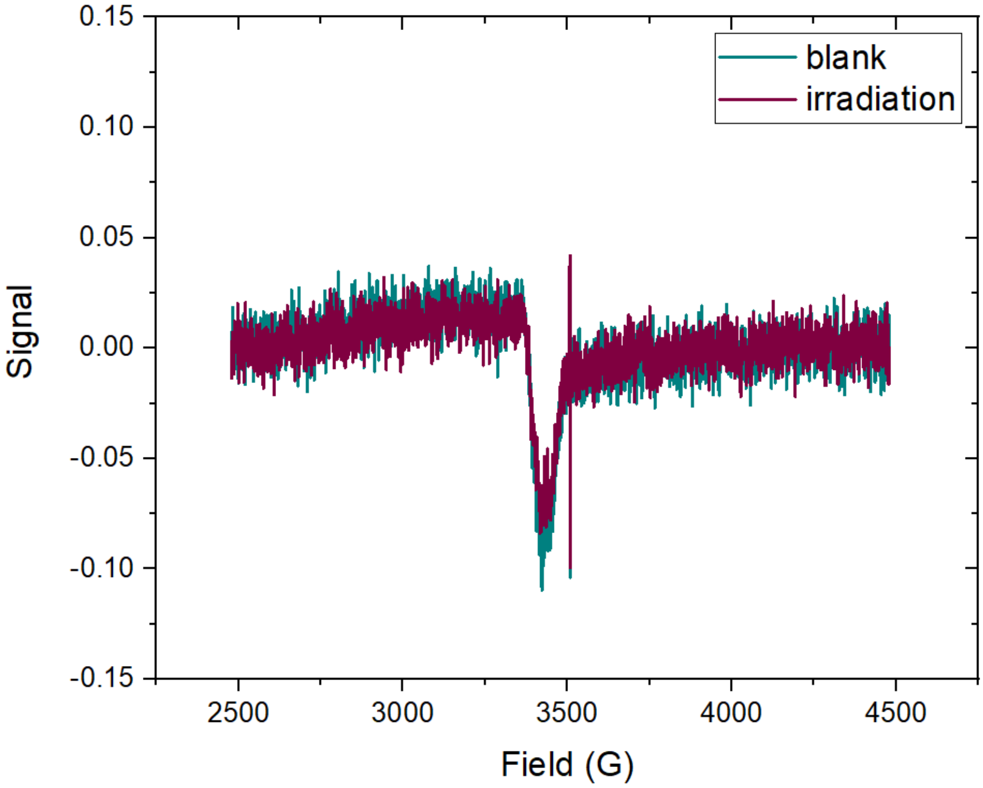

Figure S21. EPR spectrum before and during irradiation with 467 nm Kessil lamp.

7. DFT Calculations

The TD-DFT calculations were performed using the ORCA program package (version 5.0).[12] Geometry optimizations were performed at the B3LYP-D4[13] and TPSSh[14] level of theory. Natural transition orbitals and single-point calculations were performed on the optimized geometries. The triple-zeta basis set def2-TZVP was used for all elements.[15] Additionally, the resolution-of-the-identity (RIJCOSX)[16] approach was used as an auxiliary basis set. All molecular orbital plots were generated using the program ChemCraft.

XYZ Coordinates of (^{iPr}PDA)Ti(C_4Me_4)

Ti	12.86892553126095	10.15646491774512	9.82617050392511
N	13.84959803401336	10.44959067582349	7.87193175689739
N	13.33028446217939	8.35697441247037	9.11726075646780
N	13.57859241690243	12.01422421137143	9.79928058965269
C	13.99600083639135	9.39785567334565	7.06565995174980
C	14.22553526799679	11.67643300998844	7.51351912418229
C	13.36037671419193	7.18528533820502	9.92043993356196
C	14.53168774718266	6.87236403910165	10.64923421777444
C	13.54135747626655	12.86048508680354	10.94200939035303
C	14.55615937082142	12.75157349799293	11.92174751708961
C	12.40590625446860	9.71619582770175	11.78937652232516
C	12.24234106441373	6.33217036088561	9.98528909801360
C	12.51554842943038	13.81451644717052	11.09500612454706
C	12.54959969918771	14.67799871964831	12.18791173296659
H	11.76831208759822	15.41921398338742	12.30603303770412
C	14.00131853838604	12.69209349196059	8.58921628092140
H	14.91630716741604	13.28161132050159	8.74178053282304
H	13.25214520361701	13.41418701444345	8.23103937933469
C	15.64518256357330	11.69620697077707	11.80896917155916
H	15.16846069485799	10.78978169547588	11.43091434232293
C	13.55617347319443	14.59726263770150	13.13258858881079
H	13.56870234225804	15.27454487488502	13.97778849959983
C	11.04682685320918	9.85016718151399	11.78113557370178
C	14.54098219502822	13.62924385406044	13.00134244041119
H	15.30932326264413	13.56145718382314	13.75862146732760
C	14.52552678748427	9.55414541984933	5.79408145924454
H	14.64576786064163	8.70105062301792	5.13961809477966
C	10.90071187898164	10.38443909540670	9.35079527736197
C	11.36069171608614	13.91158871680869	10.11644253328528
H	11.46042618543619	13.08795736987090	9.41073542189208
C	14.91363750282587	10.82847545469202	5.39361734041758
H	15.34357597790124	10.97783663308861	4.41101547696543
C	14.76998471350074	11.90722903073208	6.25825143881806
H	15.08193194203567	12.90201111225230	5.96901014981659
C	13.17208017157706	9.33313519021263	13.02057851199753
H	12.79967201435298	8.40492083877764	13.46640298219343
H	13.11935795883204	10.11064529361369	13.79102250436348
H	14.22603964215732	9.17498582757705	12.80106523549279
C	15.75455382625968	7.77409434837021	10.59792443202178

H	15.39623139147359	8.79818923204268	10.47700061836842
C	13.55258682932569	8.11726369906198	7.70352419111888
H	12.63961783092116	7.77274971030322	7.19063986965296
H	14.29676641694499	7.33118124404051	7.52542082880252
C	10.01251414957021	13.72944541895113	10.82368116354050
H	9.79705676209908	14.56094895002679	11.49850481032430
H	10.00427378250302	12.81036001249388	11.40715315074137
H	9.20134771638069	13.67743792785967	10.09316480323761
C	10.27082161451800	10.18365636245087	10.53332550955782
C	10.96124107375873	6.65364882462798	9.24109473148769
H	11.06712223237119	7.66014995391777	8.83845372565193
C	12.31748172632270	5.17105776812241	10.75071795815018
H	11.46160965281418	4.50866997523472	10.79970985911496
C	14.55640904873001	5.70623726456323	11.40865063692268
H	15.44268889389899	5.45430271967590	11.97512588706526
C	13.46378701235620	4.85351716466146	11.45779883802440
H	13.50632819167387	3.94952448351008	12.05298771754701
C	16.29463567606232	11.34273688201353	13.14910016139580
H	16.94931266090611	10.47940944329575	13.02591753732085
H	15.55052679401334	11.09623197490879	13.90699744172335
H	16.90891341272138	12.16202351188145	13.52991184876515
C	11.38183647624376	15.23115654373314	9.33235201559802
H	10.57942600027924	15.25131362823120	8.59029484203826
H	12.33041697486838	15.37672890243523	8.81162073296369
H	11.24072976647750	16.08617825583248	9.99797858178135
C	16.63952338929020	7.46501193667747	9.38040771221237
H	16.99338675470998	6.43155443467409	9.41799082125814
H	17.51343148651613	8.12155666184164	9.36537464857112
H	16.10606811422237	7.60242974893470	8.44212977567079
C	16.73180253489443	12.08642345002534	10.79707483121650
H	17.19749244109786	13.03411868448811	11.07877982062647
H	16.32806062124382	12.18947202251441	9.79251264004077
H	17.51339492231678	11.32283719771443	10.76430412164510
C	16.60989005491699	7.72717074354371	11.86697370975490
H	16.01466254657845	7.87974332662250	12.76759183202176
H	17.37249041038564	8.50700590444974	11.82617153295974
H	17.13326076351355	6.77368847110929	11.96845375382831
C	10.73173327003349	5.68420451780749	8.07337334942821
H	11.58277972227230	5.67217214222582	7.38960666146318
H	9.84153135444469	5.96548668587827	7.50435961592134
H	10.58807861853640	4.66245321937559	8.43389780364326
C	9.74826851586738	6.66913560831869	10.17762691426475
H	9.52231084531370	5.67161665325912	10.56164540910339
H	8.86230987880773	7.02769359962715	9.64816848892633
H	9.92706145176961	7.32422470253765	11.02934292966446
C	10.22683667158072	10.71046065617158	8.05232491220053
H	9.51118775211732	11.53516299155264	8.14277047899120
H	9.67195199348840	9.85034616071630	7.65906733907464
H	10.95023171894101	10.99539325337078	7.28553483287828
C	10.22568639841626	9.66776668870234	13.03882688121572
H	9.71915813545756	10.59759235156243	13.31485484187022
H	10.84357174970447	9.36876010477413	13.88089562712369
H	9.44513969369270	8.91312539264905	12.91043593349567

C	8.76552462937704	10.26228290066848	10.65818319089720
H	8.33929966630107	9.29064674449904	10.92128861802192
H	8.30790008414293	10.57837129452197	9.72340152462396
H	8.45508585825004	10.96375451425455	11.43557459381609

XYZ Coordinates of (^{iPr}PDA)Ti($CH_2C_7H_{10}$)

Ti	2.83393041316837	7.39773318438917	18.12485461415072
N	2.83674068202035	8.88321594455547	16.79611278624095
C	3.99733670964122	9.67841523872413	16.45541364950287
H	3.90088199790037	10.71900105147767	16.80473264547269
H	4.14071132613758	9.75250985475307	15.36791743100441
N	4.94008886053655	7.97379112452981	17.78977225577348
C	5.21334369774396	9.06211739867062	17.06421591551693
N	3.96720252133793	5.98977277018674	18.94857612387928
C	6.51785232336416	9.50428394440895	16.90776706881531
H	6.72773347001048	10.38851448573909	16.32074268781592
C	7.53976408019203	8.77908138773122	17.50830818557386
H	8.56861444038841	9.09751903928102	17.39662844723279
C	7.24052336350069	7.63694987266776	18.24058594268361
H	8.02041829687285	7.04910038885835	18.70616486592594
C	5.91223785578002	7.25761305871434	18.36107194607819
C	5.40504278266480	6.05917849509026	19.09099505662390
H	5.90806865824302	5.16181951659560	18.70470400743291
H	5.71383323707789	6.12959104779850	20.14403544251654
C	1.71490871091850	9.19488032578808	15.97030969054095
C	0.77755697626289	10.16897654871991	16.36170199904635
C	-0.30049342721139	10.44480248039488	15.52309447130953
H	-1.02674837073727	11.19123299460946	15.82023149098485
C	-0.45889442003627	9.78677363196964	14.31547031866156
H	-1.30442379418238	10.01207506601538	13.67714886375306
C	0.47397216334020	8.83793071587131	13.92844268140759
H	0.34900874868320	8.32715216570583	12.98130414874687
C	1.56543078642716	8.52869581452859	14.73421091148195
C	0.93368284505420	10.94385483880203	17.65384141957449
H	1.74107888519390	10.47194135345946	18.20908948301416
C	1.33333017100038	12.40067036200466	17.37903816183966
H	2.24224107885621	12.45860688974864	16.77649284896151
H	1.51131723549124	12.93500296698286	18.31598206682495
H	0.54596141904159	12.92962163154644	16.83624383779134
C	-0.32681743810138	10.88126225253384	18.52213696601028
H	-1.16586467041256	11.39966169526555	18.05231321587420
H	-0.14779878851596	11.35907498976288	19.48817356371691
H	-0.62394461839591	9.84899064901041	18.70240554925048
C	2.56602202323709	7.49174540173801	14.25627168995937
H	3.29102539519094	7.34244969656759	15.05747026426046
C	3.33208256249766	7.98094982080811	13.01897592891941
H	4.09484081278123	7.25484848075967	12.72637494574423
H	3.82626492450931	8.93670616198305	13.20480278802459
H	2.65986686460368	8.11982102678686	12.16886512416227
C	1.90698131716728	6.13494036128793	13.98081789249804
H	1.19265658849287	6.19693188055605	13.15681071910051
H	1.37367908762187	5.77052698362455	14.85782002276011

H	2.66195431186472	5.39359934139648	13.70763754235180
C	3.39684324836883	4.80615843435194	19.50526483198475
C	3.12665575203417	4.72291654938449	20.88620334994423
C	2.56139243572620	3.55678738204468	21.39561183279985
H	2.34542105001339	3.48887620420026	22.45443147410546
C	2.27667832519465	2.47896041724530	20.57627790898142
H	1.83549576339582	1.58013847407293	20.98960639303388
C	2.56620523826981	2.55499033077154	19.22388853822891
H	2.35215364468258	1.70398620428281	18.59060933158546
C	3.12454788557085	3.70101501367373	18.66637122166868
C	3.48844155487876	5.84392479909603	21.84180772350061
H	3.73490011319396	6.71573847492333	21.23615218889654
C	2.32779508520963	6.23534288169029	22.76024807199308
H	1.43823634365243	6.46903129087625	22.18219091373885
H	2.59181088483081	7.11286987634105	23.35566382893636
H	2.07873767839976	5.43379259244206	23.45926071957397
C	4.71812690183630	5.46720443091403	22.68427424247777
H	5.56533823020816	5.17385809195622	22.06198232218791
H	4.49169806159831	4.62392586511942	23.34132809852220
H	5.02908889768182	6.30676751074891	23.31163199004282
C	3.48676161001644	3.70651452909530	17.19102609740211
H	3.57583912174845	4.74853136188772	16.88148606944472
C	4.84933799746608	3.03157535781986	16.96235717338309
H	5.13647667606713	3.08713512935553	15.90899804999973
H	4.80612729280546	1.97688852376055	17.24557868358734
H	5.63888021446386	3.49686712764680	17.55254463363463
C	2.43469455559080	3.04048821782820	16.29777789578782
H	2.68521869244347	3.19482856451498	15.24642450028056
H	1.43907086512276	3.44702406250414	16.47058953202561
H	2.38683294001373	1.96202791250619	16.46347010608509
C	1.04494712970954	6.37331635312684	17.63382947644386
H	0.57371986793571	7.11346494666154	16.97753194036798
H	1.06306490424177	5.41627418684352	17.12255525525083
C	0.33104344255419	6.34277064569372	18.95303123007411
C	0.59674341624037	7.50121781095067	19.84490820664062
C	1.76636011721928	8.17083354530657	19.70256256998388
C	-0.45158437827265	5.32905044066780	19.36655376112332
H	-0.88157749404311	5.38814956147338	20.36058732082527
C	-0.79639136109755	4.08789643844669	18.60930775931059
H	-0.56786268973190	4.17383134694743	17.54762692967260
H	-1.86331709937212	3.86331320042860	18.70564716606126
H	-0.25381327745772	3.22018171534721	18.99819878403465
C	-0.45142383482563	7.86202413881743	20.86921598140314
H	-1.42985737171483	7.96606383312780	20.39344888099802
H	-0.21523481296754	8.79224953151785	21.38250293322715
H	-0.55920281399844	7.08944724777041	21.63523140346708
C	2.22503390277467	9.30301146165965	20.57809859363782
H	3.00155301680312	9.90011155875705	20.09220990589964
H	2.66098057681108	8.92523120166868	21.50932441085606
H	1.42078760148337	9.99002689133184	20.85929008807452

XYZ Coordinates of (^{iPr}PDA)Ti($C_4Me_2(CH_2)_3$)

Ti	12.83854830361599	10.18961347960770	9.79919751196787
N	13.82538120788852	10.46799238828262	7.86980811591662
N	13.28346224869721	8.37554134381370	9.09843006785152
N	13.60220523748662	12.03794592317890	9.80314181700700
C	13.93494340578273	9.42027413233246	7.05023379954127
C	14.24552688858133	11.68349251196624	7.52025663351954
C	14.57280835620976	6.90792900648448	10.57184839711944
C	12.26466142352591	6.33599587549928	10.00824098239490
C	12.63101376508695	9.66113576123444	11.75678557475521
C	14.53058014397330	12.78422011367794	11.94618823646707
C	13.36647329956335	7.20438549263839	9.89450255287398
C	10.89631455284770	10.45515776606269	9.27982351819221
C	11.42200853507157	13.99588770420756	10.02099558481900
H	11.52667582675551	13.16706446335787	9.32210070757396
C	14.48246428882981	13.65789818220006	13.02821678444104
H	15.22771726581572	13.58629717167478	13.80800542464967
C	14.90155804613893	10.83336989066129	5.39025046659126
H	15.33626403688278	10.97669532092960	4.40885788876117
C	12.52524654922845	14.71952584532278	12.15287214198018
H	11.74697055028630	15.46758867249782	12.24480975026161
C	11.29066761953480	9.85731515205698	11.71953941071549
C	13.54543384626530	12.89534519931272	10.93600428433361
C	14.46630838654468	9.57092779670298	5.77947319715130
H	14.55466065204206	8.71956540309987	5.11774364771536
C	13.32279700246286	9.15826207760381	12.97918251756378
H	13.07805253507141	8.10318463991696	13.14551629444646
H	13.02471019297123	9.70527225247651	13.88083445860302
H	14.40478593894410	9.22300459003031	12.89729116596295
C	14.79874911481885	11.90584290106970	6.26703878101132
H	15.14730832918981	12.89187120053884	5.98989553604383
C	13.49574149473114	14.62702631289688	13.13336265653252
H	13.48165022884084	15.29950494526316	13.98242920630356
C	12.52562016988919	13.86229930536642	11.05417802410155
C	14.05764418606377	12.70134926256013	8.59830009247672
H	14.99562375933236	13.25345162323210	8.75416200899396
H	13.34332520528895	13.45493707203427	8.23435554909450
C	15.77356147794121	7.83448128643884	10.47334387196517
H	15.38547799808078	8.85099918171197	10.38795106056071
C	10.43547883556140	10.24045744766794	10.52440716169812
C	15.62957077860884	11.73667480101753	11.85943675542045
H	15.17066542909623	10.83336982096995	11.45569382344992
C	13.45698686267216	8.14713156013052	7.67656418488162
H	12.51920771162084	7.84695943200349	7.17947008642619
H	14.16585178338161	7.33668442914345	7.46724980341945
C	12.38649634034635	5.17787683090392	10.77307479630340
H	11.54256945363393	4.50396703839590	10.85897850876361
C	10.95408346651540	6.63830495214247	9.30817583985423
H	11.02880253455039	7.65087240384710	8.91275782338875
C	10.03125774299664	13.87033848096315	10.65299124617872
H	9.95355656821668	12.95532544305354	11.23669623661830
H	9.26065084761708	13.84892194572594	9.87831362724100
H	9.81154538466723	14.71141458555912	11.31423127985913
C	10.35064271682465	9.67584150833288	12.90001448209068

H	10.15528726817853	8.60619461857406	13.04257908913805
H	10.79666493221047	10.03569888672928	13.82804599242815
C	11.53373993469335	15.31296499843040	9.23946144074099
H	11.38387655694430	16.17185728842779	9.89815428594576
H	10.77701565165871	15.36155156497029	8.45191684554160
H	12.51549832638676	15.42679392288757	8.77595056247581
C	9.06639608974256	10.41854882689948	12.51959886105422
H	9.15550737869075	11.47067132285452	12.80074904511178
H	8.18352513086292	10.02007906256021	13.02130976299491
C	16.60337207995545	7.56121804532362	9.20936252778153
H	16.98708643781127	6.53769052050226	9.21829257755400
H	17.45716447506662	8.24193349919757	9.15712603022980
H	16.02014551178917	7.69163961675711	8.30037236176652
C	8.98879807336643	10.28991494257691	10.99522262571319
H	8.48917426572019	9.35620251088469	10.71647438436019
H	8.43246536638054	11.10110797678080	10.52487095417522
C	16.69287331271648	7.78871830105338	11.69677068115159
H	16.14044077914095	7.91087338094294	12.62845216822097
H	17.43262664780213	8.58916874901666	11.63216819366483
H	17.24478633009713	6.84760118471152	11.75585479920239
C	9.76639388925946	6.61870446087896	10.27614140557958
H	9.57489150400394	5.61477413403790	10.66191517201137
H	8.85762878597397	6.95609440004476	9.77159660402571
H	9.94754004084997	7.27459527699364	11.12681314096271
C	13.56712420020756	4.87641615507607	11.42902352512131
H	13.64780893422723	3.97412437595278	12.02289085251305
C	14.64616686189889	5.74275292327411	11.32920394651159
H	15.55961997553229	5.50336838557043	11.85679067532363
C	10.07838585672106	10.79252113787286	8.08197806681084
H	10.49091103943930	11.65889648378175	7.55590784744406
H	9.02939819463417	11.00608186953110	8.31244820673885
H	10.09402786034156	9.96647277340486	7.36237318365026
C	10.71161045614177	5.67634407990964	8.13667003189123
H	11.54252933436997	5.69132877759886	7.42893132655860
H	9.79900744248135	5.94293696719227	7.59674540234242
H	10.60226036930836	4.64797065261014	8.49050587841639
C	16.24162137502064	11.37836915544619	13.21629269434982
H	16.84588845860835	12.19597623972498	13.61675033408894
H	16.89998921625036	10.51507788385686	13.10826878579906
H	15.47794086426480	11.13069118009732	13.95423280529602
C	16.74631146714225	12.14907545666421	10.88966247682704
H	16.37653800558789	12.26415980644415	9.87343799995268
H	17.53427974133045	11.39148570550419	10.87256778958044
H	17.19532005059577	13.09595257064909	11.20012328309680

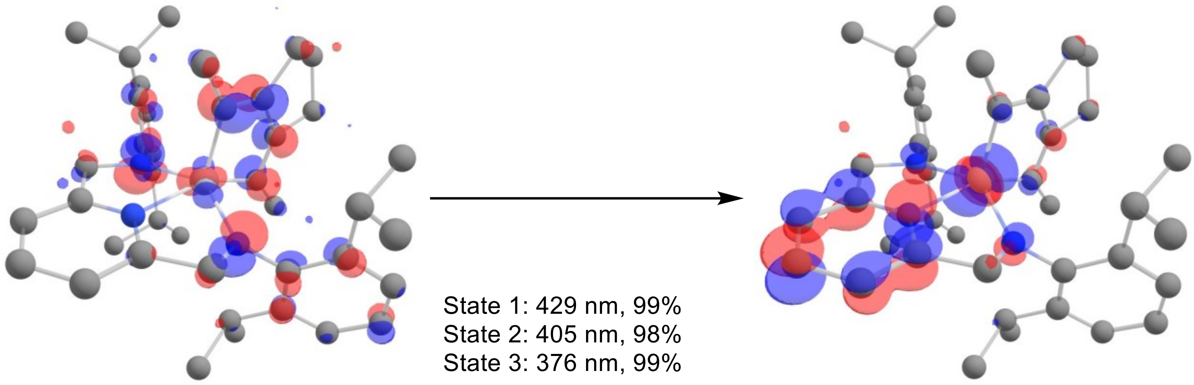

Figure S22. Natural Transition Orbitals of (^{iPr}PDA)Ti($C_4Me_2(CH_2)_3$). Performed with B3LYP level of theory and def2-TZVP basis set for all elements.

In addition to the figure in the main part, the plotted data for the B3LYP functional (Figure S23) is depicted as well as a comparison of the two functionals (Figure S24).

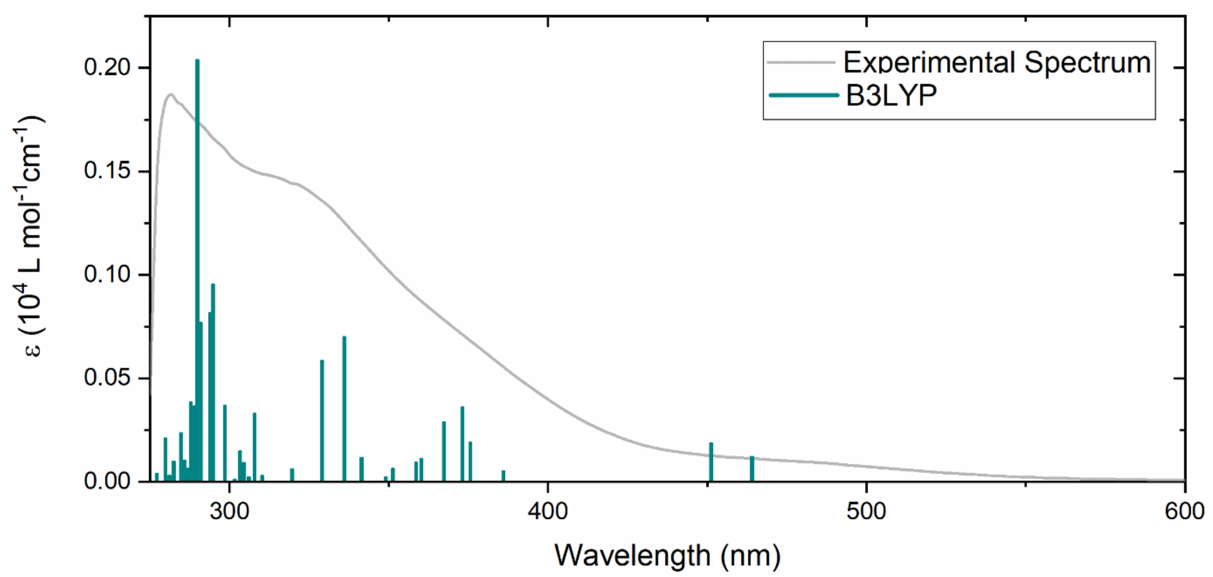

Figure S23. Experimental and computed (B3LYP level of theory) UV-Vis absorption spectra.

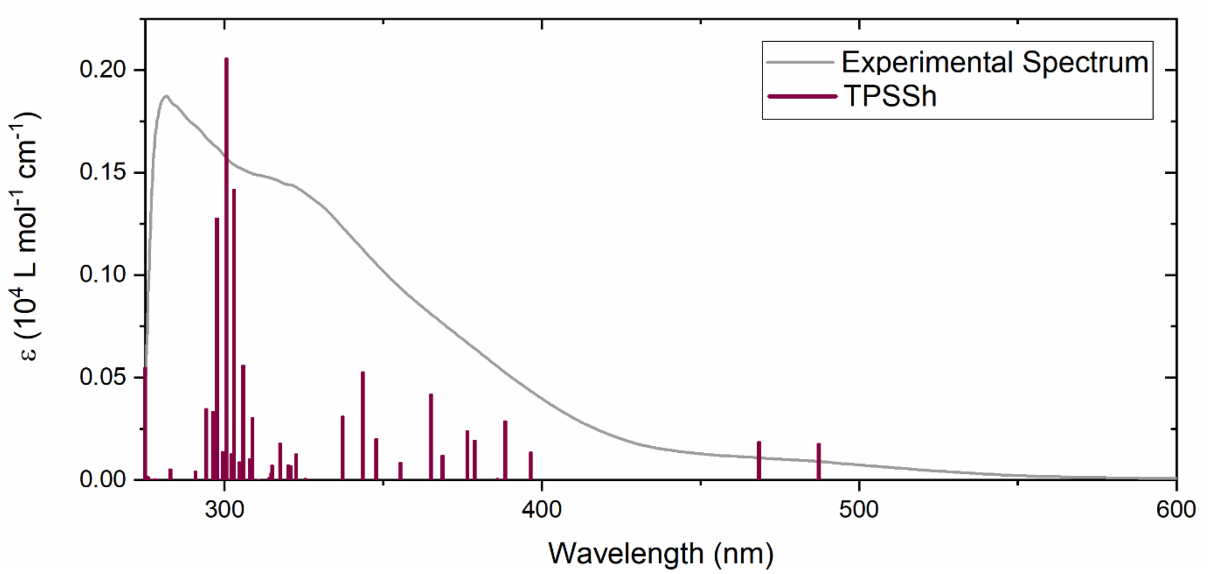

Figure S24. Experimental and computed UV-Vis absorption spectra. Computed spectra with B3LYP and TPSSh functional plotted for comparison.

8. X-ray Crystallographic Details

The crystal data of all compounds was collected on a Rigaku XtaLAB Synergy-R diffractometer with a HPA area detector and multi-layer mirror monochromated Cu-Kα radiation. Multi-scan absorption correction was applied to the data.[17,18] The structures were solved with SHELXT,[19] refined with the SHELXL[20] using full matrix least squares minimization on F2 [21] using using Olex2 as the graphical interface.[22] All non-hydrogen atoms were refined anisotropically. The hydrogen atoms were located in idealized positions and refined isotropically with a riding model.

Crystallographic data have been deposited with the Cambridge Crystallographic Data Center as supplementary publication. These data can be obtained free of charge from The Cambridge Crystallographic Data Centre *via*

www.ccdc.cam.ac.uk/data_request/cif.

Table S9. Crystallographic data and structure refinement for (^{iPr}PDA)Ti($CH_2C_7H_{10}$) and (^{iPr}PDA)Ti($C_4Me_2(CH_2)_3$)

Compound	(^{iPr}PDA)Ti($CH_2C_7H_{10}$)	(^{iPr}PDA)Ti($C_4Me_2(CH_2)_3$)
	2474808	2474809
Empirical formula	$C_{42}H_{60}N_3Ti$	$C_{40}H_{53}N_3Ti$
Formula weight	654.83	623.75
Temperature/K	100.15	100.00(10)
Crystal system	monoclinic	triclinic
Space group	$P2_1/c$	P-1
a/Å	10.1796(2)	10.9780(2)
b/Å	13.3007(2)	12.1187(2)
c/Å	28.0159(5)	14.3274(2)
α/°	90	77.2340(10)
β/°	92.030(2)	76.6100(10)
γ/°	90	70.899(2)
Volume/Å^3	3790.85(12)	1730.21(5)
Z	4	2
ρ_{calc}g/cm^3	1.147	1.197
μ/mm^{-1}	2.135	2.318
F(000)	1420.0	672.0
Crystal size/mm^3	0.15 × 0.09 × 0.04	0.187 × 0.12 × 0.057
Radiation	CuKα (λ = 1.54184)	Cu Kα (λ = 1.54184)
2Θ range for data collection/°	6.314 to 144.196	6.422 to 147.676
Index ranges	-12 ≤ h ≤ 11, -15 ≤ k ≤ 16, -31 ≤ l ≤ 34	-13 ≤ h ≤ 11, -14 ≤ k ≤ 13, -17 ≤ l ≤ 17
Reflections collected	39424	25410
Independent reflections	7367 [R_{int} = 0.0278, R_{sigma} = 0.0193]	6770 [R_{int} = 0.0189, R_{sigma} = 0.0164]
Data/restraints/parameters	7367/0/425	6770/0/407
Goodness-of-fit on F^2	1.056	1.093
Final R indexes [I>=2σ (I)]	R_1 = 0.0367, wR_2 = 0.0950	R_1 = 0.0306, wR_2 = 0.0837
Final R indexes [all data]	R_1 = 0.0392, wR_2 = 0.0964	R_1 = 0.0313, wR_2 = 0.0841
Largest diff. peak/hole / e Å^{-3}	0.64/-0.38	0.30/-0.34

9. Alternative Mechanisms

Alternative Mechanism A: Allene Intermediate

Another plausible mechanistic proposal is the formation of an allene complex (**int-2**) after the visible-light-induced bond homolysis (VLIH). After homolysis of the Ti−C bond, a hydrogen atom is shifted to the titanium forming a titanium hydride complex with an allene moiety (**int-2**). An intramolecular [2+2] cycloaddition is the second (light-induced) step generating the cyclobutene unit binding to the titanium center (**int-3**). Two equivalents of 2-butyne induce the reductive elimination and regenerate **1-C$_4$Me$_4$** for the next catalytic cycle. The intermediate **int-2** could originate from direct *β*-H-abstraction as well omitting the light-induced bond homolysis.

Our experimental data give no evidence for the formation of a hydride intermediate. Furthermore, intramolecular [2+2] cycloadditions without a linker are uncommon in the chemical literature.

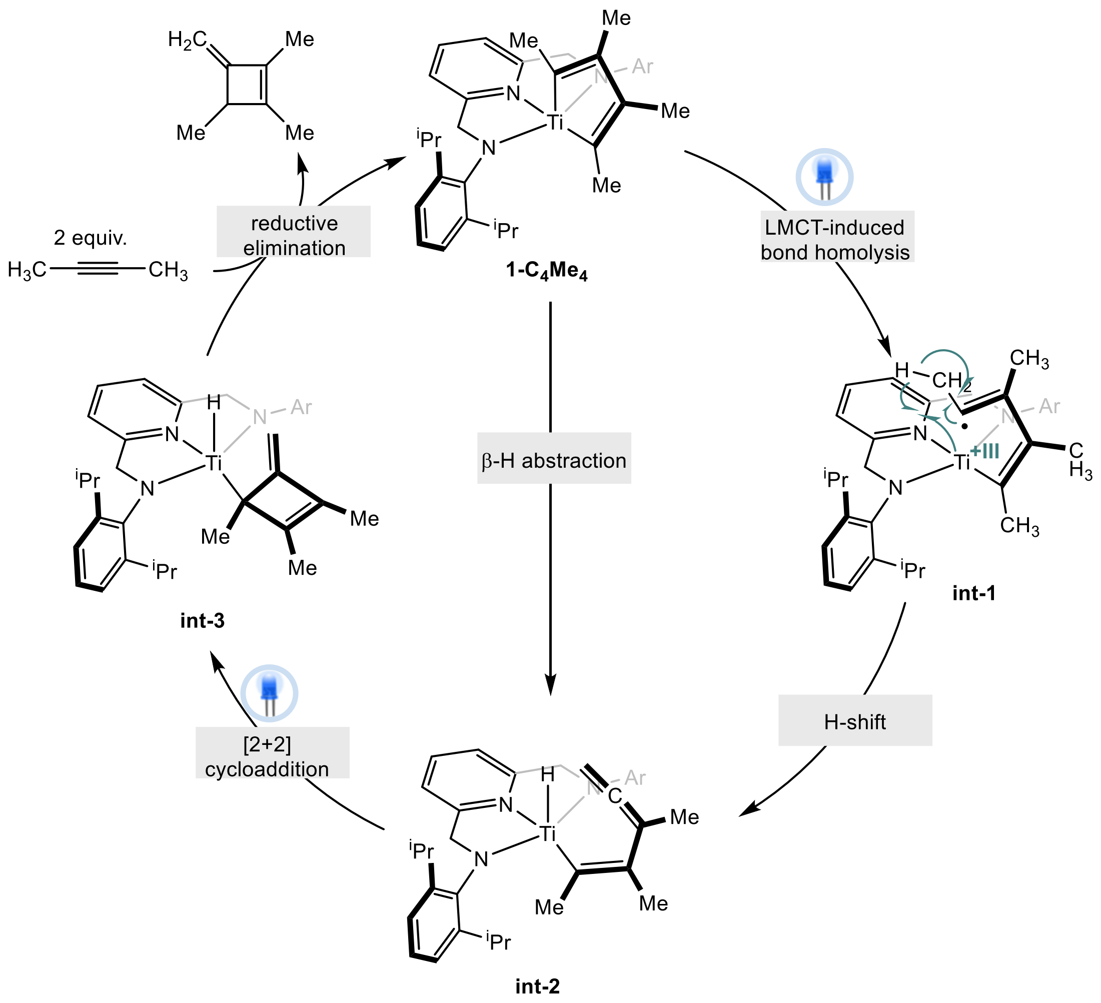

Scheme S1. Alternative Mechanism A: Allene Intermediate.

Alternative Mechanism B: Light-Induced Hydrogen Shift

An LMCT-induced hydrogen shift was another scenario that was considered. This mechanism is not in alliance with the VLIH and our stoichiometric experiments. Instead of the Ti−C bond a C-H bond is broken generating the intermediate **int-1**. **Int-1** then releases the product upon reductive elimination and stepwise coordination of two equivalents of alkyne regenerate the titanacyclopentadiene.

Again, this mechanism is not in line with the formation of (^{iPr}PDA)Ti($CH_2C_7H_{10}$), the rearranged metallacycle, and therefore considered unlikely.

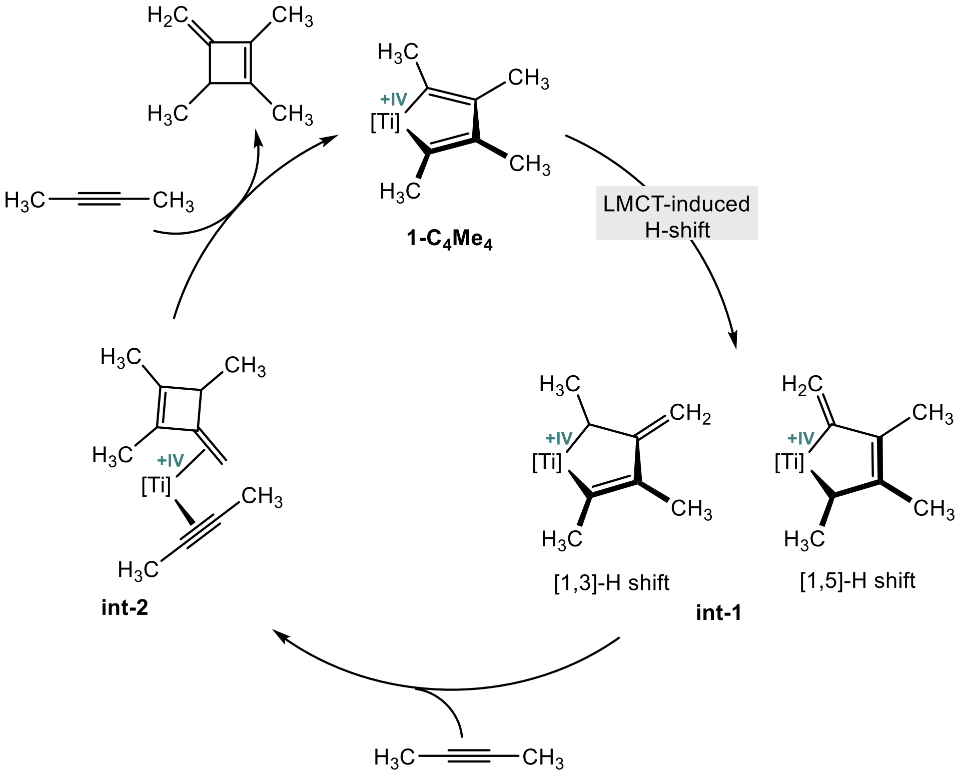

Scheme S2. Alternative Mechanism B: Light-Induced Hydrogen Shift.

10. NMR and GC-MS Spectra of Isolated Compounds

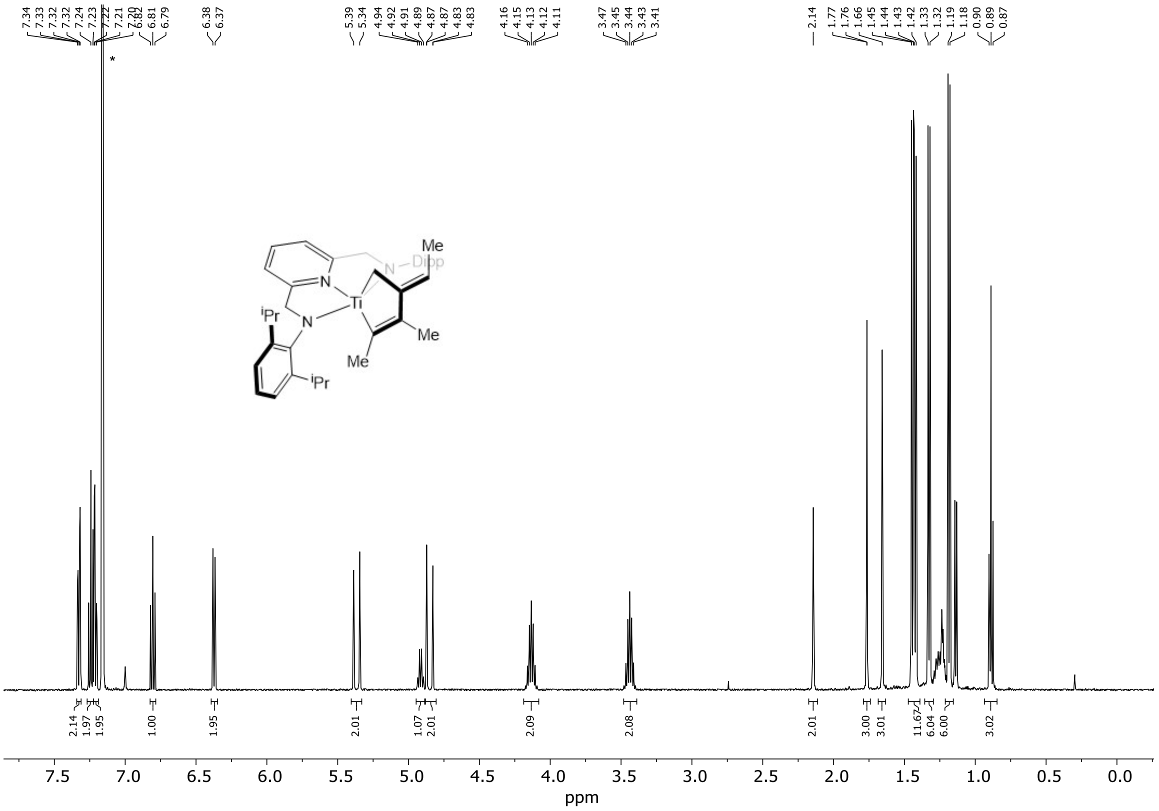

Figure S25. ^{1}H NMR spectrum (400 MHz, 298 K, benzene-*d*$_6$) of (^{iPr}PDA)Ti($CH_2C_7H_{10}$); benzene-*d*$_6$ (*).

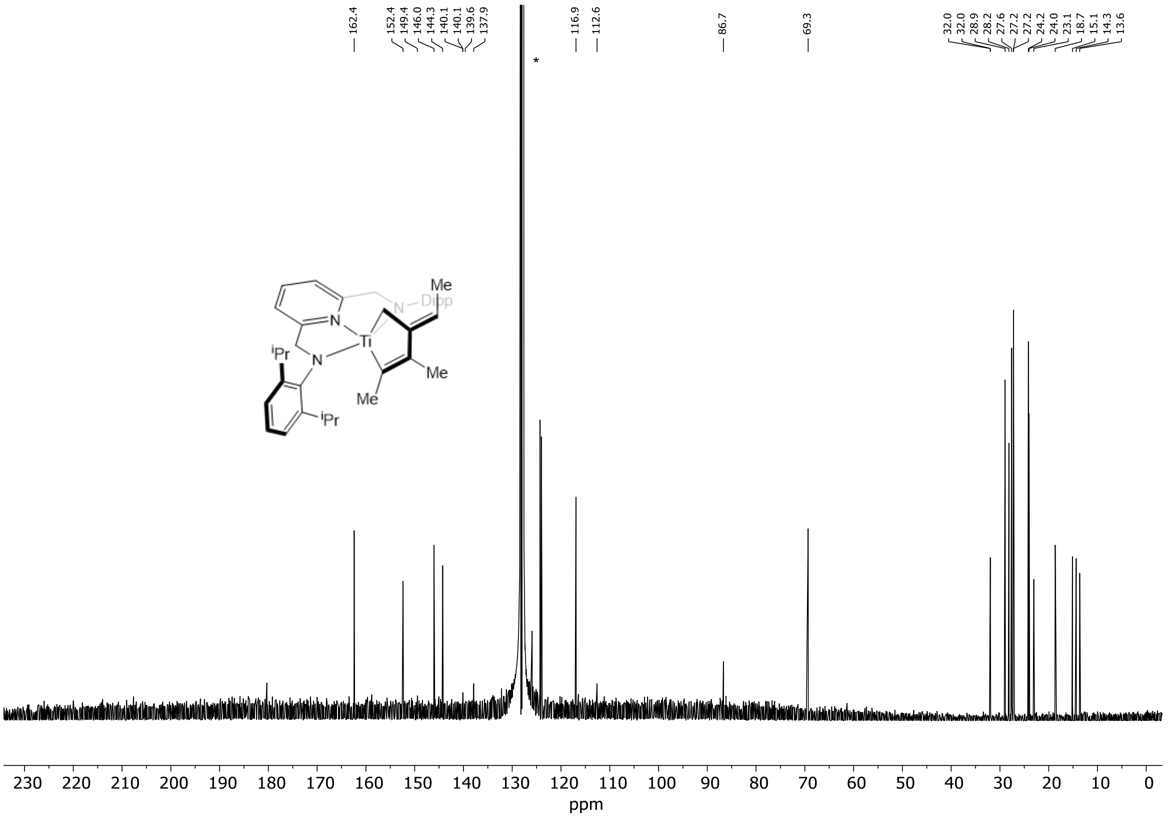

Figure S26. $^{13}C\{^{1}H\}$ NMR spectrum (101 MHz, 298 K, benzene-*d*$_6$) of (^{iPr}PDA)Ti($CH_2C_7H_{10}$); benzene-*d*$_6$ (*).

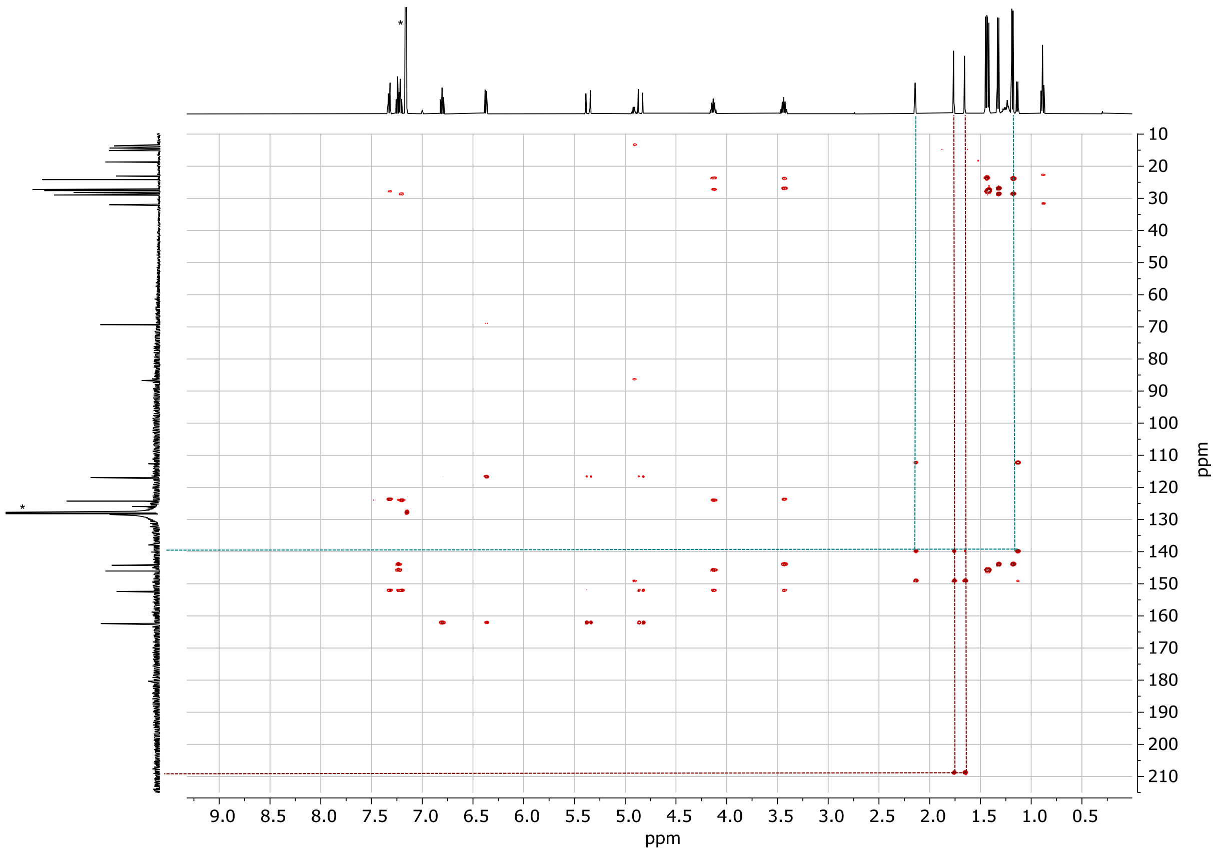

Figure S27. ^1H-^{13}C HMBC spectrum (400 MHz, 101 MHz, 298 K, benzene-*d*$_6$) of (^{iPr}PDA)Ti($CH_2C_7H_{10}$) showing the resonances with the quaternary carbon atoms at δ = 209.1 ppm and δ = 140.1 ppm; benzene-*d*$_6$ (*).

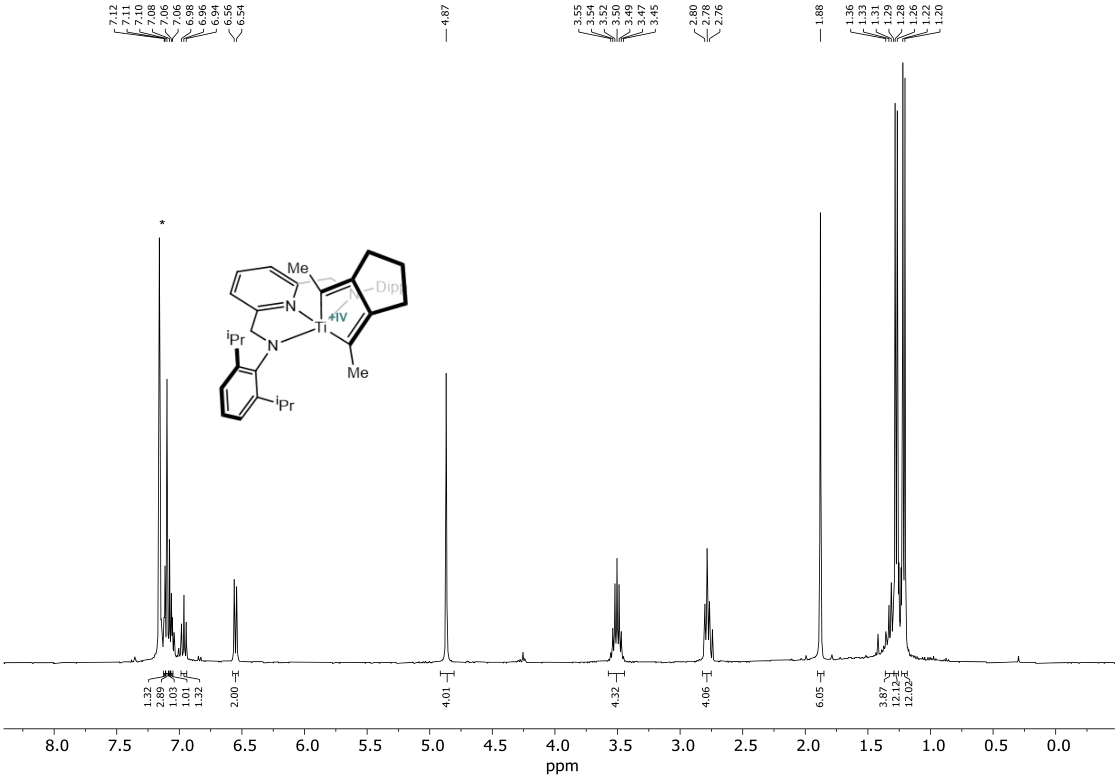

Figure S28. ^1H NMR spectrum (400 MHz, 298 K, benzene-*d*$_6$) of (^{iPr}PDA)Ti($C_4Me_2(CH_2)_3$); benzene-*d*$_6$ (*).

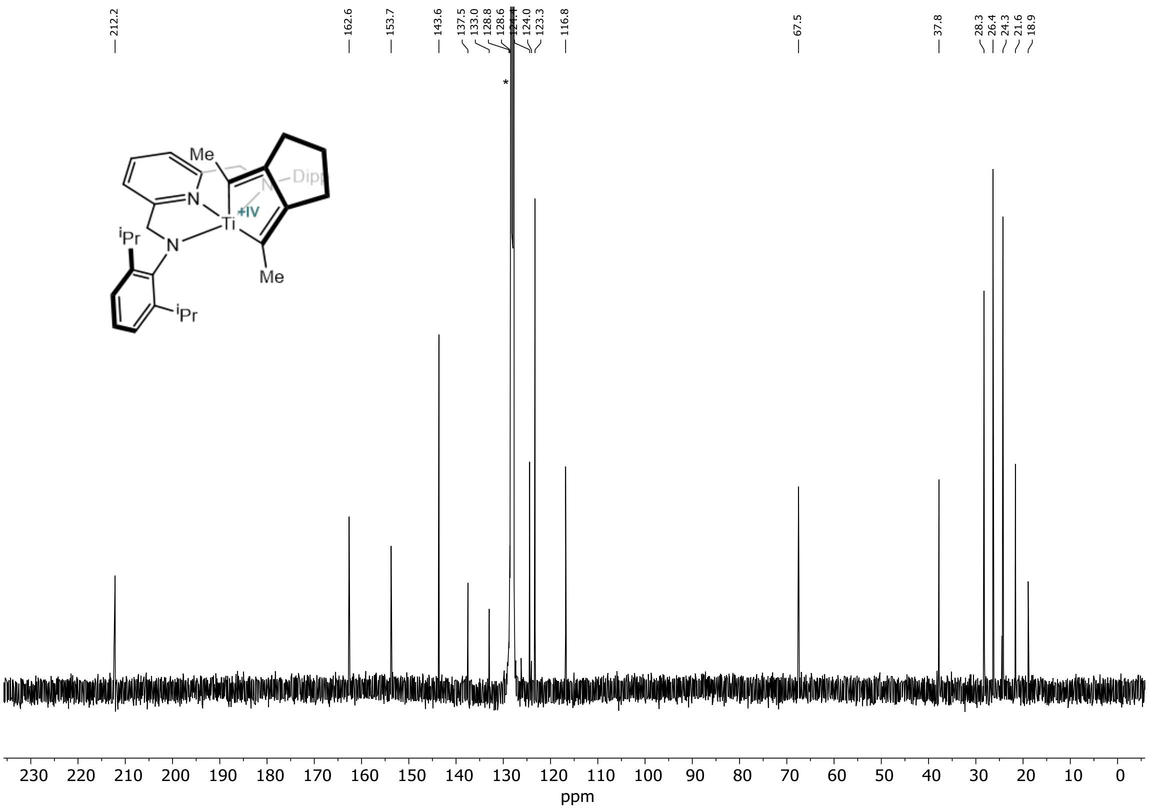

Figure S29. $^{13}C\{^{1}H\}$ NMR spectrum (101 MHz, 298 K, benzene-d_6) of (^{iPr}PDA)Ti($C_4Me_2(CH_2)_3$); benzene-d_6 (*).

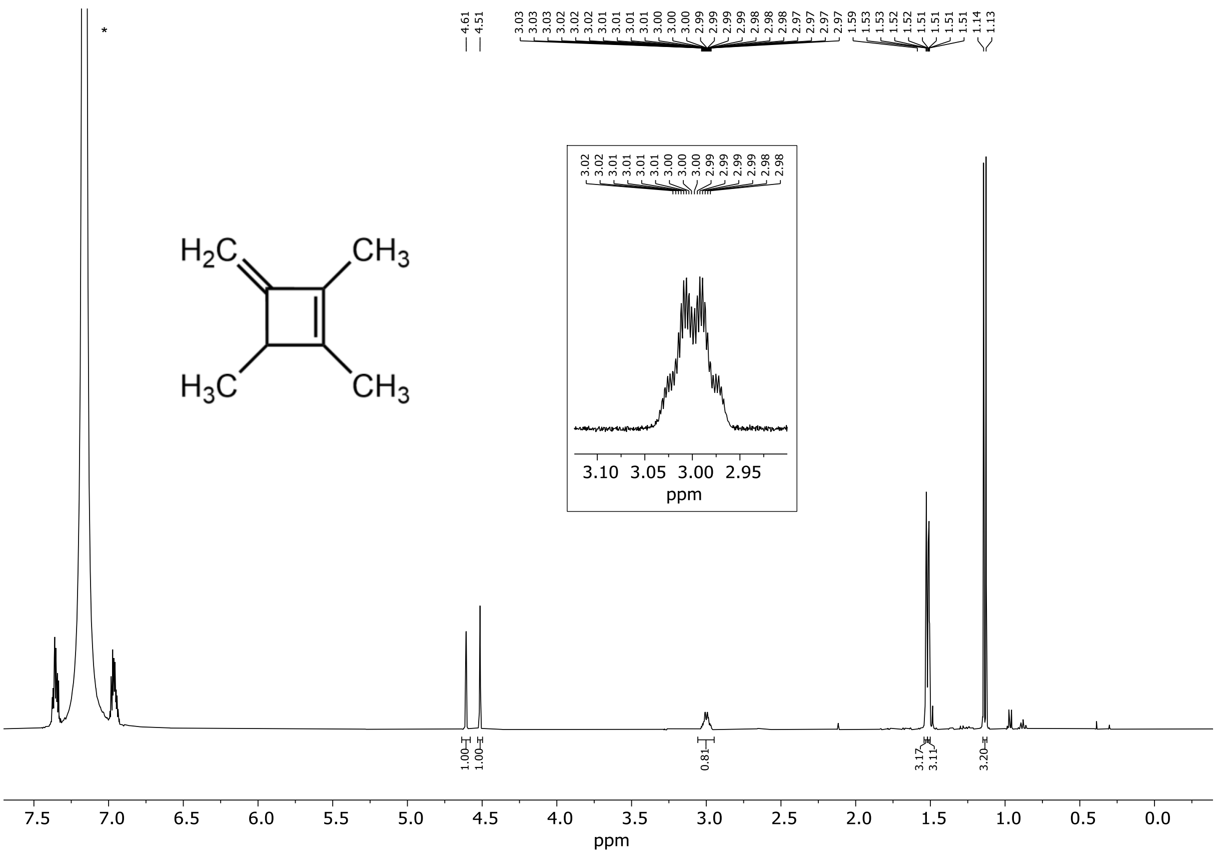

Figure S30. ^{1}H NMR spectrum (400 MHz, 298 K, benzene-d_6) of neat 1,2,3-trimethyl-4-methylenecyclobutene isolated from toluene; benzene-d_6 (*).

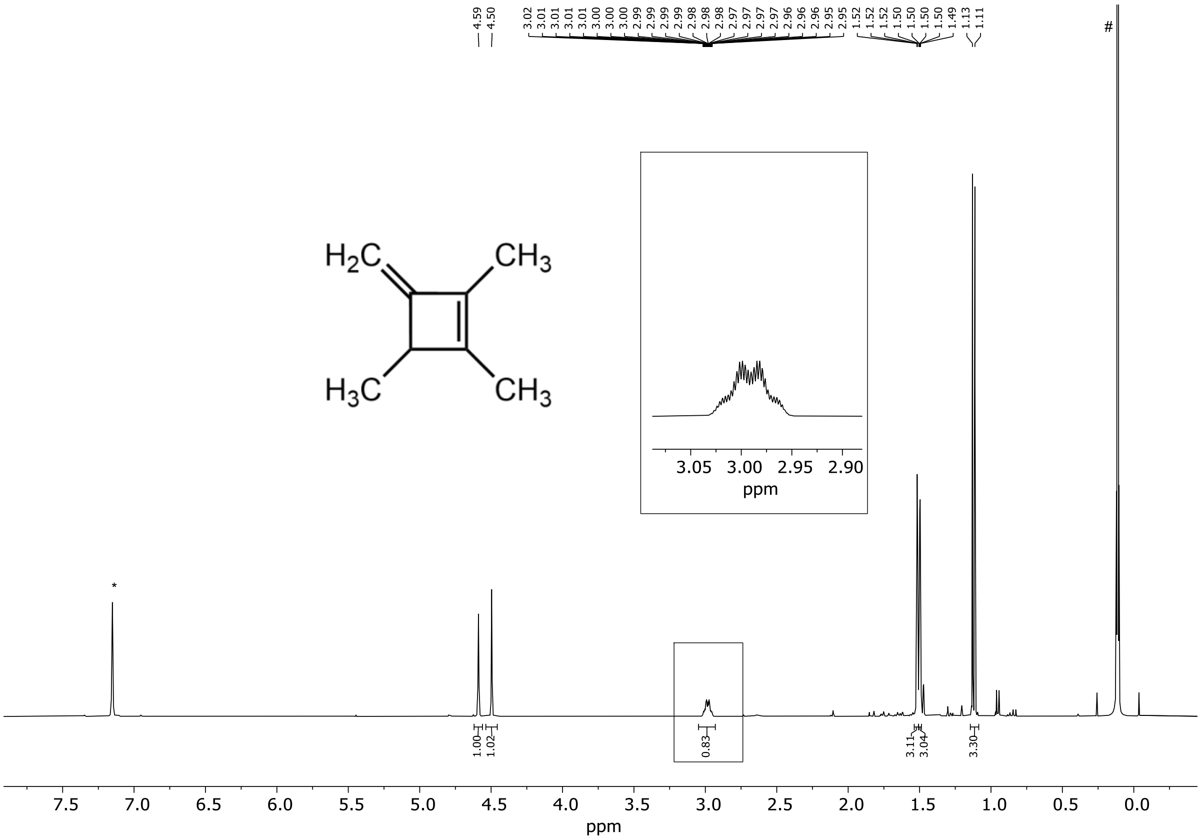

Figure S31. ^{1}H NMR spectrum (400 MHz, 298 K, benzene-d_6) of 1,2,3-trimethyl-4-methylenecyclobutene; benzene-d_6 (*), HMDSO (#).

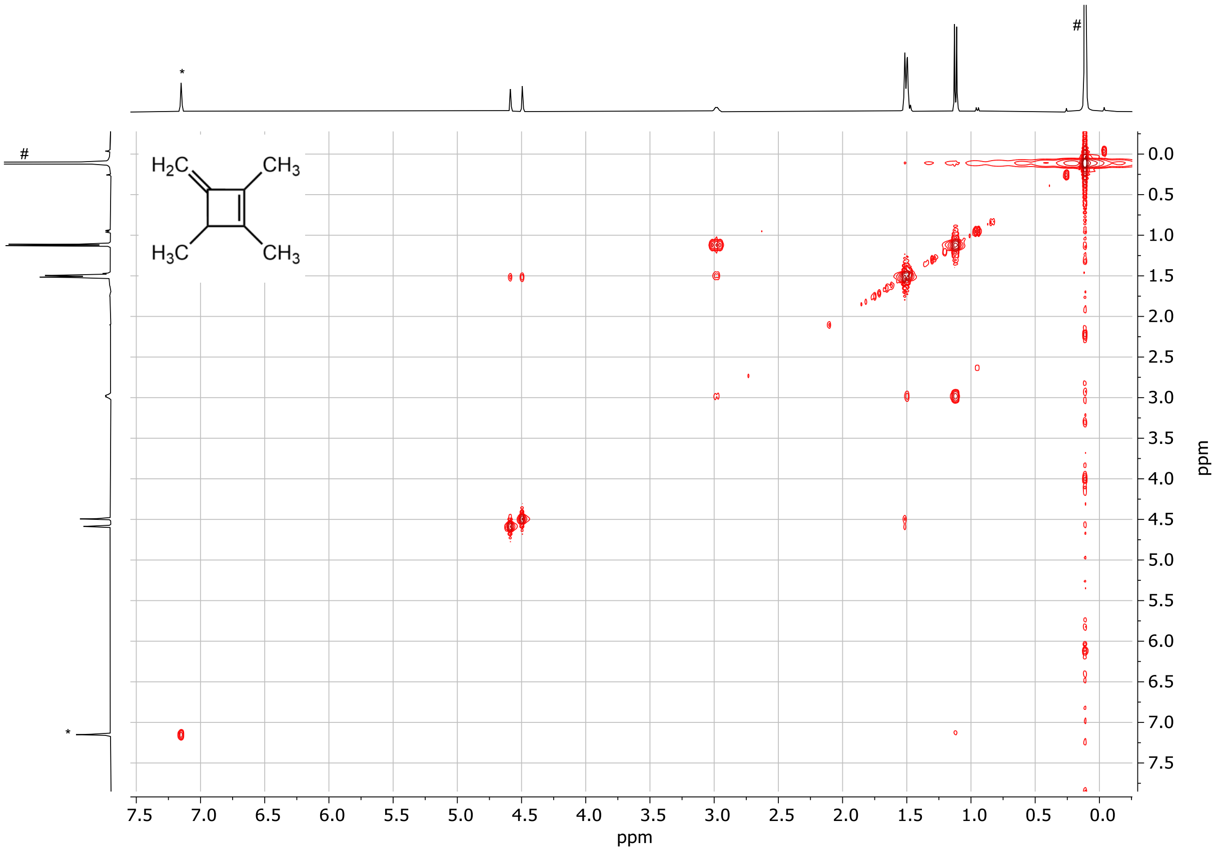

Figure S32. ^{1}H-^{1}H COSY NMR spectrum (400 MHz, 298 K, benzene-d_6) of 1,2,3-trimethyl-4-methylenecyclobutene, benzene-d_6 (*), HMDSO (#).

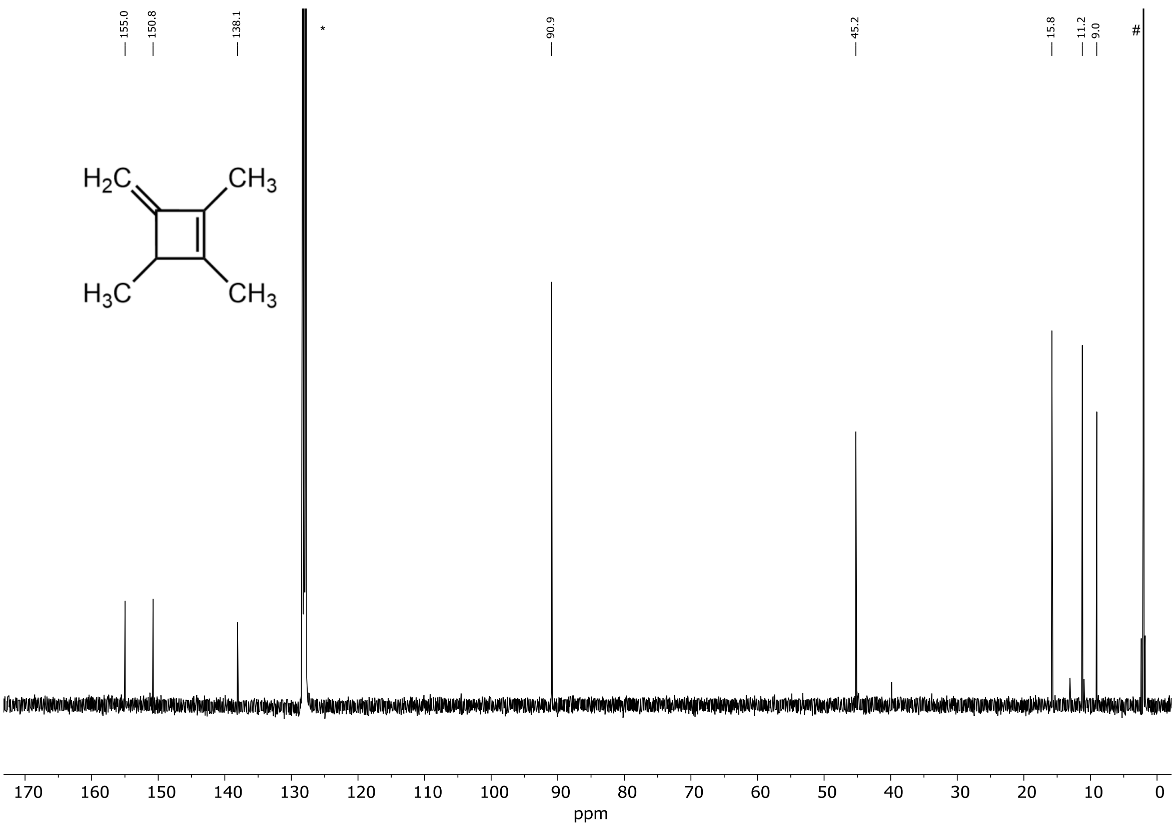

Figure S33. $^{13}C\{^{1}H\}$ NMR spectrum (101 MHz, 298 K, benzene-d_6) of 1,2,3-trimethyl-4-methylenecyclobutene; benzene-d_6 (*), HMDSO (#).

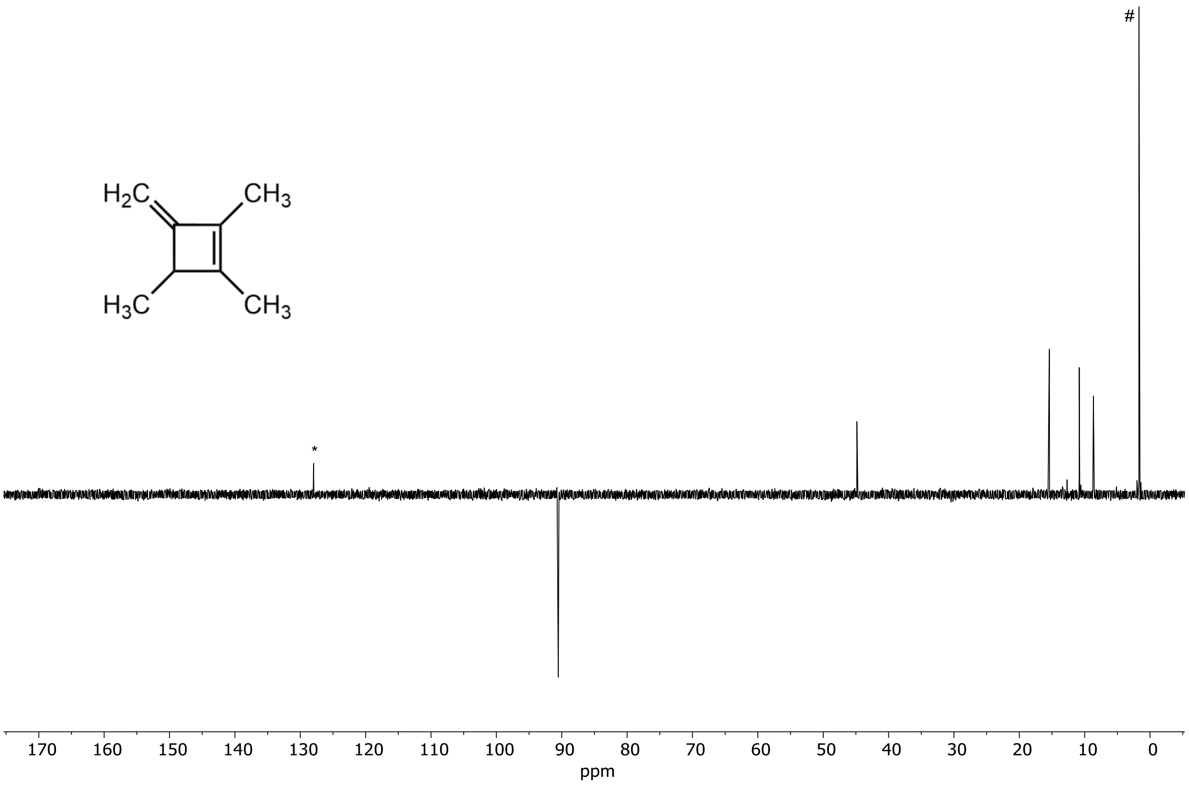

Figure S34. DEPT135 NMR spectrum (101 MHz, 298 K, benzene-d_6) of 1,2,3-trimethyl-4-methylenecyclobutene; benzene-d_6 (*), HMDSO (#).

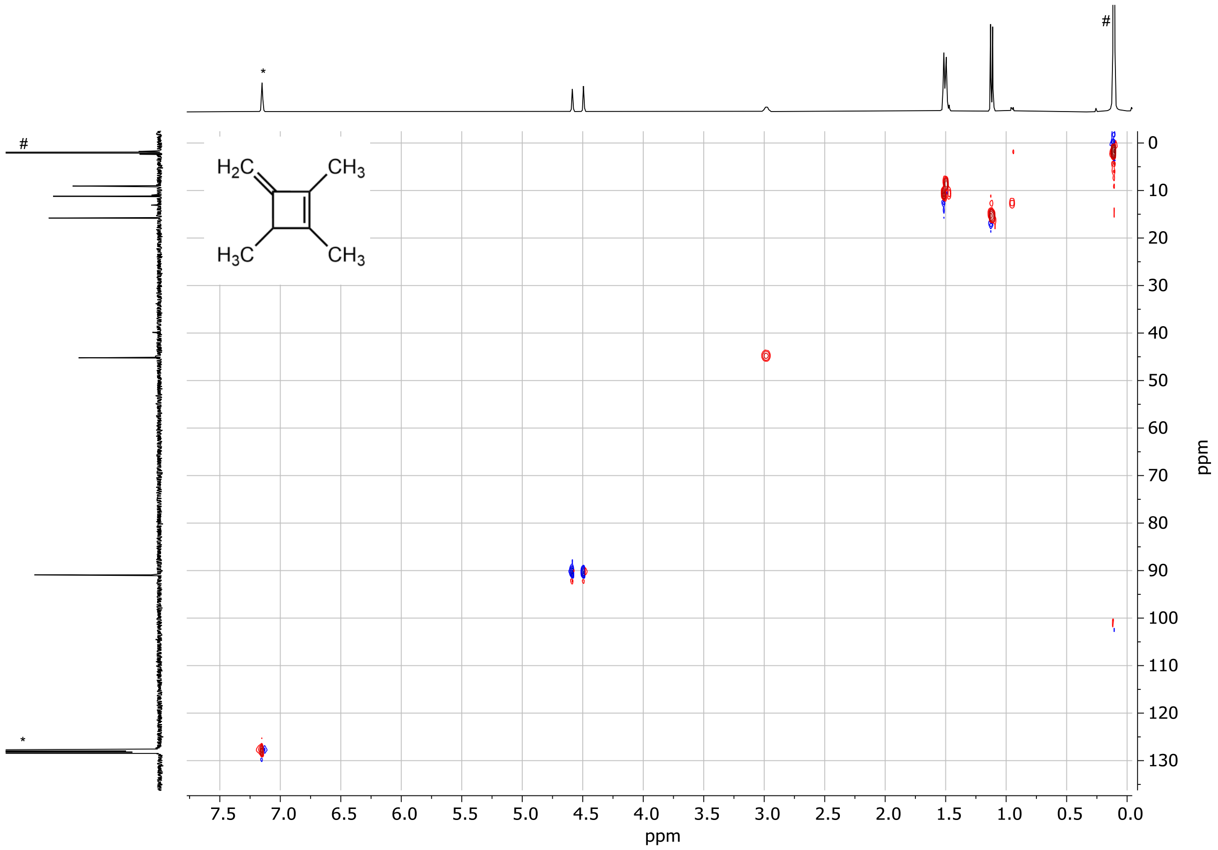

Figure S35. ^1H-^{13}C HSQC NMR spectrum of (400 MHz, 101 MHz, 298 K, benzene-d_6) 1,2,3-trimethyl-4-methylenecyclobutene; benzene-d_6 (*), HMDSO (#).

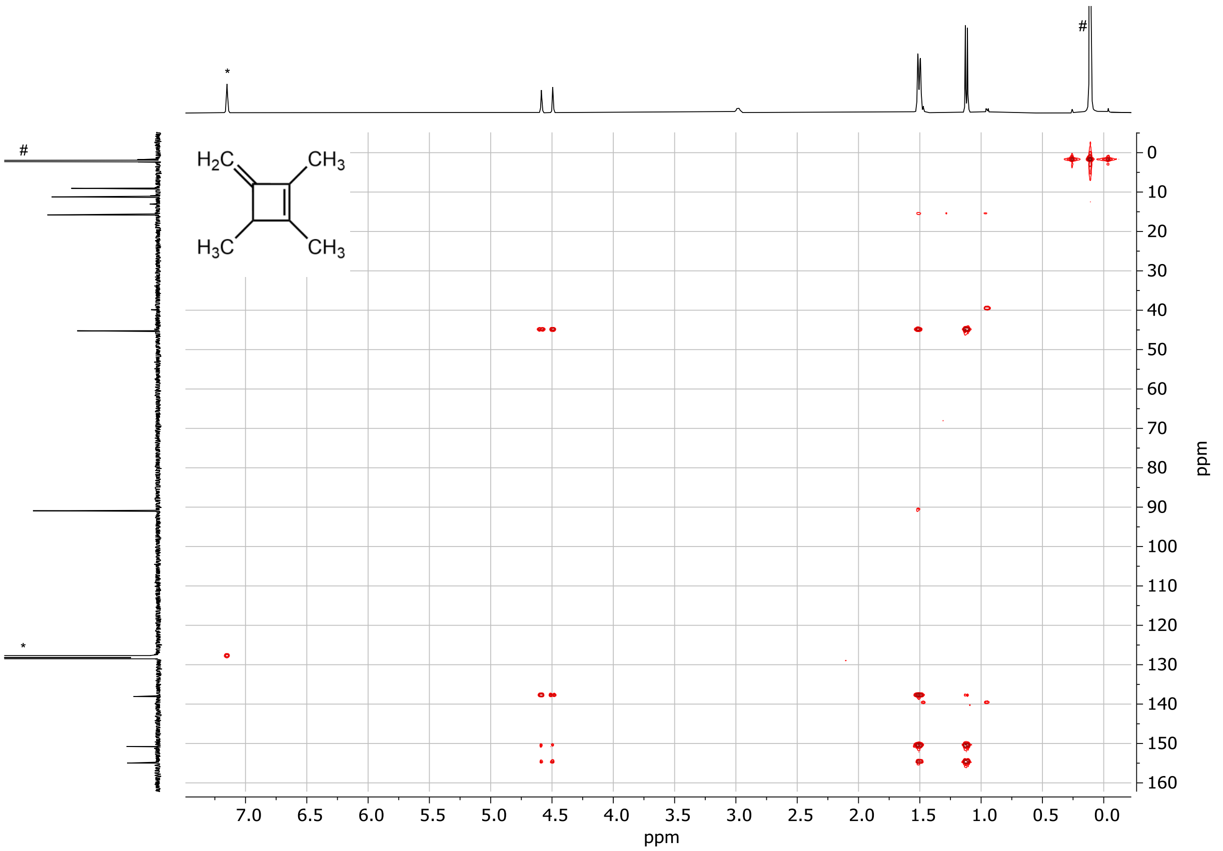

Figure S36. ^{1}H-^{13}C HMBC spectrum of (400 MHz, 101 MHz, 298 K, benzene-d_6) 1,2,3-trimethyl-4-methylenecyclobutene; benzene-d_6 (*), HMDSO (#).

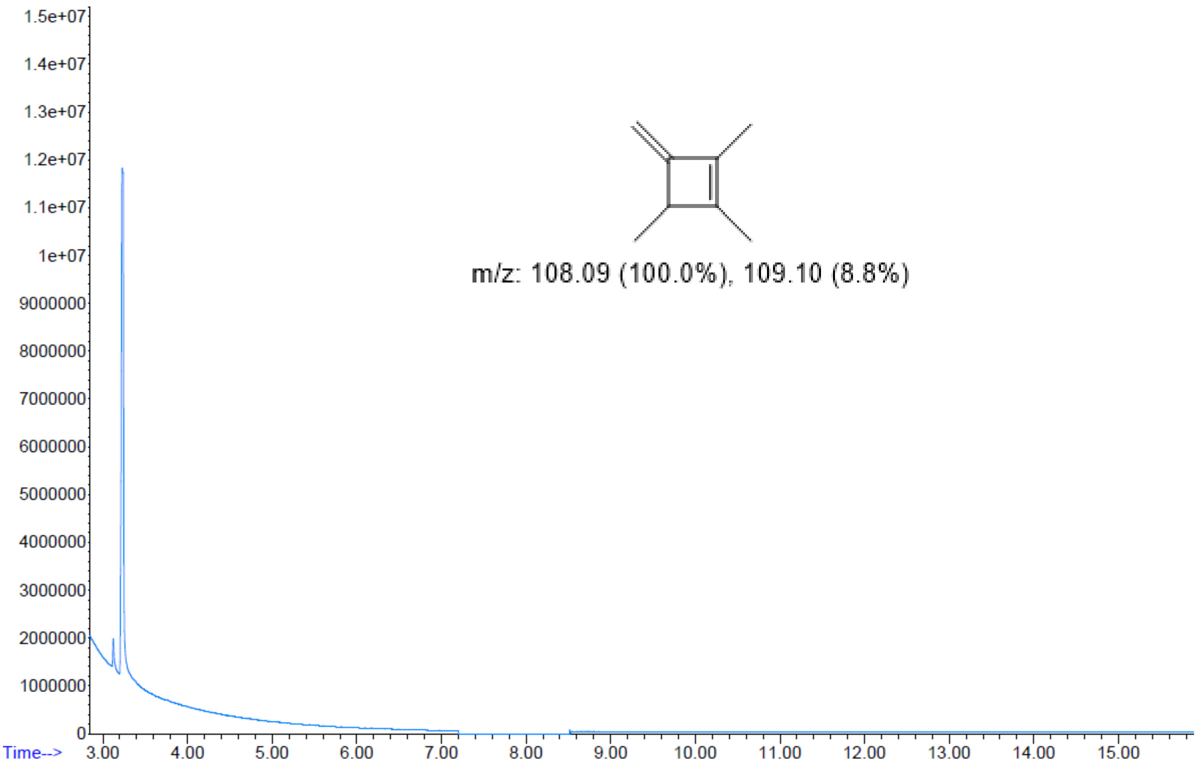

Figure S37. GC-MS chromatogram of 1,2,3-trimethyl-4-methylenecyclobutene.

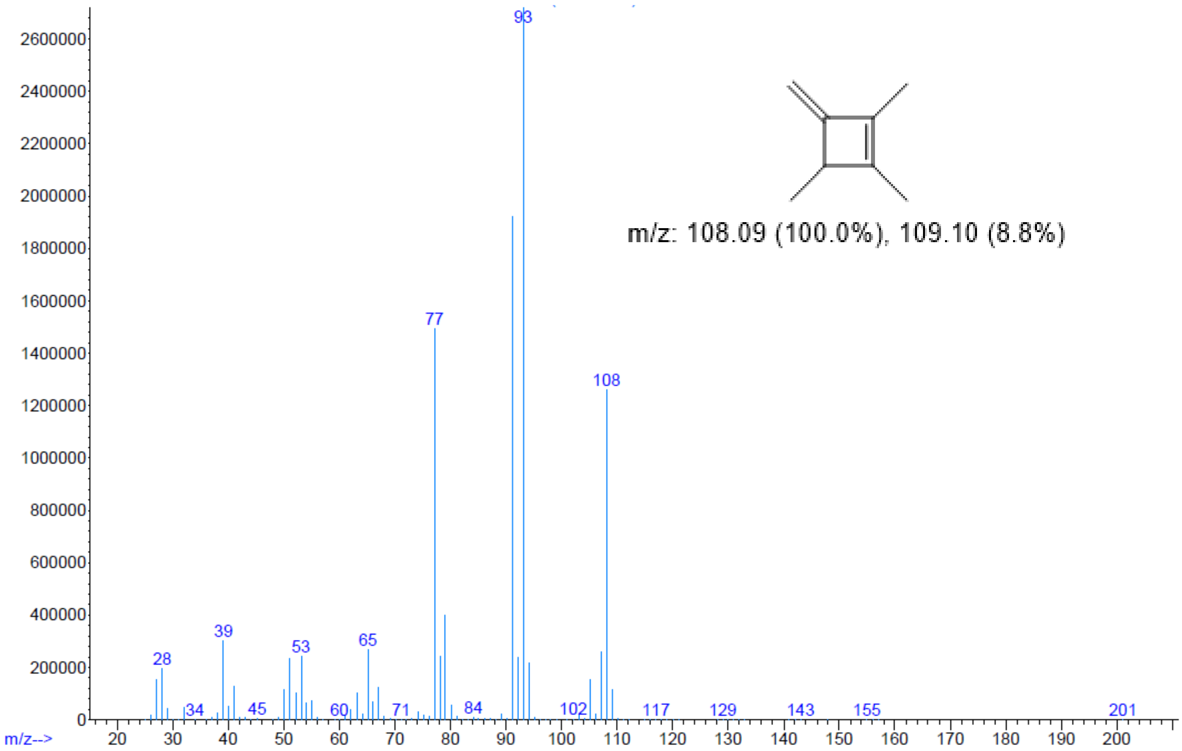

Figure S38. GC-MS spectrum (3.230 min) of 1,2,3-trimethyl-4-methylenecyclobutene.

11. Mechanistic Experiments and Corresponding NMR spectra

Reaction Condition Screening

Table S10. Reaction Conditions.

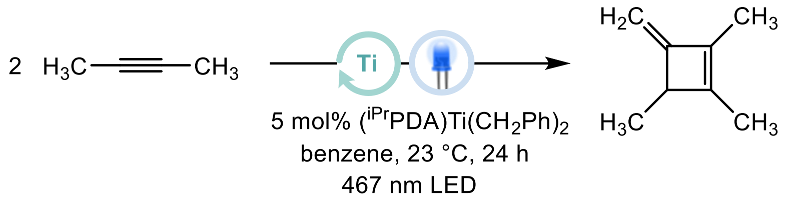

Entry	Deviation from standard conditions	Conv.[b]	Yield[b]	Figure
1	None	100%	>95%	Figure S39
2	No (^{iPr}PDA)Ti(CH_2Ph)$_2$	0%	0%	Figure S40
3	^{iPr}PDAH_2 instead of (^{iPr}PDA)Ti(CH_2Ph)$_2$	0%	0%	Figure S41
4	No Light, rt	0%	0%	Figure S42
5	No Light, 60 °C	0%	0%	Figure S43
6	(^{Me}PDA)Ti(CH_2Ph)$_2$ instead of (^{iPr}PDA)Ti(CH_2Ph)$_2$	57%	9%	Figure S44
7	[Cp_2Ti(η^2-Me_3SiCCSiMe_3)] instead of (^{iPr}PDA)Ti(CH_2Ph)$_2$	90%	0%	Figure S45
8	Ti(CH_2Ph)$_4$ instead of (^{iPr}PDA)Ti(CH_2Ph)$_2$	89%	0%	Figure S46
9	(^{iPr}PDA)Ti(C_4Me_4) instead of (^{iPr}PDA)Ti(CH_2Ph)$_2$	95%	85%	Figure S47
10	(^{iPr}PDA)Ti($CH_2C_7H_{10}$) instead of (^{iPr}PDA)Ti(CH_2Ph)$_2$	0%	0%	Figure S48
11	LED intensity 0.25 instead of 0.5	85%	78%	Figure S49

[a] standard conditions: 0.37 mmol 2-butyne, [Ti] = 0.05 mol/L [b] conversion and yield were determined by ^{1}H NMR spectroscopy using HMDSO as internal standard.

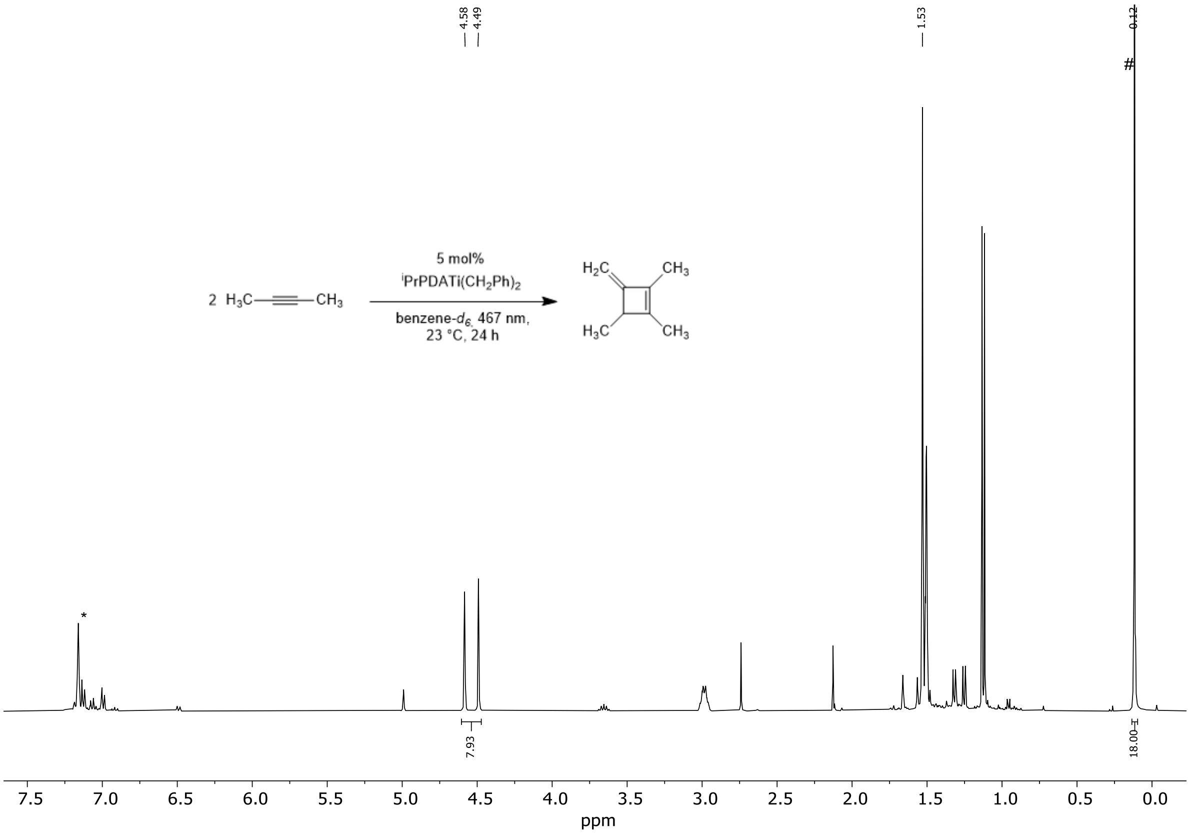

Figure S39. ^{1}H NMR spectrum (400 MHz, 298 K, benzene-d_6) after 24 h irradiation at 467 nm (Standard Conditions, Table 1, Entry 1); benzene-d_6 (*), HMDSO (#).

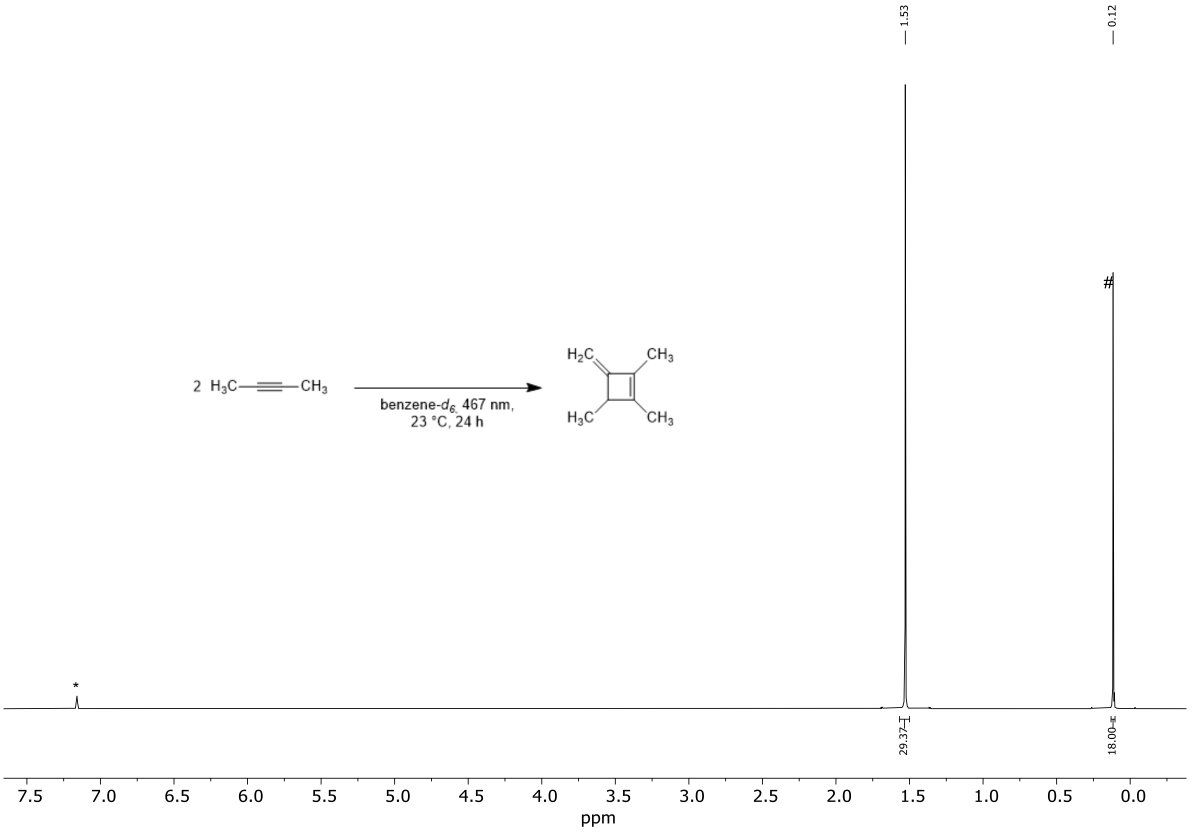

Figure S40. ^{1}H NMR spectrum (400 MHz, 298 K, benzene-d_6) after 24 h irradiation at 467 nm without (^{iPr}PDA)Ti(CH_2Ph)$_2$ (Table 1, Entry 2); benzene-d_6 (*), HMDSO (#).

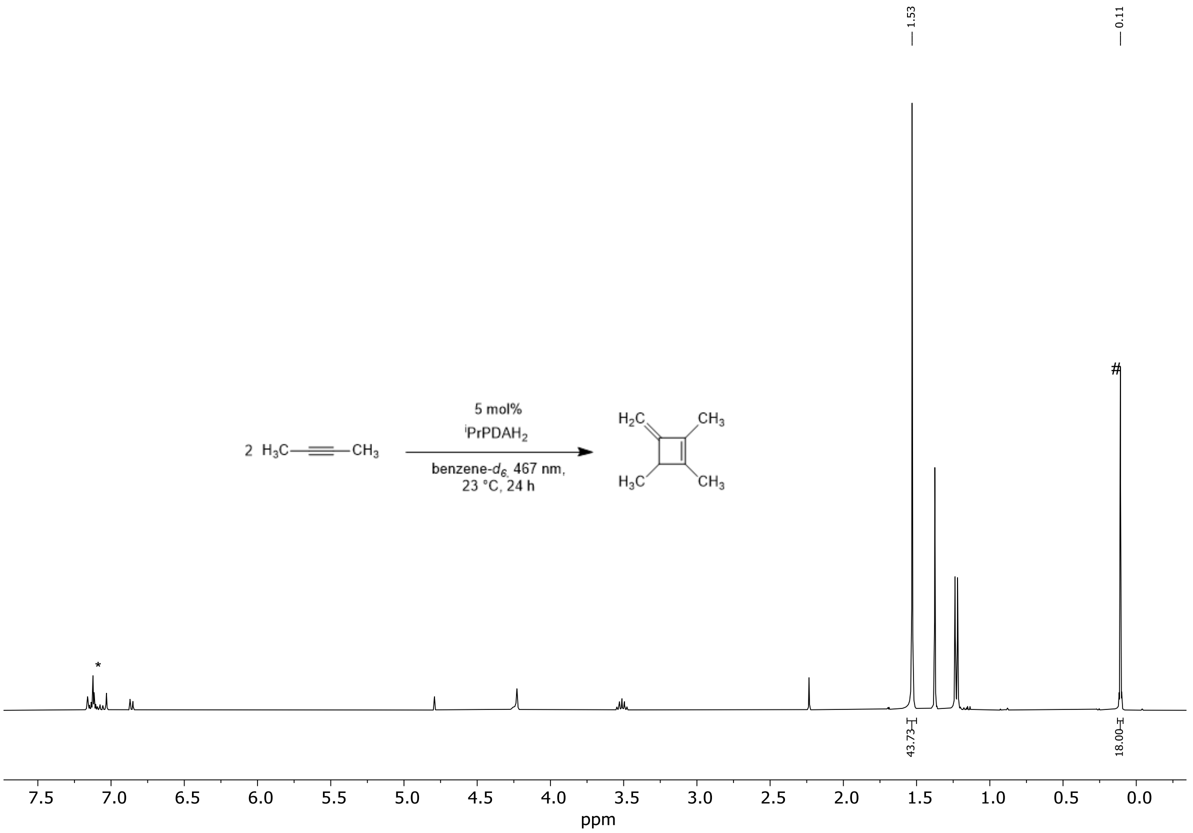

Figure S41. ^{1}H NMR spectrum (400 MHz, 298 K, benzene-d_6) after 24 h irradiation at 467 nm with $^{iPr}PDA-H_2$ instead of ($^{iPr}PDA)Ti(CH_2Ph)_2$ (Table 1, Entry 3); benzene-d_6 (*), HMDSO (#).

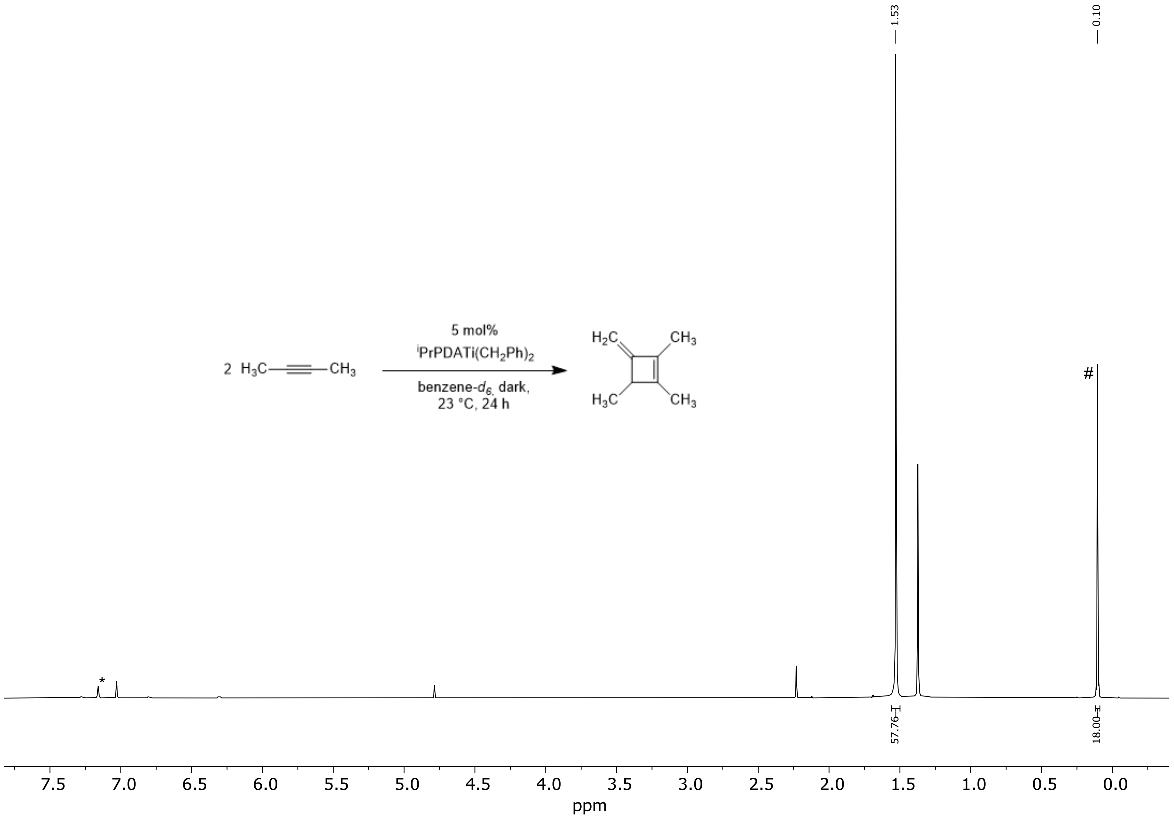

Figure S42. ^{1}H NMR spectrum (400 MHz, 298 K, benzene-d_6) after 24 h without irradiation (Table 1, Entry 4); benzene-d_6 (*), HMDSO (#).

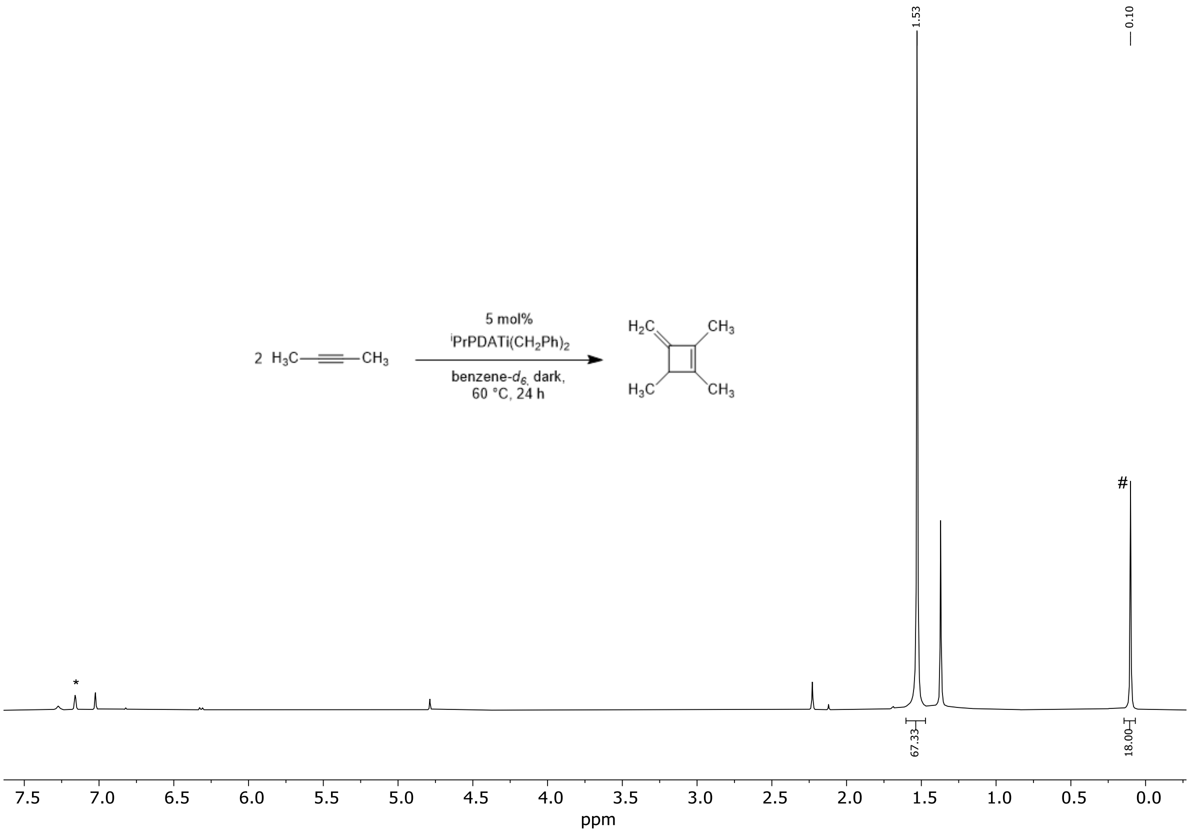

Figure S43. ^{1}H NMR spectrum (400 MHz, 298 K, benzene-d_6) after 24 h without irradiation at 60 °C (Table 1, Entry 5); benzene-d_6 (*), HMDSO (#).

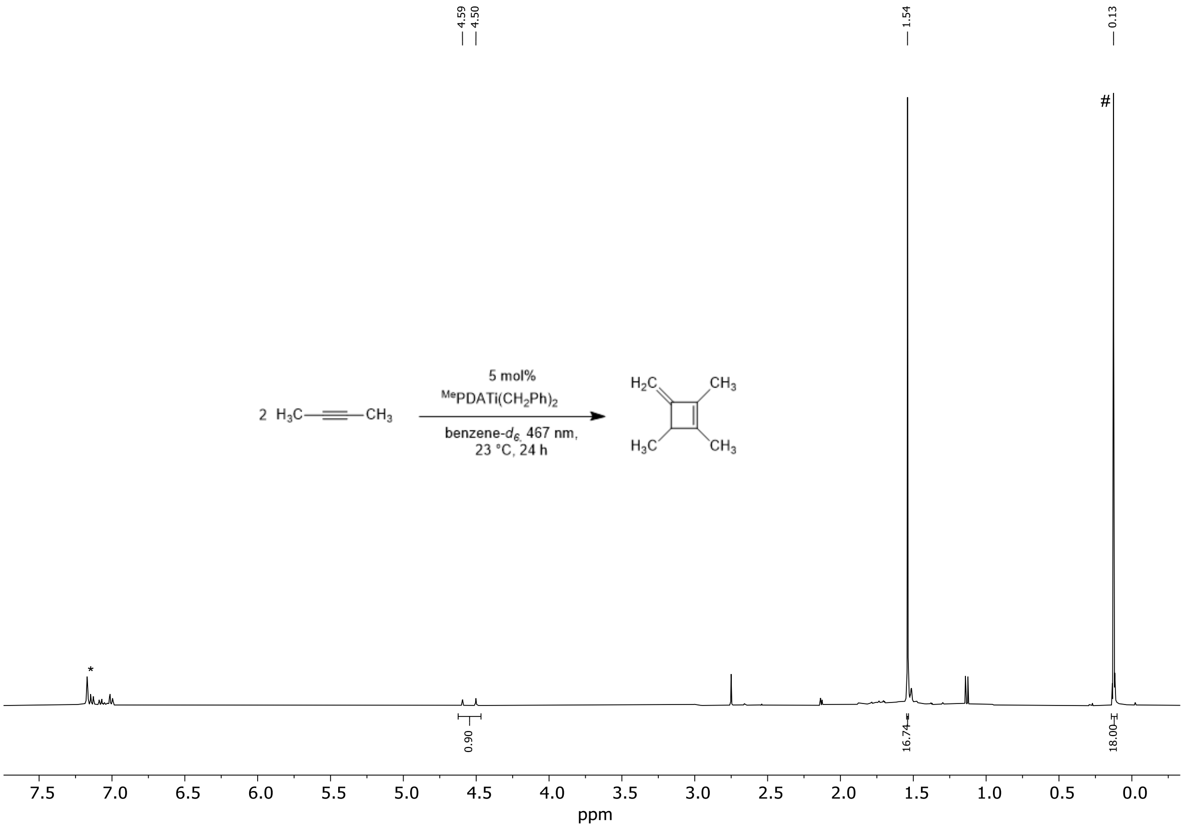

Figure S44. ^{1}H NMR spectrum (400 MHz, 298 K, benzene-*d*$_6$) after 24 h irradiation at 467 nm with (^{Me}PDA)Ti(CH$_2$Ph)$_2$ instead of (^{iPr}PDA)Ti(CH$_2$Ph)$_2$ (Table 1, Entry 6); benzene-*d*$_6$ (*), HMDSO (#).

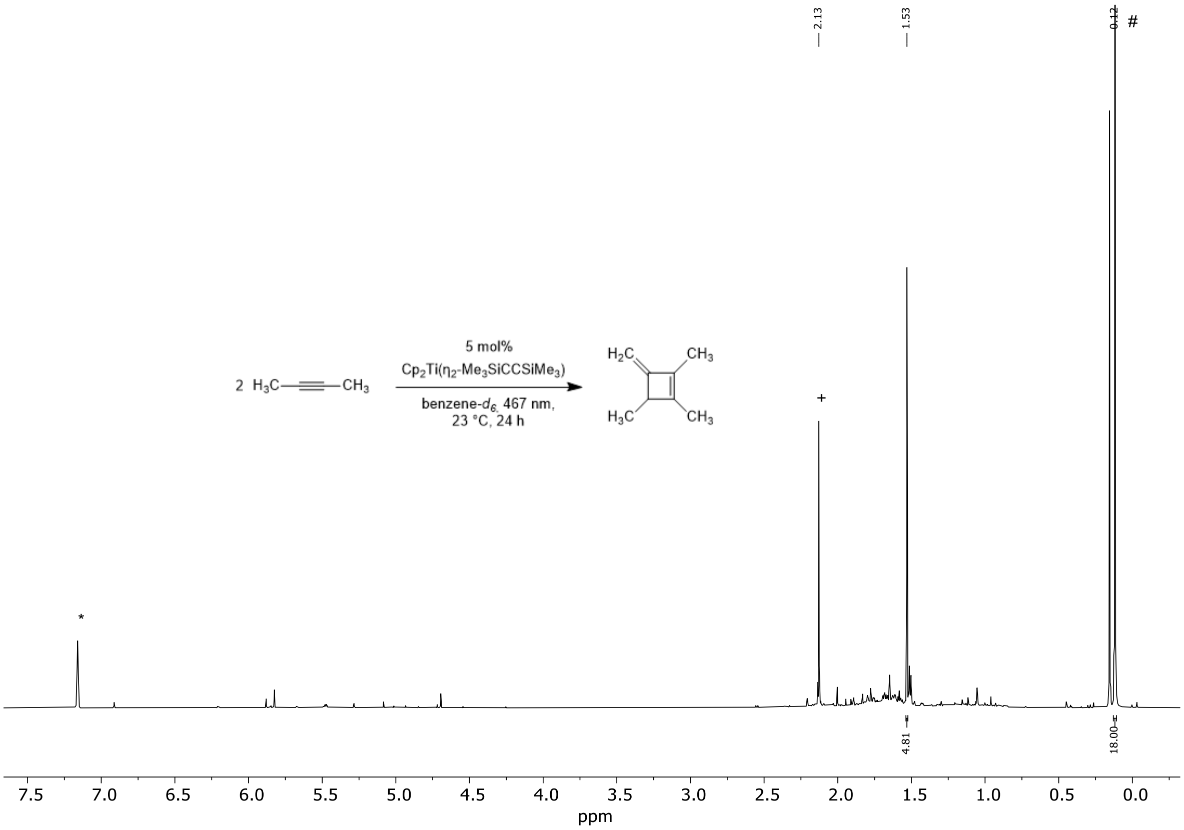

Figure S45. ^{1}H NMR spectrum (400 MHz, 298 K, benzene-d_6) after 24 h irradiation at 467 nm with [$Cp_2Ti(\eta^2\text{-}Me_3SiCCSiMe_3)$] instead of (^{iPr}PDA)$Ti(CH_2Ph)_2$ (Table 1, Entry 7); benzene-d_6 (*), HMDSO (#), Hexamethylbenzene (+).

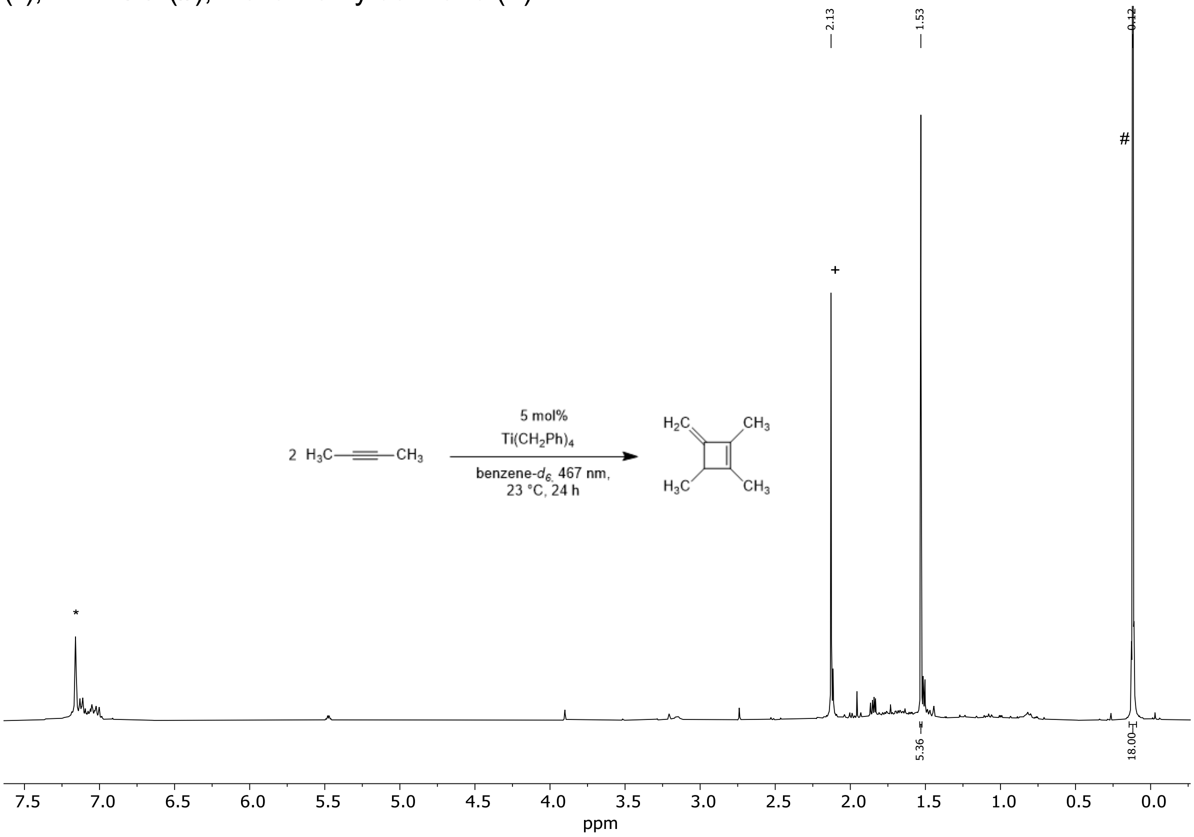

Figure S46. ^{1}H NMR spectrum (400 MHz, 298 K, benzene-d_6) after 24 h irradiation at 467 nm with $Ti(CH_2Ph)_4$ instead of (^{iPr}PDA)$Ti(CH_2Ph)_2$ (Table 1, Entry 8); benzene-d_6 (*), HMDSO (#), Hexamethylbenzene (+).

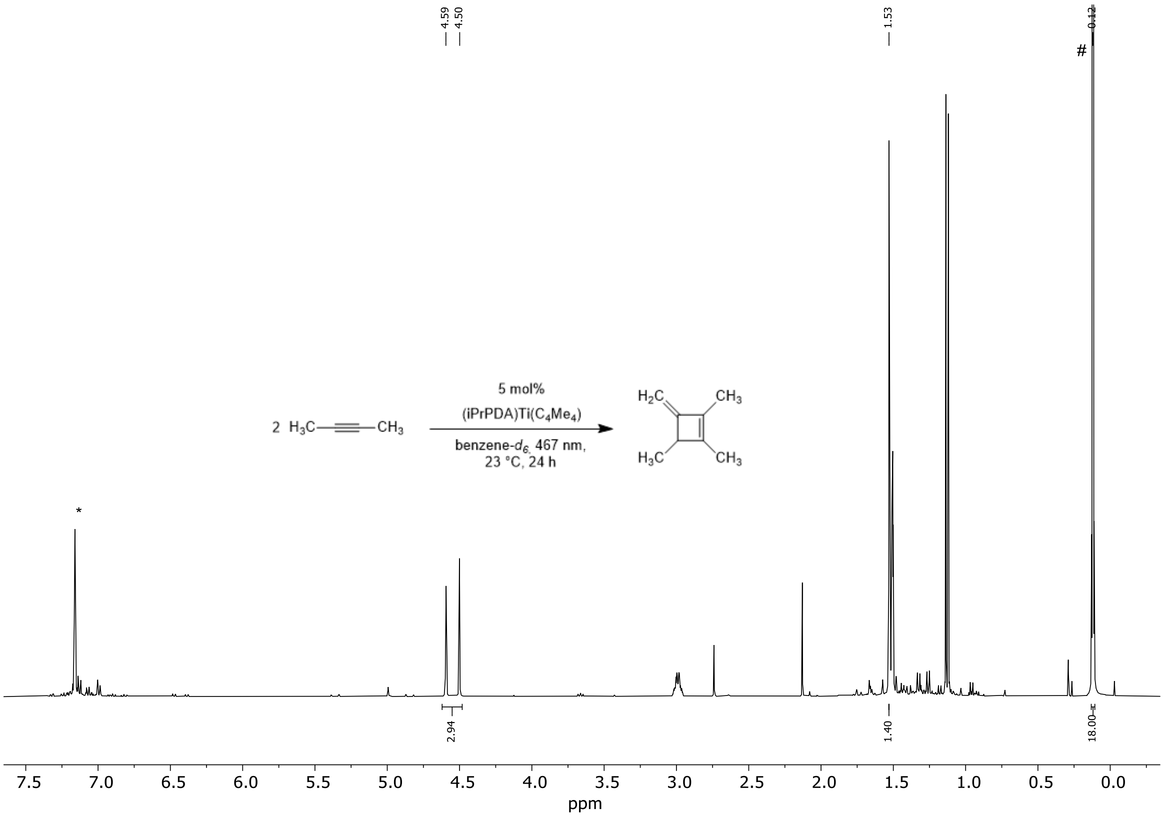

Figure S47. ^{1}H NMR spectrum (400 MHz, 298 K, benzene-d_6) after 24 h irradiation at 467 nm with (^{iPr}PDA)$Ti(C_4Me_4)$ instead of (^{iPr}PDA)$Ti(CH_2Ph)_2$ (Table 1, Entry 9); benzene-d_6 (*), HMDSO (#).

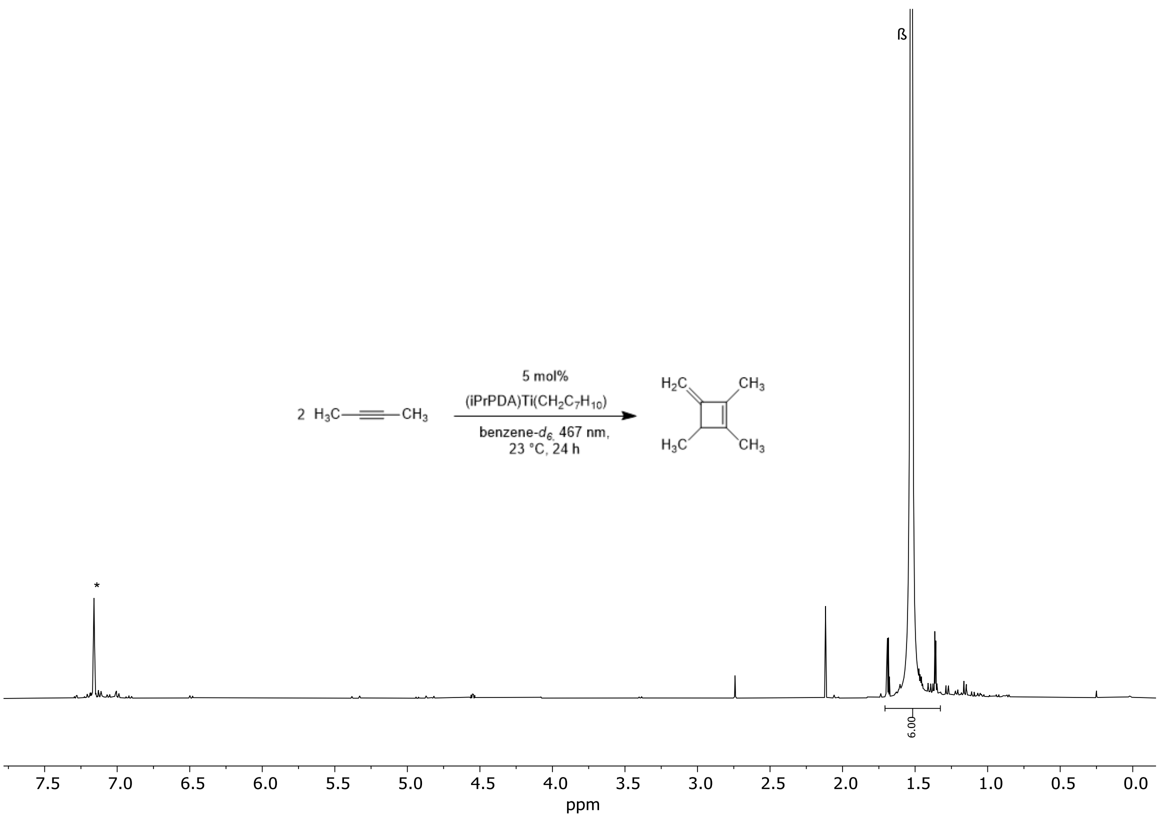

Figure S48. ^{1}H NMR spectrum (400 MHz, 298 K, benzene-d_6) after 24 h irradiation at 467 nm with (^{iPr}PDA)Ti($CH_2C_7H_{10}$) instead of (^{iPr}PDA)Ti(CH_2Ph)$_2$ (Table 1, Entry 10); benzene-d_6 (*), 2-butyne (ß).

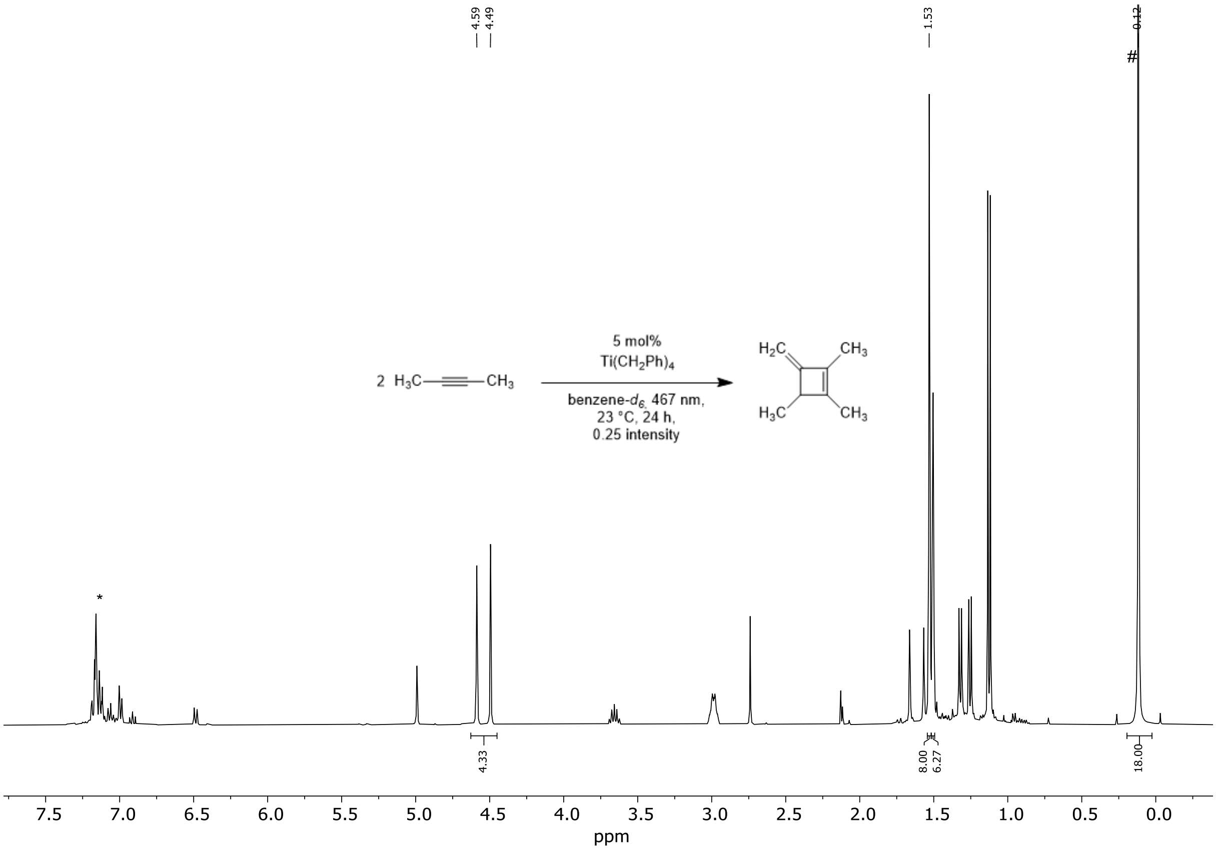

Figure S49. ^{1}H NMR spectrum (400 MHz, 298 K, benzene-d_6) after 24 h irradiation at 467 nm with 0.25 intensity instead of 0.5 intensity (Table 1, Entry 11); benzene-d_6 (*), HMDSO (#).

EXSY NMR Studies on Ground State Interaction

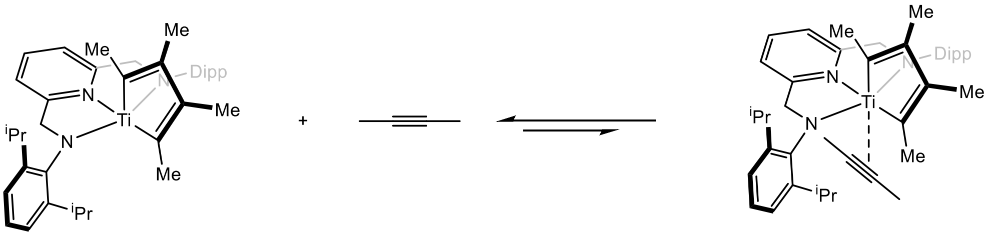

In an argon glovebox, (^{iPr}PDA)Ti(C_4Me_4) (10.0 mg, 16.4 µmol, 1.0 equiv.) was combined in a J Young NMR tube with 2-butyne (8.8 mg, 0.16 mmol, 10.0 equiv.) with the corresponding solvent. For 25 °C and 75 °C EXSY NMR measurements benzene-d_6 was used as the solvent and for the measurement at –40 °C toluene-d_8 was used.

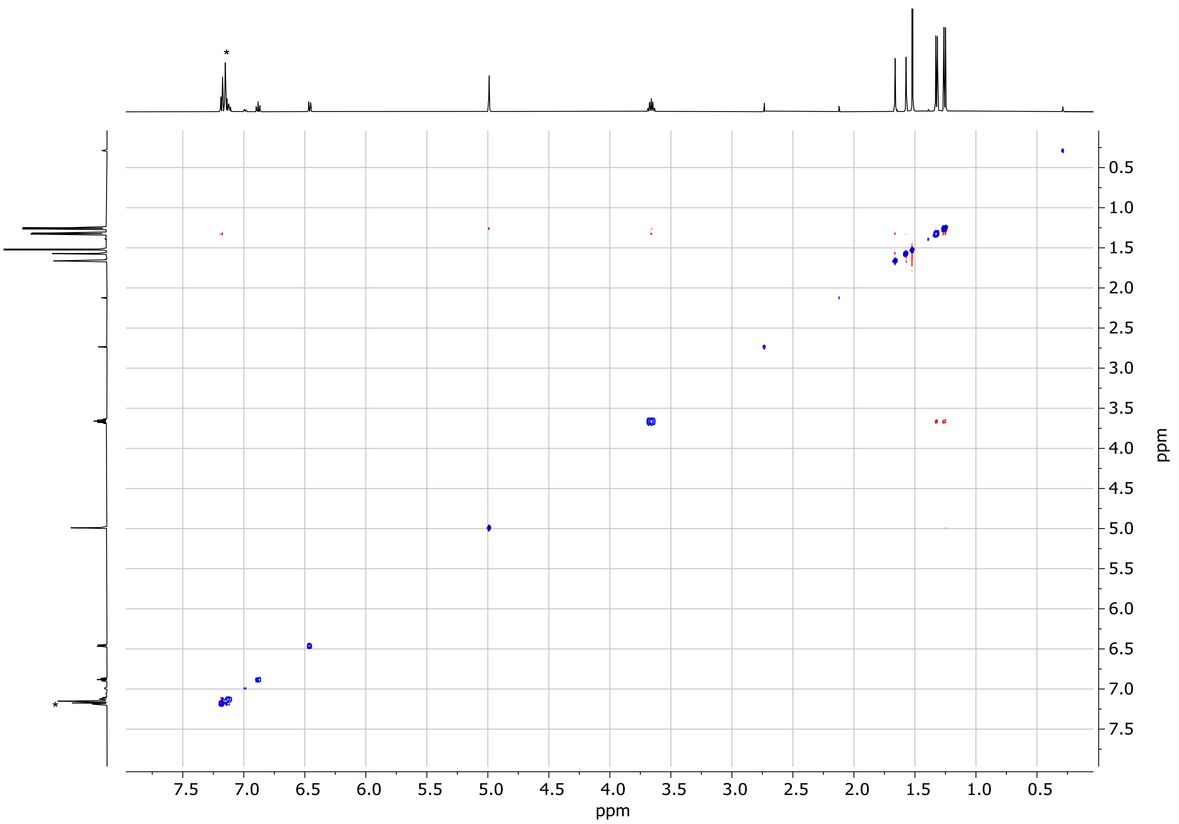

Figure S50. ^{1}H-^{1}H EXSY NMR spectrum (500 MHz, 298 K, benzene-d_6) of (^{iPr}PDA)Ti(C_4Me_4) and 2-butyne; benzene-d_6 (*).

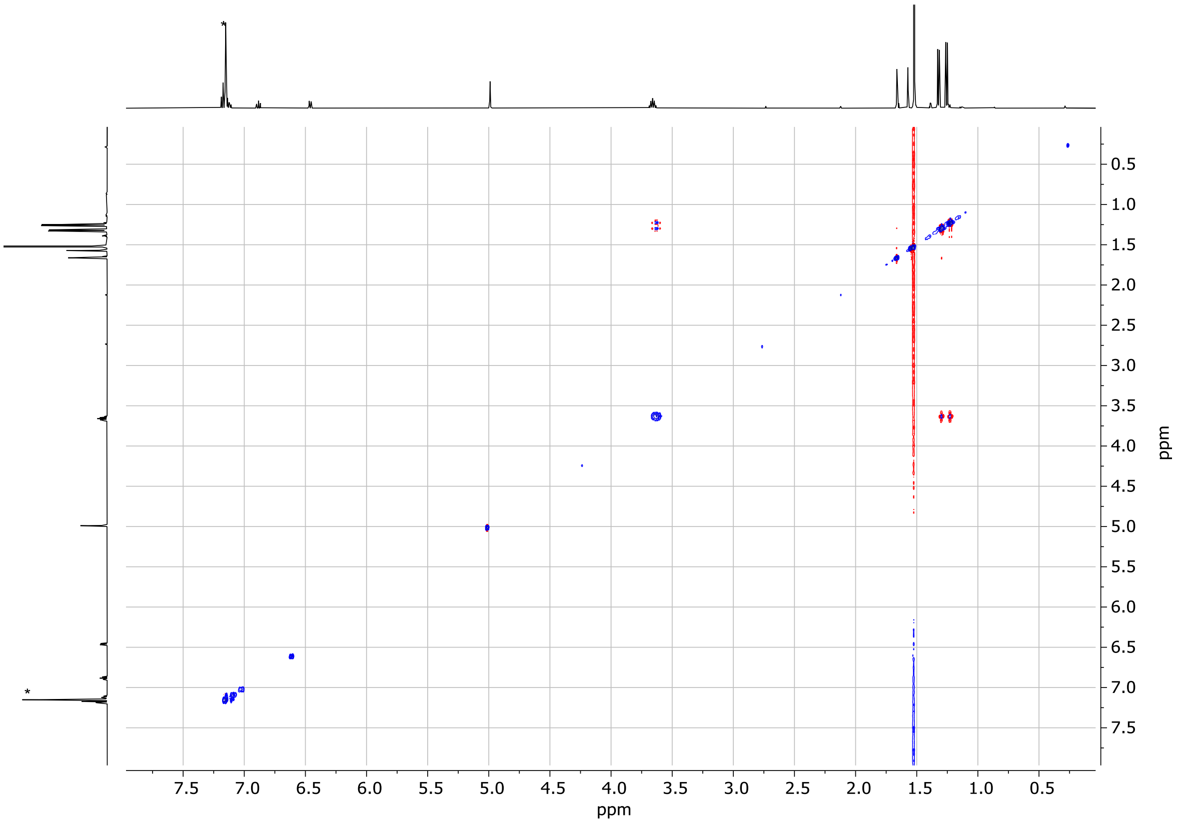

Figure S51. ^{1}H-^{1}H EXSY NMR spectrum (500 MHz, 348 K, benzene-d_6) of (^{iPr}PDA)Ti(C_4Me_4) and 2-butyne; benzene-d_6 (*).

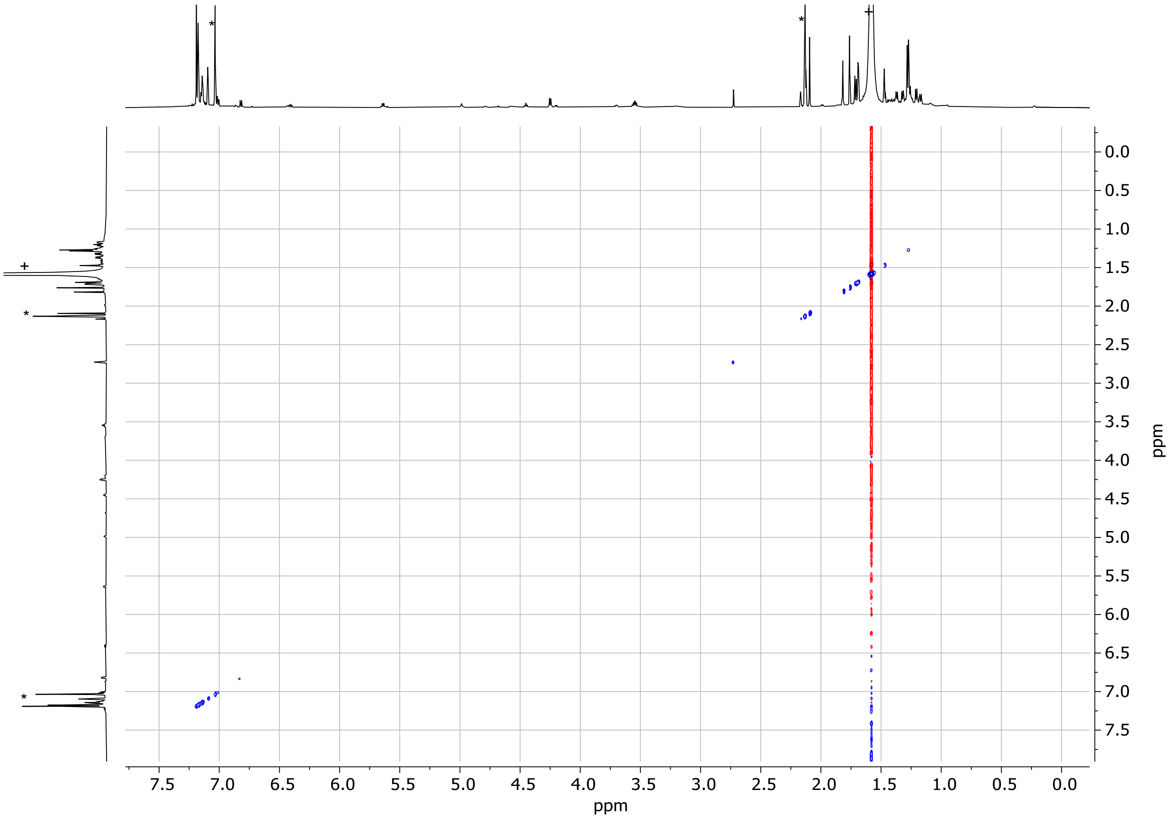

Figure S52. ^{1}H-^{1}H EXSY NMR spectrum (600 MHz, 233 K, toluene-d_8) of (^{iPr}PDA)Ti(C_4Me_4) and 2-butyne; toluene-d_8 (*), 2-butyne (+).

Single-Turnover Experiments

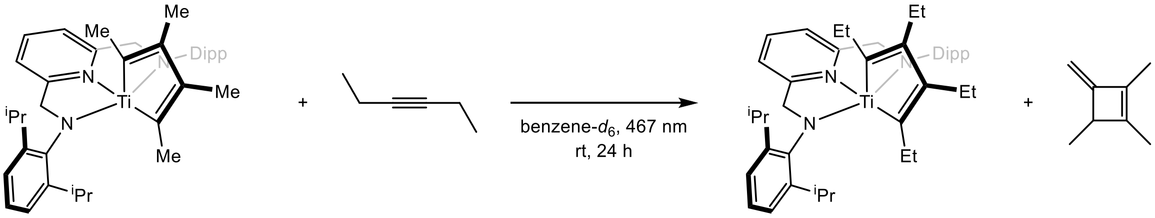

A J Young NMR tube was charged with (^{iPr}PDA)Ti(C_4Me_4) (20.0 mg, 0.33 mmol, 1.0 equiv), 3-hexyne (5.4 mg, 0.65 mmol, 2.0 equiv.) and benzene-d_6 (0.4 mL, 0.1 M). The J Young tube was placed in front of a 467 nm Kessil lamp and irradiated for 24 h at room temperature. The contents of the NMR tube were analyzed by ^{1}H NMR spectroscopy.

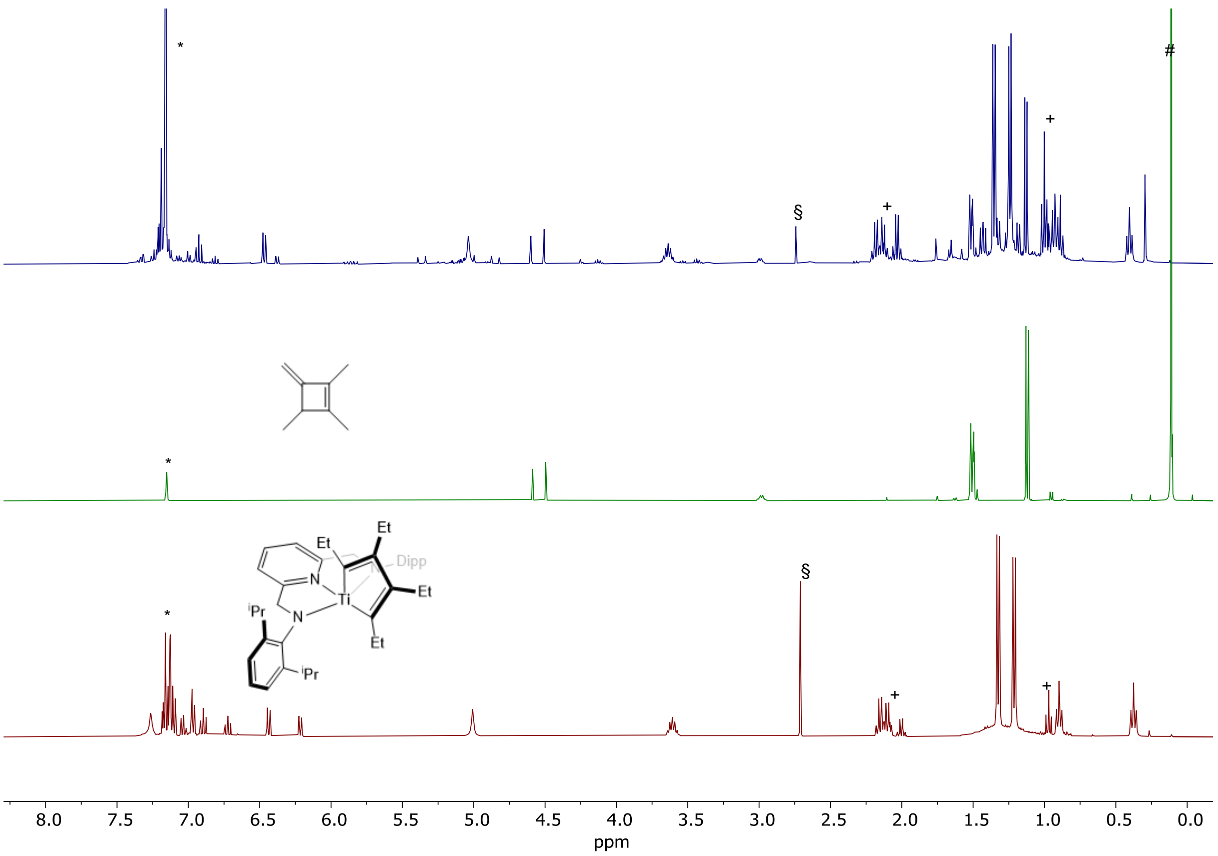

Figure S53. ^{1}H NMR spectrum (400 MHz, 298 K, benzene-d_6) of the single-turnover experiment of (^{iPr}PDA)Ti(C_4Me_4) with 3-hexyne; **Top:** ^{1}H NMR spectrum of the reaction mixture showing free 2-methylene cyclobutene; **Middle:** ^{1}H NMR spectrum of 1,2,3-trimethyl-4-methylenecyclobutene; **Bottom:** ^{1}H NMR spectrum of (^{iPr}PDA)Ti(C_4Et_4) (with residual 1,2-diphenylethane and 3-hexyne); benzene-d_6 (*), HMDSO (#), 3-hexyne (+), diphenylethane (§).

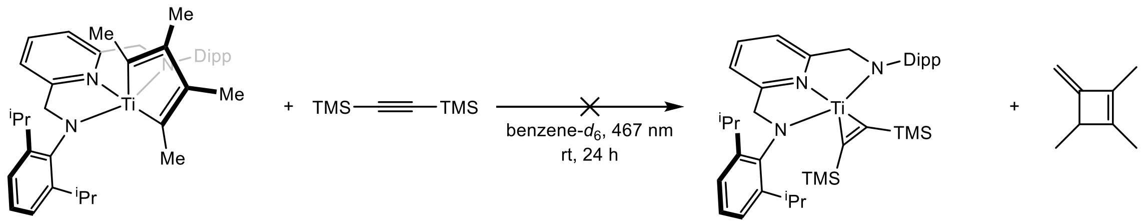

A J Young NMR tube was charged with (^{iPr}PDA)Ti(C_4Me_4) (20.0 mg, 0.33 mmol, 1.0 equiv) bis(trimethylsilylacetylene) (5.6 mg, 0.33 mmol, 1.0 equiv.) and benzene-d_6 (0.4 mL, 0.8 M). The J Young tube was placed in front of a 467 nm Kessil lamp and irradiated for 24 h at room temperature.

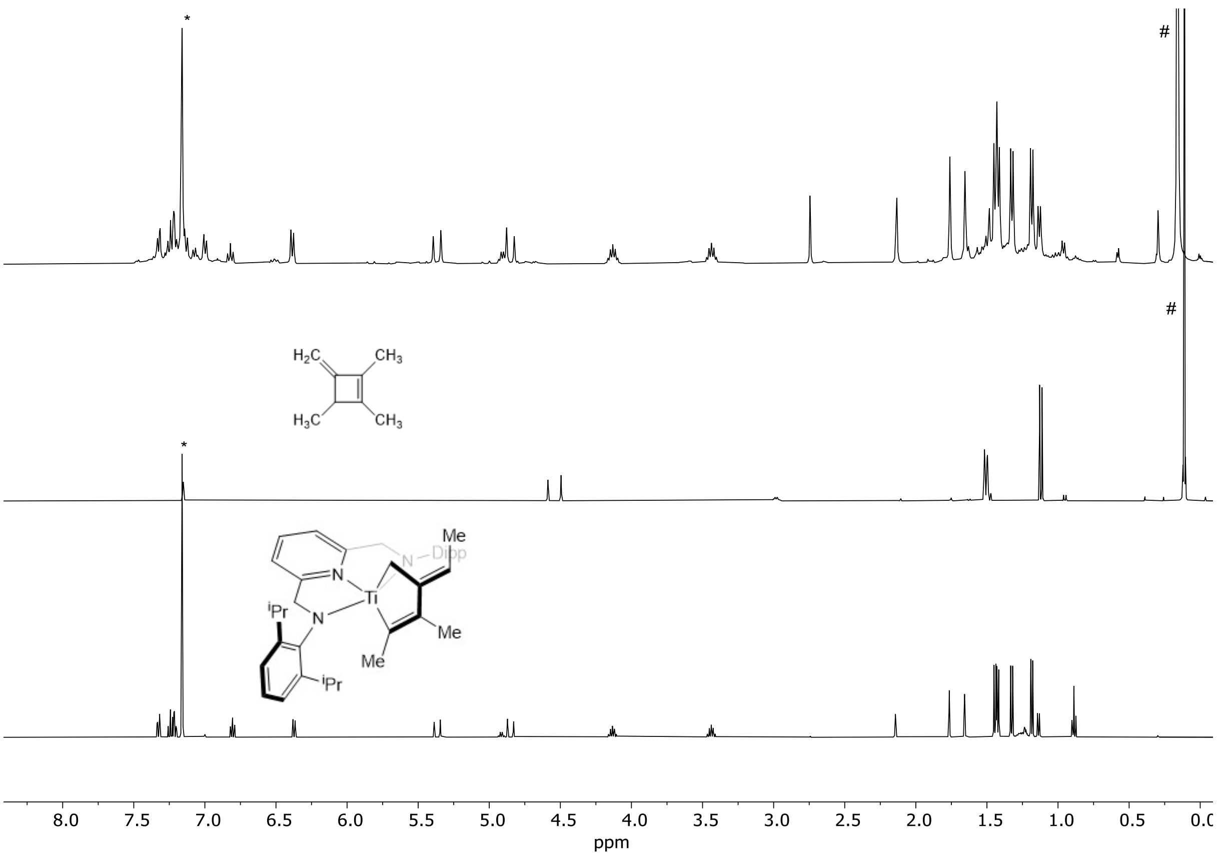

Figure S54. ^{1}H NMR spectrum (400 MHz, 298 K, benzene-d_6) of the single-turnover experiment with BTMSA; **Top:** ^{1}H NMR spectrum of the reaction mixture showing no free 4-methylene cyclobutene; **Middle:** ^{1}H NMR spectrum of isolated 2-methylene cyclobutene; **Bottom:** ^{1}H NMR spectrum of isolated (^{iPr}PDA)Ti($CH_2C_7H_{10}$); benzene-d_6(*).

Reductive cleavage of 1,2,3-trimethyl-4-methylenecyclobutene

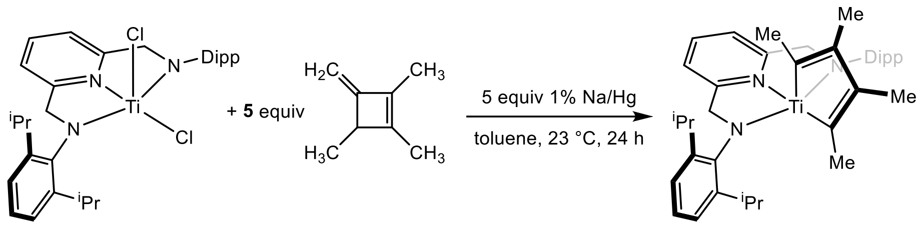

A 20 mL vial was charged with sodium (11.5 mg, 0.005 mmol, 1.0 equiv.) and toluene (2 mL). Mercury (1.4 g) was added, and the amalgam was stirred for 20 minutes. In a separate vial, (^{iPr}PDA)TiCl$_2$ (28.0 mg, 0.05 mmol, 1.0 equiv.) and methylene cyclobutene (35.0 mg, 0.32 mmol, 5.0 equiv.) were suspended in benzene (2 mL) and then added to the sodium amalgam. The reaction was stirred for 6 h at room temperature and subsequently filtered over Celite. The solvent was removed from the filtrate under reduced pressure. The residue was analyzed by ^{1}H NMR spectroscopy, showing the formation of (^{iPr}PDA)TiC$_4$Me$_4$ (

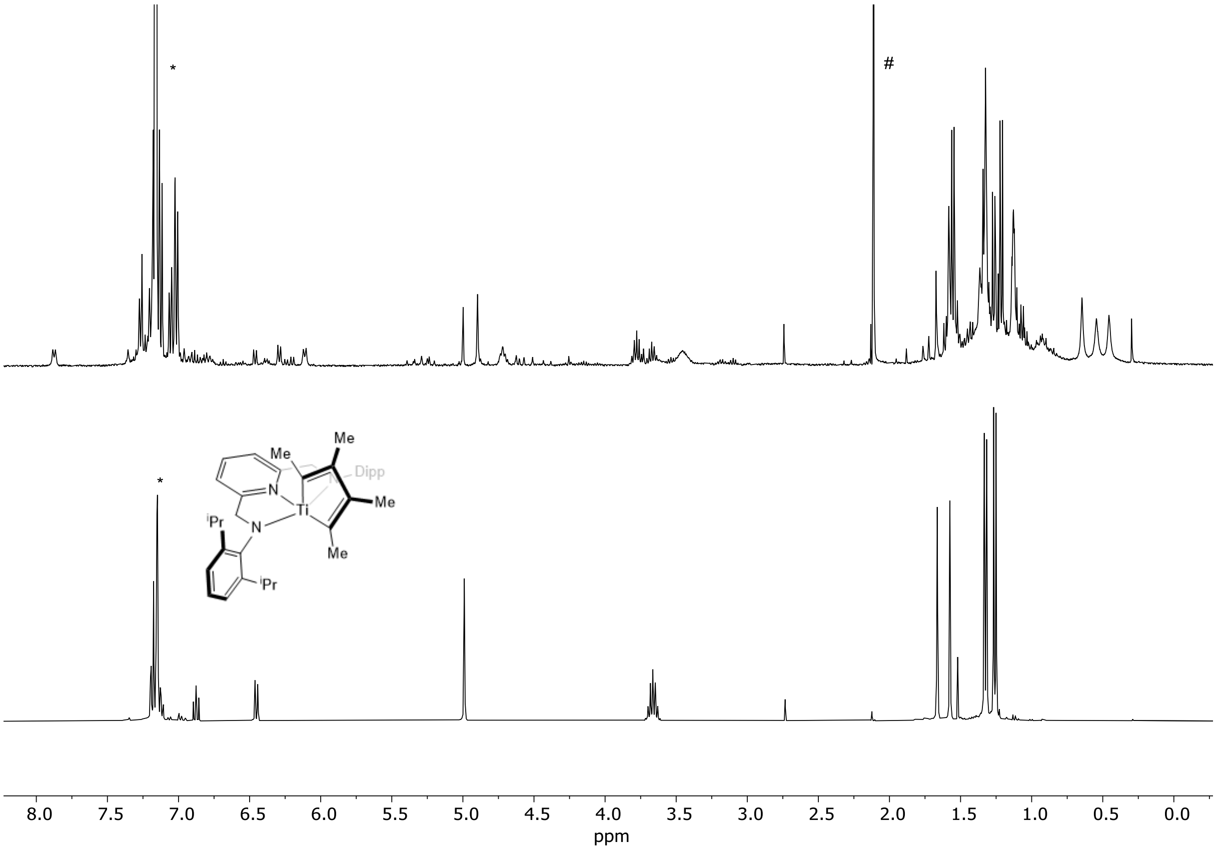

Figure **S*55***).

To ensure that methylenecyclobutene is not reduced by sodium amalgam, the reaction was repeated without the titanium complex.

A 20 mL vial was charged with sodium (736 mg, 0.32 mmol, 1.0 equiv.) and benzene-*d*$_6$ (3 mL). Mercury (89.6 g) was added, and the amalgam was stirred for 20 minutes. In a separate vial methylene cyclobutene (35.0 mg, 0.32 mmol, 1.0 equiv.) in benzene-*d*$_6$ (1 mL) and then added

to the sodium amalgam. The reaction was stirred for 6 h at room temperature and subsequently filtered over Celite. 0.4 mL of the filtered solution were transferred to a J Young NMR tube and HMDSO (10 µL, 47 µmol, 0.15 equiv.) was added. 2-Butyne was not detected by ^{1}H NMR spectroscopy (Figure S56).

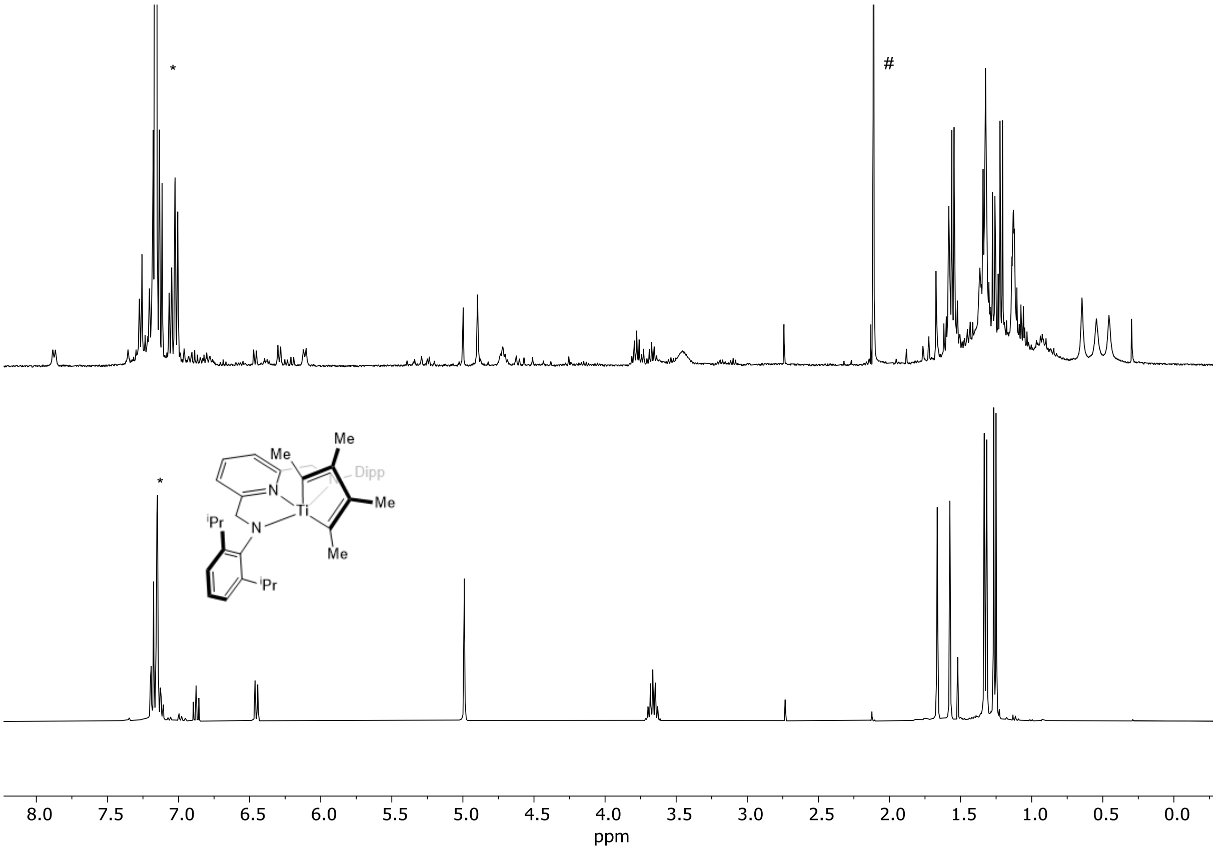

Figure S55. ^{1}H NMR spectra (400 MHz, 298 K, benzene-*d*$_6$) of the reductive cleavage of 1,2,3-trimethyl-4-methylenecyclobutene affording (^{iPr}PDA)Ti(C_4Me_4); **Top:** ^{1}H NMR of the reaction mixture showing (^{iPr}PDA)Ti(C_4Me_4) formation; **Bottom:** ^{1}H NMR spectrum of an authentic sample of (^{iPr}PDA)Ti(C_4Me_4); benzene-*d*$_6$ (*), toluene (#).

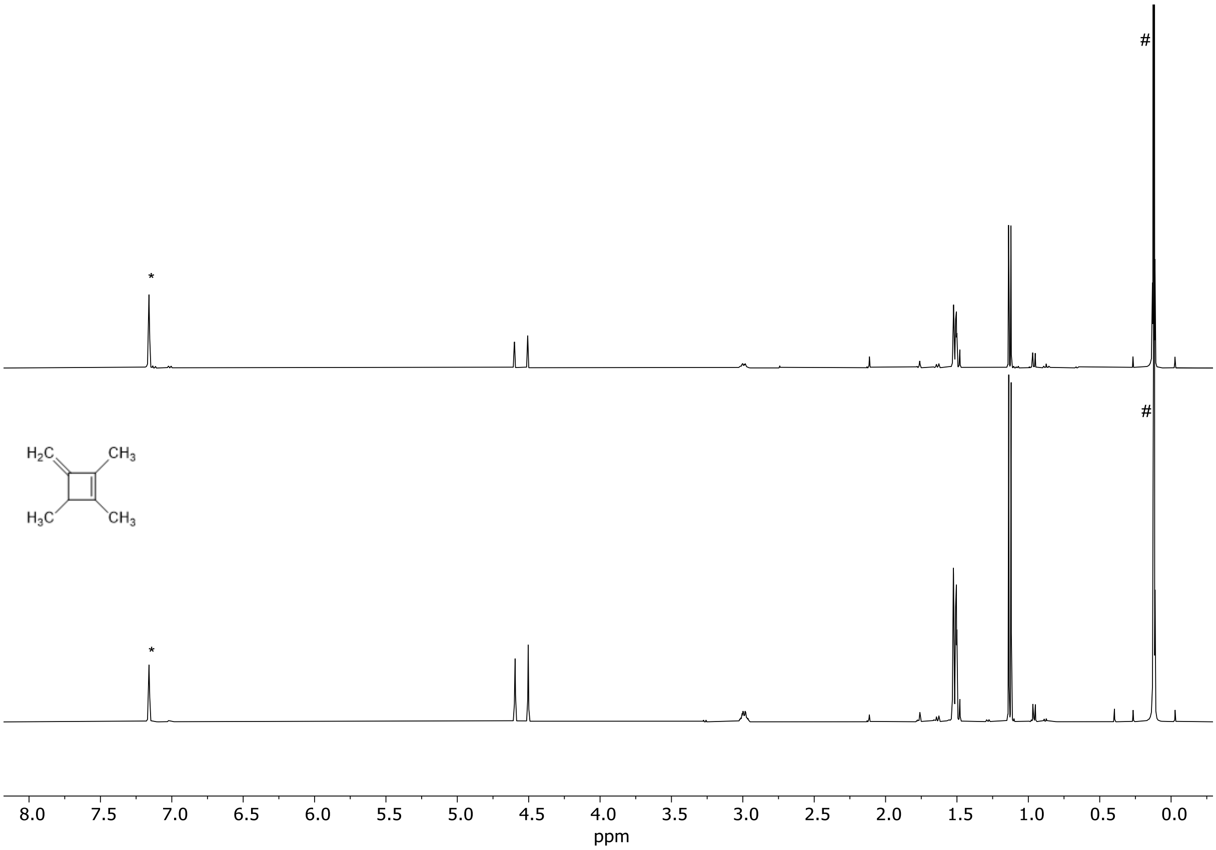

Figure S56. ^{1}H NMR spectra (400 MHz, 298 K, benzene-d_6) of the reduction of 1,2,3-trimethyl-4-methylenecyclobutene affording no reaction; **Top:** ^{1}H NMR spectrum of the reaction mixture after work-up showing no reactivity; **Bottom:** ^{1}H NMR spectrum of an authentic sample of 1,2,3-trimethyl-4-methylenecyclobutene; benzene-d_6 (*), HMDSO (#).

Oxidation of (^{iPr}PDA)TiC$_4$Me$_4$

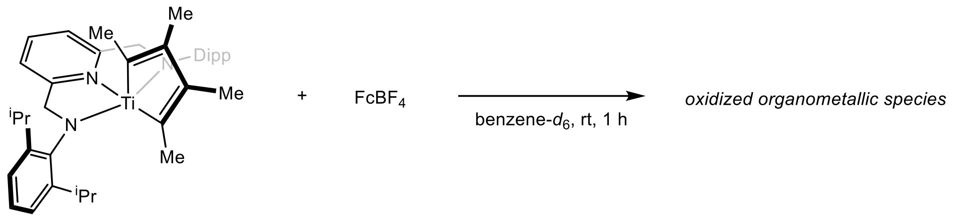

A J Young NMR tube was charged with (^{iPr}PDA)Ti(C$_4$Me$_4$) (30.0 mg, 0.49 mmol, 1.0 equiv.). A suspension of ferrocenium tetrafluoroborate (16.1 mg, 0.59 mmol, 1.2 equiv.) in benzene-d_6 (0.4 mL, 1.2 M) was added. The J Young tube was placed in an ultrasonic bath for 15 min and then continuously rotated for 1 h at room temperature. The contents of the NMR tube were subsequently analyzed by ^{1}H NMR spectroscopy, showing no formation of 1,2,3-trimethyl-4-methylenecyclobutene.

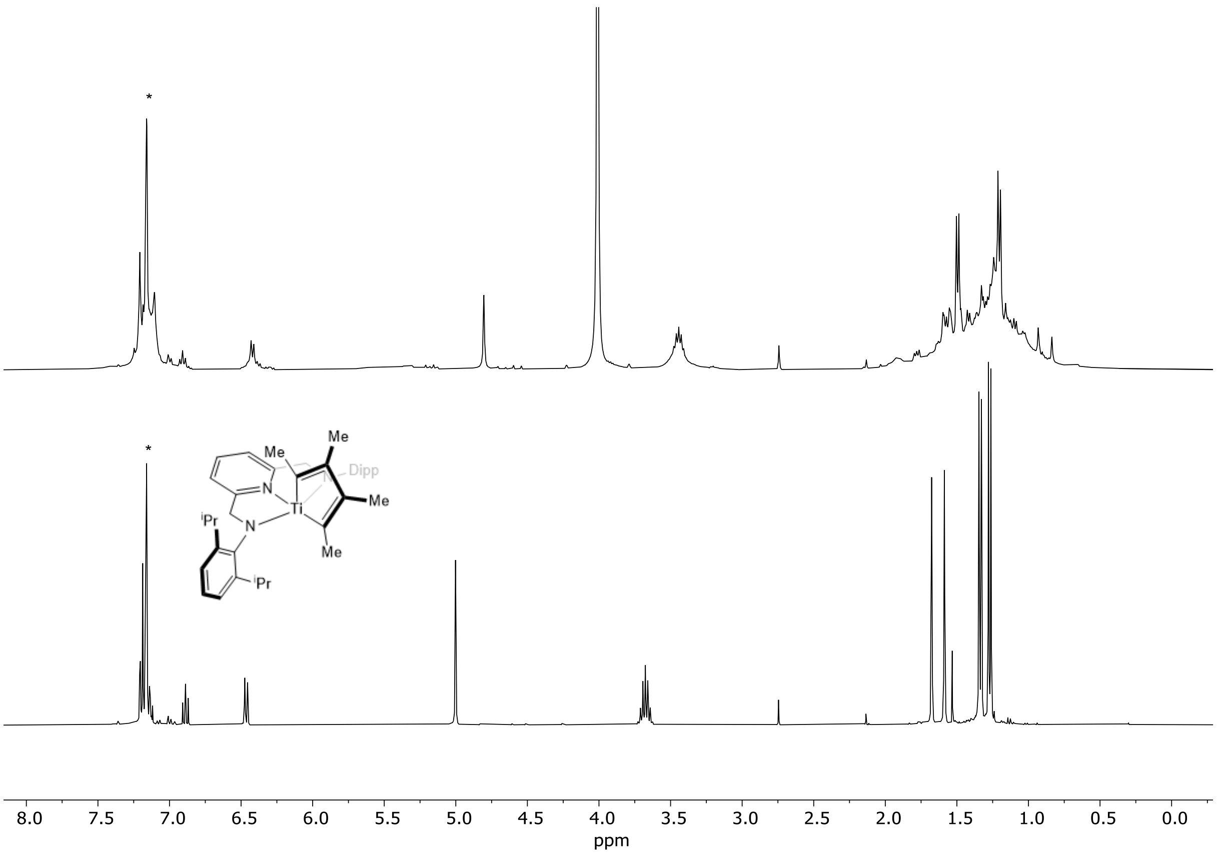

Figure S57. ^{1}H NMR spectrum (400 MHz, 298 K, benzene-d_6) of the oxidation of (^{iPr}PDA)Ti(C$_4$Me$_4$) with FcBF$_4$. **Top:** ^{1}H NMR spectrum of the reaction mixture. **Bottom:** ^{1}H NMR spectrum of isolated (^{iPr}PDA)Ti(C$_4$Me$_4$); benzene-d_6 (*).

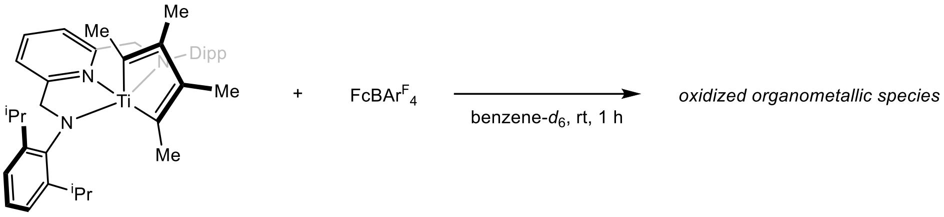

A J Young NMR tube was charged with (^{iPr}PDA)Ti(C_4Me_4) (30.0 mg, 0.49 mmol, 1.0 equiv). A suspension of $FcBAr^F{}_4$ (61.8 mg, 0.59 mmol, 1.2 equiv) in benzene-d_6 (0.4 mL, 1.2 M) was added. The J Young tube was placed in an ultrasonic bath for 15 min and then continuously rotated for 1 h at room temperature. The contents of the NMR tube were subsequently analyzed by ^{1}H NMR spectroscopy, showing no formation of 1,2,3-trimethyl-4-methylenecyclobutene.

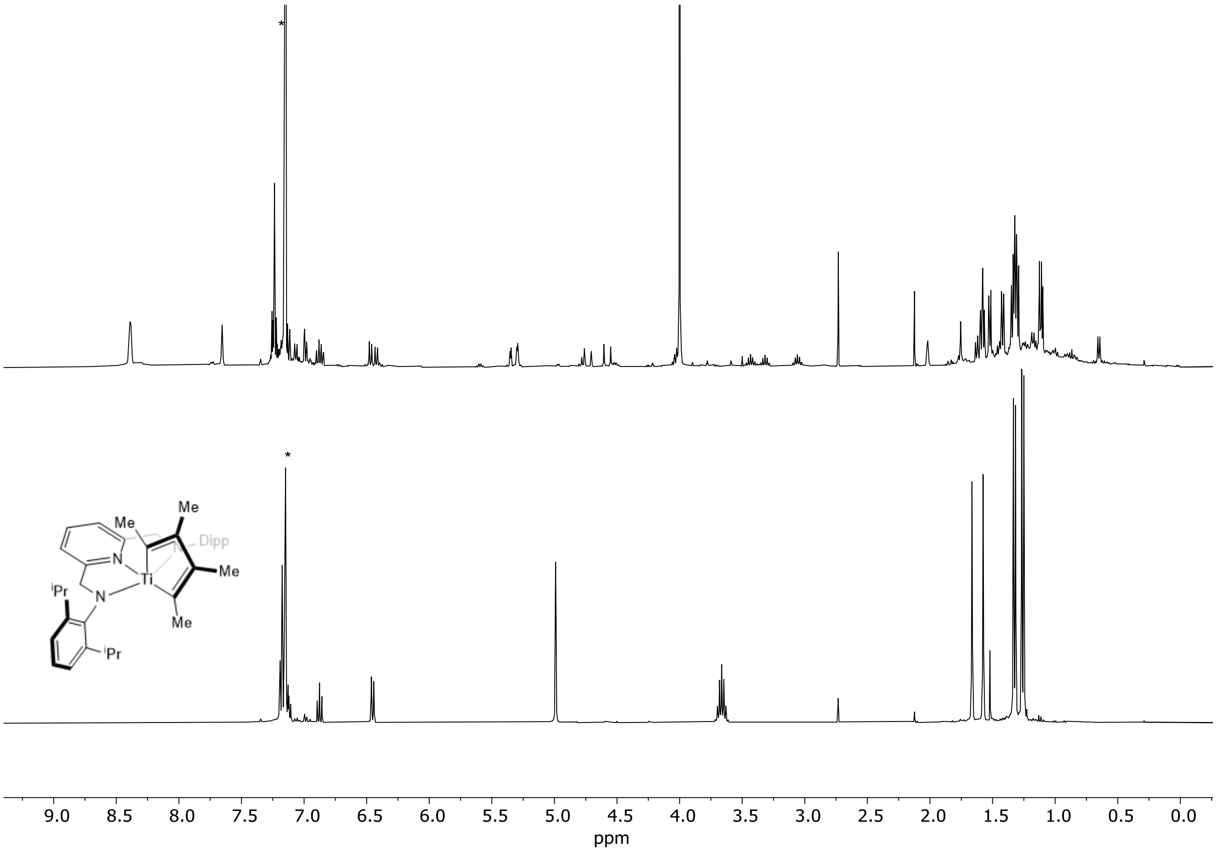

Figure S58. ^{1}H NMR spectrum (400 MHz, 298 K, benzene-d_6) of the oxidation of (^{iPr}PDA)Ti(C_4Me_4) with $FcBAr^F{}_4$. **Top:** ^{1}H NMR spectrum of the reaction mixture. **Bottom:** ^{1}H NMR spectrum of isolated (^{iPr}PDA)Ti(C_4Me_4); benzene-d_6 (*).

12. Substrate Scope

2-Butyne (1)

Following the standard procedure described above (Table 1).

3-Hexyne (2)

A J Young NMR tube was charged with (^{iPr}PDA)Ti(CH_2Ph)$_2$ (8.4 mg, 12.2 µmol, 5.0 mol%), 3-hexyne (20.0 mg, 0.24 mmol, 1.0 equiv.) and benzene-d_6 (0.4 mL, 0.6M). HMDSO (10 µL, 47 µmol) was added to quantify the yield and conversion by ^{1}H NMR spectroscopy. The J Young tube was placed in front of a 467 nm Kessil lamp and irradiated for 24 h at room temperature. The product was condensed in an empty J Young tube under reduced pressure using a vacuum line (10^{-2} mbar) (Table 1).

2-Pentyne (3)

A J Young NMR tube was charged with (^{iPr}PDA)Ti(CH_2Ph)$_2$ (10.1 mg, 14.7 µmol, 5.0 mol%), 2-pentyne (20.0 mg, 0.29 mmol, 1.0 equiv.) and benzene-d_6 (0.45 mL, 0.6M). HMDSO (10 µL, 47 µmol) was added to quantify the yield and conversion by ^{1}H NMR spectroscopy. The J Young tube was placed in front of a 467 nm Kessil lamp and irradiated for 24 h at room temperature. The product was condensed in an empty J Young tube under reduced pressure using a vacuum line (10^{-2} mbar) (Table 1).

2-Hexyne (4)

A J Young NMR tube was charged with (^{iPr}PDA)Ti(CH_2Ph)$_2$ (8.4 mg, 12.2 µmol, 5.0 mol%), 2-hexyne (20.0 mg, 0.24 mmol, 1.0 equiv.) and benzene-d_6 (0.4 mL, 0.6M). HMDSO (10 µL, 47 µmol) was added to quantify the yield and conversion by ^{1}H NMR spectroscopy. The J Young tube was placed in front of a 467 nm Kessil lamp and irradiated for 24 h at room temperature. The product was condensed in an empty J Young tube under reduced pressure using a vacuum line (10^{-2} mbar) (Table 1).

Methylphenylacetylene (5)

A J Young NMR tube was charged with (^{iPr}PDA)Ti(CH_2Ph)$_2$ (10.3 mg, 15.0 µmol, 5.0 mol%), methylphenylacetylene (34.8 mg, 0.30 mmol, 1.0 equiv.) and benzene-d_6 (0.5 mL, 0.6M). HMDSO (10 µL, 47 µmol) was added to quantify the yield and conversion by ^{1}H NMR spectroscopy. The J Young tube was placed in front of a 427 nm Kessil lamp and irradiated for

24 h at room temperature. The product was condensed in an empty J Young tube under reduced pressure using a vacuum line (10^{-2} mbar) (Table 1).

Diphenylacetylene (6)

A J Young NMR tube was charged with (^{iPr}PDA)Ti(CH_2Ph)$_2$ (10.0 mg, 14.6 μmol, 2.0 mol%), diphenylacetylene (130 mg, 0.73 mmol, 1.0 equiv.) and benzene-*d*$_6$ (0.5 mL, 1.5M). HMDSO (10 μL, 47 μmol) was added to quantify the yield and conversion by ^{1}H NMR spectroscopy. The J Young tube was placed in front of a 427 nm Kessil lamp and irradiated for 24 h at room temperature. The product was condensed in an empty J Young tube under reduced pressure using a vacuum line (10^{-2} mbar) (Table 1).

Cyclooctyne (7)

A J Young NMR tube was charged with (^{iPr}PDA)Ti(CH_2Ph)$_2$ (15.0 mg, 24.0 μmol, 5.0 mol%), cyclooctyne (52.6 mg, 0.48 mmol, 1.0 equiv.) and benzene-*d*$_6$ (0.6 mL, 0.8M). HMDSO (10 μL, 47 μmol) was added to quantify the yield and conversion by ^{1}H NMR spectroscopy. The J Young tube was placed in front of a 427 nm Kessil lamp and irradiated for 24 h at room temperature. The product was condensed in an empty J Young tube under reduced pressure using a vacuum line (10^{-2} mbar) (Table 1).

2,7-Nonadiyne (8)

A J Young NMR tube was charged with (^{iPr}PDA)Ti(CH_2Ph)$_2$ (15.0 mg, 24.0 μmol, 5.0 mol%), 2,7-nonadiyne (58.0 mg, 0.48 mmol, 1.0 equiv.) and benzene-*d*$_6$ (0.6 mL, 0.8M). HMDSO (10 μL, 47 μmol) was added to quantify the yield and conversion by ^{1}H NMR spectroscopy. The J Young tube was placed in front of a 427 nm Kessil lamp and irradiated for 24 h at room temperature. The product was condensed in an empty J Young tube under reduced pressure using a vacuum line (10^{-2} mbar) (Table 1).

tert-Butyl acetylene (9)

A J Young NMR tube was charged with (^{iPr}PDA)Ti(CH_2Ph)$_2$ (4.2 mg, 6.1 μmol, 2.0 mol%), tert-butyl acetylene (25.0 mg, 0.30 mmol, 1.0 equiv.) and benzene-*d*$_6$ (0.6 mL, 0.8M). HMDSO (10 μL, 47 μmol) was added to quantify the yield and conversion by ^{1}H NMR spectroscopy. The J Young tube was placed in front of a 427 nm Kessil lamp and irradiated for 24 h at room

temperature. The product was condensed in an empty J Young tube under reduced pressure using a vacuum line (10^{-2} mbar) (Table 1).

Table S11. Substrates tested for photochemical cyclodimerization.

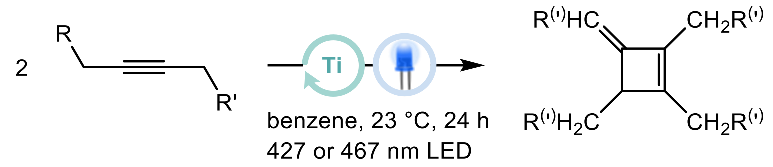

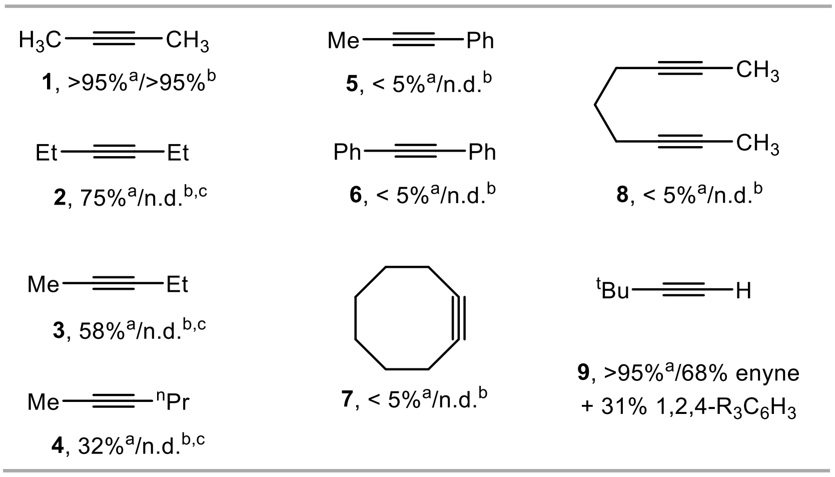

a) conversion b) cyclobutene yield c) due to the formation of complex isomeric mixtures, the yield of the cyclic dimers could not be determined unambiguously. GC-MS data, however, clearly show the formation of alkyne dimers.

Generally, reaction mixtures were analyzed by ^{1}H NMR spectroscopy directly after the reaction to determine the conversion of alkyne. Further analysis of the product mixture was performed with the volatiles of the reaction mixtures, revealing complex mixtures of alkyne dimers (for reactions with **2**, **3** and **4**).

With substrates **5**, **6**, **7** and **8**, formation of the metallacycle was observed and no new organic products were identified. The terminal alkyne **9** afforded a mixture of the *Z*-enyne and 1,2,4-tri-*tert*-butylbenzene.[23] For all substrates, the presence of polymers cannot be excluded unambiguously.

3-Hexyne

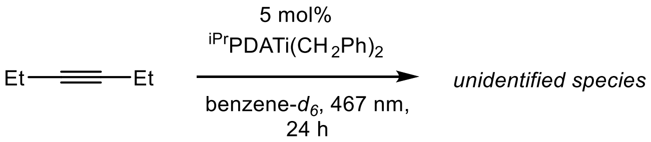

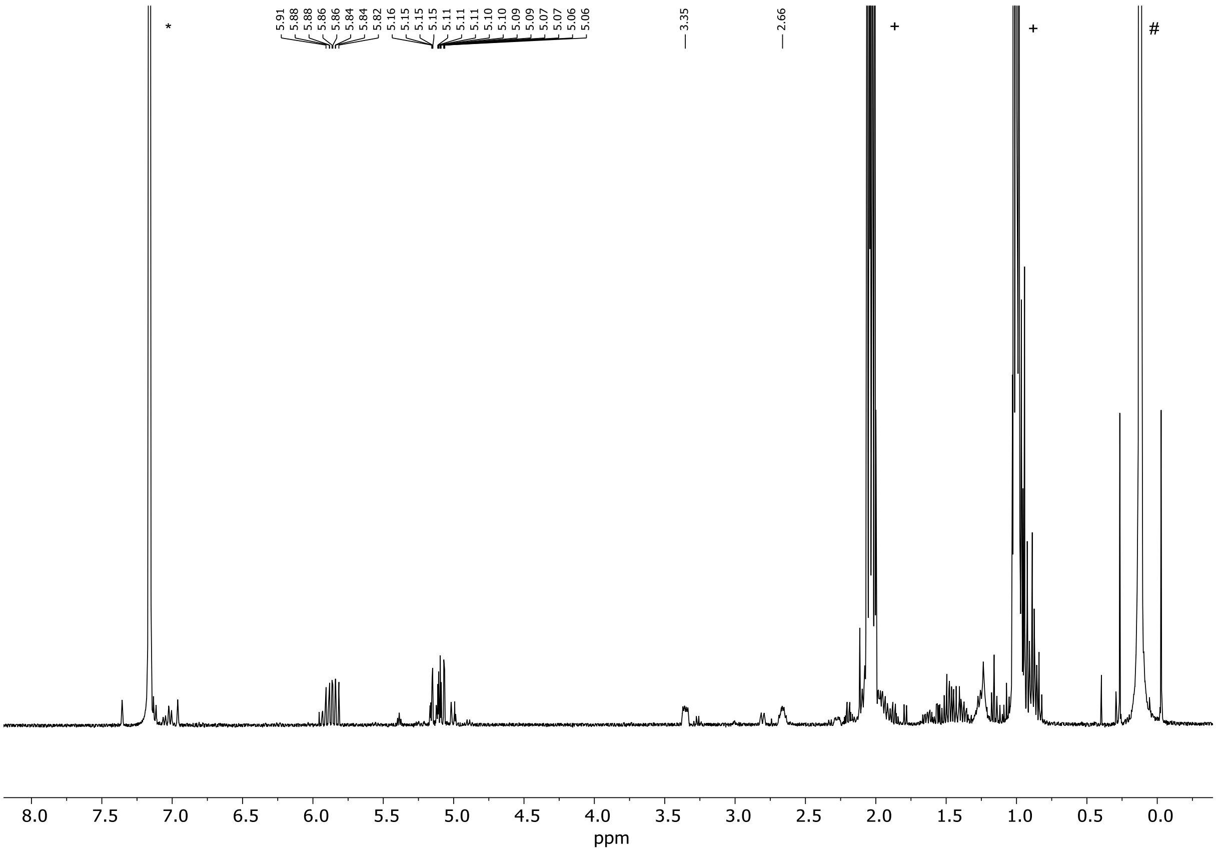

Figure S59. ^{1}H NMR spectrum (400 MHz, 298 K, benzene-d_6) of volatiles of the reaction with 3-hexyne; benzene-d_6 (*), HMDSO (#), 3-hexyne (+).

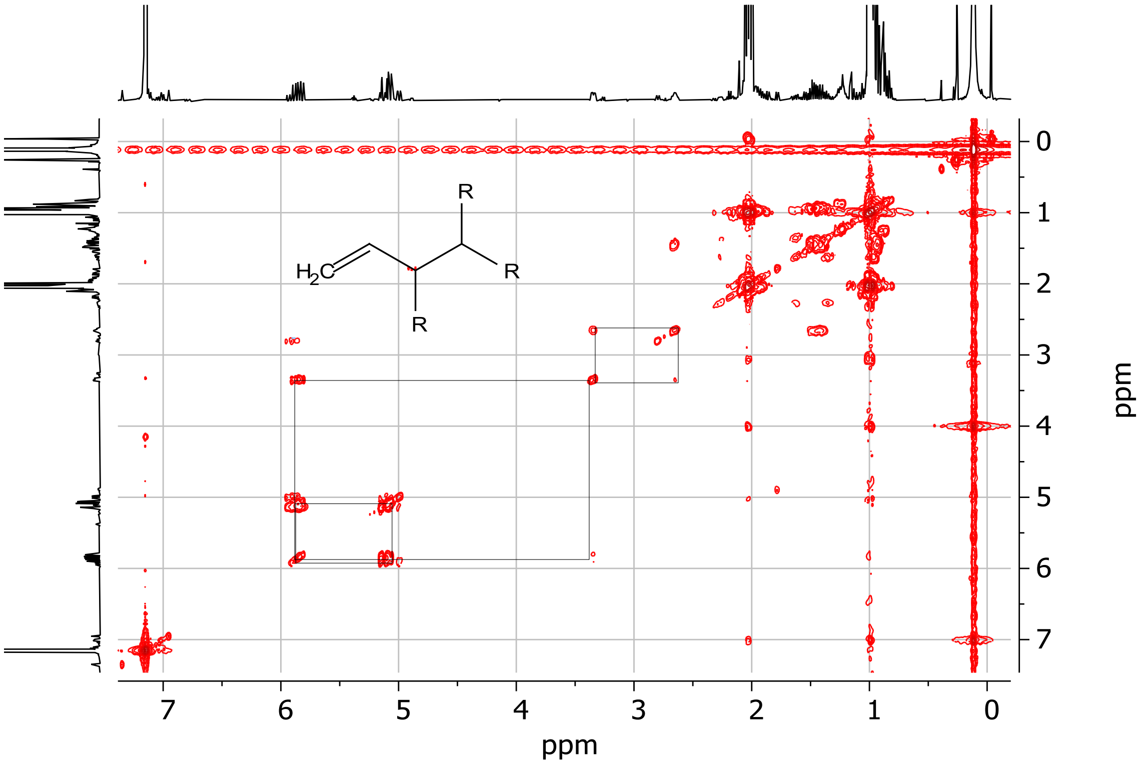

Figure S60. ^{1}H-^{1}H COSY NMR spectrum (400 MHz, 298 K, benzene-d_6) of the volatiles of the reaction with 3-hexyne.

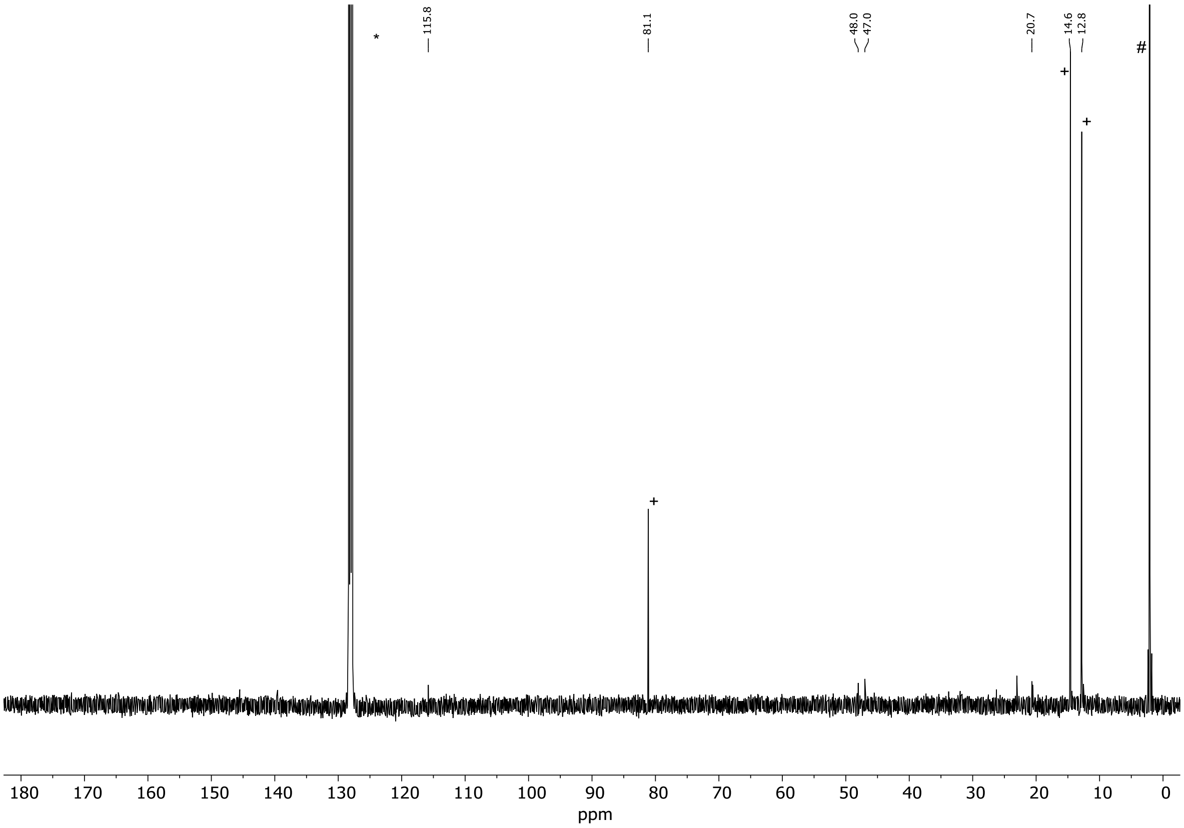

Figure 61. $^{13}C\{^{1}H\}$ NMR spectrum (101 MHz, 298 K, benzene-d_6) of the volatiles of the reaction with 3-hexyne; benzene-d_6 (*), HMDSO (#), 3-hexyne (+).

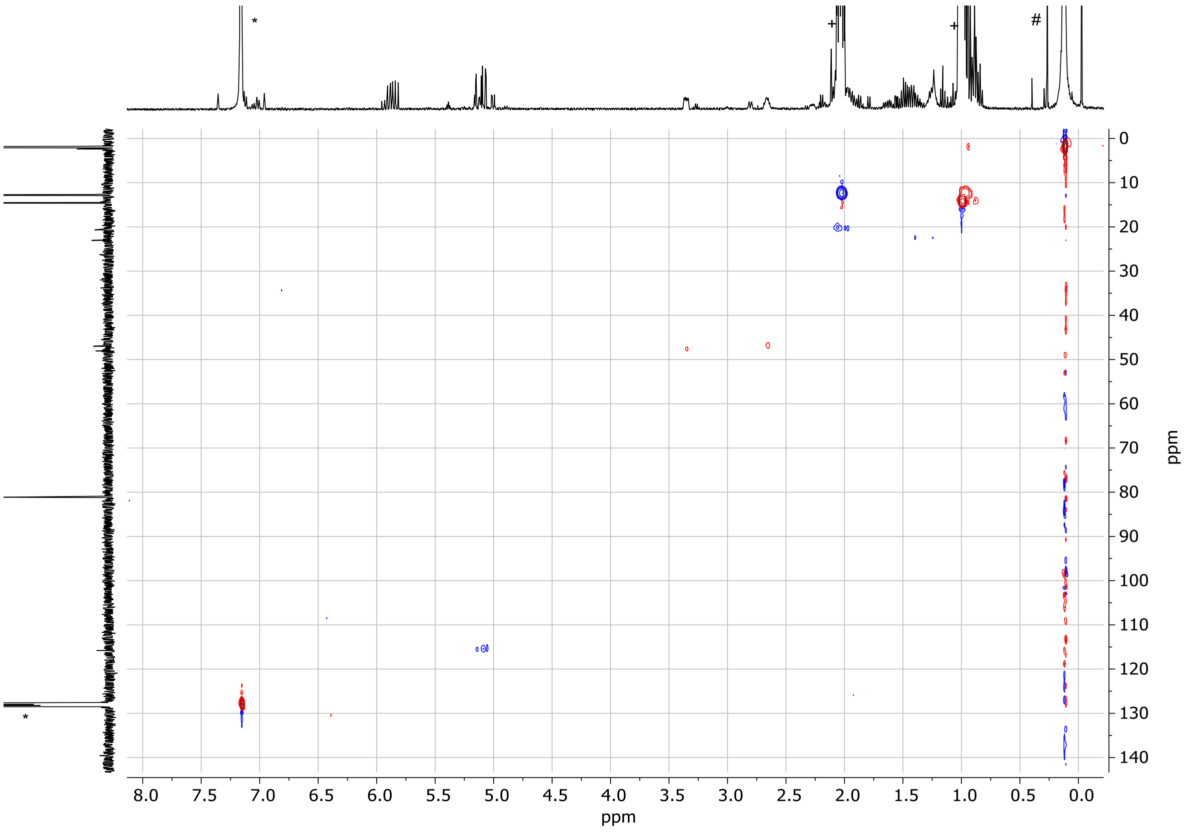

Figure S62. ^{1}H-^{13}C HSQC NMR spectrum (400 MHz, 101 MHz, 298 K, benzene-d_6) of the volatiles of the reaction with 3-hexyne; benzene-d_6 (*), HMDSO (#), 3-hexyne (+).

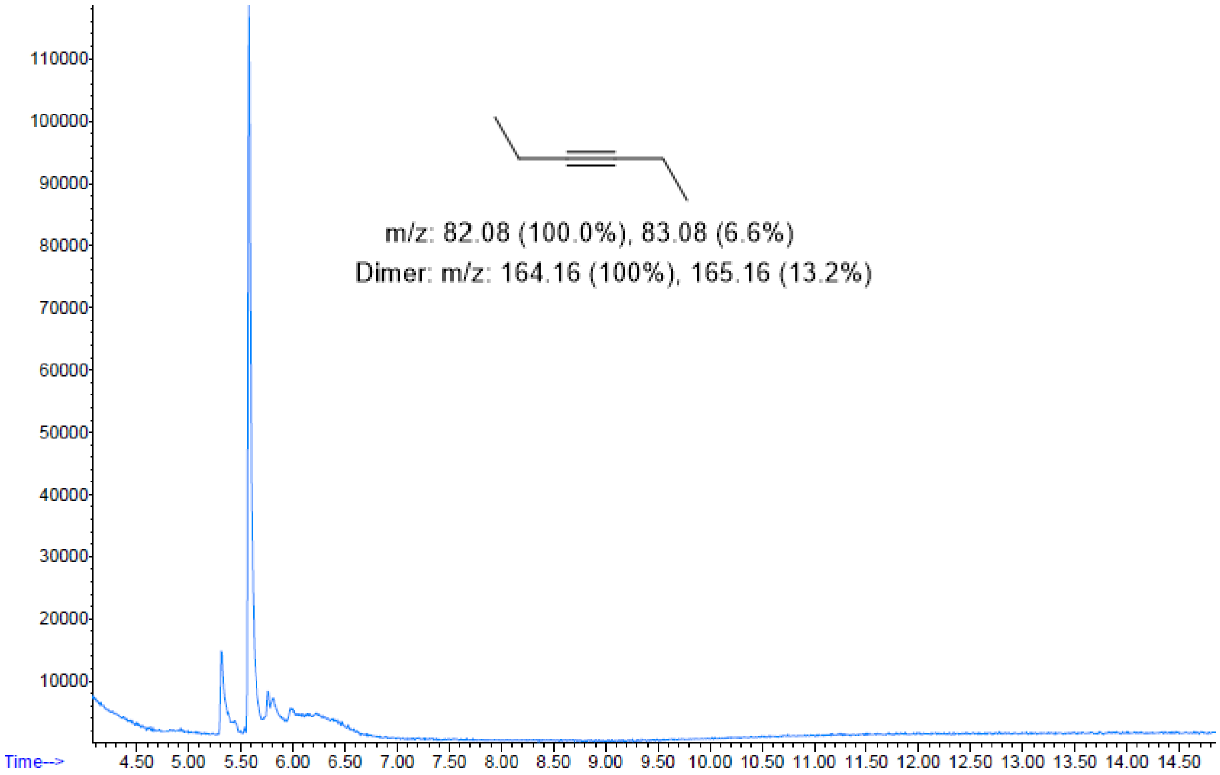

Figure S63. GC-MS chromatogram of the volatiles of the reaction with 3-hexyne.

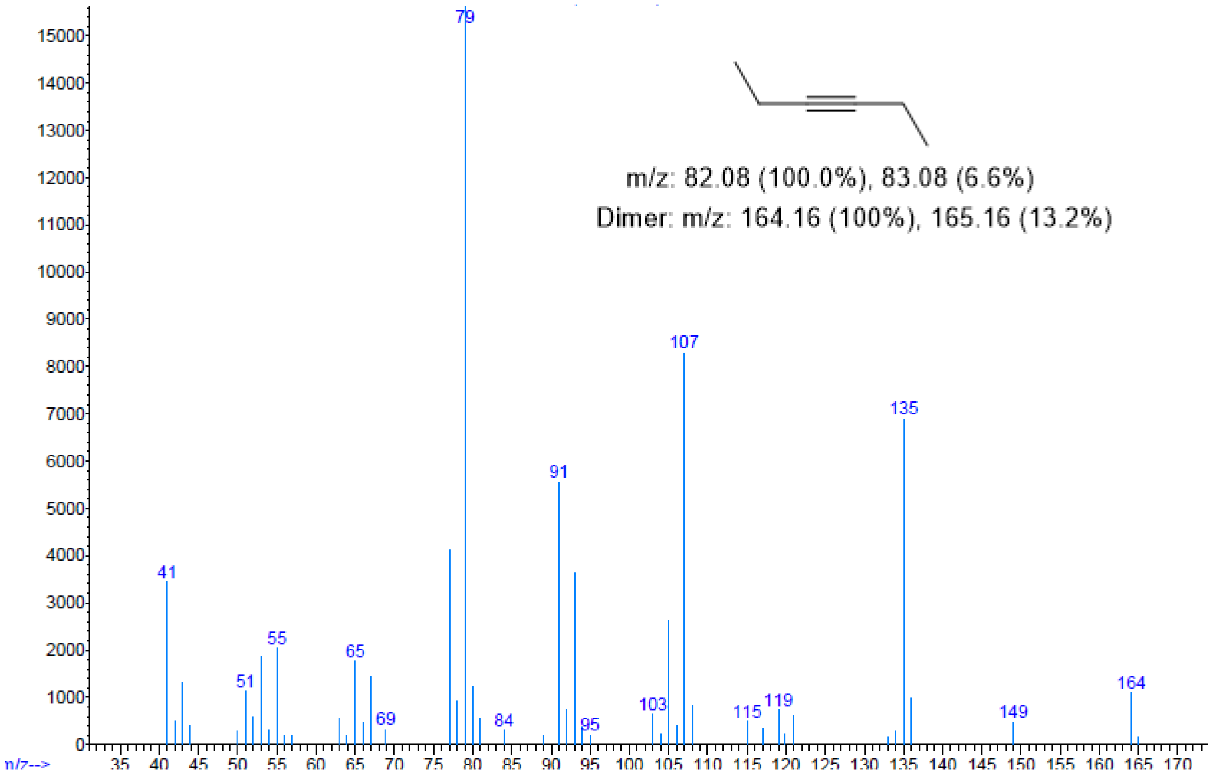

Figure S64. GC-MS spectrum (5.591 min) of the volatiles of the reaction with 3-hexyne.

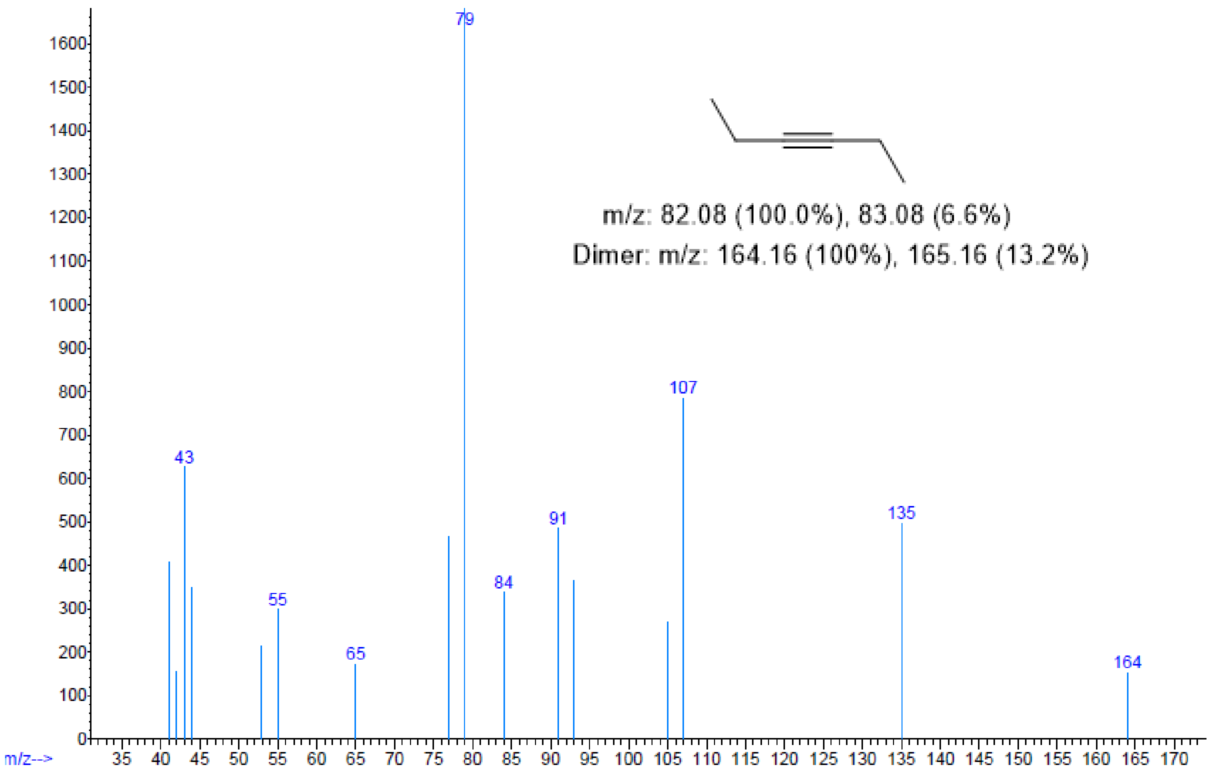

Figure S65. GC-MS spectrum (5.807 min) of the volatiles of the reaction with 3-hexyne.

2-Hexyne

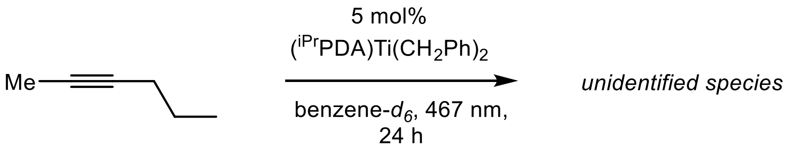

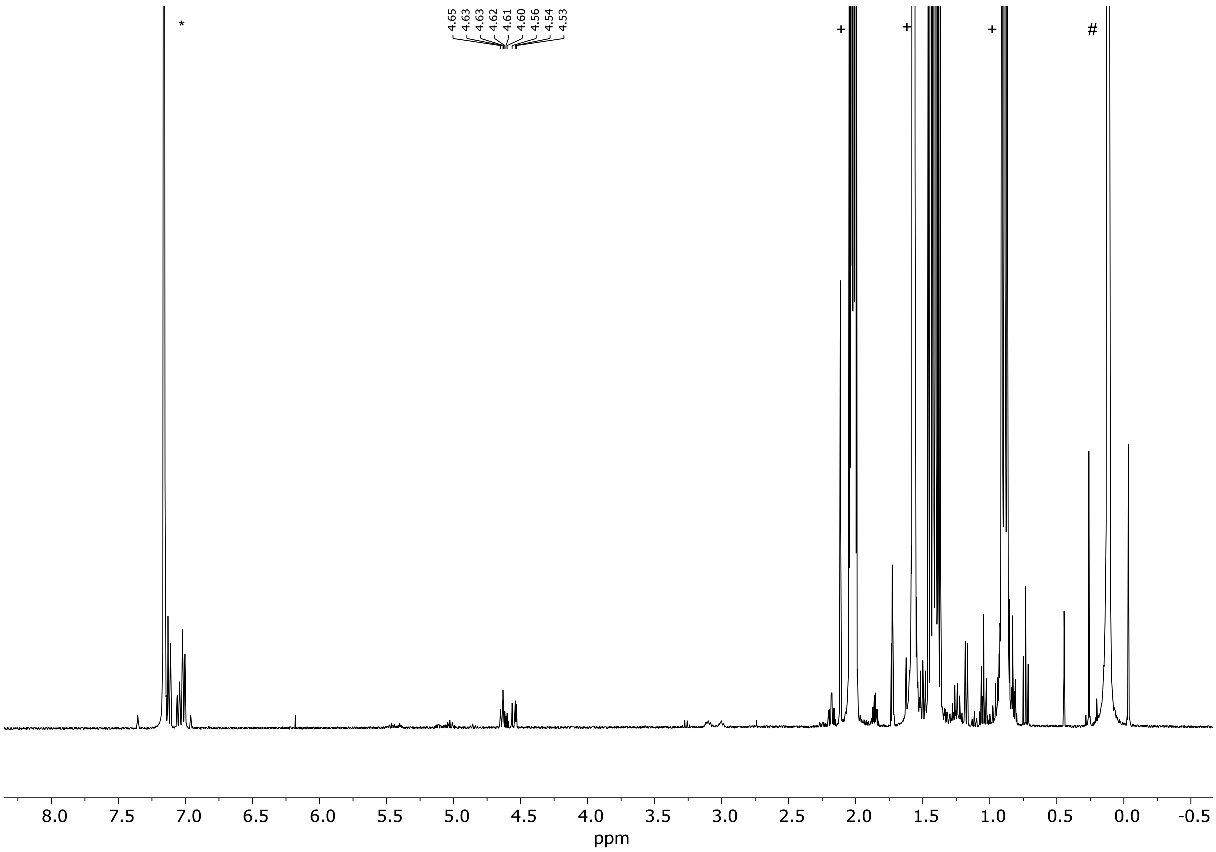

Figure S66. ^{1}H NMR spectrum (400 MHz, 298 K, benzene-d_6) of volatiles of the reaction with 2-hexyne; benzene-d_6 (*), HMDSO (#), 2-hexyne (+).

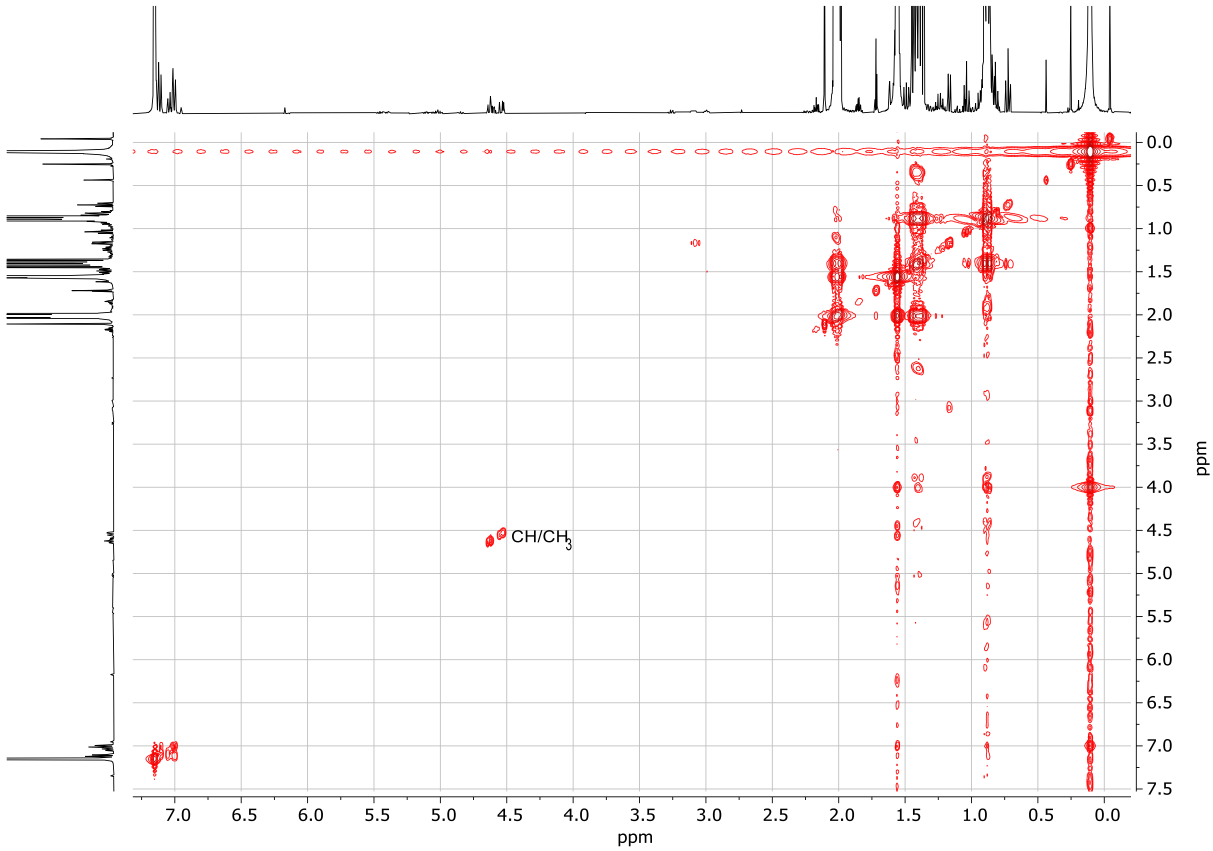

Figure S67. ^{1}H-^{1}H COSY NMR spectrum (400 MHz, 298 K, benzene-d_6) of the volatiles of the reaction with 2-hexyne; benzene-d_6 (*), HMDSO (#), 2-hexyne (+).

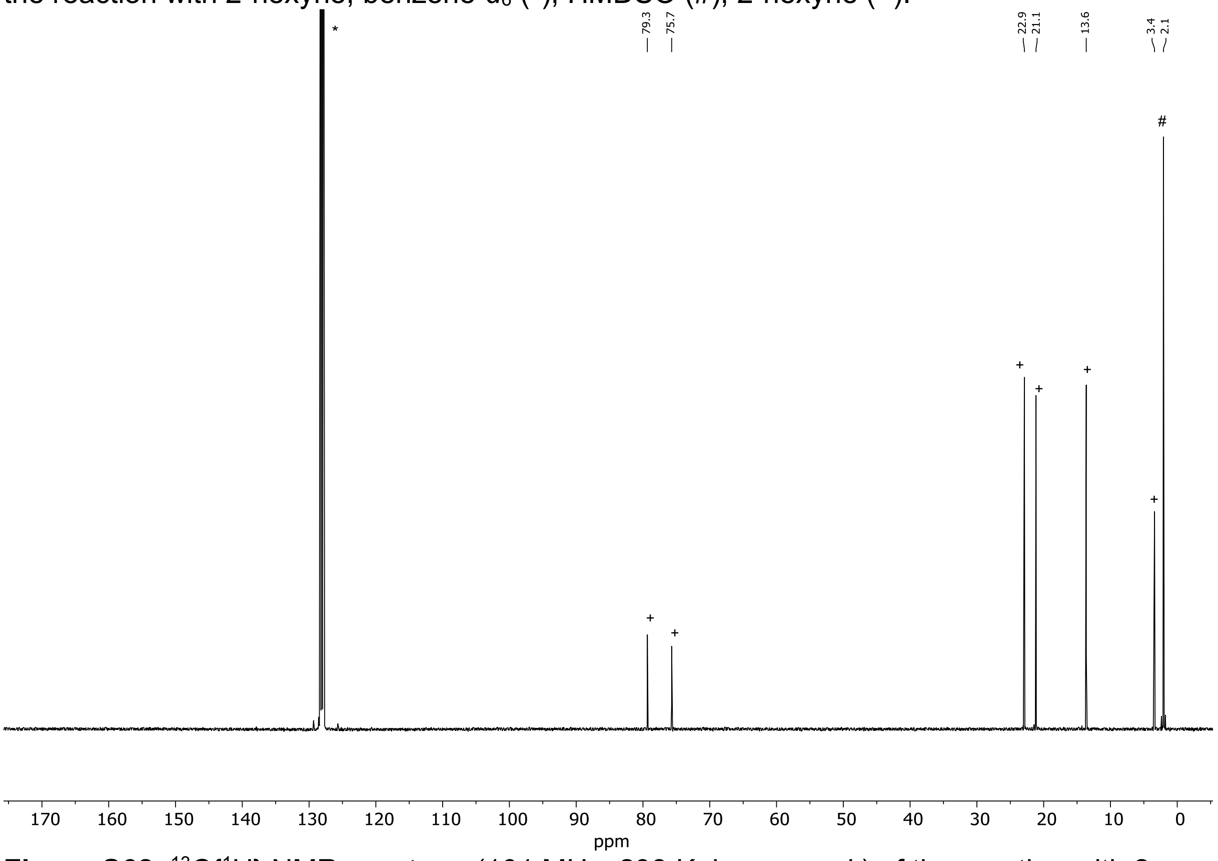

Figure S68. $^{13}C\{^1H\}$ NMR spectrum (101 MHz, 298 K, benzene-d_6) of the reaction with 2-hexyne; benzene-d_6 (*), HMDSO (#), 2-hexyne (+).

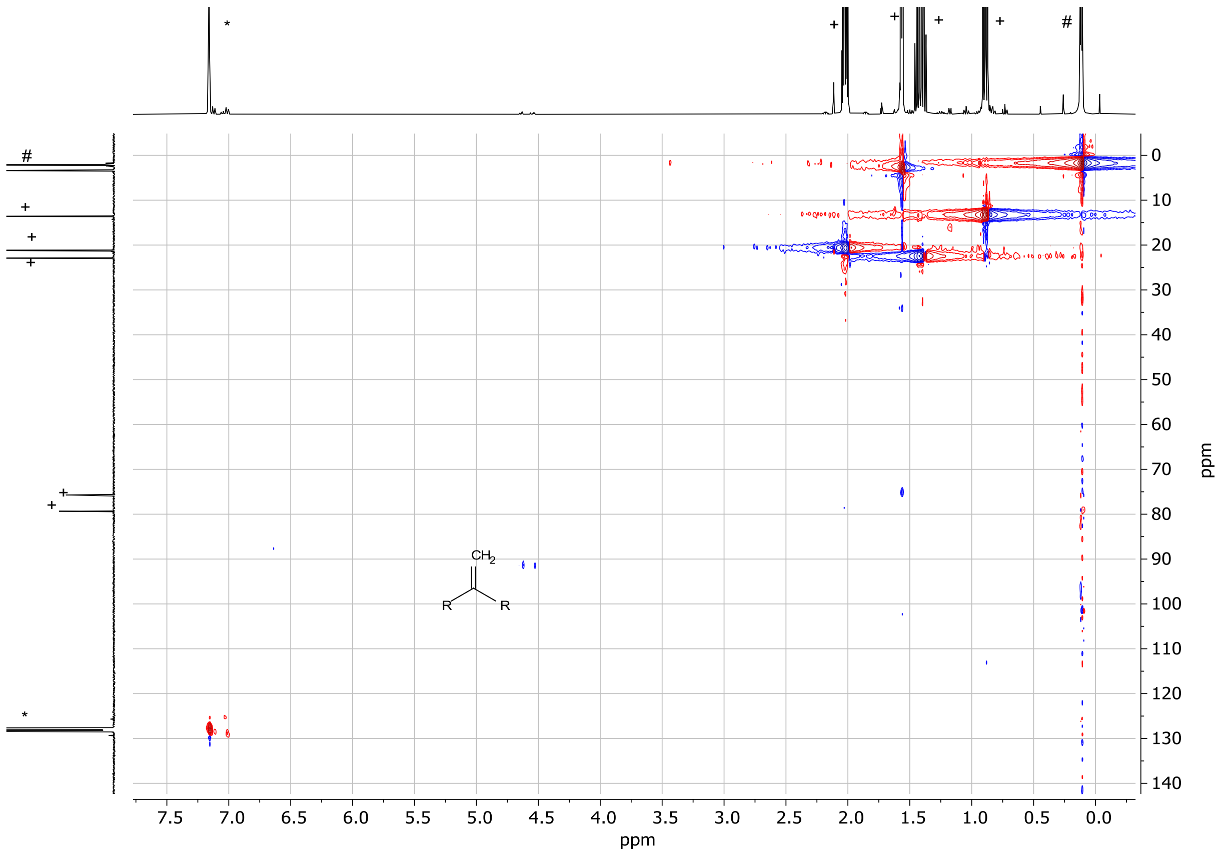

Figure S69. ^{1}H-^{13}C HSQC NMR spectrum (400 MHz, 101 MHz, 298 K, benzene-d_6) of the reaction with 2-hexyne; benzene-d_6 (*), HMDSO (#), 2-hexyne (+).

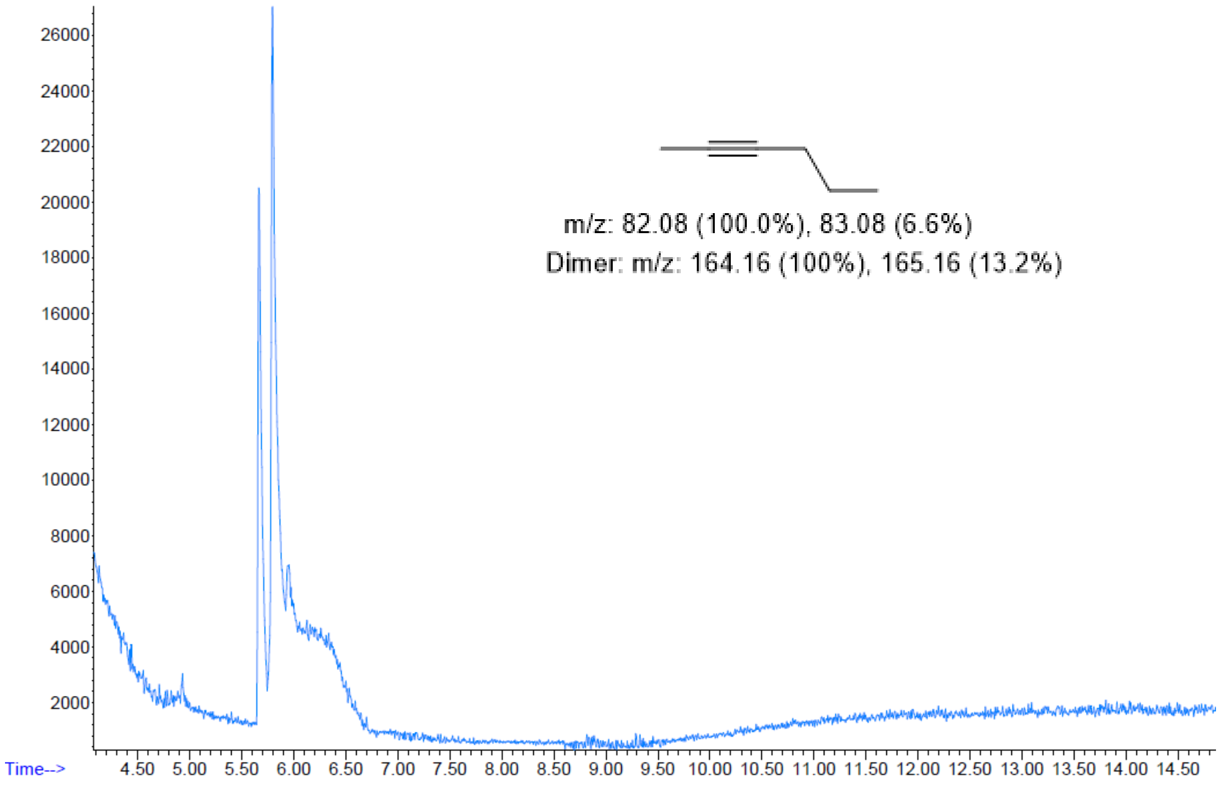

Figure S70. GC-MS chromatogram of the volatiles from the reaction with 2-hexyne.

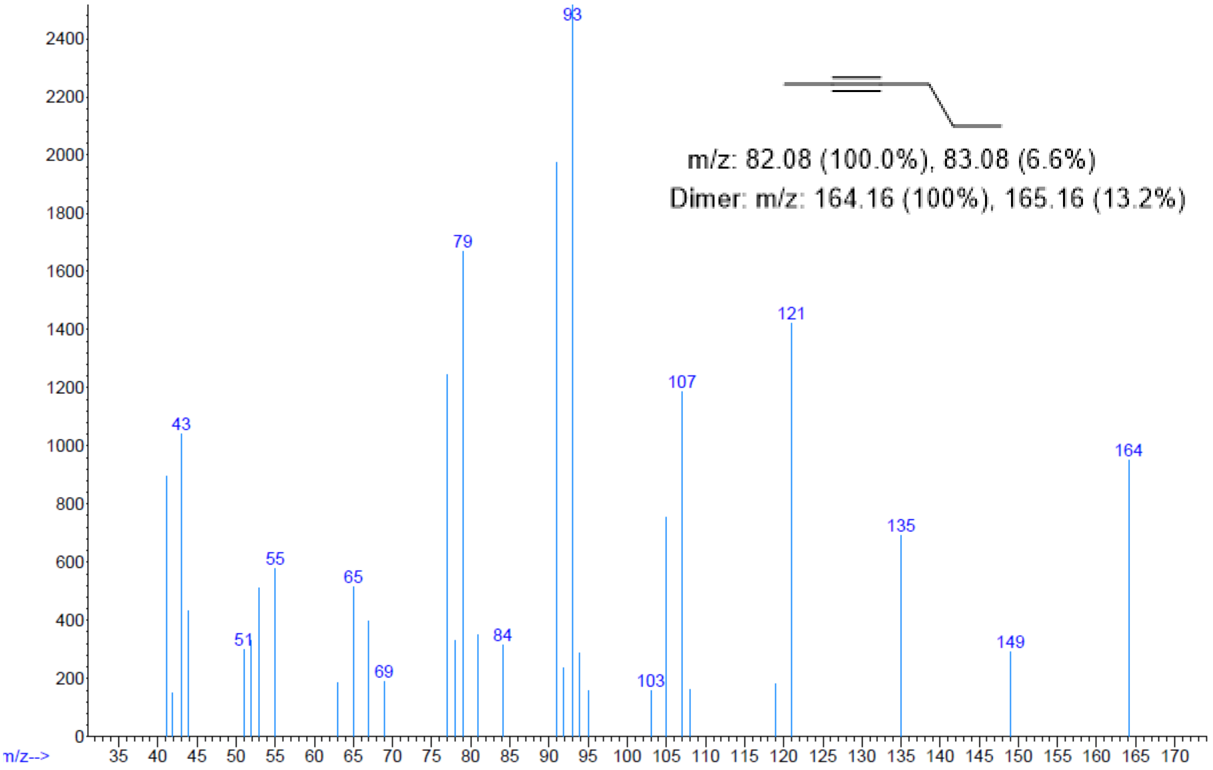

Figure S71. GC-MS spectrum (5.667 min) of the volatiles from the reaction with 2-hexyne.

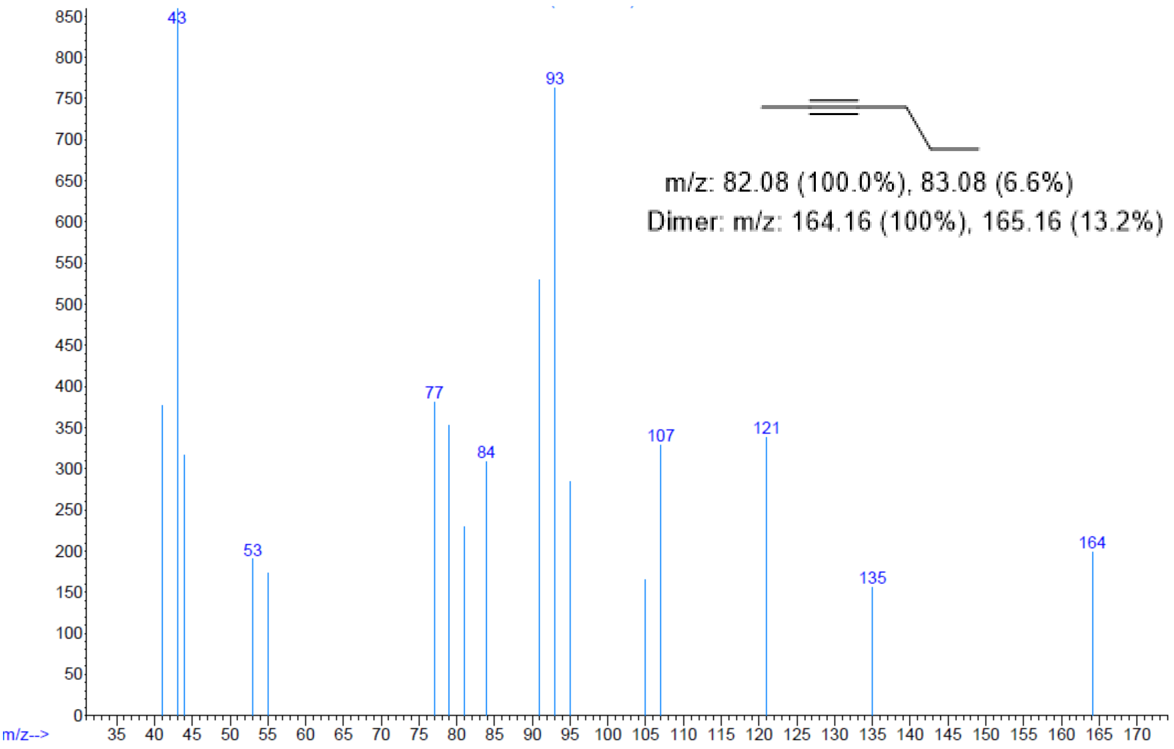

Figure S72. GC-MS spectrum (5.775 min) of the volatiles from the reaction with 2-hexyne.

2-Pentyne

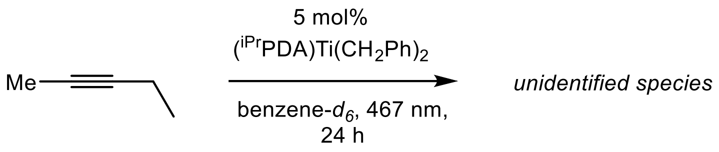

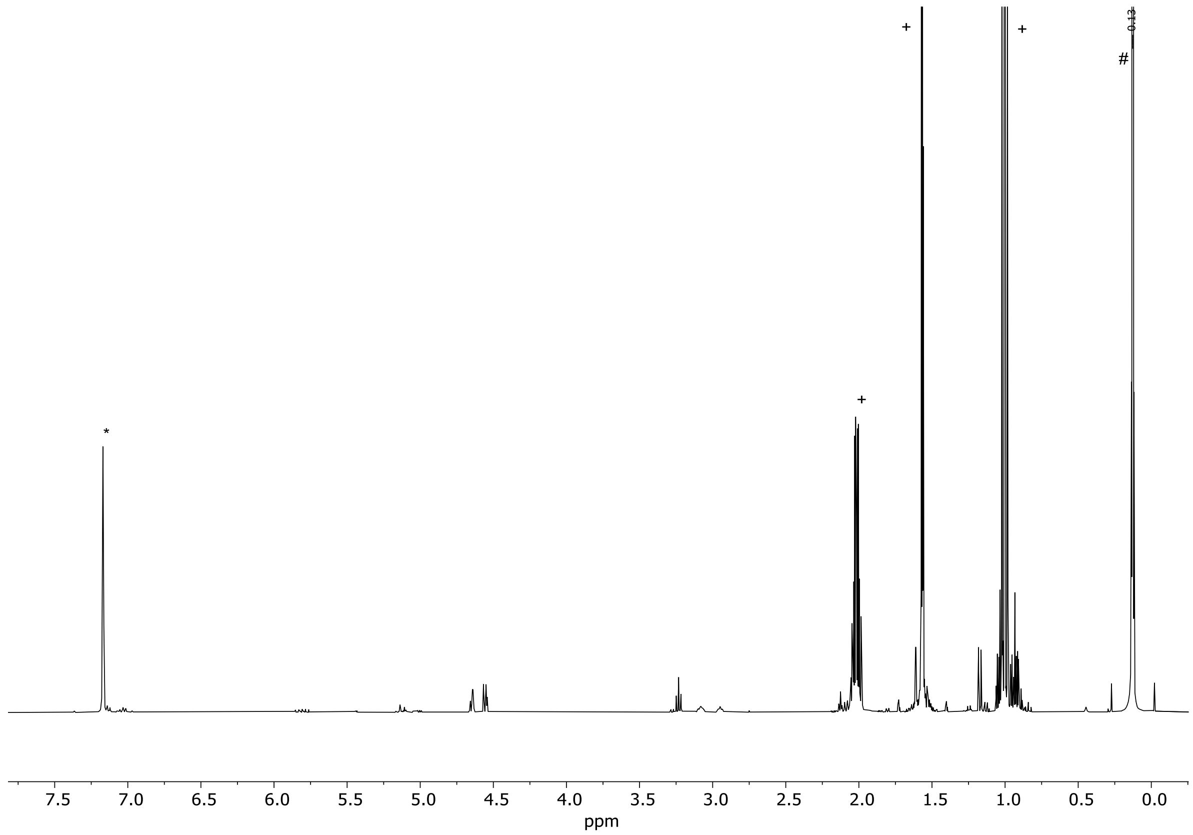

Figure S73. ^{1}H NMR spectrum (400 MHz, 298 K, benzene-d_6) of volatiles of the reaction with 2-pentyne; benzene-d_6 (*), HMDSO (#), 2-pentyne (+).

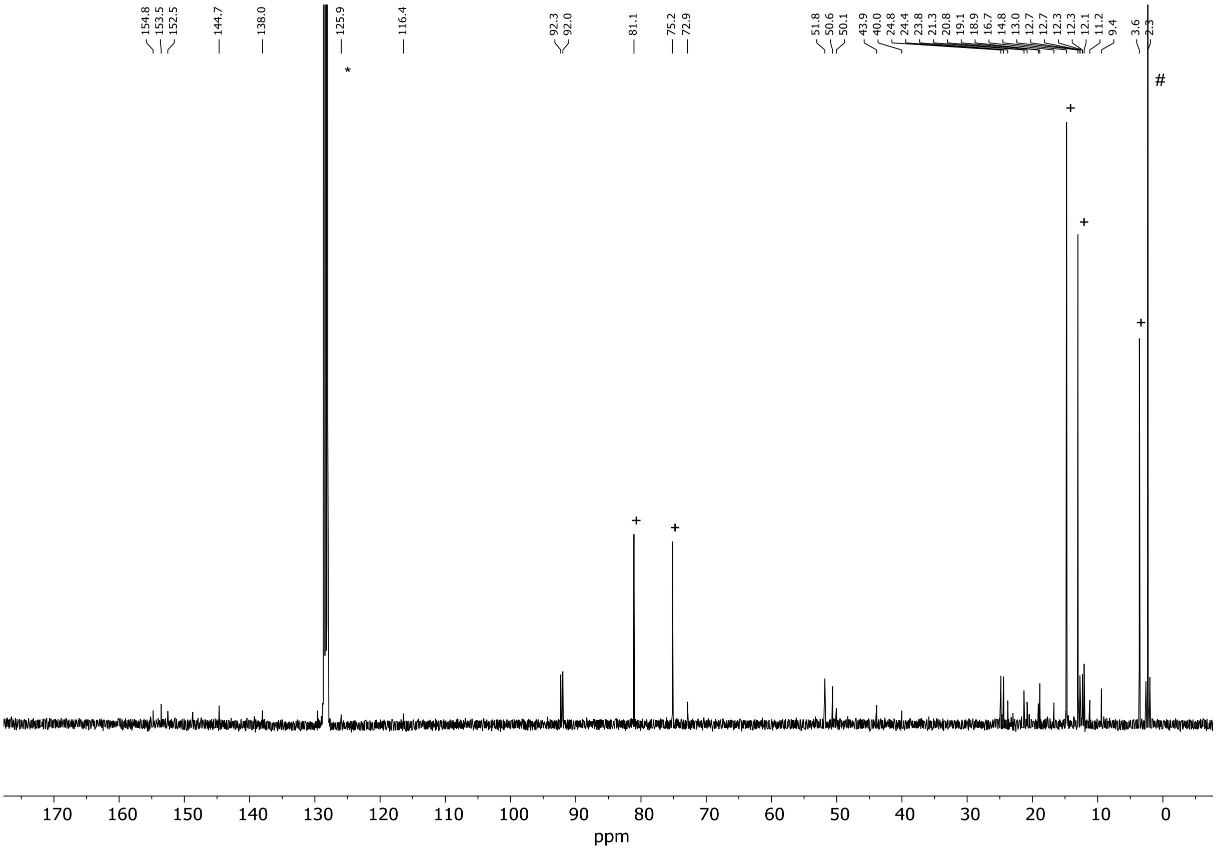

Figure S74. $^{13}C\{^1H\}$ NMR (101 MHz, 298 K, benzene-d_6) of the volatiles of the reaction with 2-pentyne; benzene-d_6 (*), HMDSO (#), 2-pentyne (+).

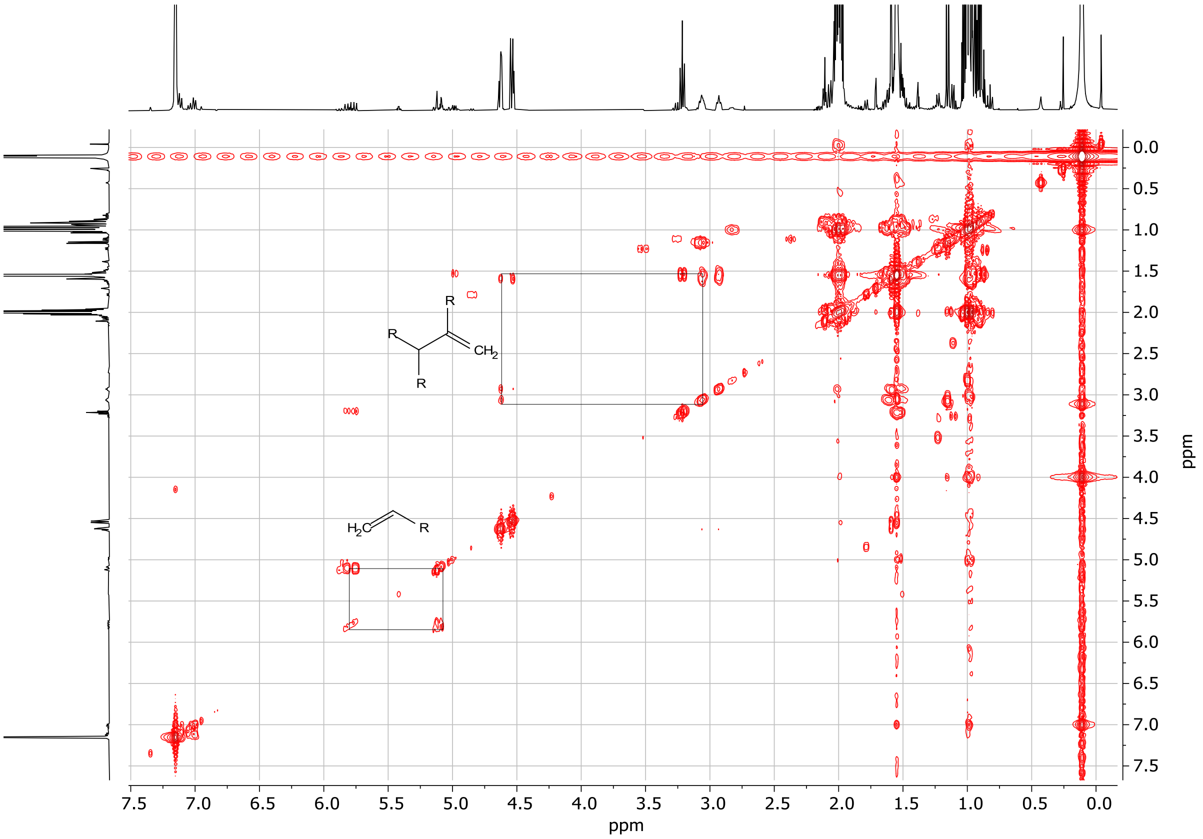

Figure S75. ^1H-^1H COSY NMR spectrum (400 MHz, 298 K, benzene-d_6) of the volatiles of the reaction with 2-pentyne; benzene-d_6 (*), HMDSO (#), 2-pentyne (+).

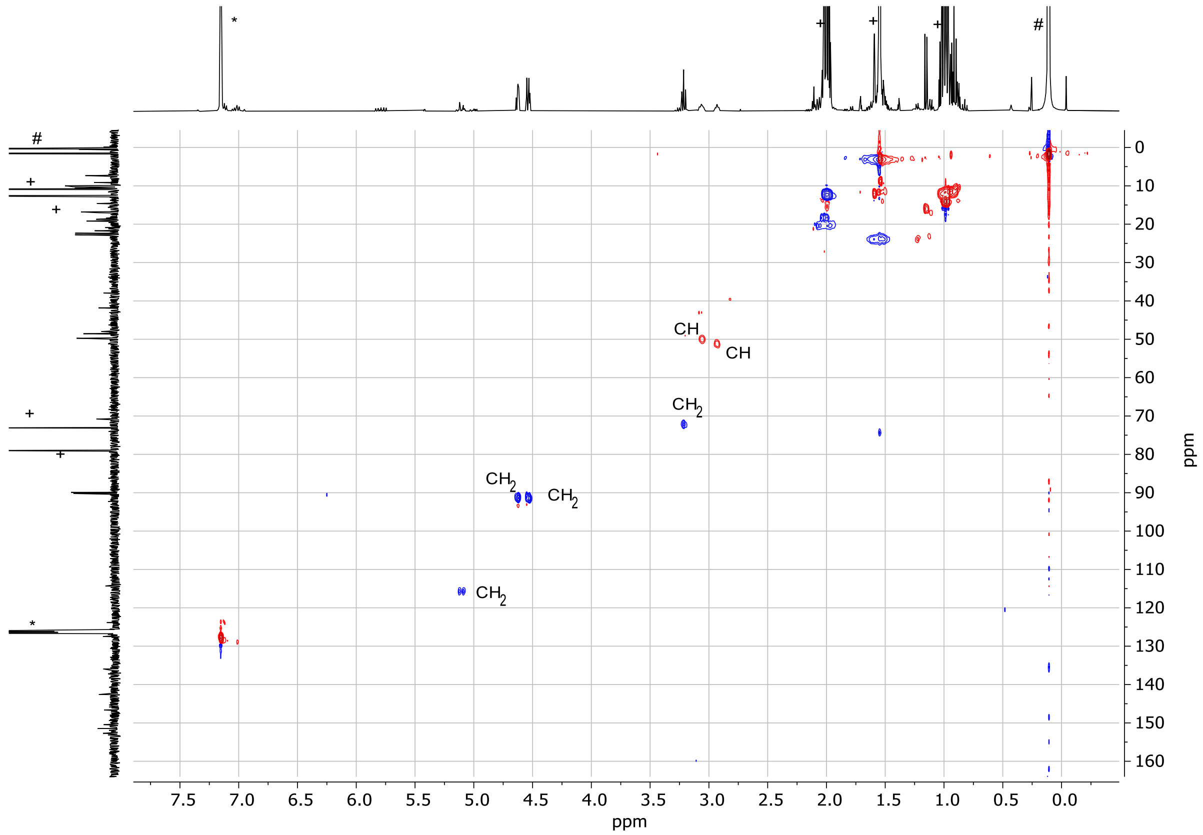

Figure S76. $^{1}H^{13}C$ HSQC NMR spectrum (400 MHz, 298 K, benzene-d_6) of the volatiles of the reaction with 2-pentyne; benzene-d_6 (*), HMDSO (#), 2-pentyne (+).

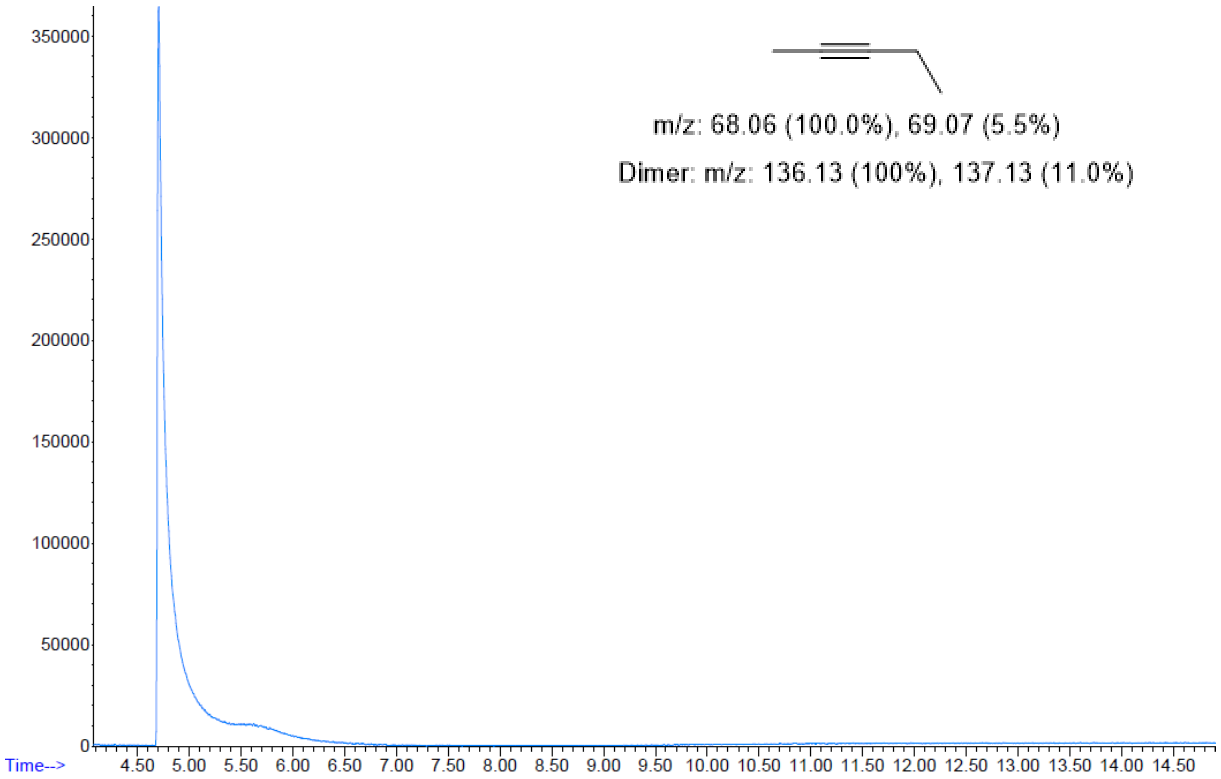

Figure S77. GC-MS spectrum chromatogram of the volatiles of the reaction with 2-pentyne.

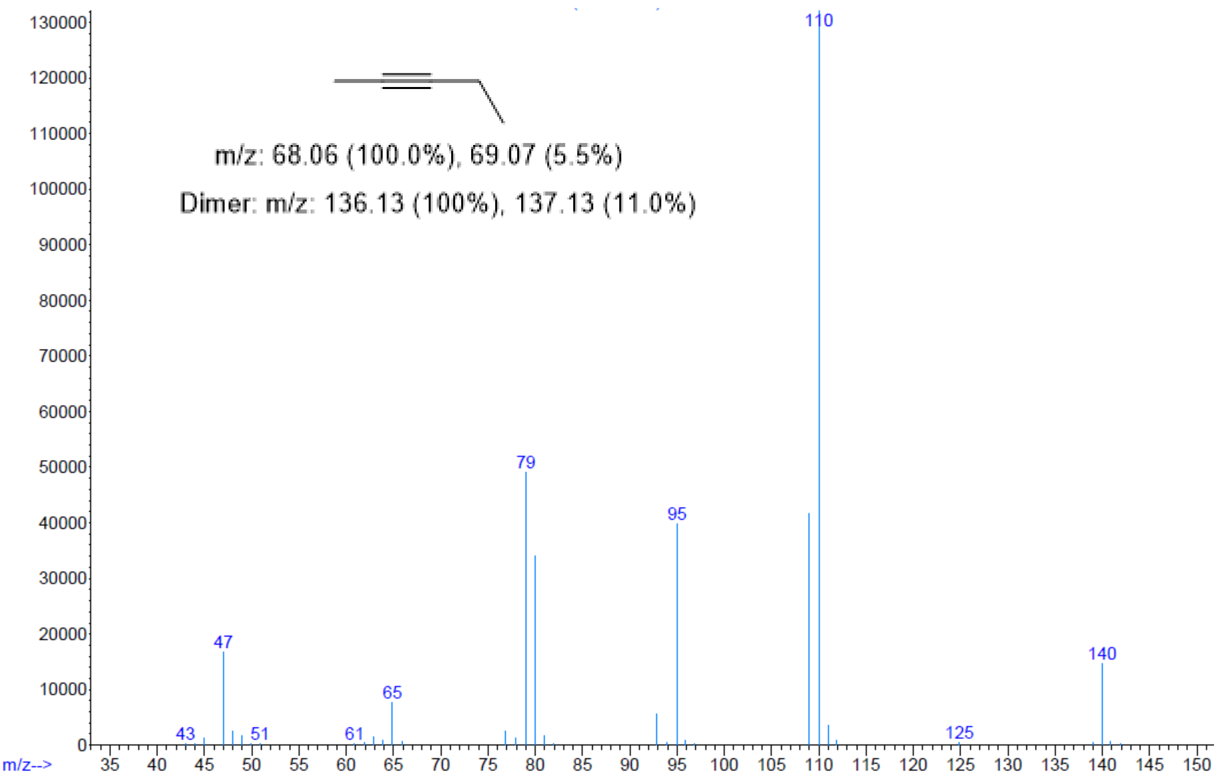

Figure S78. GC-MS spectrum (4.706 min) of the volatiles of the reaction with 2-pentyne.

Summary of alkylic alkynes

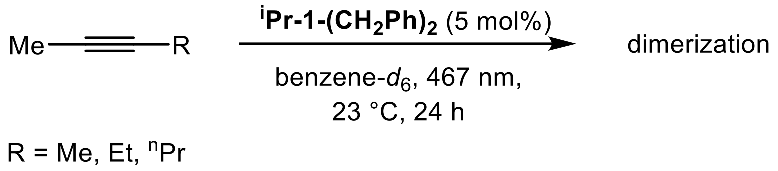

Alkyne	k_{rel}
R = Me (2-butyne)	1.00
R = Et (2-pentyne)	0.58
R = ^{n}Pr (2-hexyne)	0.32
3-hexyne	0.75

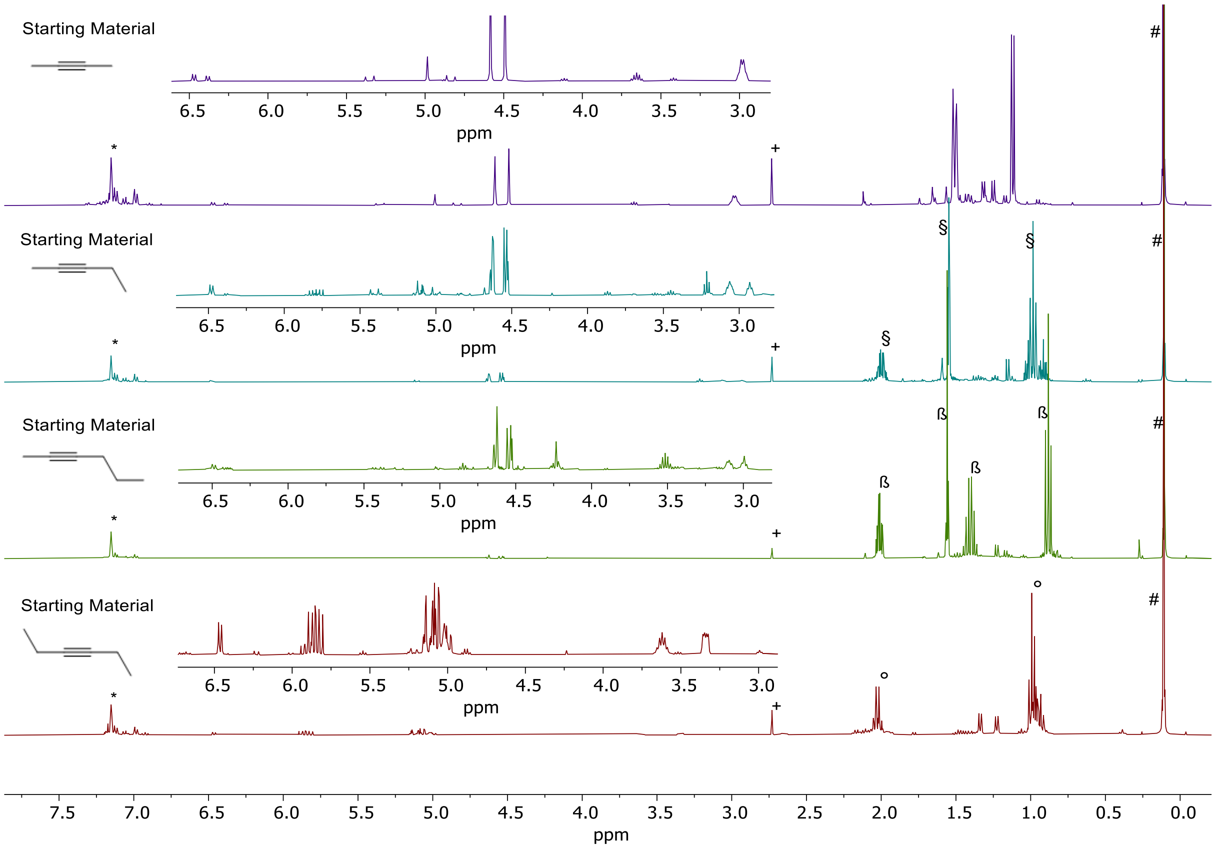

Figure S79. Stacked ^{1}H NMR spectra (400 MHz, 298 K, benzene-d_6) of dimerization of **1**, **2**, **3**, and **4**. The spectra were obtained after the reaction using conditions shown in the table above; benzene-d_6 (*), HMDSO (#), bibenzyl (+), 2-pentyne (§), 2-hexyne (ß), 3-hexyne (°).

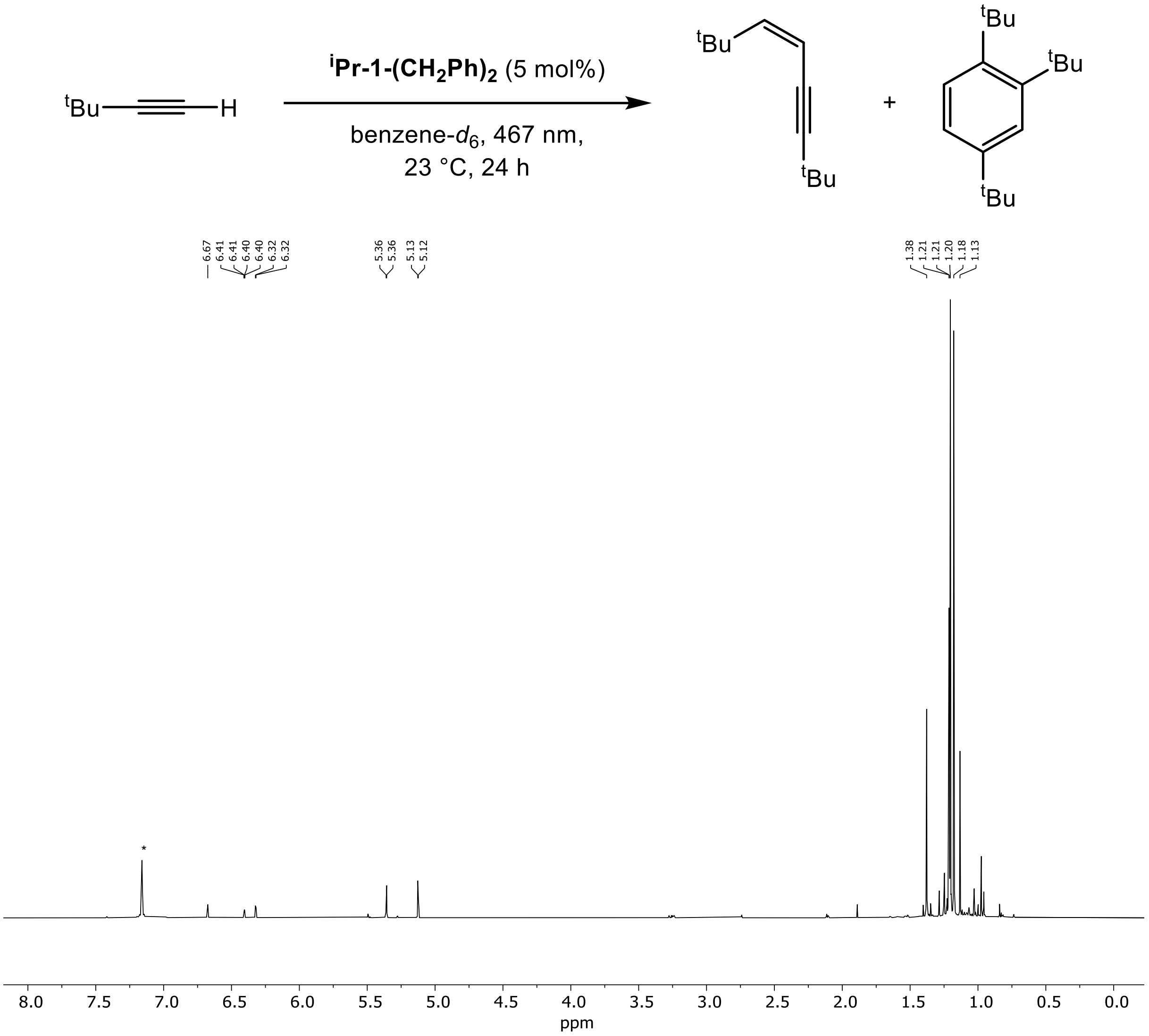

Figure S80. ^{1}H NMR spectrum (400 MHz, 298 K, benzene-d_6) after the reaction with *tert*-butyl acetylene; benzene-d_6 (*).

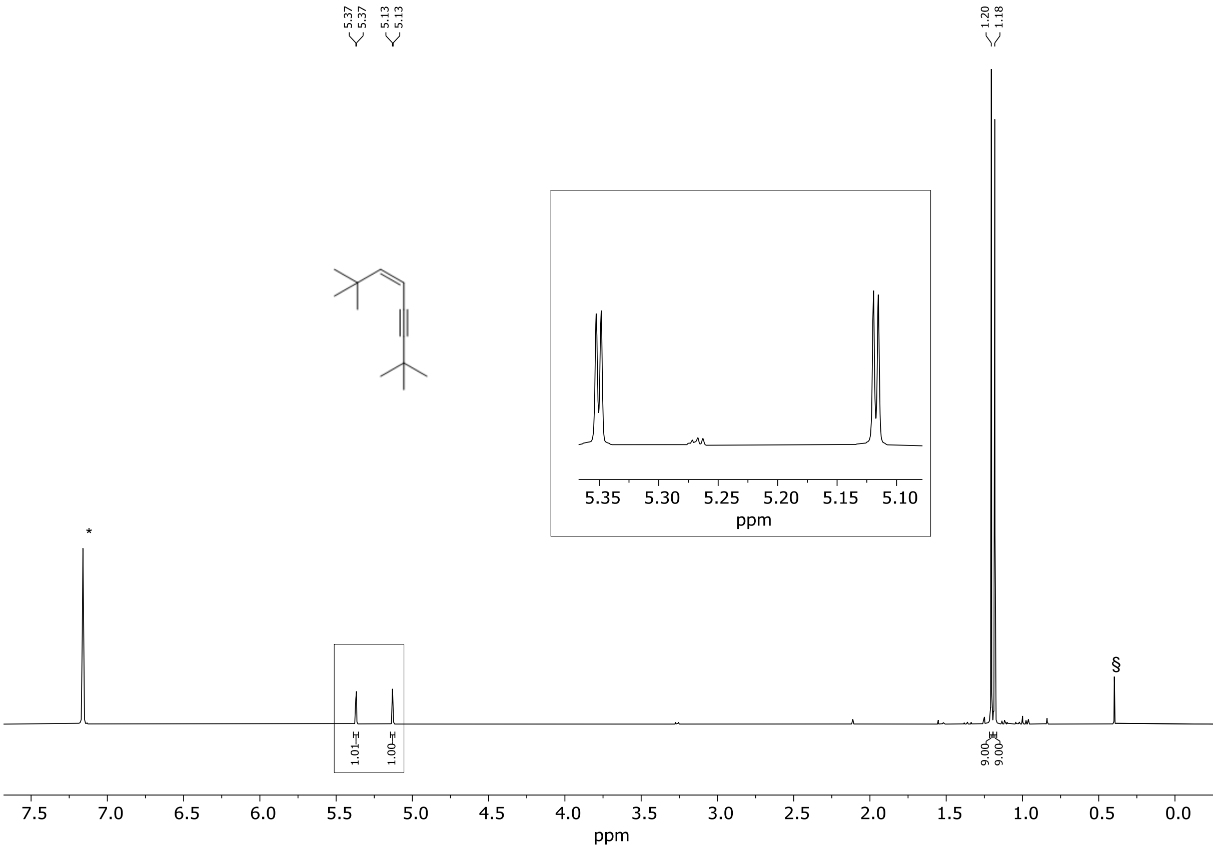

Figure S81. ^{1}H NMR spectrum (400 MHz, 298 K, benzene-*d*$_6$) of *Z*-enyne isolated by condensation under reduced pressure (10^{-2} mbar); benzene-*d*$_6$ (*), grease (§).

Steric influence of the ligand on the conversion

The steric influence of the ligand was tested for the two best performing substrates except 2-butyne.

<u>3-Hexyne:</u>

A J Young NMR tube was charged with (^{Me}PDA)Ti(CH_2Ph)$_2$ (6.9 mg, 12.2 µmol, 5.0 mol%), 3-hexyne (20.0 mg, 0.24 mmol, 1.0 equiv.) and benzene-*d*$_6$ (0.4 mL, 0.6M). HMDSO (10 µL, 47 µmol) was added to quantify the yield and conversion by ^{1}H NMR spectroscopy. The J Young tube was placed in front of a 467 nm Kessil lamp and irradiated for 24 h at room temperature. The conversion was determined by the internal standard (Table S12).

<u>2-Pentyne:</u>

A J Young NMR tube was charged with (^{Me}PDA)Ti(CH_2Ph)$_2$ (8.4 mg, 14.7 µmol, 5.0 mol%), 2-pentyne (20.0 mg, 0.29 mmol, 1.0 equiv.) and benzene-*d*$_6$ (0.5 mL, 0.6M). HMDSO (10 µL, 47 µmol) was added to quantify the yield and conversion by ^{1}H NMR spectroscopy. The J Young tube was placed in front of a 467 nm Kessil lamp and irradiated for 24 h at room temperature. The conversion was determined by the internal standard (Table S12).

Table S12: Conversion of 2-pentyne and 3-hexyne depending on the sterical hindrance.

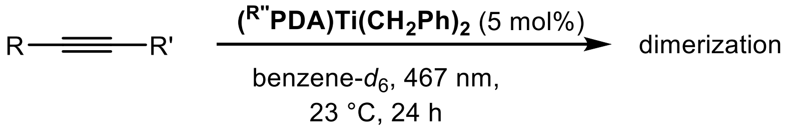

R = Me, Et   R'' = Me, iPr

R' = Et

Alkyne	Titanium complex	conversion
R = Me, R' = Et (2-pentyne)	$(^{Me}PDA)Ti(CH_2Ph)_2$	65%
R = Me, R' = Et (2-pentyne)	$(^{iPr}PDA)Ti(CH_2Ph)_2$	58%
R, R' = Et (3-hexyne)	$(^{Me}PDA)Ti(CH_2Ph)_2$	11%
R, R' = Et (3-hexyne)	$(^{iPr}PDA)Ti(CH_2Ph)_2$	75%

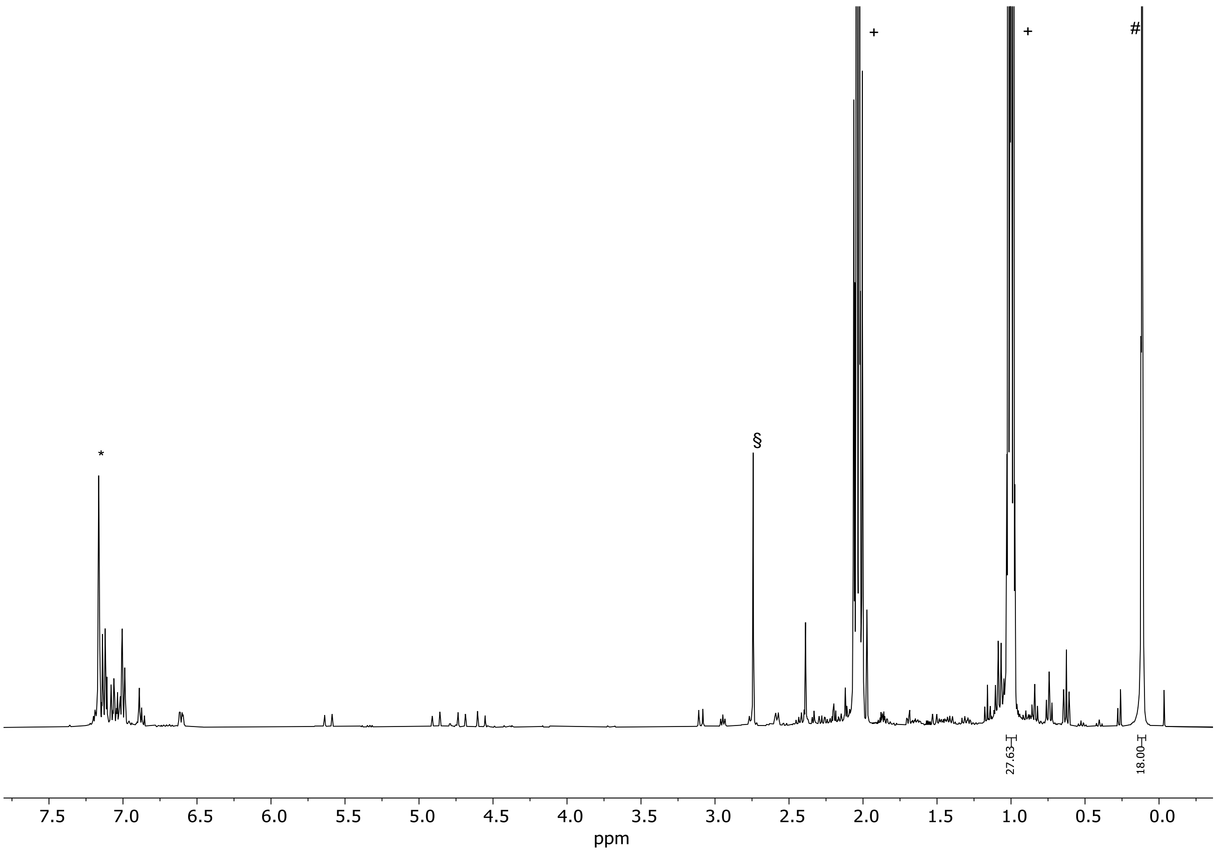

Figure S82. ^{1}H NMR spectrum (400 MHz, 298 K, benzene-d_6) of the reaction with 3-hexyne in the presence of $(^{Me}PDA)Ti(CH_2Ph)_2$ (5 mol%); benzene-d_6 (*), HMDSO (#), 3-hexyne (+), bibenzyl (§).

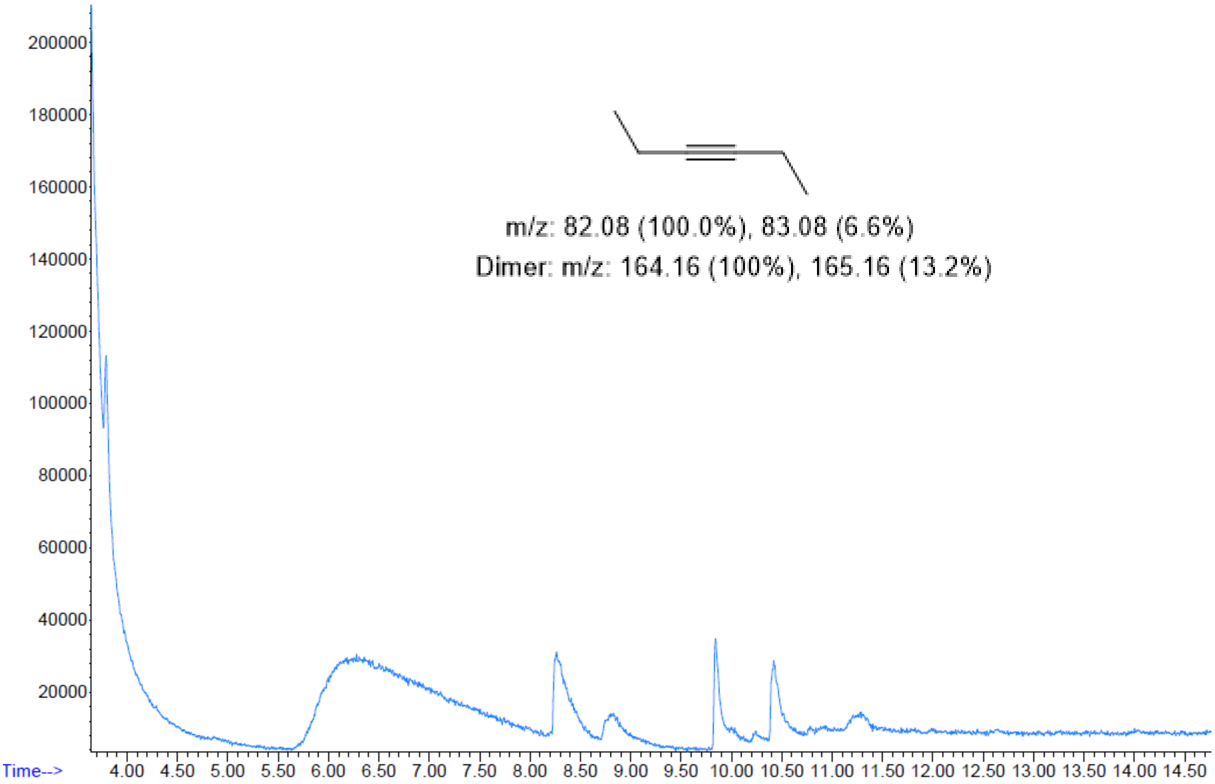

Figure S83. GC-MS chromatogram of the reaction with 3-hexyne and (^{Me}PDA)Ti(CH_2Ph)$_2$. The broad peaks between 8.3 min and 11.2 min result from catalyst decomposition by protonation.

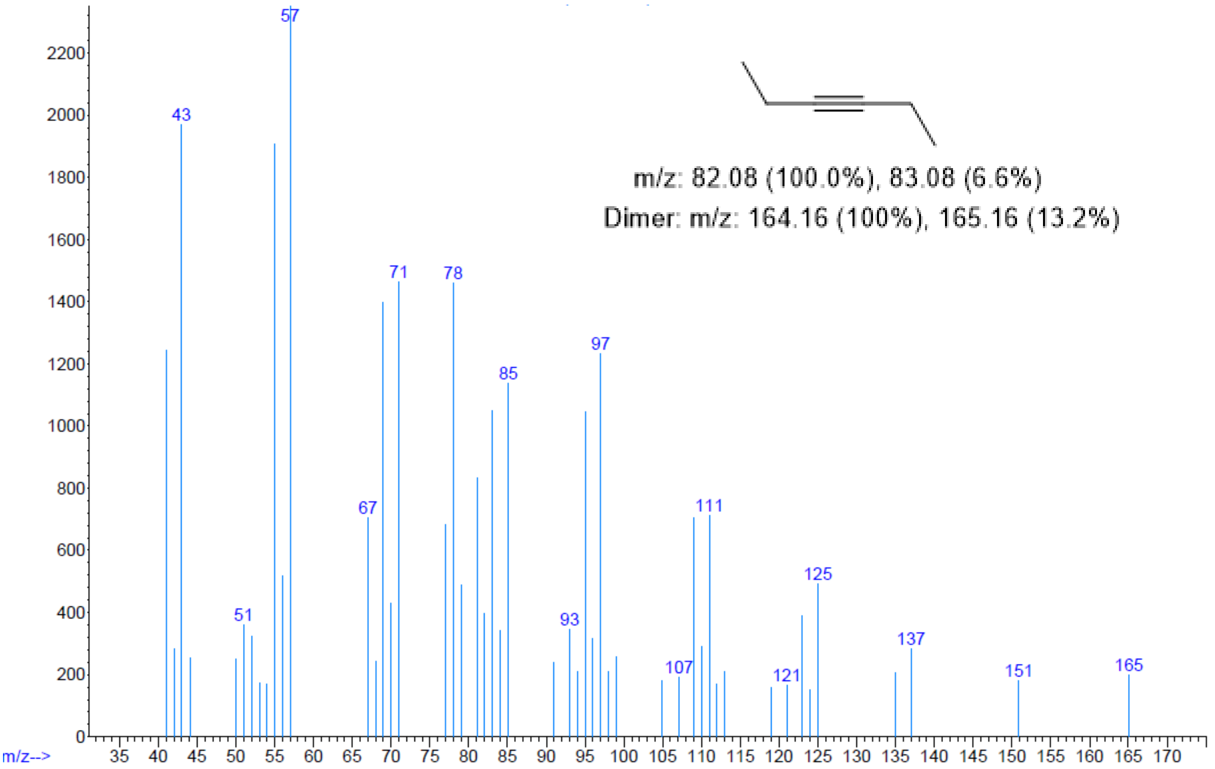

Figure S84. GC-MS spectrum (6.372 min) of the reaction with 3-hexyne and (^{Me}PDA)Ti(CH_2Ph)$_2$.

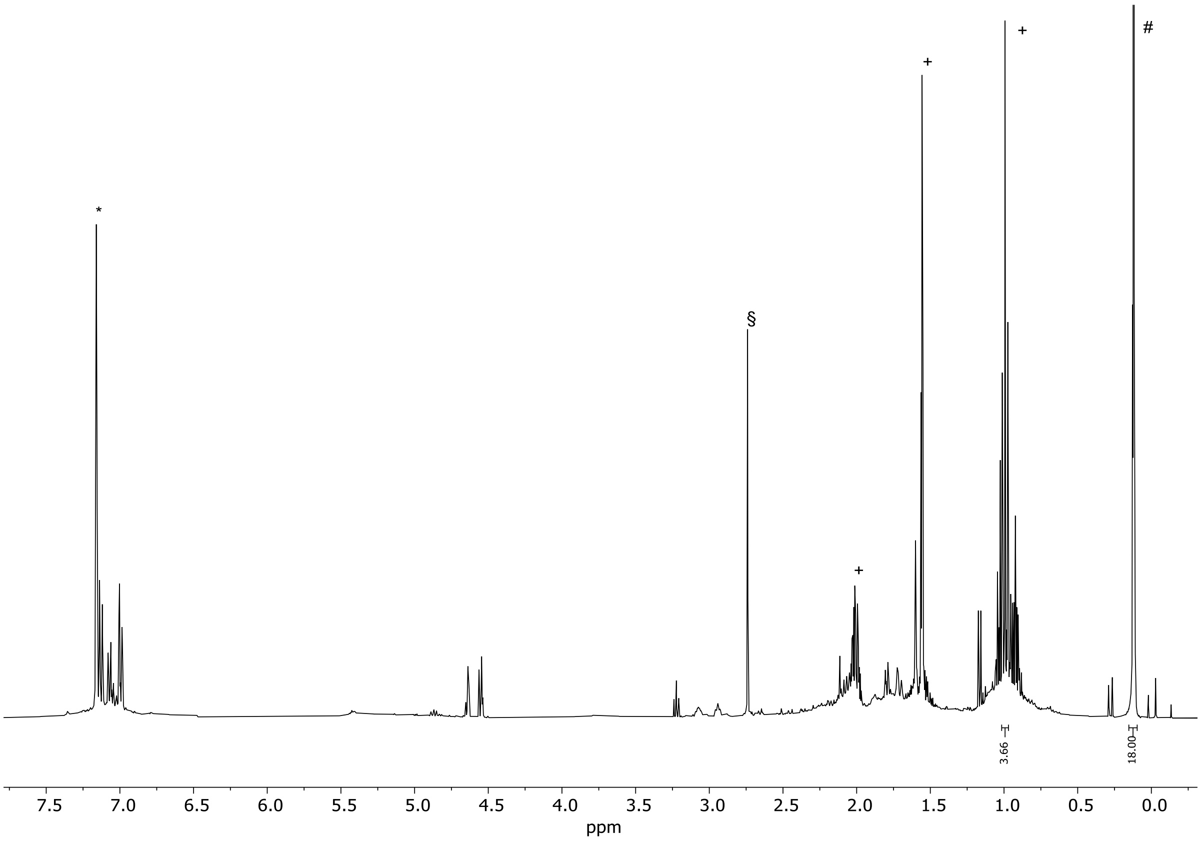

Figure S85. ^{1}H NMR spectrum (400 MHz, 298 K, benzene-d_6) of the reaction with 2-pentyne in the presence of (^{Me}PDA)Ti(CH_2Ph)$_2$ (5 mol%); benzene-d_6 (*), HMDSO (#), 2-pentyne (+), bibenzyl (§).

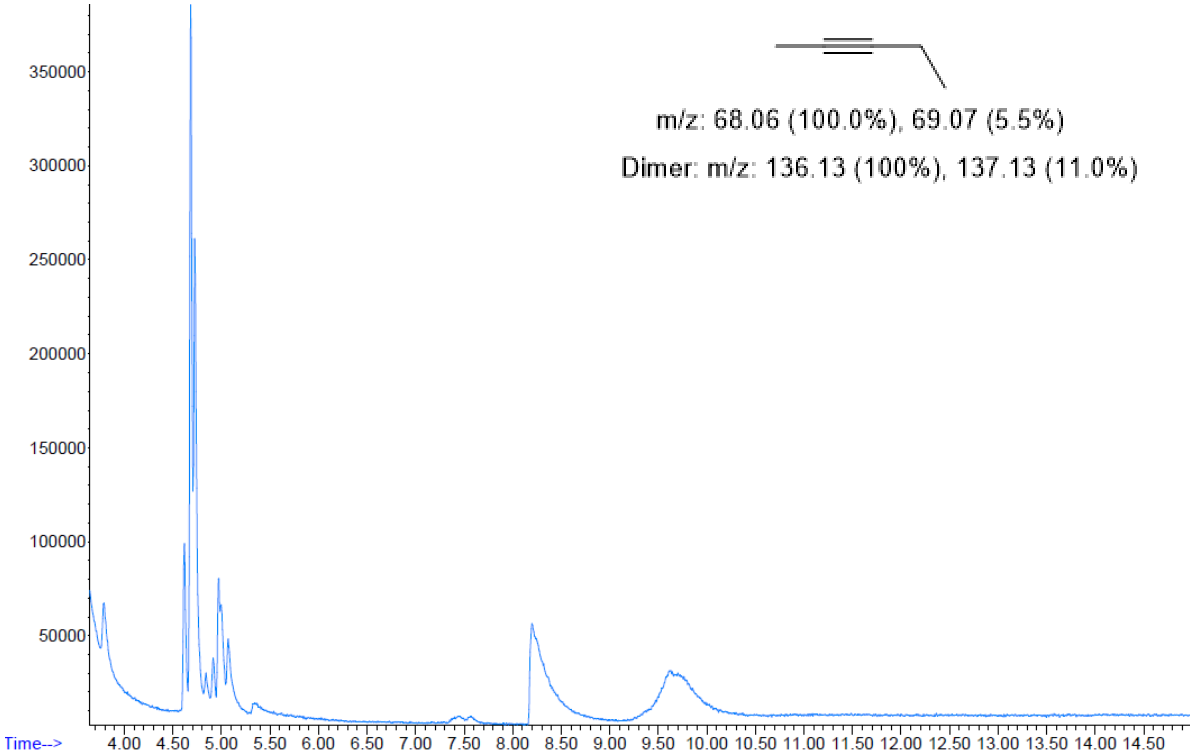

Figure S86. GC-MS chromatogram of the reaction with 2-pentyne and (^{Me}PDA)Ti(CH_2Ph)$_2$. The broad peaks at 8.3 min and 8.7 min result from catalyst decomposition by protonation.

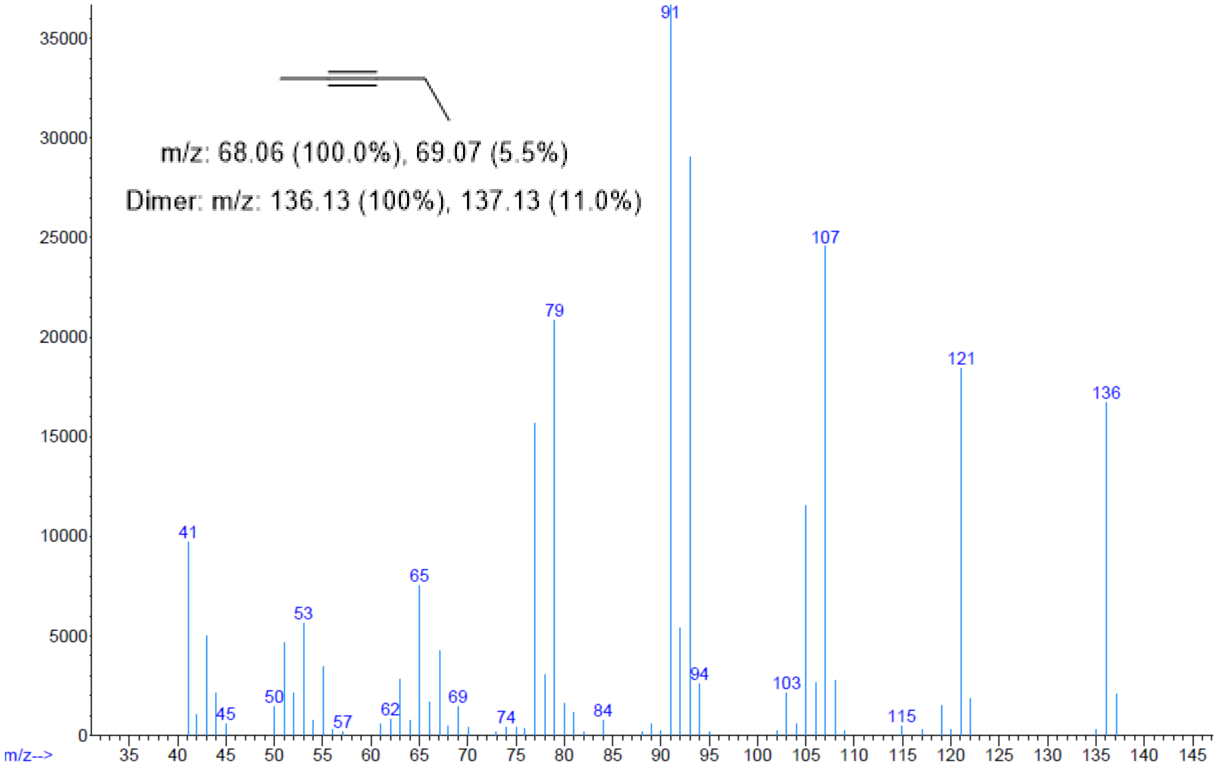

Figure S87. GC-MS spectrum (4.693 min) of the reaction with 2-pentyne and (^{Me}PDA)Ti(CH_2Ph)$_2$.

Cross-dimerization of 2-butyne and 3-hexyne

A J Young NMR tube was charged with (^{iPr}PDA)Ti(CH_2Ph)$_2$ (12.7 mg, 18.5 µmol, 5.0 mol%), 2-butyne (20 mg, 0.37 mmol, 1.0 equiv) and 3-hexyne (20.0 mg, 0.24 mmol, 1.0 equiv.) and benzene-d_6 (0.5 mL, 0.7M). HMDSO (10 µL, 47 µmol) was added to quantify the yield and conversion by ^{1}H NMR spectroscopy. The J Young tube was placed in front of a 467 nm Kessil lamp and irradiated for 24 h at room temperature. The conversion was determined by the internal standard.

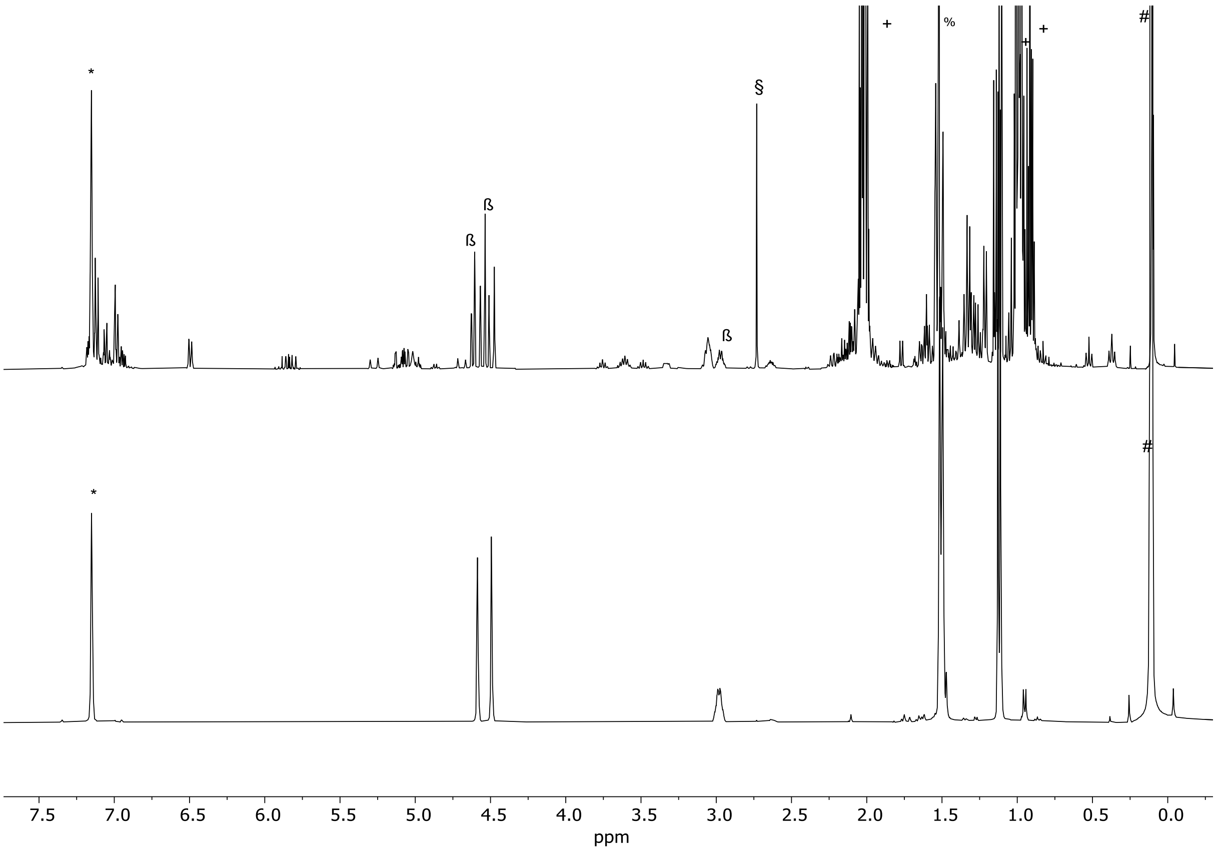

Figure S88. ^{1}H NMR spectrum (400 MHz, 298 K, benzene-d_6) of the reaction of 2-butyne and 3-hexyne in the presence of (^{iPr}PDA)Ti(CH_2Ph)$_2$. **Top:** ^{1}H NMR spectrum of the reaction mixture. **Bottom:** ^{1}H NMR spectrum of isolated 1,2,3-Trimethyl-4-methylenecyclobutene; benzene-d_6 (*), HMDSO (#), 3-hexyne (+), 2-butyne (%), bibenzyl (§), 1,2,3-Trimethyl-4-methylenecyclobutene (ß).

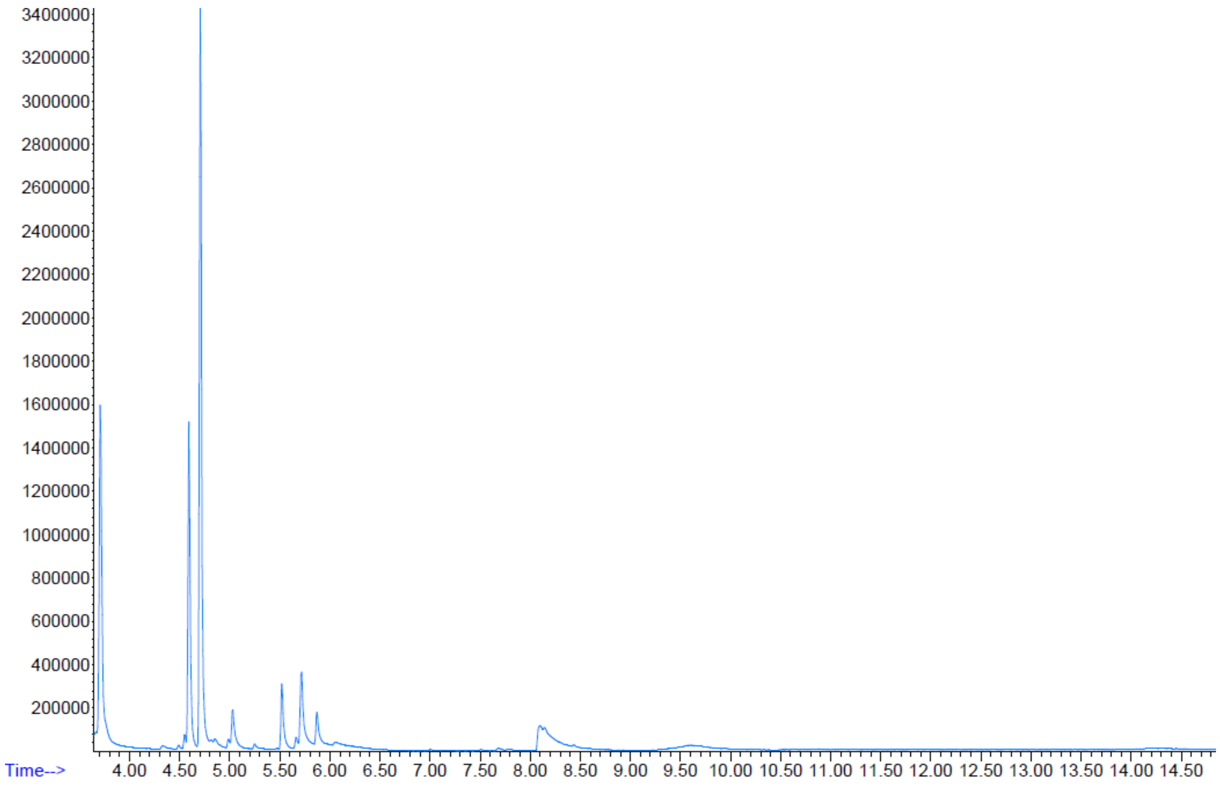

Figure S89. GC-MS chromatogram of the reaction of 2-butyne and 3-hexyne in the presence of (^{iPr}PDA)Ti(CH_2Ph)$_2$.

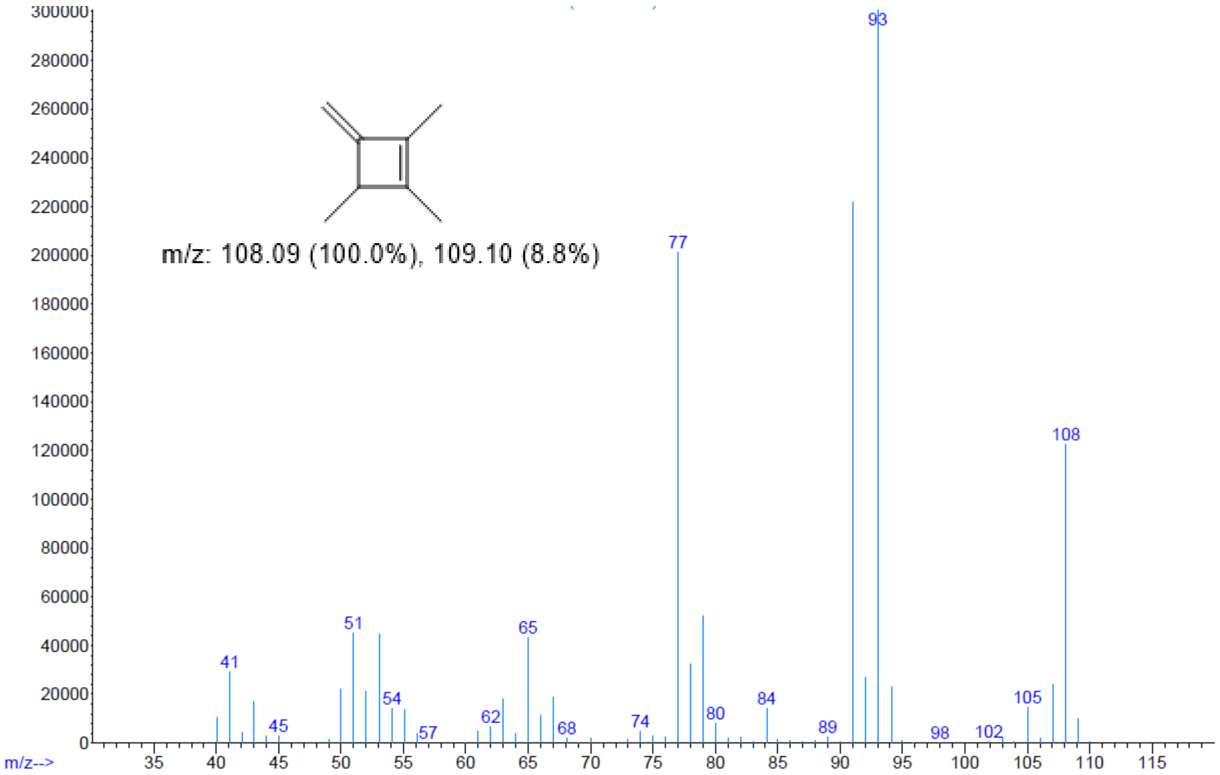

Figure S90. GC-MS spectrum (3.713 min) of the reaction of 2-butyne and 3-hexyne in the presence of (^{iPr}PDA)Ti(CH_2Ph)$_2$. 4

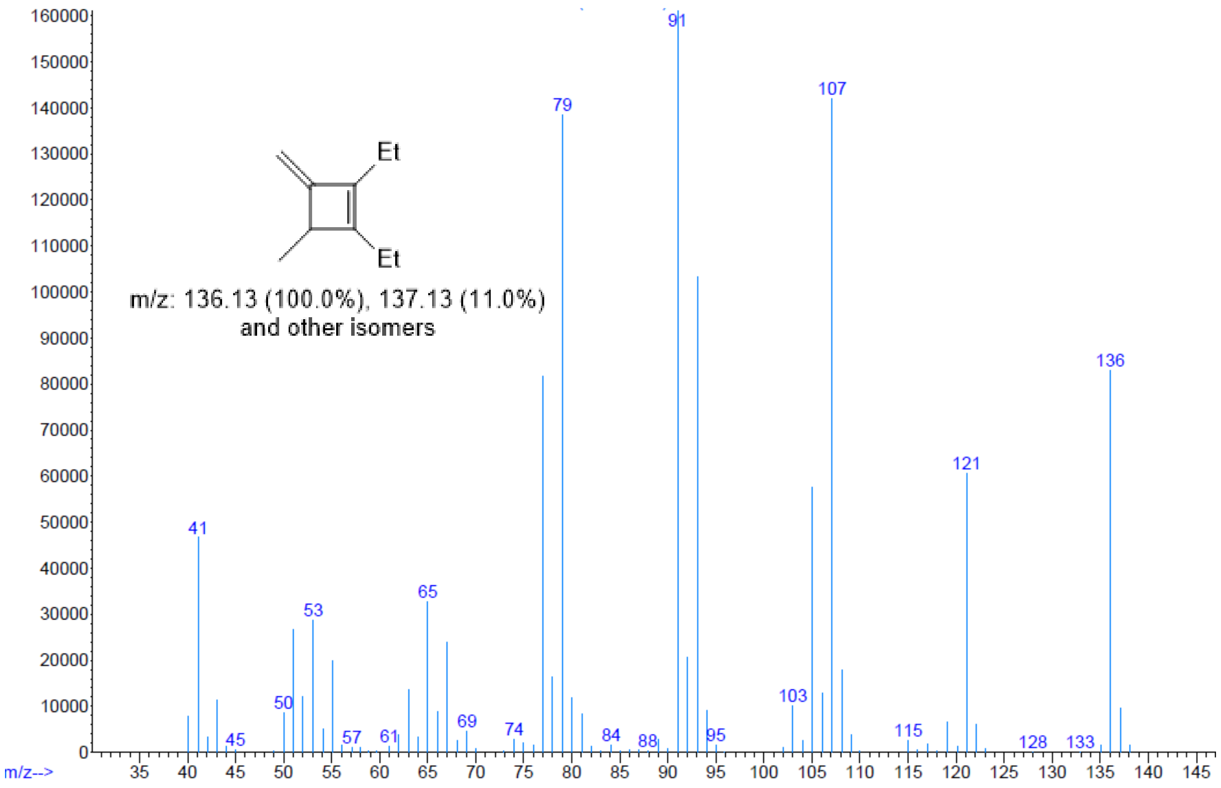

Figure S91. GC-MS spectrum (4.500 to 5.000 min) of the reaction of 2-butyne and 3-hexyne in the presence of (^{iPr}PDA)Ti(CH_2Ph)$_2$.

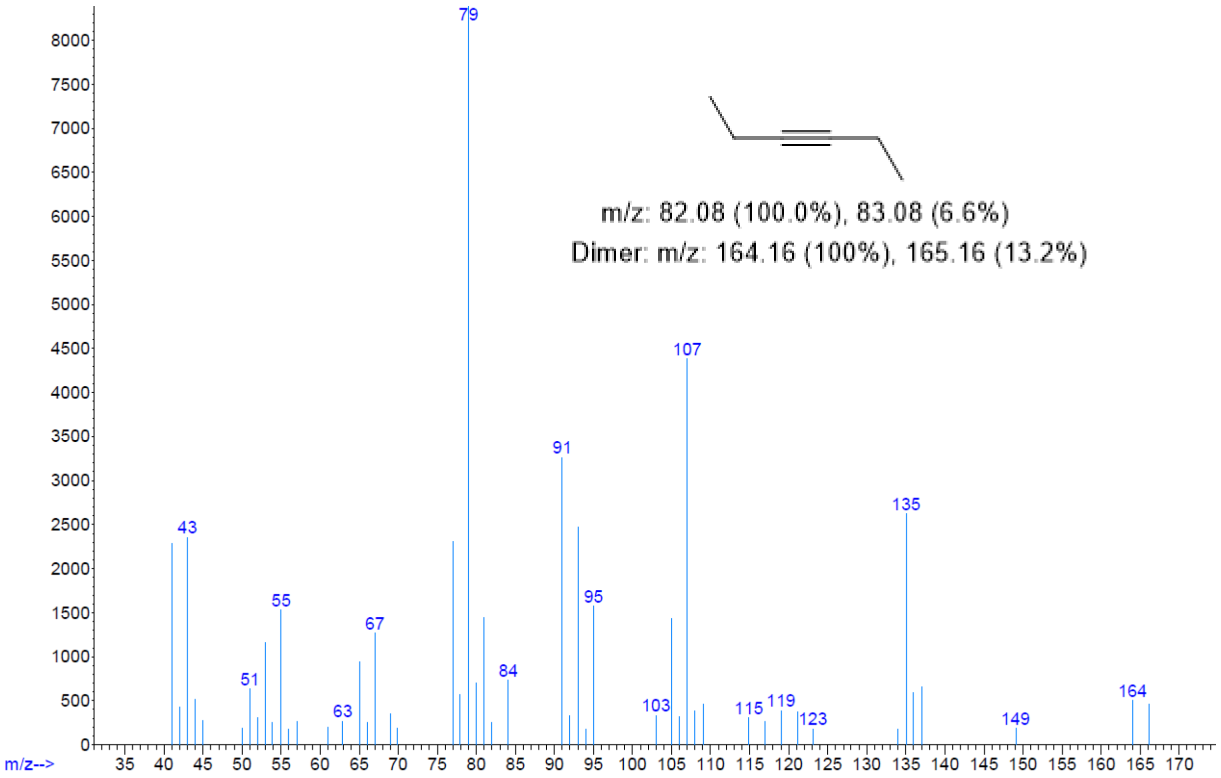

Figure S92. GC-MS spectrum (5.500 to 6.000 min) of the reaction of 2-butyne and 3-hexyne in the presence of (^{iPr}PDA)Ti(CH_2Ph)$_2$.

12. References

[1] U. Zucchini, E. Albizzati, U. Giannini, Synthesis and properties of some titanium and zirconium benzyl derivatives, *J. Organometal. Chem.* **1971**, *26*, 357–372.
[2] Arias, O., Petrov, A. R., Bannenberg, T., Altenburger, K., Arndt, P., Jones, P. G., Tamm, M.; Titanocene and zirconocene complexes with diaminoacetylenes: formation of unusual metallacycles and fulvene complexes, *Organometallics* **2014**, 33, 1774–1786.
[3] N. S. Lambic, R. D. Sommer, E. A. Ison, Tuning catalytic activity in the hydrogenation of unactivated olefins with transition-metaloxos as the Lewis base component of frustrated Lewis pairs, *ACS Catal.* **2017**, *7*, 1170–1180.
[4] Fritsche, P., Geyer, L., Czernetzki, C., Hierlmeier, G., Coordination-induced reductive elimination from a titanium (IV) complex, *Chem. Commun.* **2024**, *60*, 9030–9033.
[5] Guérin, F., McConville, D. H., & Payne, N. C., Conformationally rigid diamide complexes: synthesis and structure of titanium (IV) alkyl derivatives, *Organometallics* **1996**, *15*, 5085–5089.
[6] D. A. Roberts, B. S. Pilgrim, G. Sirvinskaite, T. K. Ronson, J. R. Nitschke, Covalent Post-assembly Modification Triggers Multiple Structural Transformations of a Tetrazine-Edged Fe_4L_6 Tetrahedron, *J. Am. Chem. Soc.* **2018**, *140*, 9616–9623.
[7] M. Amatore, D. Leboeuf, M. Malacria, V. Gandon, C. Aubert, Highly enantioselective rhodium-catalyzed [2+2+2] cycloaddition of diynes to sulfonimines, *J. Am. Chem. Soc.* **2013**, *135*, 4576–4579.
[8] Hüther, H., Brune, H. A., Protonenresonanz-spektroskopische Untersuchungen an substituierten Cyclobutenen. Ein Beitrag zur Kenntnis der Bindungsverhältnisse im komplex-gebundenen Cyclobutadien. *Org. Magn. Reson.* **1971**, *3*, 737–757.
[9] Zhang, Y.; Lee, T. S.; Petersen, J. L.; Milsmann, C. A, Zirconium Photosensitizer with a Long-Lived Excited State: Mechanistic Insight into Photoinduced Single-Electron Transfer. *J. Am. Chem. Soc.* **2018**, *140*, 5934–5947.
[10] Wegner, E. E., Adamson, A. W., Photochemistry of complex ions. III. Absolute quantum yields for the photolysis of some aqueous chromium (III) complexes. Chemical actinometry in the long wavelength visible region. *J. Am. Chem. Soc.* **1986**, *88*, 394–404.
[11] Ji, Y., DiRocco, D. A., Kind, J., Thiele, C. M., Gschwind, R. M., Reibarkh, M., LED-Illuminated NMR spectroscopy: a practical tool for mechanistic studies of photochemical reactions. *ChemPhotoChem* **2019**, *3*, 984–992.
[12] Neese, F., Software update: The ORCA program system—Version 5.0. *Wiley Interdisciplinary Reviews: Computational Molecular Science* **2022**, *12*, e1606.
[13] Becke, A. D., Density functional calculations of molecular bond energies, *J. Chem. Phys.* **1986**, *84*, 4524–4529.
[14] (a) V. N. Staroverov, G. E. Scuseria, J. Tao and J. P. Perdew, Comparative assessment of a new nonempirical density functional: Molecules and hydrogen-bonded complexes, *J. Chem. Phys.* **2003**, 119, 12129. (b) J. M. Tao, J. P. Perdew, V. N. Staroverov, and G. E. Scuseria, Climbing the density functional ladder: Nonempirical meta-generalized gradient approximation designed for molecules and solids, *Phys. Rev. Lett.* **2009**, *91*,146401.
[15] (a) Schäfer, A., Horn, H. Ahlrichs, R., Fully optimized contracted Gaussian basis sets for atoms Li to Kr., *J. Chem. Phys.* **1992**, *97*, 2571–2577. (b) Schäfer, A., Huber, C., Ahlrichs, R. Fully optimized contracted Gaussian basis sets of triple zeta valence quality for atoms Li to Kr. *J. Chem. Phys.* **1994**, *100*, 5829–5835. (c) Weigend, F. Ahlrichs, R. Balanced basis sets of split valence, triple zeta valence and quadruple zeta valence quality for H to Rn: design and assessment of accuracy. *Phys. Chem. Chem. Phys.* **2005**, *7*, 3297–305.
[16] (a) Neese, F., Wennmohs, F., Hansen, A. Becker, U., Efficient, approximate and parallel Hartree-Fock and hybrid DFT calculations. A 'chain-of-spheres' algorithm for the HartreeFock exchange. *Chem. Phys.* **2009**, *356*, 98–109. (b) Kossmann, S., Neese, F. Comparison of two efficient approximate Hartee-Fock approaches. *Chem. Phys. Lett.* **2009**, *481*, 240–243. (c) Neese, F., An Improvement of the Resolution of the Identity Approximation for the Formation of the Coulomb Matrix, *J. Comput. Chem.* **2003***, 24*, 1740–1747.

[17] Sheldrick, G. M. SADABS, Bruker AXS, Madison, USA, **2007**.

[18] CrysAlisPro, Scale3 Abspack, Rigaku Oxford Diffraction, **2019**.

[19] Sheldrick, G. M. SHELXT – Integrated space-group and crystal-structure determination. *Acta Cryst. A* **2015**, *71*, 3–8.

[20] Sheldrick, G. M. Crystal structure refinement with SHELXL. *Acta Cryst. C* **2015**, *71*, 3–8.

[21] Sheldrick, G. M. A short history of SHELX. *Acta Cryst. A* **2008**, *64*, 112–122.

[22] Dolomanov, O. V.; Bourhis, L. J.; Gildea, R. J.; Howard, J. A. K.; Puschmann, H. OLEX2: A complete structure solution, refinement and analysis program. *J. Appl. Crystallogr.* **2009**, 42, 339–341.

[23] Enyne: Alos, J., Bolano, T., Esteruelas, M. A., Olivan, M., Onate, E., Valencia, M., POP-Pincer Osmium-Polyhydrides: Head-to-Head (Z)-Dimerization of Terminal Alkynes, *Inorg. Chem.* **2013**, *52*, 6199–6213. 1,2,4-*tert*-butylbenzene: Brenna, D., Villa, M., Gieshoff, T. N., Fischer, F., Hapke, M., & Jacobi von Wangelin, A., Iron-Catalyzed Cyclotrimerization of Terminal Alkynes by Dual Catalyst Activation in the Absence of Reductants, *Angew. Chem. Int. Ed.* **2017**, *56*, 8451–8454.
